# Acceptorless cross-dehydrogenative coupling for C(sp^3^)-H heteroarylation mediated by a heterogeneous GaN/ketone photocatalyst/photosensitizer system

**DOI:** 10.1038/s42004-023-00947-w

**Published:** 2023-09-01

**Authors:** Hyotaik Kang, Lida Tan, Jing-Tan Han, Chia-Yu Huang, Hui Su, Aleksei Kavun, Chao-Jun Li

**Affiliations:** Department of Chemistry, FRQNT Centre for Green Chemistry and Catalysis, McGill University, 801 Sherbrooke Street W., Montréal, Québec H3A0B8 Canada

**Keywords:** Heterogeneous catalysis, Synthetic chemistry methodology, Photocatalysis, Synthetic chemistry methodology

## Abstract

Alkanes are naturally abundant chemical building blocks that contain plentiful C(*sp*^3^)-H bonds. While inert, the activation of C(*sp*^3^)-H via hydrogen atom abstraction (HAT) stages an appealing approach to generate alkyl radicals. However, prevailing shortcomings include the excessive use of oxidants and alkanes that impede scope. We herein show the use of gallium nitride (GaN) as a non-toxic, recyclable, heterogeneous photocatalyst to enable alkyl C(*sp*^*3*^)-H in conjunction with the catalytic use of simple photosensitizer, benzophenone, to promote the desired alkyl radical generation. The dual photocatalytic cycle enables cross-dehydrogenative Minisci alkylation under mild and chemical oxidant-free conditions.

## Abstract (web summary)

Cross-dehydrogenative coupling is an efficient route for C–C bond formation by direct C–H functionalization, however, this protocol usually requires stoichiometric oxidants under harsh reaction conditions. Here, the authors report a cross-dehydrogenative Minisci-alkylation using a heterogeneous Rh_2_O_3_/GaN photocatalyst and a benzophenone photosensitizer under oxidant-free conditions.

## Introduction

**Fig. 1:**
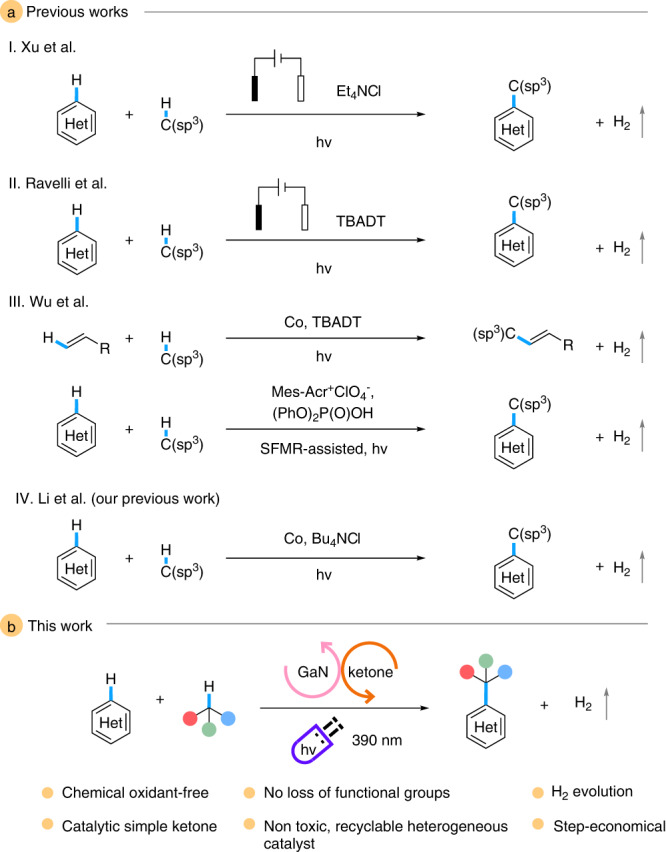
Chemical oxidant-free CDC pathways. **a** Previous chemical oxidant-free CDC works. I Work of Xu et al. on external oxidant-free, photochemical dehydrogenative cross-coupling. II Work of Ravelli et al. on external oxidant-free, photochemical dehydrogenative cross-coupling. III Works of Wu et al. on tetra-*n*-butylammonium decatundstate (TBADT) and cobaloxime-mediated and stop-flow microtubing reactor (SFMR)-assisted dehydrogenative cross-coupling. IV Work of Li et al. on cobalt-catalyzed dehydrogenative cross-coupling. **b** The work reported herein.

With many sectors of industries becoming more environmentally conscious and seeking more sustainable alternatives, there has been a propensity for greener chemistry^1,2^. In response, modern synthetic chemistry developments emphasize atom- and step-economies, core principles of green chemistry^3–6^. Within this domain, cross-dehydrogenative coupling (CDC) stands out as one of the most sustainable and efficient routes for the formation of C-C bonds by direct C-H functionalization and formal loss of H_2_^7–11^. The use of inert C(sp^3^)-H bond is a well-established challenge that remains desirable due to its omnipresence in nature^12,13^. Generally, stoichiometric oxidants such as peroxide or persulfate were involved in CDC protocols for effective alkane activation, representing a major shortcoming of harsh reaction conditions with elevated temperatures^14–16^. Of late, radical-mediated methodologies in photo- and electrochemistry have shown considerable promise in C-H activation under milder reaction conditions^17–24^. Examples include reports by Xu et al. and Ravelli et al. replacing oxidants with electricity in the presence of different hydrogen atom transfer (HAT) reagents in an elegant photoelectrochemical fashion (Fig. 1a I and II)^25,26^. Works by Wu et al. introduce tetra-*n*-butylammonium decatundstate and cobaloxime-mediated hydrogen evolution cross-coupling and an efficient stop-flow microtubing reactor-assisted system where the acid plays a dual function of activating the heterocycle and promoting the HAT process (Fig. 1a III)^27,28^. Our group has also made a recent contribution with cobalt-catalyzed Minisci-alkylation driven by H_2_ evolution (Fig. 1a IV)^29^. Considering the pursuit of greener synthesis with atom- and step-efficiency, developing oxidant-free CDC-type transformation without the use of specialized dehydrogenative Minisci-alkylation using Rh_2_O_3_/GaN as the key turnover catalyst and a simple ketone, benzophenone, as the C(sp^3^)-H activating catalyst under mild and sustainable photochemical conditions (Fig. 1b).

Of widely utilized photosensitizers, ketones are established hydrogen atom abstractors^30,31^. It is affordable, readily available, and has shown many practical photochemical transformations. Yet, its application is largely confined to excess loading and diminished reactivity on a catalytic scale without the presence of a sacrificial reagent^32,33^. Therefore, the regeneration of ketones without stoichiometric loading of strong oxidants would be beneficial. After thoughtful consideration, we envisioned a photoexcited semiconductor that can generate electron-hole pairs as surface redox sites to act as a sustainable alternative to chemical oxidants^34–36^. Semiconductors have had a surge of contributions as heterogeneous photocatalysts in organic transformations with features like quantum dots^37–39^. GaN is of particular interest due to its wide band gap (3.4 eV) and the position of the gap structure that’s accessible in the UV-Vis spectrum^40^. When activated, the valence band is positioned to regenerate the spent HAT agent; meanwhile, the conduction band could be responsible for the support of H_2_ evolution^41–43^. Our earlier works have shown that GaN powder can efficiently be tuned to promote surface chemistry, while being recyclable and non-toxic^43,44^. Endowed with the presented literature findings, we propose the catalyst combination of GaN with a ketone in a CDC reaction. To our delight, experimental results demonstrate a hydrogen evolution-driven Minisci-alkylation via a dual photocatalytic strategy for an effective HAT pathway under chemical oxidant-free conditions. The heterogeneous catalyst, Rh_2_O_3_/GaN, could be recycled multiple times, and a simple aryl ketone served as an efficient HAT agent in catalytic loading. The resulting protocol allowed for the formation of C-C bonds in an atom-economical and sustainable fashion, avoiding the use of a stoichiometric amount of strong oxidants or expensive photocatalysts.

## Results and discussion

### Reaction optimization

In the preliminary studies, we subjected the dual catalytic system of commercial GaN powder (c-GaN, 30 mol%) and benzophenone (15 mol%) to 2-phenylquinoline **1** (0.1 mmol) and cyclohexane **2** (0.8 mL) under a broad wavelength spectrum xenon lamp with 2 equivalents of trifluoroacetic acid (TFA) under an inert atmosphere in acetonitrile (CH_3_CN) to witness the yield of the desired product **3** was unsatisfyingly low (Table 1, Entry 2). More favorable reaction efficiency was obtained with the addition of benzene (PhH) (albeit toxic) as a cosolvent to increase solubility, as cyclohexane and CH_3_CN are immiscible (Table 1, Entry 3). Gladly, the reaction yield was significantly improved by changing the light source to a controlled wavelength of 370 or 390 nm (Table 1, Entries 4 and 5). Our recent report demonstrated that co-catalysts on the c-GaN surface were a simple yet effective way to increase reactivity^44^.

**Table 1:** Reaction optimizations^a^.


Entry	Variation from std. reaction conditions	3 yield (%)^b^
1	None	89^c^
2	c-GaN (30 mol%), Xe lamp, no PhH	12
3	c-GaN (30 mol%), Xe lamp	23
4	c-GaN (30 mol%), Kessil 370 nm	35
5	c-GaN (30 mol%)	43
6	1 wt% Rh_2_O_3_/GaN (30 mol%)	85
7	Under the air atmosphere	24
8	No benzophenone	22
9	No GaN	12
10	In the dark, 35 °C	n.d.

*c-GaN* commercial GaN powder.

^a^All reactions were conducted at a 0.1 mmol scale of **1** under an inert atmosphere unless otherwise noted.

^b^Yields obtained by ^1^H NMR with dibromomethane as internal standard.

^c^Isolated yield.

Optimization with various metal sources showed that Rh exhibited the highest reactivity in the reaction (Table 1, Entry 6). Reduced loading of GaN led to slight improvement for the optimized conditions of 1 wt% Rh_2_O_3_/GaN (15 mol%), benzophenone (15 mol%), 2 equivalents of TFA in CH_3_CN, and PhH, the product **3** was formed in 89% yield (Table 1, Entry 1). Differing control experiments showed that GaN, benzophenone, light, and an inert atmosphere are all indispensable for the reaction to proceed (Table 1, Entries 7–10). Further optimization efforts are shown in Supplementary Table 1 and Supplementary Fig. 3.

### Heterogeneous catalyst characterizations

**Fig. 2:**
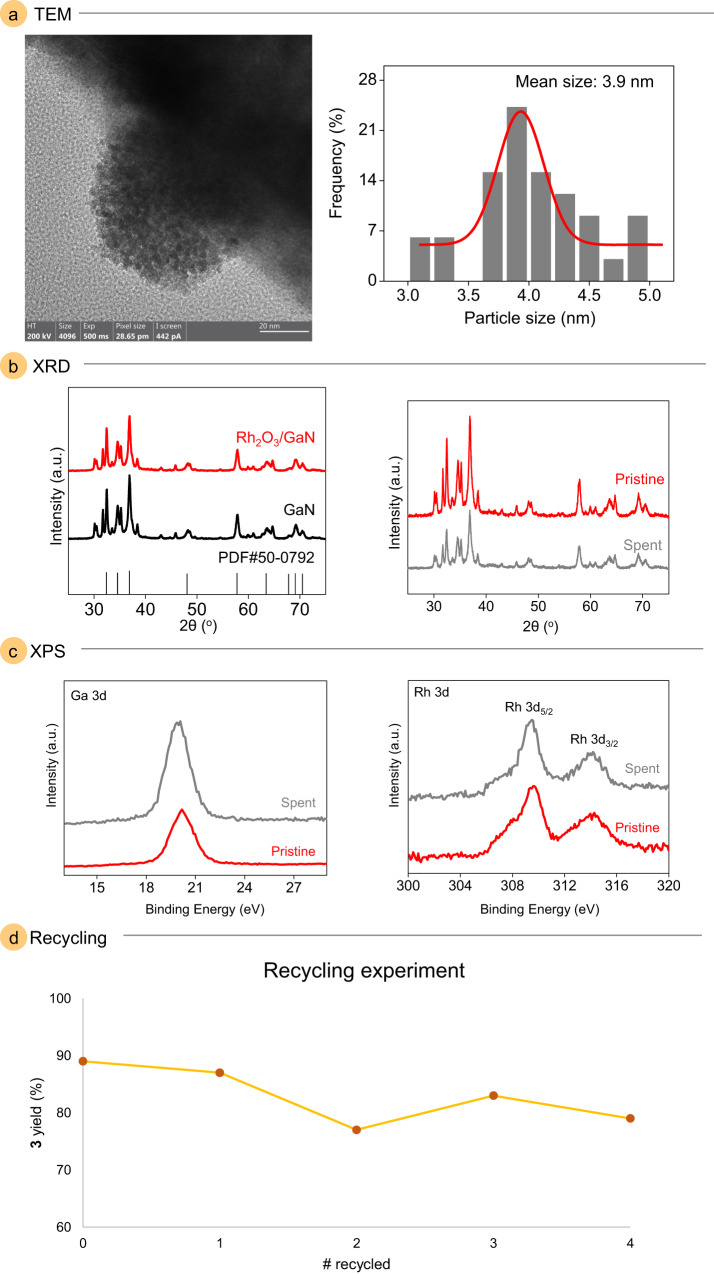
Characterization of the heterogeneous catalyst. **a** Typical transmission electron microscopy (TEM) of Rh_2_O_3_ nanoparticles on GaN and size distribution. **b** X-ray diffraction (XRD) patterns of pristine Rh_2_O_3_/GaN (red), commercial GaN (black), and spent Rh_2_O_3_/GaN (gray). **c** X-ray photoelectron spectroscopy (XPS) spectra of pristine (red) and spent (gray) Rh_2_O_3_/GaN in the Ga 3*d* and Rh 3*d* regions. **d** Recycling experiments of the heterogeneous catalyst.

To better understand the physical properties of the modified GaN catalyst, transmission electron microscopy was conducted. The formation of nanoclusters of rhodium co-catalyst on the GaN surface was observed, suggesting that the photodeposition method with methanol as a sacrificial reductant efficiently formed nanoparticles on the surface (Fig. 2a and Supplementary Fig. 13). The pristine and spent catalyst was investigated with X-ray diffraction analysis to show no obvious changes to the structure as well as the modification of GaN with Rh_2_O_3_ did not alter the surface sites of GaN (Fig. 2b and Supplementary Figs. 9, 10). X-ray photoelectron spectroscopy showed the presence of the c-plane of GaN in the Ga 3d region and the Rh 3d region revealed the presence of Rh_2_O_3_ as the Rh species with no significant difference in the pristine and spent catalysts (Fig. 2c and Supplementary Figs. 11,12)^45,46^. The X-ray diffraction and X-ray photoelectron spectroscopy of the spent catalyst suggested a robust material; therefore, with the model reaction, we evaluated the recyclability of the heterogeneous catalyst (Fig. 2d and Supplementary Data 2). After each reaction, the heterogeneous catalyst was separated from the solution by centrifugation and subjected to the next reaction. To our delight, the catalyst reactivity did not significantly decrease after five iterations (Supplementary Fig. 5). The pristine and spent catalyst was further investigated with transmission electron microscopy and scanning electron microscopy with no changes observed (Supplementary Figs. 15–18). Moreover, energy-dispersive X-ray elemental mapping images displayed the uniform distribution of the Rh_2_O_3_ nanoclusters along the entire GaN surface (Supplementary Fig. 14). It is noted that, the GaN modified with Rh_2_O_3_ was more accommodating for the protocol than c-GaN. The increased reactivity is likely due to a significant acceleration in the charge transfer, accomplishing a more efficient catalyst. The improvement in the charge transfer by Rh_2_O_3_ will suppress the electron-hole recombination and allow for greater amounts of surface reactions to occur^47^.

### Reaction scope

**Fig. 3:**
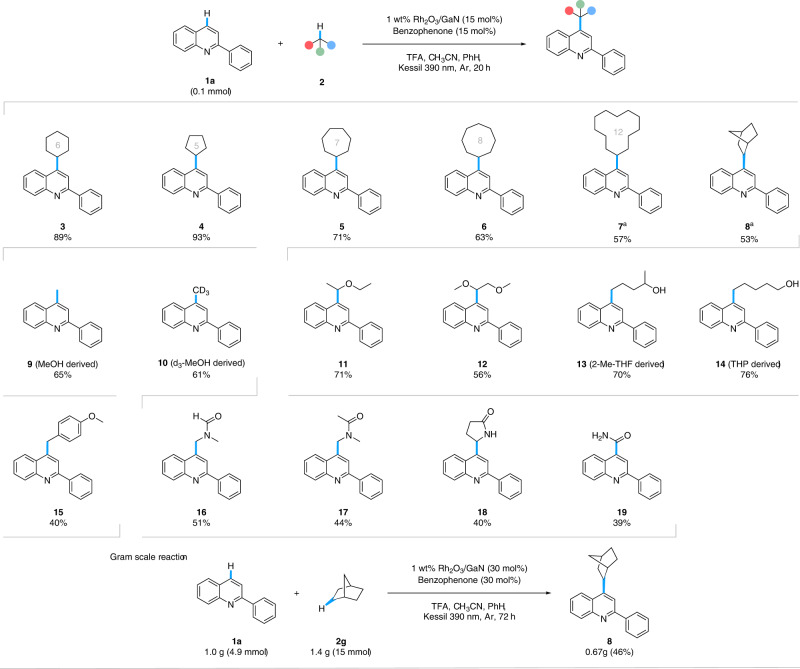
Substrate scope of alkane radical source. Reaction conditions: **1a** (0.1 mmol). **2** (0.125 M for liquids or 3.5 equiv for solids), 1 wt% Rh_2_O_3_/GaN (15 mol%), benzophenone (15 mol%), TFA (2 equiv), CH_3_CN (0.2 mL for liquid alkane or 0.8 mL for solid alkane source), and PhH (0.1 mL for liquid alkane or 0.3 mL for solid alkane source) under the Kessil lamp light source (390 nm) for 20 h under Ar atmosphere at 35 °C. Isolated yields are reported unless otherwise noted. Reaction conditions for gram-scale reaction: **1a** (4.9 mmol). **2** **g** (15 mmol), 1 wt% Rh_2_O_3_/GaN (30 mol%), benzophenone (30 mol%), TFA (4 equiv), CH_3_CN (10 mL), and PhH (3 mL) under the Kessil lamp light source (390 nm) for 72 h under Ar atmosphere at 35 °C. ^a^PhH (0.4 mL) and 28 h reaction time. TFA trifluoroacetic acid, 2-Me-THF 2-methyltetrahydrofuran, THP tetrahydropyran.

With the optimized conditions in hand, we evaluated a range of C(sp^3^)-H substrates with 2-phenylquinoline (**1**) as the heterocycle substrate (Fig. 3). Assorted cyclic hydrocarbons gave moderate to good yields with a trend of increased carbon count leading to decreased yields (**4**-**8**). The larger cyclic alkanes, cyclododecane, and norbornane required extended reaction time and increased solvent loading to aid solubility for sufficient yields (**7** and **8**). The influence of functional groups was examined, beginning with alcohol moieties. Both methanol and its deuterated version are compatible in the reaction to give the alkylated products (**9** and **10**). Likewise, linear ethers, diethyl, and dimethoxy ethers resulted in the desired products (**11** and **12**). However, the C-O bond was cleaved in cyclic ethers likely due to ring strain to give the respective alcohol products of ring opening, as observed in our previous work (**13** and **14**)^29^. Activation of benzylic C(sp^3^)-H was demonstrated with 4-methyl anisole, giving a moderate yield (**15**). Common amides, DMF, DMA, and 2-pyrrolidinone are also viable C(sp^3^) radical sources in this protocol (**16**-**18**). Notably, formamide was employed as a C(sp^2^) radical source for heteroarene formamidation (**19**). The application was extended to a gram-scale reaction between heteroarene **1a** and alkane **2** **g**, although higher catalyst loading was required (Supplementary Fig. 2).

**Fig. 4:**
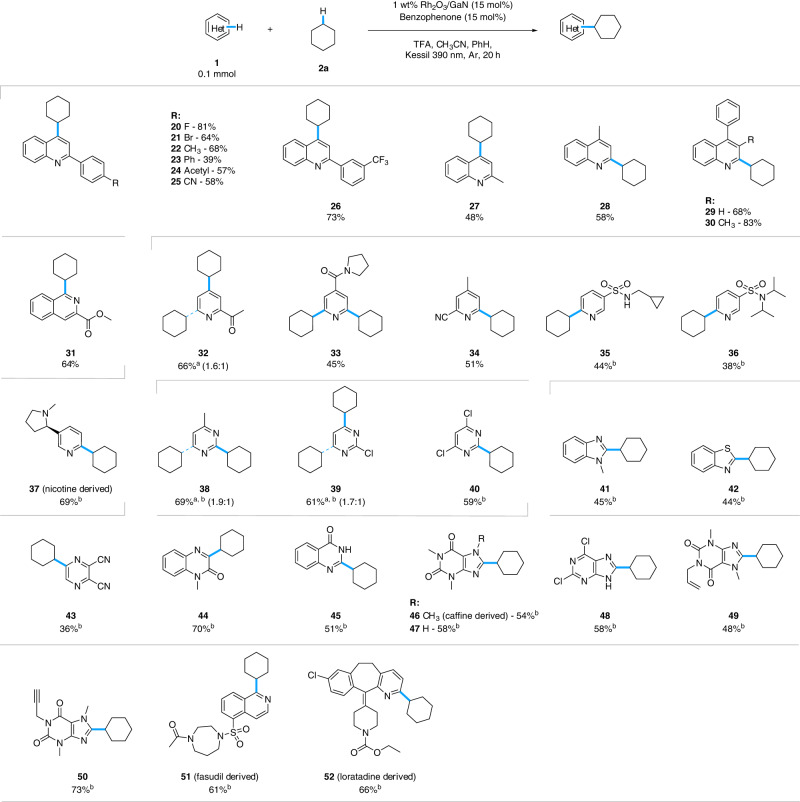
Substrate scope of heterocycle source. Reaction conditions: **1a** (0.1 mmol). **2** (0.125 M), 1 wt% Rh_2_O_3_/GaN (15 mol%), benzophenone (15 mol%), TFA (2 equiv), CH_3_CN (0.2 mL), and PhH (0.1 mL) under the Kessil lamp light source (390 nm) for 20 h under Ar atmosphere at 35 °C. Isolated yields are reported unless otherwise noted. ^a^Product ratio (mono:di). ^b^TFA (4 equiv). TFA trifluoroacetic acid.

The extent of the heteroarene partner was looked at with cyclohexane (**2a)** as the model alkylating partner (Fig. 4). Assortment of substituents was tolerated on the 2-phenylquinoline scaffold with satisfying yields in the presence of halo, methyl, phenyl, acetyl, and cyano groups (**20**–**27**). C4-substituted quinolines and isoquinoline are also feasible for alkylation (**28**–**31**). With substituted pyridines, depending on the steric bulk of the respective substituent, the mono- or di-alkylated products were observed. Specifically, with nicotine, a mono-alkylated product was obtained exclusively in moderate yield (**32**–**37**). Additionally, pyrimidine, benzimidazole, benzothiazole, pyrazine, quinoxaline, and quinazoline derivatives showcased the broad applicability of the protocol towards various heterocyclic systems with moderate to good yields (**38**–**45**). Next, purines, heterocycles well-known as privileged scaffolds for their biological activities and presence in natural products, were successfully transformed into the desired products with a higher loading of TFA (**46**–**50**)^48,49^. The applicability of our method for late-stage functionalization of pharmaceutically relevant molecules was successfully investigated with Fasudil, a Rho-Kinase inhibitor for cardiovascular disease treatment, and loratadine, an antihistamine medication (**51** and **52**)^50,51^. NMR data are presented in Supplementary Data 3, and notable unsuccessful substrates are shown in Supplementary Fig. 4.

### Mechanistic investigations

**Fig. 5:**
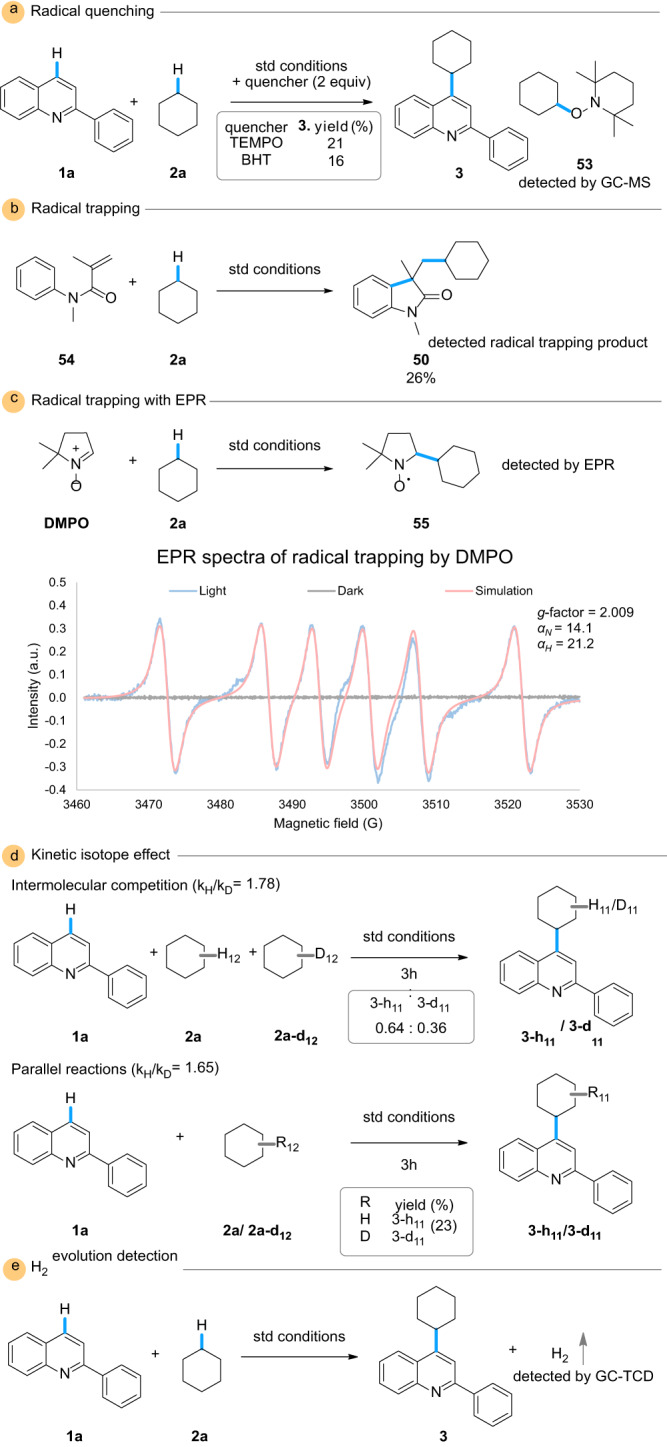
Mechanistic investigations. **a** Radical quenching with quenchers 2,2,6,6-tetramethylpiperidine 1-oxyl (TEMPO) and 3,5-di-*tert*-4-butylhydroxytoluene (BHT). **b** Radical trapping with **54**. **c** Radical trapping with 5,5-dimethyl-1-pyrroline *N*-oxide (DMPO) and electron paramagnetic resonance (EPR) analysis of trapped product **55**. **d** Kinetic isotope effect via intermolecular competition and parallel reactions. **e** Hydrogen gas evolution detection. GC-MS gas chromatography-mass spectrometry, GC-TCD gas chromatography-thermal conductivity detector.

**Fig. 6:**
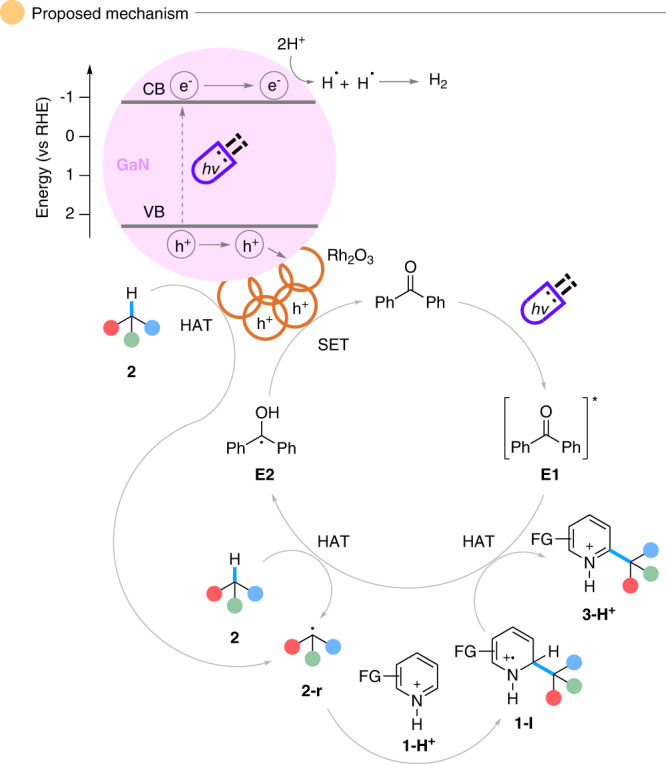
Proposed mechanism. The proposed dehydrogenative Minisci-alkylation mechanism.

A series of experiments were completed to understand the mechanism of this transformation (Fig. 5). Radical quenching experiments demonstrated significant suppression of the desired product formation: specifically, when 2,2,6,6-tetramethylpiperidine 1-oxyl was the radical quencher, the radical adduct **53** was observed by gas chromatography-mass spectrometry, suggesting a radical nature of the transformation (Fig. 5a). The involvement of an alkyl radical was studied with the radical trapper **54** as a substitute for the heterocycle, and the cycloalkylated product **50** was isolated (Fig. 5b). As additional evidence of an alkyl radical intermediate, an electron paramagnetic resonance experiment was conducted (Fig. 5c and Supplementary Data 1). Under light irradiation, the cyclohexane radical was trapped by 5,5-dimehtyl-1-pyrroline-*N*-oxide, in which the signal is in correlation with previous literature as well as our simulation data (Supplementary Fig. 6)^52^. In an attempt to find the rate-determining step, both parallel and competing kinetic isotope effects were examined (Fig. 5d). Nonetheless, in both experiments, the low k_H_/k_D_ did not suggest the involvement of the alkyl C-H cleavage in the rate-determining step, which is shown in previous literature^29,53^. The H_2_ evolution was confirmed by gas chromatography-thermal conductivity detector analysis (Fig. 5e). Lastly, fluorescence quenching of benzophenone was observed (Supplementary Figs. 7, 8). With the gathered data in hand, we constructed a plausible mechanism (Fig. 6). Upon light irradiation, the ketone achieves its excited state while simultaneously, the GaN generates electron-hole pairs on the valence and conduction bands, respectively. The excited ketone undergoes HAT with the alkane (**2**) to generate the alkyl radical (**2-r**) along with the intermediate **E2**. Afterward, **E2** reverts to benzophenone by reducing the electron hole on the valence band of the semiconductor following deprotonation, closing the catalytic ketone cycle. Additionally, it is possible that the electron hole on the valence band can be reduced by **2** to generate the radial **2-r**. The accumulated electrons on the semiconductor’s conduction band could reduce protons into H_2_ and complete the cycle of the heterogeneous catalyst. The Rh_2_O_3_ nanoclusters are shown to possibly aggregate the electron hole, effectively increasing the charge transfer and suppressing the electron-hole recombination.

## Methods

### General experimental procedure for the cross-dehydrogenative coupling of alkanes and heterocycles (Supplementary Methods)

To a 10 mL Pyrex microwave tube equipped with a Teflon-coated magnetic stirring bar were added heteroarene (0.1 mmol), 1 wt% Rh_2_O_3_/GaN (1.3 mg, 0.015 mmol), and benzophenone (2.7 mg, 0.015 mmol). For liquid alkanes, the tube was sealed, evacuated, and backfilled with argon three times using freeze-pump-thaw before the alkane (0.8 mL), CH_3_CN (0.2 mL), PhH (0.1 mL), and TFA (15 μL, 0.2 mmol) were sequentially added in the glovebox, and then sealed with an aluminum cap with a septum. For solid alkanes, 3.5 equivalents were added to the vial before being sealed, evacuated, and backfilled with argon three times using freeze-pump-thaw before the CH_3_CN (0.8 mL), PhH (0.3 mL), and TFA (15 μL, 0.2 mmol) were sequentially added in the glovebox and then sealed with an aluminum cap with a septum. The reaction vial was taken out of the glovebox and stirred under the irradiation of a 390 nm Kessil lamp at 100% light intensity for 20–28 h at 35 °C (Supplementary Fig. 1). After the reaction was completed, the solution was basified with saturated sodium bicarbonate (aq), followed by extracting the organic layer with ethyl acetate and filtering through a short pad of magnesium sulfate. The volatiles were removed under reduced pressure to obtain the crude product. The product was isolated by preparative thin-layer chromatography.

### Preparation of Rh_2_O_3_/GaN (Supplementary Methods)

Rh_2_O_3_/GaN was prepared based on a reported photodeposition method^41^. To a 10 mL quartz tube equipped with a Teflon-coated magnetic stirring bar were added commercial GaN powder (50 mg), RhCl_3_•xH_2_O (1.3 mg, 1 wt%), deionized water (3 mL), and methanol (2 mL). The tube was sealed, evacuated, and backfilled with argon three times using freeze-pump-thaw and sonicated for 30 min. The reaction was stirred under photoirradiation of a Xenon lamp (PE300 BUV) for 3 h. The suspension was collected by centrifugation and washed with deionized water three times and then with methanol twice. The final sample was obtained after drying under a vacuum overnight.

### Supplementary information

Supplementary Information

Description of Additional Supplementary Files

Supplementary Data 1

Supplementary Data 2

Supplementary Data 3

## Data Availability

The data supporting the findings of this study is included in the article and its Supplementary Information. Electron paramagnetic resonance experiment in Supplementary Data 1. Recyclability of the heterogeneous catalyst in Supplementary Data 2. NMR data in Supplementary Data 3. All data are available from the corresponding author upon reasonable request.
