# Supplementary material for: Acceptorless cross-dehydrogenative coupling for C(sp3)-H heteroarylation mediated by a heterogeneous GaN/ketone photocatalyst/photosensitizer system
Source: Commun Chem. 2023 Sep 1;6:181. doi: 10.1038/s42004-023-00947-w (PMC10474291; doi:10.1038/s42004-023-00947-w)
Supplement: Supplementary file 1 — Supplementary Information [file 42004_2023_947_MOESM1_ESM.pdf]

Supporting Information

Acceptorless cross-dehydrogenative coupling for C(sp^3)-H heteroarylation mediated by a heterogeneous GaN/ketone photocatalyst/photosensitizer system

Hyotaik Kang,‡ Lida Tan,‡ Jing-Tan Han,‡ Chia-Yu Huang, Hui Su, Aleksei Kavun, and Chao-Jun Li*

Department of Chemistry and FRQNT Centre for Green Chemistry and Catalysis, McGill University, 802 Sherbrooke St. W., Montréal, Quebec H3A 0B8, Canada

*Corresponding author: cj.li@mcgill.ca

Table of Contents

Supplementary Methods 2
General Information 2
Coupling of heteroarenes and liquid hydrocarbons 3
Coupling of heteroarenes and solid hydrocarbons 3
Gram-scale synthesis of **8** 4
Reaction optimizations 4
Unsuccessful substrates (yield <30%) 6
Preparation of GaN catalyst 6
Recycling test of the heterogeneous catalyst 6
Mechanistic studies 7
Radical quenching experiment 7
Alkyl radical trapping experiment 8
Radical trapping with EPR 8
Kinetic isotope effect (KIE) experiment 9
Detection of the H_2 evolution 10
Quantum yield measurement 11
Fluorescence quenching experiment of benzophenone 11
Characterization of the heterogeneous catalyst 13
X-ray powder diffraction (XRD) data 13
X-ray photoelectron spectroscopy (XPS) data 14
Transmission electron microscopy (TEM) data 15
Scanning electron microscopy (SEM) data 17
Characterization data for reported compounds 18
Supplementary References 33

Supplementary Methods

General Information

All reactions were carried out in 10 mL Pyrex sealed tubes under an inert atmosphere with dry solvents under anhydrous conditions unless otherwise stated. Experiments under light irradiation were performed using a 390 nm PR160L Kessil lamp with 100% light intensity equipped with a cooling fan for temperature control (https://kessil.com/products/science_PR160L.php). Column chromatography was performed with E. Merck silica gel 60 (230–400 mesh). Dry solvents were prepared over 4 Å molecular sieves (beads, 8-12 mesh) and degassed by purging with argon for 30 min. The 4 Å molecular sieves were purchased from Sigma-Aldrich chemical company and were freshly activated in the oven for 12 h at 380 ℃ before use. Reagents were purchased from the following vendors: Sigma-Aldrich, Combi-Blocks, TCI America, Oakwood, and Fisher Scientific chemical and were used without further purification unless otherwise indicated. Nuclear magnetic resonance (NMR) spectra, including ^{1}H NMR, ^{13}C NMR, ^{2}H NMR, and ^{19}F NMR, were recorded on Bruker 500 MHz spectrometer. The chemical shifts are references to solvent residual signal ($CDCl_3$: ^{1}H: δ 7.28 ppm and ^{13}C: NMR δ 77.02 ppm). NMR data are reported as follows: multiplicity (s = singlet, d = doublet, t = triplet, q = quartet, m = multiplet, dd = doublet of doublet, etc.), coupling constant (*J*, Hz), and integration. All NMR spectra were recorded at room temperature. Gas chromatography-mass spectroscopy (GC-MS) was obtained from the Agilent gas chromatography-mass spectroscopy system with helium (He) as the carrier gas. High-resolution mass spectrometry (HRMS) was conducted by using atmosphere pressure chemical ionization (APCI) or electro-spraying ionization (ESI) and was performed by McGill Chemistry Characterization Facility on a Thermo-Scientific Exactive Orbitrap. Protonated/deprotonated molecular ions $(M\pm H)^+$ or sodium adducts $(M+Na)^+$ were used for empirical formula confirmation. Gas chromatography-thermal conductivity detector (GC-TCD) was conducted on an Agilent 6890N Network Gas Chromatograph for hydrogen gas (H_2) analysis using argon (Ar) as the carrier gas. Fluorescence quenching was performed on VARIAN CARY Eclipse fluorescence spectrophotometer. Electron paramagnetic resonance (EPR) was performed on a Bruker Elexsys E580 X-band EPR Spectrometer. The bright field transmission electron microscopy (TEM) observations were carried out on FEI Tecnai G2 F20 S/TEM at accelerating voltage of 200kV. The scanning electron microscopy (SEM) was carried out on a FEI Quanta 450 Environment scanning electron microscopy (FE-ESEM) with EDAX Octane Super 60 mm^2 SDD and TEAM EDS Analysis system. The X-ray photoelectron spectroscopy (XPS) was conducted on an ESCALAB 250 X-ray photoelectron spectrometer with a monochromated X-ray source (Al Kα hv = 1486.6 eV), and the energy calibration of the spectrometer was performed using C 1s peak at 284.4 eV. The powder X-ray diffraction (XRD) patterns were obtained on a Bruker DD8 Advanced diffractometer with Cu Kα radiation (λ=1.5418Å).

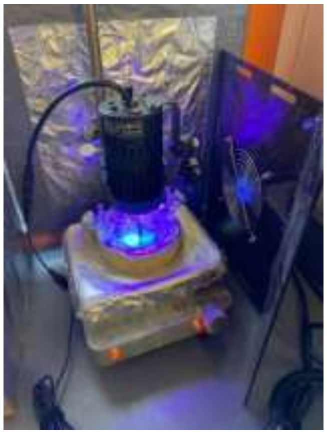

Supplementary Figure 1. Photoreaction setup for the general procedures.

Coupling of heteroarenes and liquid hydrocarbons

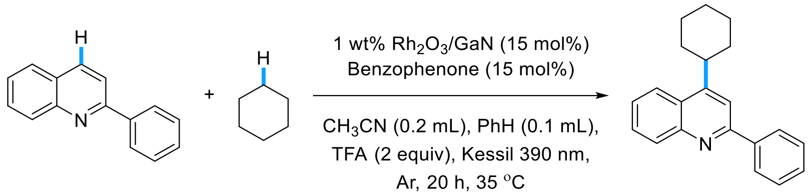

1a **2a** **3**

General procedure A. As a representative to the coupling of heteroarenes and liquid hydrocarbons unless otherwise stated. To a 10 mL Pyrex microwave tube equipped with a Teflon-coated magnetic stirring bar were added heteroarene **1a** (20.5 mg, 0.1 mmol), 1 wt% Rh_2O_3/GaN (1.3 mg, 0.015 mmol), and benzophenone (2.7 mg, 0.015 mmol). The tube was sealed, evacuated, and backfilled with argon three times using freeze-pump-thaw before the alkane **2a** (0.8 mL), CH_3CN (0.2 mL), PhH (0.1 mL), and TFA (15 μL, 0.2 mmol) were sequentially added in the glovebox, and then sealed with an aluminium cap with a septum. The reaction vial was taken out of the glovebox and stirred under the irradiation of a 390 nm Kessil lamp. After the reaction was completed, the solution was basified with saturated $NaHCO_3$ (aq), followed by extracting the organic layer with EtOAc, and filtered through a short pad of $MgSO_4$. The volatiles were removed under reduced pressure to obtain the crude product. The product was isolated by preparative thin-layer chromatography.

Coupling of heteroarenes and solid hydrocarbons

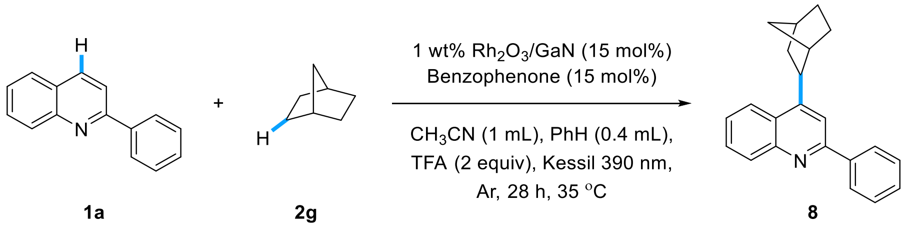

1a **2g** **8**

General procedure B. As a representative to the coupling of heteroarenes and solid hydrocarbons unless otherwise stated. To a 10 mL Pyrex microwave tube equipped with a Teflon-coated magnetic stirring bar were added heteroarene **1a** (0.1 mmol), alkane **2g** (0.35 mmol, 3.5 equiv), 1 wt% Rh_2O_3/GaN (1.3 mg, 0.015 mmol), and benzophenone (2.7 mg, 0.015 mmol). The tube was sealed, evacuated, and backfilled with argon three times using freeze-pump-thaw before the CH_3CN (0.8 mL), PhH (0.3 mL), and TFA (15 μL, 0.2 mmol) were sequentially added in the glovebox, and then sealed with an aluminium cap with a septum. The reaction vial was taken out of the glovebox and stirred under the irradiation of a 390 nm Kessil lamp. After the reaction was completed, the solution was basified with saturated $NaHCO_3$ (aq) followed by extracting the organic layer with EtOAc and filtered through a short pad of $MgSO_4$. The volatiles were removed under reduced pressure to obtain the crude product. The product was isolated by preparative thin-layer chromatography.

Gram-scale synthesis of **8**

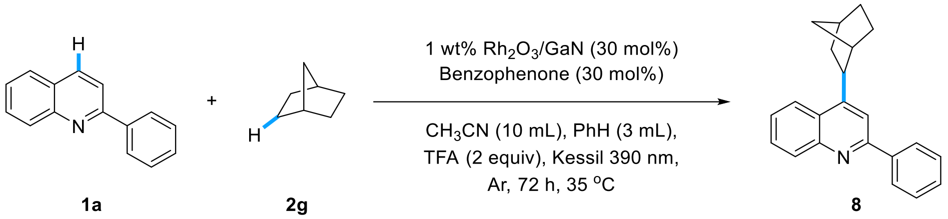

Gram-scale synthesis of 3. In a 30 mL microwave vial flask equipped with a Teflon-coated magnetic stirring bar were added heteroarene **1a** (1.0 g, 4.9 mmol), alkane **2g** (1.4 g, 15 mmol), 1 wt% Rh_2O_3/GaN (123 mg, 1.5 mmol), and benzophenone (268 mg, 1.5 mmol). The tube was sealed, evacuated, and backfilled with argon three times using freeze-pump-thaw before the CH_3CN (10 mL), PhH (3 mL), and TFA (0.7 mL, 9.8 mmol) were sequentially added in the glovebox, and then sealed with an aluminium cap with a septum. The reaction vial was taken out of the glovebox and stirred under the irradiation of a 390 nm Kessil lamp. After the reaction was completed, the solution was basified with saturated $NaHCO_3$ (aq) followed by extracting the organic layer with EtOAc and filtered through a short pad of $MgSO_4$. The volatiles were removed under reduced pressure to obtain the crude product. Product **8** was isolated by column chromatography using Hex/EtOAc eluent (0-5%) as an oil (0.67 g, 46%).

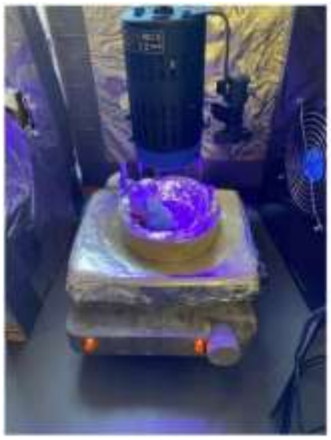

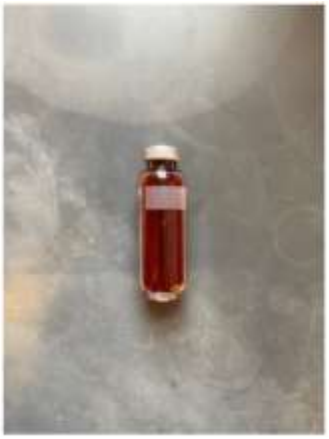

Supplementary Figure 2. Experimental setup for gram scale reaction.

Reaction optimizations

Supplementary Table 1. Reaction optimizations.

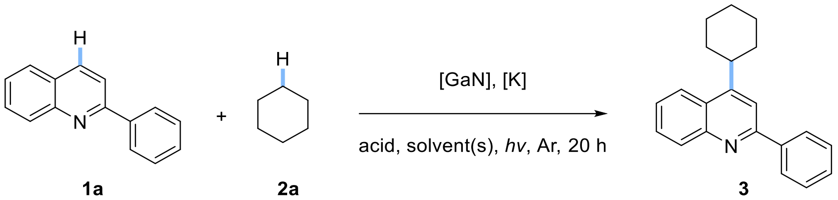

Entry	2a (mL)	[GaN] (mol%)	[K] (mol%)	acid (equiv)	solvent(s) (mL)	*hv* (nm)	3 yield (%)[a]
1	0.8	c-GaN (30)	K1 (15)	TFA (2)	CH_3CN (0.2), PhH (0.1)	Xenon (broad spectrum)	23
2	0.8	c-GaN (30)	K2 (15)	TFA (2)	CH_3CN (0.2), PhH (0.1)	Xenon (broad spectrum)	17

3	0.8	c-GaN (30)	K3 (15)	TFA (2)	CH_3CN (0.2), PhH (0.1)	Xenon (broad spectrum)	19
4	0.8	c-GaN (30)	K4 (15)	TFA (2)	CH_3CN (0.2), PhH (0.1)	Xenon (broad spectrum)	12
5	0.8	c-GaN (30)	K5 (15)	TFA (2)	CH_3CN (0.2), PhH (0.1)	Xenon (broad spectrum)	15
6	0.8	c-GaN (30)	K6 (15)	TFA (2)	CH_3CN (0.2), PhH (0.1)	Xenon (broad spectrum)	13
7	0.8	c-GaN (30)	K7 (15)	TFA (2)	CH_3CN (0.2), PhH (0.1)	Xenon (broad spectrum)	16
8	0.8	c-GaN (30)	K8 (15)	TFA (2)	CH_3CN (0.2), PhH (0.1)	Xenon (broad spectrum)	17
9	1.2	c-GaN (30)	K1 (15)	TFA (2)	CH_3CN (0.2), PhH (0.1)	Xenon (broad spectrum)	22
10	0.8	c-GaN (30)	K1 (15)	TFA (2)	CH_3CN (0.2), PhH (0.1)	Kessil (370)	35
11	0.8	c-GaN (30)	K1 (15)	TFA (2)	CH_3CN (0.2), PhH (0.1)	Kessil (390)	43
12	0.8	c-GaN (30)	K1 (15)	TFA (2)	CH_3CN (0.2), PhH (0.1)	Kessil (427)	18
13	0.8	1 wt% Fe/GaN (30)	K1 (15)	TFA (2)	CH_3CN (0.2), PhH (0.2)	Kessil (390)	50
14	0.8	1 wt% Co/GaN (30)	K1 (15)	TFA (2)	CH_3CN (0.2), PhH (0.1)	Kessil (390)	66
15	0.8	1 wt% Ru/GaN (30)	K1 (15)	TFA (2)	CH_3CN (0.2), PhH (0.1)	Kessil (390)	71
16	0.8	1 wt% Rh_2O_3/GaN (30)	K1 (15)	TFA (2)	CH_3CN (0.2), PhH (0.1)	Kessil (390)	80
17	0.8	1 wt% Rh_2O_3/GaN (15)	K1 (15)	TFA (2)	CH_3CN (0.2), PhH (0.1)	Kessil (390)	89[b]
18	0.8	1 wt% Rh_2O_3/GaN (15)	K1 (15)	TFA (1.2)	CH_3CN (0.2), PhH (0.1)	Kessil (390)	72
19	0.8	1 wt% Rh_2O_3/GaN (15)	K1 (15)	$HClO_4$ (2)	CH_3CN (0.2), PhH (0.1)	Kessil (390)	61
20	0.8	1 wt% Rh_2O_3/GaN (15)	K1 (15)	TfOH (2)	CH_3CN (0.2), PhH (0.1)	Kessil (390)	50
21	0.8	1 wt% Rh_2O_3/GaN (15)	K1 (15)	TFA (2)	DCM (0.2), PhH (0.1)	Kessil (390)	53
22	0.8	1 wt% Rh_2O_3/GaN (15)	K1 (15)	TFA (2)	CH_3Cl (0.3)	Kessil (390)	60

[a]Yields were determined by ^{1}H NMR with CH_2Br_2 as the internal standard. [b]Isolated yields. TFA: trifluoroacetic acid, $HClO_4$: perchloric acid, TfOH: triflic acid.

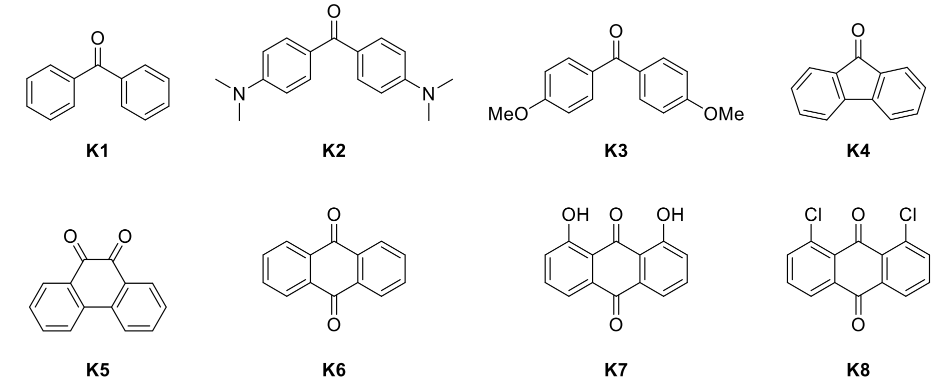

Supplementary Figure 3. Photosensitizer [K] in supplementary table 1.

Unsuccessful substrates (yield <30%)

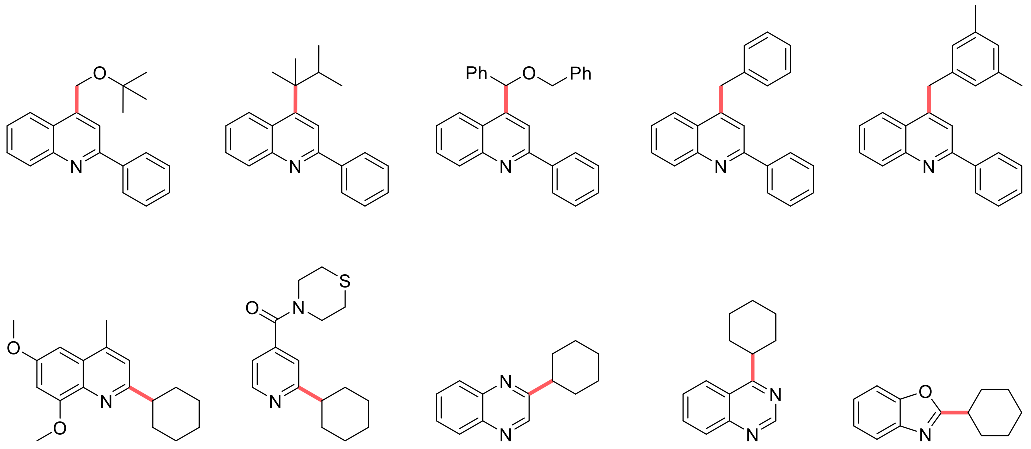

Supplementary Figure 4. Unsuccessful substrates in the reported protocol.

Preparation of GaN catalyst

Synthesis of Rh_2O_3/GaN was prepared based on the reported photodeposition method.[1] To a 10 mL quartz tube equipped with a Teflon-coated magnetic stirring bar were added commercial GaN powder (50 mg), $RhCl_3 \cdot xH_2O$ (1.3 mg, 1 wt% of Rh), deionized water (3 mL), and methanol (2 mL). The tube was sealed, evacuated, and backfilled with argon three times using freeze-pump-thaw and sonicated for 30 minutes. The reaction was stirred under photoirradiation of a Xenon lamp (PE300 BUV) for 3 h. The suspension was collected by centrifugation and washed with deionized water three times and then with methanol twice. The final sample was obtained after drying under a vacuum overnight.

Recycling test of the heterogeneous catalyst

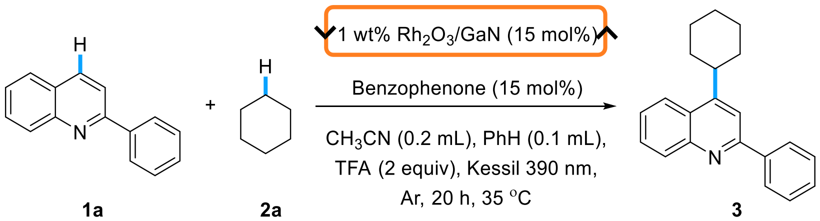

1a **2a** **3**

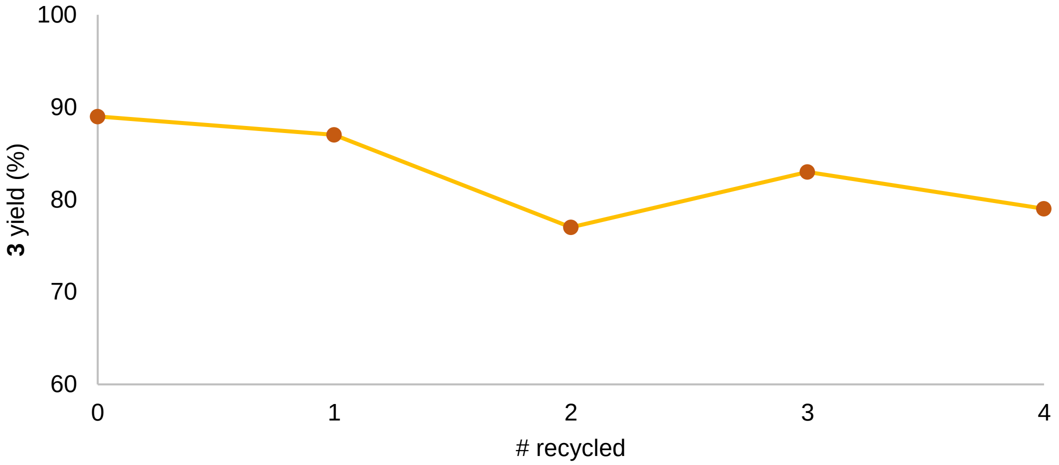

Supplementary Figure 5. The yield of product **3** in the recycling of 1 wt % Rh_2O_3/GaN catalyst.

To a 10 mL Pyrex microwave tube equipped with a Teflon-coated magnetic stirring bar were added heteroarene **1a** (20.5 mg, 0.1 mmol), 1 wt% Rh_2O_3/GaN (1.3 mg, 0.015 mmol), and benzophenone (2.7 mg, 0.015 mmol). The tube was sealed, evacuated, and backfilled with argon three times using freeze-pump-thaw before the alkane **2a** (0.8 mL), CH_3CN (0.2 mL), PhH (0.1 mL), and TFA (15 µL, 0.2 mmol) were sequentially added in the glovebox, and then sealed with an aluminium cap with a septum. The reaction vial was taken out of the glovebox and stirred under the irradiation of a 390 nm Kessil lamp for 20 h. After the reaction was completed, the solution was transferred to centrifuge tubes for centrifugation. The heterogeneous catalyst at the bottom was collected and washed with EtOAc three times. The catalyst was dried under a vacuum overnight before the next use. The washed EtOAc solution was combined with the original reaction solution and basified with saturated $NaHCO_3$ (aq), followed by extracting the organic layer with EtOAc, and filtered through a short pad of $MgSO_4$. The volatiles were removed under reduced pressure to obtain the crude product. The product was isolated by preparative thin-layer chromatography using Hex/EtOAc (10:1) as the eluent. The catalyst was recycled 4 four times, for a total of five reactions.

Mechanistic studies

Radical quenching experiment

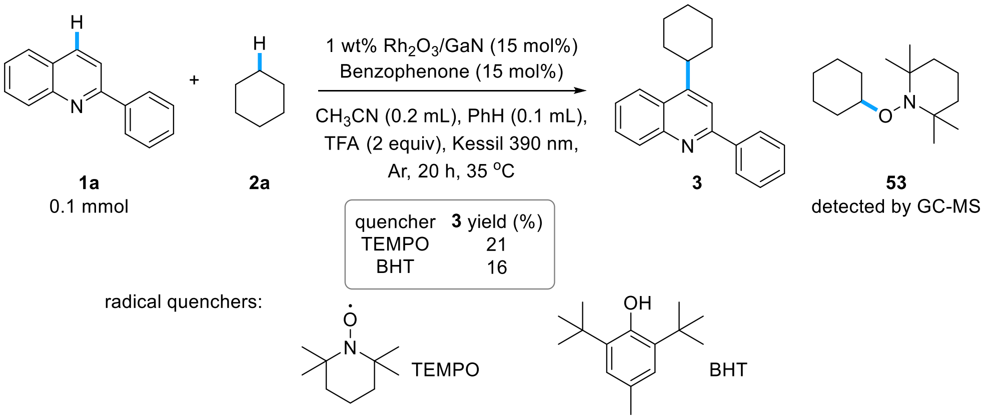

To a 10 mL Pyrex microwave tube equipped with a Teflon-coated magnetic stirring bar were added heteroarene **1a** (20.5 mg, 0.1 mmol), 1 wt% Rh_2O_3/GaN (1.3 mg, 0.015 mmol), benzophenone (2.7 mg, 0.015 mmol), and radical quencher (TEMPO or BHT, 0.2 mmol). The tube was sealed, evacuated, and backfilled with argon three times using freeze-pump-thaw before the alkane **2a** (0.8 mL), CH_3CN (0.2 mL), PhH (0.1 mL), and TFA (15 μL, 0.2 mmol) were sequentially added in the glovebox, and then sealed with an aluminium cap with a septum. The reaction vial was taken out of the glovebox and stirred under the irradiation of a 390 nm Kessil lamp. After the reaction was completed, the solution was basified with saturated $NaHCO_3$ (aq), and the organic layer was extracted with EtOAc, and filtered through a short pad of $MgSO_4$. The volatiles were removed under reduced pressure to obtain the crude product. The yield of **3** was determined by ^{1}H NMR using CH_2Br_2 as the internal standard. In the presence of either radical quencher, the formation of **3** was significantly suppressed (21% with TEMPO and 16% with BHT) which suggests the reaction proceeds through a radical-involved pathway. With TEMPO as the radical quencher, the side product **53** was detected by GC-MS, possible evidence for cyclohexyl radical formation.

Alkyl radical trapping experiment

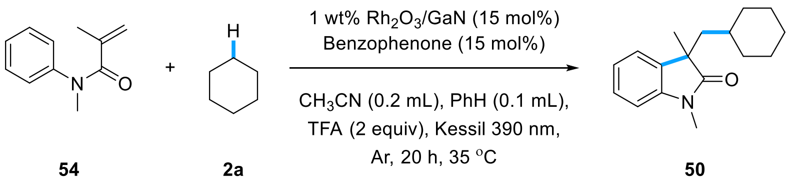

54 **2a** **50**

26%

detected radical trapping product

To a 10 mL Pyrex microwave tube equipped with a Teflon-coated magnetic stirring bar were added alkyl radical acceptor **54** (17.5 mg, 0.1 mmol), 1 wt% Rh_2O_3/GaN (1.3 mg, 0.015 mmol), and benzophenone (2.7 mg, 0.015 mmol). The tube was sealed, evacuated, and backfilled with argon three times using freeze-pump-thaw before the alkane **2a** (0.8 mL), CH_3CN (0.2 mL), PhH (0.1 mL), and TFA (15 μL, 0.2 mmol) were sequentially added in the glovebox, and then sealed with an aluminium cap with a septum. The reaction vial was taken out of the glovebox and stirred under the irradiation of a 390 nm Kessil lamp. After the reaction was completed, Et_2O was added and filtered through a short pad of silica gel with additional Et_2O. The volatiles were removed under reduced pressure to obtain the crude product. The isolated product **50** was obtained in 26% yield by preparative thin-layer chromatography using Hex/EtOAc (5:1) as the eluent. The formation of product **50** in this reaction is possible evidence for cyclohexyl radical formation.

Radical trapping with EPR

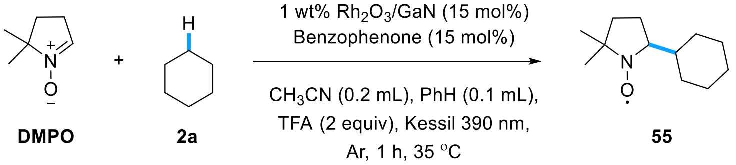

DMPO **2a** **55**

detected by EPR

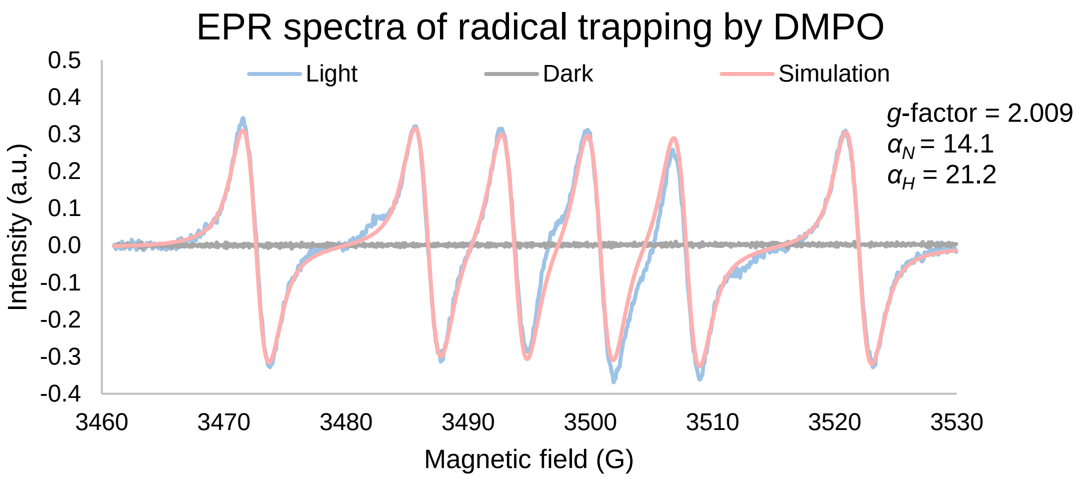

Supplementary Figure 6. The combined electron paramagnetic resonance (EPR) spectra of reactions with and without light, and simulation.

Two 10 mL Pyrex microwave tubes equipped with a Teflon-coated magnetic stirring bar were added 5,5-dimethyl-1-pyrroline-*N*-oxide (DMPO, 11.3 mg, 0.1 mmol, 1 equiv), 1 wt% Rh_2O_3/GaN (1.3 mg, 0.015 mmol), and benzophenone (2.7 mg, 0.015 mmol). The tube was sealed, evacuated, and backfilled with argon three times using freeze-pump-thaw before the alkane **2a** (0.8 mL), CH_3CN (0.2 mL), PhH (0.1 mL), and TFA (15 μL, 0.2 mmol) were sequentially added in the glovebox, and then sealed with an aluminium cap with a septum. The reaction vial was taken out of the glovebox. One reaction vial was stirred at 35 °C under the irradiation of a 390 nm Kessil lamp and another at 35 °C in the dark. After 1 h, the reactions were taken for electron paramagnetic resonance (EPR) analysis.

As shown in supplementary figure 6, signals were observed only with the reaction conducted under light irradiation. The observed signal was consistent with the reported literature and simulated results for the cyclohexyl radical DMPO adduct **55**. The observation of product **55** is possible evidence for cyclohexyl radical formation.

Kinetic isotope effect (KIE) experiment

Intermolecular competition (k_H/k_D = 1.78)

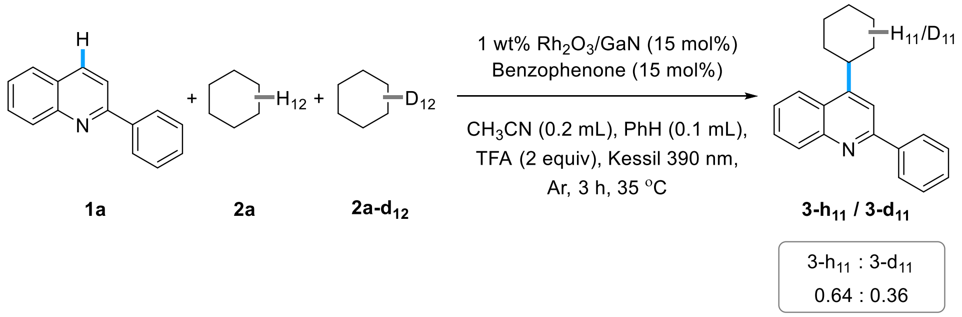

To a 10 mL Pyrex microwave tube equipped with a Teflon-coated magnetic stirring bar were added heteroarene **1a** (20.5 mg, 0.1 mmol), 1 wt% Rh_2O_3/GaN (1.3 mg, 0.015 mmol), and benzophenone (2.7 mg, 0.015 mmol). The tube was sealed, evacuated, and backfilled with argon three times using freeze-pump-thaw before the alkane **2a** (0.3 mL), alkane **2a-d12** (0.3 mL), CH_3CN (0.2 mL), PhH (0.1 mL), and TFA (15 μL, 0.2 mmol) were sequentially added in the glovebox, and then sealed with an aluminium cap with a septum. The reaction vial was taken out of the glovebox and stirred under the irradiation of a 390 nm Kessil

lamp for 3 h. After the reaction was completed, the solution was basified with saturated $NaHCO_3$ (aq), followed by extracting the organic layer with EtOAc, and filtered through a short pad of $MgSO_4$. The volatiles were removed under reduced pressure to obtain the crude product. The NMR yield of the mixture was determined to be 22% using CH_2Br_2 as the internal standard. The product mixture gave a ratio of 0.64:0.36 for 3-h_{11}:3-d_{11}, resulting in k_H/k_D = 1.78.

Parallel reactions (k_H/k_D = 1.65)

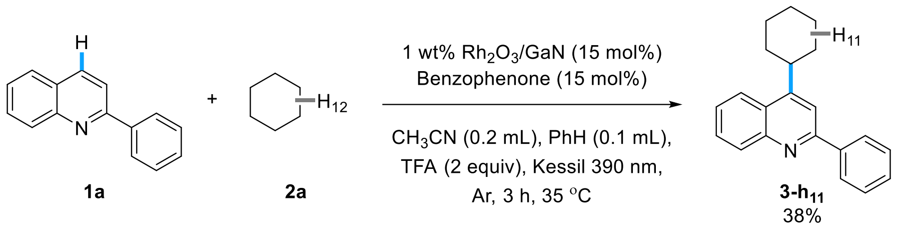

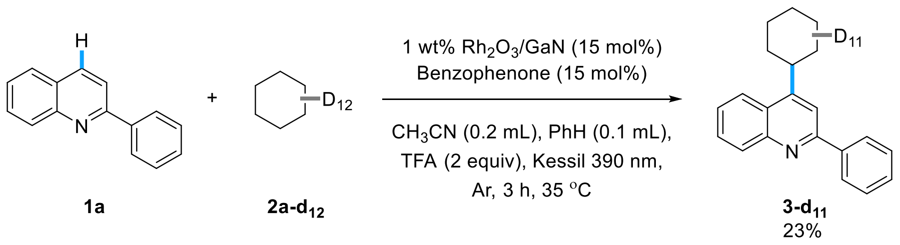

Two 10 mL Pyrex microwave tubes equipped with a Teflon-coated magnetic stirring bar were added heteroarene **1a** (20.5 mg, 0.1 mmol), 1 wt% Rh_2O_3/GaN (1.3 mg, 0.015 mmol), and benzophenone (2.7 mg, 0.015 mmol). The tube was sealed, evacuated, and backfilled with argon three times using freeze-pump-thaw before CH_3CN (0.2 mL), PhH (0.1 mL), TFA (15 μL, 0.2 mmol), were added and the alkane **2a** (0.4 mL) or **2a-d$_{12}$** (0.4 mL) was sequentially added in the glovebox and then sealed with an aluminium cap with a septum. The reaction vials were taken out of the glovebox and stirred under the irradiation of a 390 nm Kessil lamp for 3 h. After the reaction was completed, the solution was basified with saturated $NaHCO_3$ (aq), followed by extracting the organic layer with EtOAc, and filtered through a short pad of $MgSO_4$. The volatiles were removed under reduced pressure to obtain the crude product. The NMR yield of **3-h$_{11}$** (38%) and **3-d$_{11}$** (23%) using CH_2Br_2 as the internal standard gave k_H/k_D = 1.65. The isolated product **3-d$_{11}$** was obtained by preparative thin-layer chromatography using Hex/EtOAc (10:1) as the eluent. There was no H/D scrambling observed in the deuterated product **3-d$_{11}$.**

Both kinetic isotope experiments did not show any obvious isotope effect, suggesting that the alkyl C-H cleavage might not be the rate-determining step.[2]

Detection of the H_2 evolution

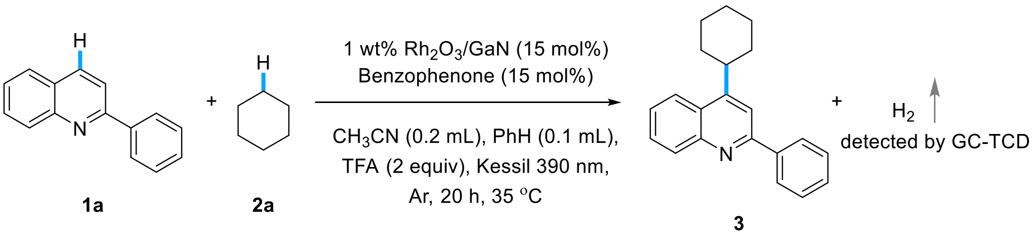

To a 10 mL Pyrex microwave tube equipped with a Teflon-coated magnetic stirring bar were added heteroarene **1a** (20.5 mg, 0.1 mmol), 1 wt% Rh_2O_3/GaN (1.3 mg, 0.015 mmol), and benzophenone (2.7 mg, 0.015 mmol). The tube was sealed, evacuated, and backfilled with argon three times using freeze-pump-

thaw before the alkane **2a** (0.8 mL), CH_3CN (0.2 mL), PhH (0.1 mL), and TFA (15 μL, 0.2 mmol) were sequentially added in the glovebox, and then sealed with an aluminium cap with a septum. The reaction vial was taken out of the glovebox and stirred under the irradiation of a 390 nm Kessil lamp for 20 h. After the reaction was completed, the upper atmosphere of the tube was extracted by a gas-tight syringe and analysed by GC-TCD. The detection of the H_2 signal indicated the formation of hydrogen in the reaction.

Quantum yield measurement

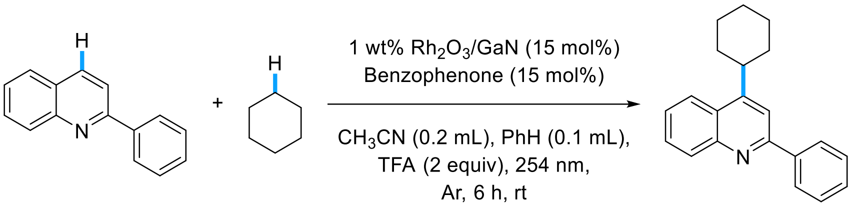

1a **2a** **3** 9%

For an accurate measure of light power, here we used UV-box with single wavelength 254 nm UVC lamps for the reaction (Luzchem Research INC, with LZC-UVC lamps). To a 10 mL quartz tube equipped with a Teflon-coated magnetic stirring bar were added heteroarene **1a** (20.5 mg, 0.1 mmol) 1 wt% Rh_2O_3/GaN (1.3 mg, 0.015 mmol), and benzophenone (2.7 mg, 0.015 mmol). The tube was sealed, evacuated, and backfilled with argon three times using freeze-pump-thaw before the alkane **2a** (0.8 mL), CH_3CN (0.2 mL), PhH (0.1 mL), and TFA (15 μL, 0.2 mmol) were sequentially added in the glovebox, and then sealed with a screw cap. The reaction tube was taken out of the glovebox and stirred in the UV box at room temperature under irradiation of 6X8 W 254 nm UVC lamps. After 6 h, the reaction was basified with saturated $NaHCO_3$ (aq), followed by extracting the organic layer with EtOAc, and filtered through a short pad of $MgSO_4$. The volatiles were removed under reduced pressure to obtain the crude sample. The NMR yield of **3** was 9% using CH_2Br_2 as the internal standard.

The light power of the 6X8 W 254 nm UVC lamps was determined by an optical power meter to be $4.36 \cdot 10^{-3}$ W.

$$\text{photon flux} = \frac{\mathrm{p}}{\mathrm{N_a} \cdot {}^{\mathrm{hc}}/_{\lambda}} = \frac{4.36 \cdot 10^{-3}}{6.02 \cdot 10^{23} \,.\, 6.63 \cdot 10^{-34} \cdot 3 \cdot 10^{8}/_{254 \cdot 10^{-9}}} = 9.25 \cdot\ 10^{-9}\ einsteins\ \cdot\ s^{-1}$$

With the assumption that all the incident light was absorbed under the reaction conditions (f > 0.9999), the production of $9.1\ 10^{-6}$ mol of 3 in $2.16\ 10^{4}$ s (6 h) led to the quantum yield (Φ) of 4.6%.

$$\Phi = \frac{\text{mol product}}{\text{photon flux} \cdot\ \mathrm{t} \cdot \mathrm{f}} = \frac{9.1 \cdot 10^{-6}}{9.25 \cdot 10^{-9} \cdot 2.16 \cdot 10^{4} \cdot 1} = 4.6\%$$

Fluorescence quenching experiment of benzophenone

A stock solution of benzophenone (10 μM in CH_3CN) was prepared in a volumetric flask. A quartz cuvette (1cm X 1cm X 3cm) was filled with the stock solution and its fluorescence emission was measured with an excitation wavelength of 254 nm. A maximum wavelength of 507 nm was measured from the stock solution. Quenching experiments were performed under matching conditions with the addition of 100 μL, 200 μL, 300 μL, 400 μL, 500 μL, 600 μL, 700 μL, and 800 μL of THF. The resulting fluorescence emissions spectra are shown.

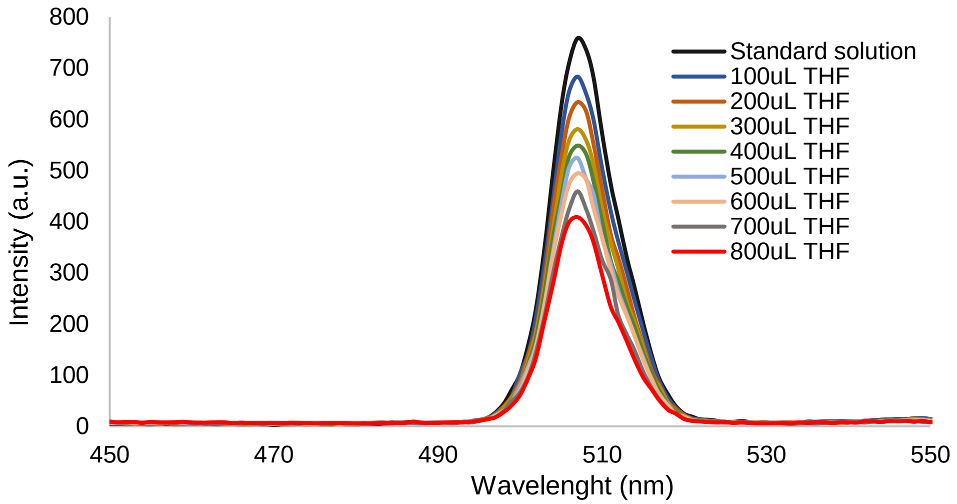

Supplementary Figure 7. Fluorescence emission of standard solution of benzophenone quenched by varied amounts of tetrahydrofuran (THF).

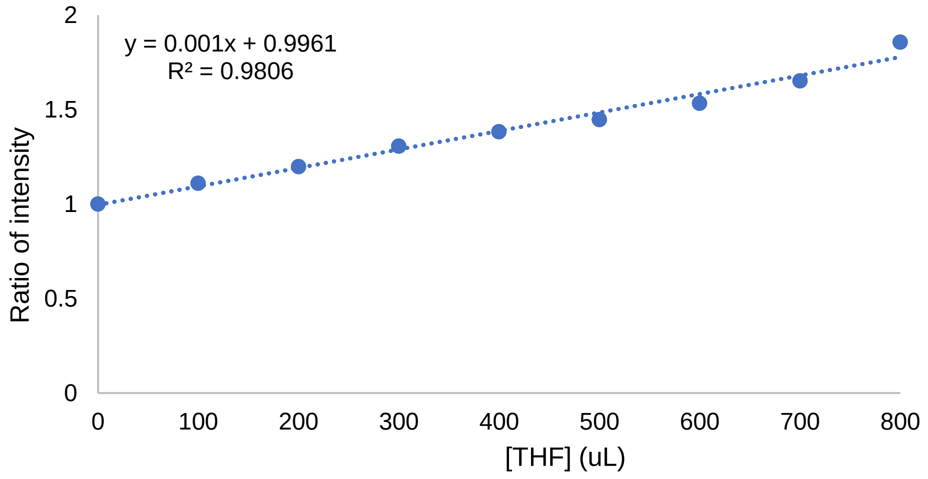

Supplementary Figure 8. Stern-Volmer plot of benzophenone and tetrahydrofuran (THF).

Significant fluorescence quenching is observed in supplementary figures 7 and 8 of benzophenone by THF, suggesting a strong interaction between the excited benzophenone and THF. It is noted that cyclohexane was not used as the quenching partner as it is immiscible in acetonitrile.

Characterization of the heterogeneous catalyst

X-ray powder diffraction (XRD) data

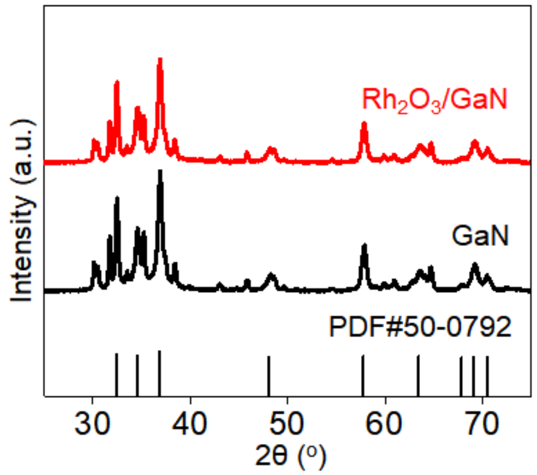

Supplementary Figure 9. Typical X-ray powder diffraction (XRD) patterns of pristine Rh_2O_3/GaN (red) and commercial GaN (black) samples. There is no obvious change to the XRD patterns after deposition of Rh_2O_3 on the GaN. This reveals that decent active regions of GaN remain after the deposition.

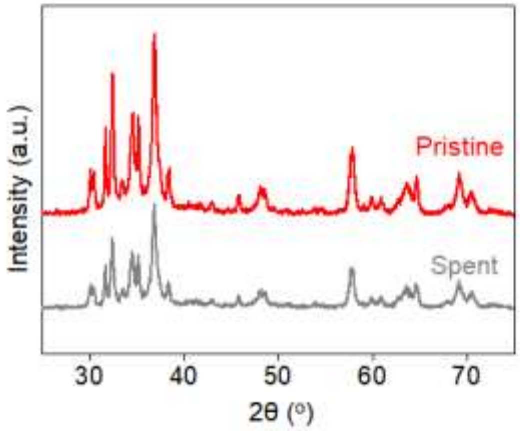

Supplementary Figure 10. X-ray powder diffraction (XRD) patterns of pristine (red) and spent (grey) Rh_2O_3/GaN samples. There is no obvious change in the pristine and spent version. This suggests good stability of the heterogeneous catalyst structure over extended use.

X-ray photoelectron spectroscopy (XPS) data

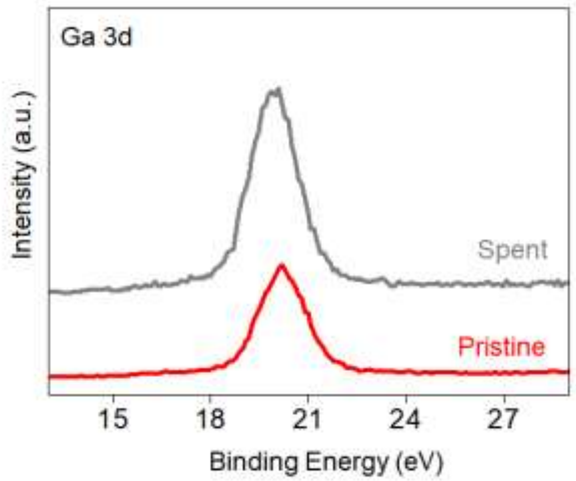

Supplementary Figure 11. X-ray photoelectron spectroscopy (XPS) spectra of the Ga 3d region of pristine (red) and spent (grey) heterogeneous catalyst.

The XPS spectra of the Ga 3d region matches the literature values of GaN.[3] There is no obvious change in the pristine and spent catalyst, suggesting a robust catalyst.

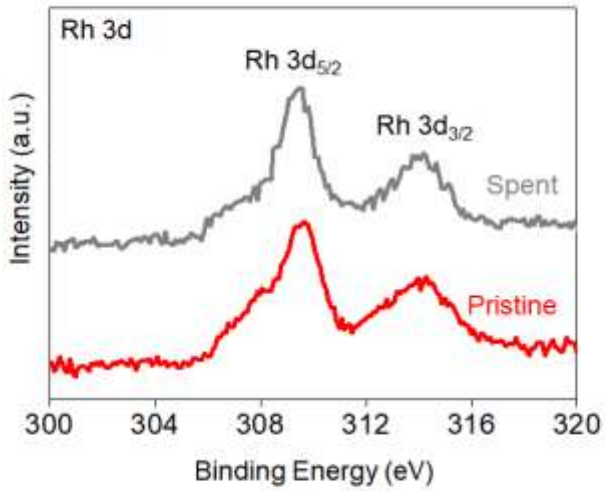

Supplementary Figure 12. X-ray photoelectron spectroscopy (XPS) spectra of the Rh 3d region of pristine (red) and spent (grey) heterogeneous catalyst.

The XPS spectra of the Rh 3d region shows the presence of Rh_2O_3 matching the reported literature.[4] There is obvious change in the pristine and spent catalyst, suggesting a robust catalyst.

Transmission electron microscopy (TEM) data

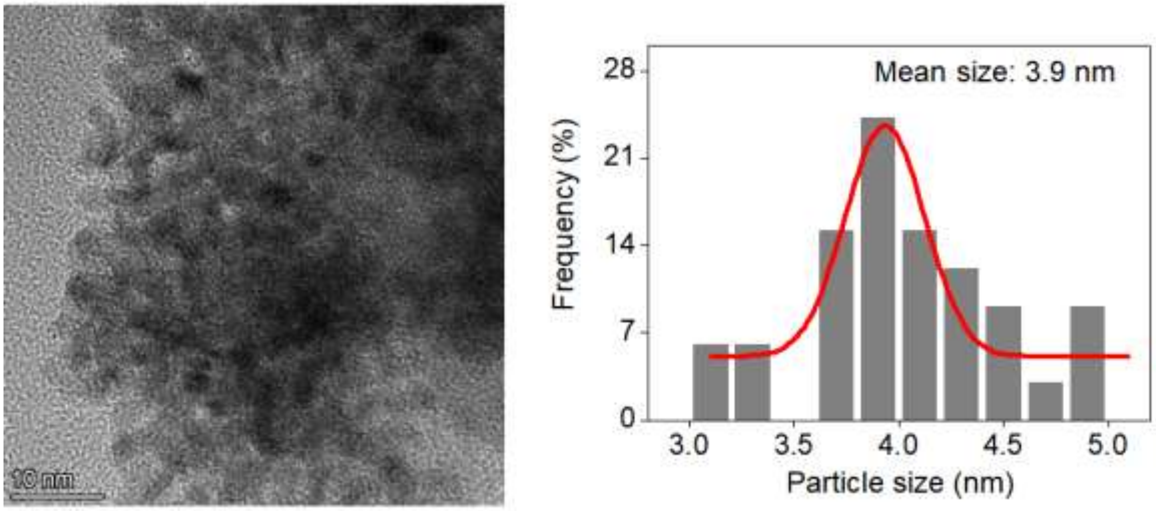

Supplementary Figure 13. Representative transmission electron microscopy (TEM) images of Rh_2O_3/GaN and its size distribution. The mean size of the Rh_2O_3 nanoparticles is 3.9 nm.

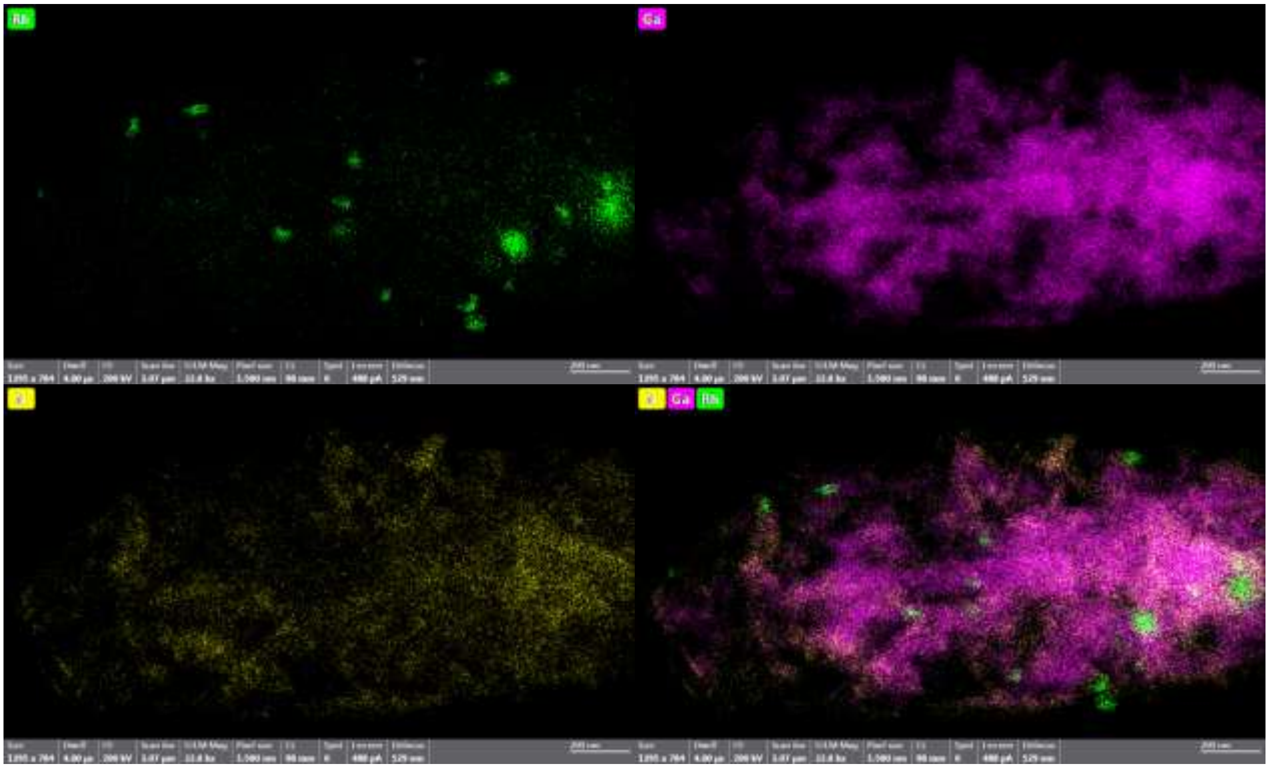

Supplementary Figure 14. Representative scanning transmission electron microscopy–energy dispersive X-ray spectroscopy (STEM-EDS) elemental mapping profile of Rh_2O_3/GaN. This observation displays the homogeneous distribution of Rh_2O_3 ensembles on the GaN surface.

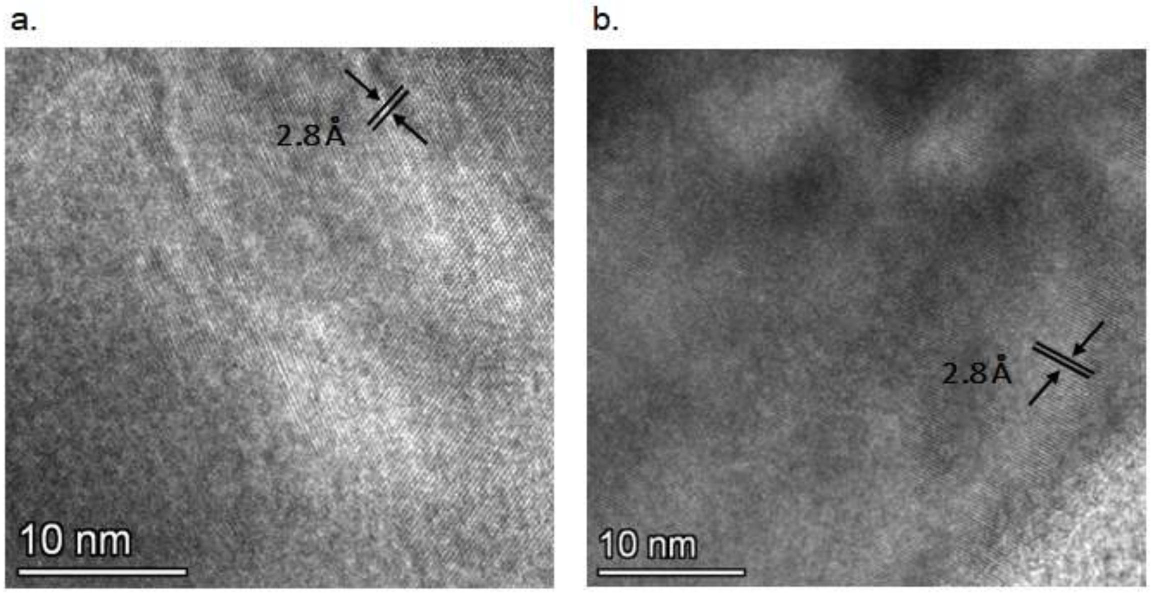

Supplementary Figure 15. High-resolution transmission electron microscopy (TEM) of the GaN surface. The c-plane of the GaN in both a. pristine and b. spent catalyst are observed.[5]

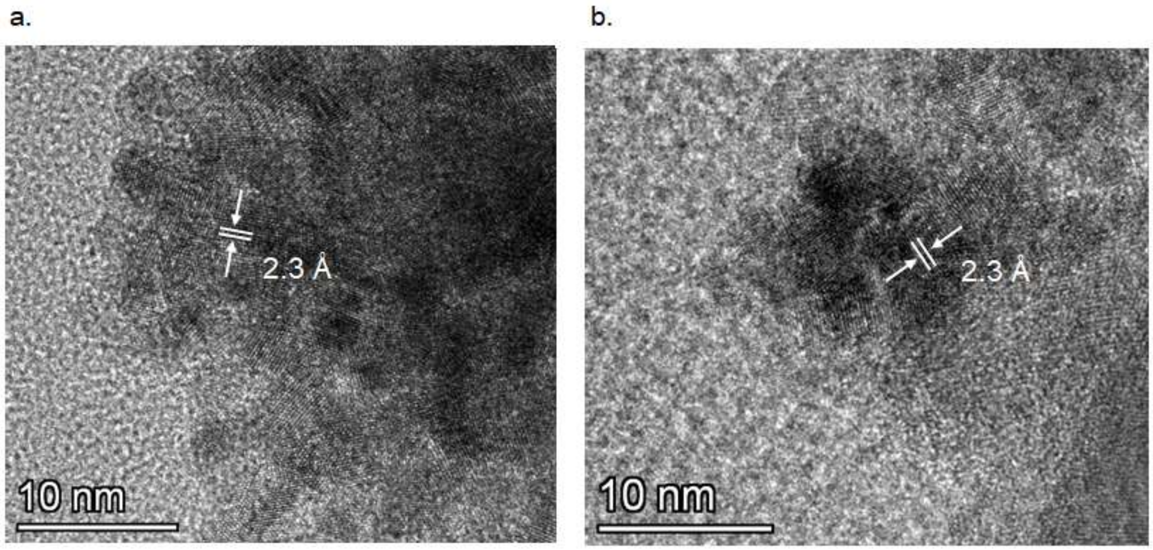

Supplementary Figure 16. High-resolution transmission electron microscopy (TEM) of the Rh_2O_3 (006) is observed in both a. pristine and b. spent catalyst.

Scanning electron microscopy (SEM) data

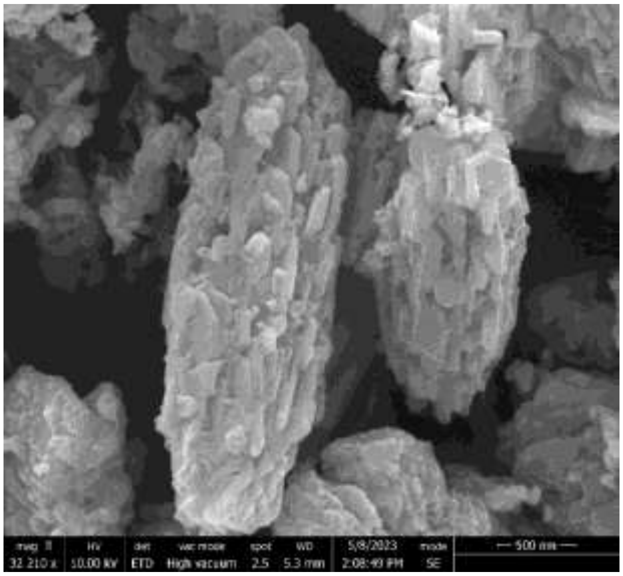

Supplementary Figure 17. Representative scanning electron microscopy (SEM) images of pristine Rh_2O_3/GaN.

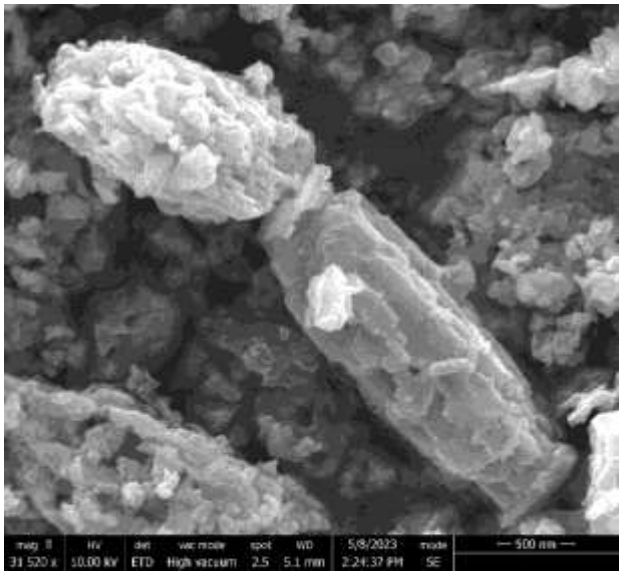

Supplementary Figure 18. Representative scanning electron microscopy (SEM) images of spent Rh_2O_3/GaN.

SEM images of pristine and spent catalyst do not show any obvious changes to the morphology of the heterogeneous catalyst.

Characterization data for reported compounds

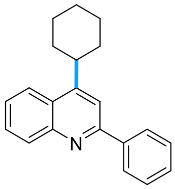

4-cyclohexyl-2-phenylquinoline (**3**). Following the general procedure A, the product was isolated by preparative TLC with Hex/EtOAc (10:1) as the eluent to give a solid (25.6 mg, 89%). **^{1}H NMR** (500 MHz, $CDCl_3$) δ 8.19 (d, *J* = 9.7 Hz, 1H), 8.15 – 8.13 (m, 2H), 8.09 (d, *J* = 9.9 Hz, 1H), 7.76 (s, 1H), 7.72 – 7.68 (m, 1H), 7.55 – 7.51 (m, 3H), 7.48 – 7.44 (m, 1H), 3.38 (tt, *J* = 11.4, 3.1 Hz, 1H), 2.11 – 2.08 (m, 2H), 1.98 – 1.95 (m, 2H), 1.90 – 1.87 (m, 1H), 1.65 – 1.55 (m, 4H), 1.40 – 1.37 (m, 1H). **^{13}C NMR** (126 MHz, $CDCl_3$) δ 157.4, 154.0, 148.6, 140.3, 130.7, 129.1, 129.0, 128.8 (X2), 127.6 (X2), 125.9 (X2), 122.9, 115.5, 39.1, 33.7 (X2), 27.0 (X2), 26.3. **GC-MS** (EI, m/z) for $C_{21}H_{21}N$ calcd: 287.2, found: 287.1. The spectra data is consistent with the reported literature.[6]

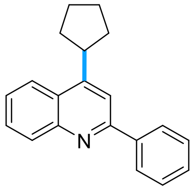

4-cyclopentyl-2-phenylquinoline (**4**). Following the general procedure A, the product was isolated by preparative TLC with Hex/EtOAc (10:1) as the eluent to give a solid (25.4 mg, 93%). **^{1}H NMR** (500 MHz, $CDCl_3$) δ 8.19 (d, *J* = 7.2 Hz, 1H), 8.16 – 8.10 (m, 3H), 7.78 (s, 1H), 7.72 – 7.69 (m, 1H), 7.56 – 7.50 (m, 3H), 7.48 – 7.44 (m, 1H), 3.87 – 3.80 (m, 1H), 2.31 – 2.22 (m, 2H), 1.96 – 1.84 (m, 6H). **^{13}C NMR** (126 MHz, $CDCl_3$) δ 157.3, 152.8, 148.5, 140.3, 130.5, 129.1, 129.0, 128.8 (X2), 127.6 (X2), 126.8, 125.8, 123.6, 115.2, 40.8, 33.4 (X2), 25.5 (X2). **GC-MS** (EI, m/z) for $C_{20}H_{19}N$ calcd: 273.2, found: 273.1. The spectra data is consistent with the reported literature.[7]

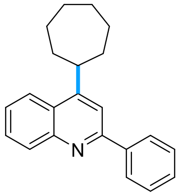

4-cycloheptyl-2-phenylquinoline (**5**). Following the general procedure A, the product was isolated by preparative TLC with Hex/EtOAc (10:1) as the eluent to give an oil (21.3 mg, 71%). **^{1}H NMR** (500 MHz, $CDCl_3$) δ 8.18 (dd, *J* = 8.6, 1.3 Hz, 1H), 8.15 – 8.13 (m, 2H), 8.08 (d, *J* = 8.5 Hz, 1H), 7.76 (s, 1H), 7.72 – 7.68 (m, 1H), 7.56 – 7.50 (m, 3H), 7.47 – 7.44 (m, 1H), 3.54 (ddt, *J* = 10.4, 6.7, 3.3 Hz, 1H), 2.14 – 2.09 (m, 2H), 1.95 – 1.80 (m, 6H), 1.74 – 1.68 (m, 4H). **^{13}C NMR** (126 MHz, $CDCl_3$) δ 157.3, 155.9, 148.6, 140.3, 130.7, 129.1, 129.0, 128.8 (X2), 127.6 (X2), 125.9, 125.6, 123.0, 115.8, 40.8, 35.9 (X2), 27.9 (X2), 27.6 (X2). **GC-MS** (EI, m/z) for $C_{22}H_{23}N$ calcd: 301.2, found: 301.2. The spectra data is consistent with the reported literature.[7]

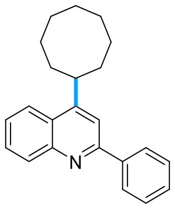

4-cyclooctyl-2-phenylquinoline (**6**). Following the general procedure A, the product was isolated by preparative TLC with Hex/EtOAc (10:1) as the eluent to give an oil (19.9 mg, 63%). **^{1}H NMR** (500 MHz, $CDCl_3$) δ 8.19 (d, *J* = 7.3 Hz, 1H), 8.16 – 8.11 (m, 2H), 8.09 (d, *J* = 7.2 Hz, 1H), 7.75 (s, 1H), 7.72 – 7.69 (m, 1H), 7.56 – 7.51 (m, 3H), 7.48 – 7.44 (m, 1H), 3.68 – 3.63 (m, 1H), 2.05 – 1.97 (m, 4H), 1.91 – 1.86 (m, 2H), 1.79 – 1.71 (m, 8H). **^{13}C NMR** (126 MHz, $CDCl_3$) δ 157.3, 156.4, 148.7, 140.3, 130.8, 129.1, 129.0, 128.8 (X2), 127.6 (X2), 125.9, 125.6, 123.1, 116.3, 33.7, 26.8 (X3), 26.6 (X2), 26.2 (X2). **GC-MS** (EI, m/z) for $C_{23}H_{25}N$ calcd: 315.2, found: 315.2. The spectra data is consistent with the reported literature.[8]

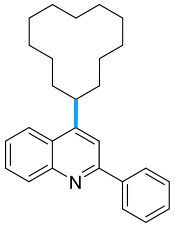

4-cyclododecyl-2-phenylquinoline (**7**). Following the general procedure B, the product was isolated by preparative TLC with Hex/EtOAc (10:1) as the eluent to give a solid (21.1 mg, 57%). **^{1}H NMR** (500 MHz, $CDCl_3$) δ 8.20 (d, *J* = 8.5 Hz, 1H), 8.16 – 8.12 (m, 3H), 7.75 (s, 1H), 7.73 – 7.69 (m, 1H), 7.57 – 7.50 (m, 3H), 7.49 – 7.45 (m, 1H), 3.73 – 3.68 (m, 1H), 2.01 – 1.95 (m, 2H), 1.79 – 1.72 (m, 2H), 1.57 – 1.38 (m, 16H), 1.32 – 1.26 (m, 2H). **^{13}C NMR** (126 MHz, $CDCl_3$) δ 157.1, 153.9, 148.7, 140.3, 130.8, 129.1, 129.0, 128.8 (X2), 127.7 (X2), 126.5, 125.9, 122.9, 116.7, 34.6, 30.2 (X2), 24.1 (X2), 23.9, 23.5 (X2), 23.5 (X2), 22.6 (X2). **GC-MS** (EI, m/z) for $C_{27}H_{33}N$ calcd: 371.3, found: 371.3. The spectra data is consistent with the reported literature.[8]

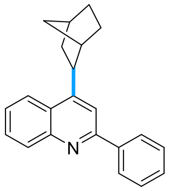

4-((2S)-bicyclo[2.2.1]heptan-2-yl)-2-phenylquinoline (**8**). Following the general procedure B, the product was isolated by preparative TLC with Hex/EtOAc (10:1) as the eluent to give an oil (15.9 mg, 53%). (**8**). **^{1}H NMR** (500 MHz, $CDCl_3$) 8.18 (d, *J* = 7.1 Hz, 1H), 8.15 – 8.13 (m, 2H), 8.06 (d, *J* = 7.1 Hz, 1H), 7.74 (s, 1H), 7.72 – 7.68 (m, 1H), 7.55 – 7.51 (m, 3H), 7.48 – 7.45 (m, 1H), 3.44 – 3.41 (m, 1H), 2.68 (d, *J* = 4.2 Hz, 1H), 2.46 – 2.44 (m, 1H), 2.07 – 2.02 (m, 1H), 1.80 – 1.66 (m, 4H), 1.60 – 1.54 (m, 1H), 1.47 – 1.42 (m, 1H), 1.39 – 1.36 (m, 1H). **^{13}C NMR** ^{13}C NMR (126 MHz, $CDCl_3$) δ 157.2, 153.3, 148.7, 140.4, 130.5, 129.1, 129.0, 128.8 (X2), 127.7 (X2), 126.5, 125.8, 123.9, 114.9, 43.0, 41.3, 39.2, 37.0, 36.7, 30.3, 29.1. **GC-MS** (EI, m/z) for $C_{22}H_{21}N$ calcd: 299.2, found: 299.1. The spectra data is consistent with the reported literature.[7]

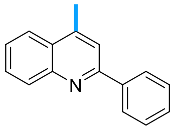

4-methyl-2-phenylquinoline (**9**). Following the general procedure A, the product was isolated by preparative TLC with Hex/EtOAc (10:1) as the eluent to give an oil (14.2 mg, 65%). **^{1}H NMR** (500 MHz, $CDCl_3$) δ 8.19 – 8.14 (m, 3H), 8.01 (d, *J* = 9.8 Hz, 1H), 7.74 – 7.71 (m, 2H), 7.57 – 7.51 (m, 3H), 7.48 – 7.44 (m, 1H), 2.78 (s, 3H). **^{13}C NMR** (126 MHz, $CDCl_3$) δ 157.1, 148.2, 144.8, 139.9, 130.3, 129.3, 129.2, 128.8 (X2), 127.6 (X2), 127.3, 126.0, 123.6, 119.8, 19.0. **GC-MS** (EI, m/z) for $C_{16}H_{13}N$ calcd: 219.1, found: 219.1. The spectra data is consistent with the reported literature.[9]

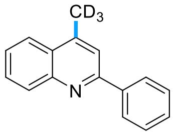

4-(methyl-*d*$_3$)-2-phenylquinoline (**10**). Following the general procedure A, the product was isolated by preparative TLC with Hex/EtOAc (10:1) as the eluent to give an oil (13.5 mg, 61%). **^{1}H NMR** (500 MHz, $CDCl_3$) δ 8.19 – 8.15 (m, 3H), 8.00 (dd, *J* = 8.4, 1.4 Hz, 1H), 7.74 – 7.71 (m, 2H), 7.57 – 7.51 (m, 3H), 7.48 – 7.44 (m, 1H).**^{13}C NMR** (126 MHz, $CDCl_3$) δ 157.1, 148.2, 144.6, 139.8, 130.3, 129.3, 129.2, 128.8 (X2), 127.6 (X2), 127.3, 126.0, 123.6, 119.8. **^{2}H NMR** (77 MHz, $CDCl_3$) δ 2.71. **GC-MS** (EI, m/z) for $C_{16}H_{10}D_3N$ calcd: 222.1, found: 222.1. The spectra data is consistent with the reported literature.[7]

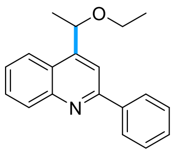

(*R*)-4-(1-ethoxyethyl)-2-phenylquinoline (**11**). Following the general procedure A, the product was isolated by preparative TLC with Hex/EtOAc (10:1) as the eluent to give an oil (19.7 mg, 71%). **^{1}H NMR** (500 MHz, $CDCl_3$) δ 8.22 – 8.18 (m, 3H), 8.11 (d, *J* = 7.1 Hz, 1H), 8.00 (s, 1H), 7.74 – 7.71 (m, 1H), 7.55 – 7.52 (m, 3H), 7.49 – 7.45 (m, 1H), 5.20 (q, *J* = 6.6 Hz, 1H), 3.54 – 3.48 (m, 2H), 1.64 (d, *J* = 6.6 Hz, 3H), 1.28 (t, *J* = 7.0 Hz, 3H). **^{13}C NMR** (126 MHz, $CDCl_3$) δ 157.4, 150.3, 148.7, 139.8, 130.7, 129.3, 129.2, 128.8 (X2), 127.6 (X2), 126.1, 125.1, 122.9, 115.4, 74.6, 64.8, 23.5, 15.5. **GC-MS** (EI, m/z) for $C_{19}H_{19}NO$ calcd: 277.1, found: 277.0. The spectra data is consistent with the reported literature.[7]

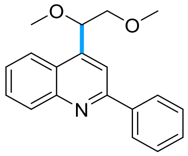

(*R*)-4-(1,2-dimethoxyethyl)-2-phenylquinoline (**12**). Following the general procedure A, the product was isolated by preparative TLC with Hex/EtOAc (10:1) as the eluent to give an oil (16.4 mg, 56%). **^{1}H NMR** (500 MHz, $CDCl_3$) δ 8.24 – 8.19 (m, 3H), 8.10 (d, *J* = 7.8 Hz, 1H), 8.01 (s, 1H), 7.76 – 7.73 (m, 1H), 7.58 – 7.53 (m, 3H), 7.49 – 7.46 (m, 1H), 5.22 (dd, *J* = 7.8, 3.0 Hz, 1H), 3.76 – 3.66 (m, 2H), 3.45 (s, 6H). **^{13}C NMR** (126 MHz, $CDCl_3$) δ 157.2, 148.7, 144.8, 139.6, 130.8, 129.5, 129.4, 128.9 (X2), 127.6 (X2), 126.5, 125.4, 122.6, 116.7, 80.1, 76.5, 59.4, 57.8. **GC-MS** (EI, m/z) for $C_{19}H_{19}NO_2$ calcd: 293.1, found 293.2. The spectra data is consistent with the reported literature.[7]

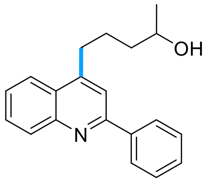

5-(2-phenylquinolin-4-yl)pentan-2-ol (**13**) Following the general procedure A, the product was isolated by preparative TLC with Hex/EtOAc (5:2) as the eluent to give an oil (20.4 mg, 70%). **^{1}H NMR** (500 MHz, $CDCl_3$) δ 8.19 (d, *J* = 8.4 Hz, 1H), 8.15 (d, *J* = 7.2 Hz, 2H), 8.03 (d, *J* = 8.3 Hz, 1H), 7.73 – 7.69 (m, 2H), 7.55 – 7.51 (m, 3H), 7.47 – 7.44 (m, 1H), 3.90 – 3.84 (m, 1H), 3.18 – 3.12 (m, 2H), 2.00 – 1.83 (m, 2H), 1.66 – 1.59 (m, 2H), 1.43 – 1.40 (br, 1H), 1.21 (d, *J* = 6.3 Hz, 3H). **^{13}C NMR** (126 MHz, $CDCl_3$) δ 157.1, 148.8, 148.5, 139.9, 130.5, 129.3, 129.2, 128.8 (X2), 127.6 (X2), 126.5, 126.1, 123.3, 118.8, 67.9, 39.1, 32.4, 26.4, 23.8. **GC-MS** (EI, m/z) for $C_{20}H_{21}NO$ calcd: 291.2, found: 291.1. The spectra data is consistent with the reported literature.[7]

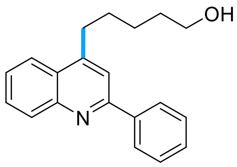

5-(2-phenylquinolin-4-yl)pentan-1-ol (**14**). Following the general procedure A, the product was isolated by preparative TLC with Hex/EtOAc (5:2) as the eluent to give an oil (22.1 mg, 76%). **^{1}H NMR** (500 MHz, $CDCl_3$) δ 8.18 (d, *J* = 7.2 Hz, 1H), 8.16 – 8.14 (m, 2H), 8.03 (d, *J* = 8.5 Hz, 1H), 7.73 – 7.69 (m, 2H), 7.56 – 7.51 (m, 3H), 7.48 – 7.44 (m, 1H), 3.68 (t, *J* = 6.4 Hz, 2H), 3.17 – 3.14 (m, 2H), 1.90 – 1.83 (m, 2H), 1.70 – 1.64 (m, 2H), 1.59 – 1.53 (m, 3H). **^{13}C NMR** (126 MHz, $CDCl_3$) δ 157.1, 149.0, 148.5, 139.9, 130.6, 129.2, 129.2, 128.8 (X2), 127.6 (X2), 126.5, 126.0, 123.4, 118.8, 62.8, 32.6, 32.6, 30.1, 25.9. **HRMS** ($M+H^+$) for $C_{20}H_{22}NO$ calcd: 292.1699, found: 292.1696. The compound is not reported in the literature.

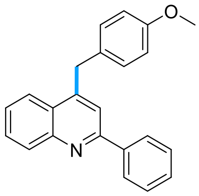

4-(4-methoxybenzyl)-2-phenylquinoline (**15**). Following the general procedure A, the product was isolated by preparative TLC with Hex/EtOAc (10:1) as the eluent to give a solid (13.0 mg, 40%). **^{1}H NMR** (500 MHz, $CDCl_3$) δ 8.19 (d, *J* = 8.3 Hz, 1H), 8.11 – 8.10 (m, 2H), 8.03 (d, *J* = 7.1 Hz, 1H), 7.72 – 7.68 (m, 1H), 7.63 (s, 1H), 7.52 – 7.48 (m, 3H), 7.46 – 7.43 (m, 1H), 7.17 – 7.15 (m, 2H), 6.87 – 6.85 (m, 2H), 4.45 (s, 2H), 3.79 (s, 3H). **^{13}C NMR** (126 MHz, $CDCl_3$) δ 158.3, 157.2, 148.6, 147.5, 139.8, 130.7, 130.5, 129.9 (X2), 129.3, 129.2, 128.8 (X2), 127.6 (X2), 126.6, 126.2, 123.7, 119.7, 114.2 (X2), 55.3, 37.7. **HRMS** ($M+H^+$) for $C_{23}H_{20}NO$ calcd: 326.1539, found: 326.1539. The spectra data is consistent with the reported literature.[7]

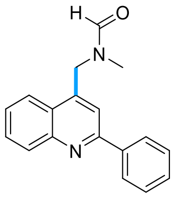

N-methyl-*N*-((2-phenylquinolin-4-yl)methyl)formamide (**16**), a mixture of 1:05 rotamers. Following the general procedure A, the product was isolated by preparative TLC with Hex/EtOAc/MeOH (1:1:0.2) as the eluent to give an oil (14.1 mg, 51%). **^{1}H NMR** (500 MHz, $CDCl_3$) δ 8.42 (s, 1H), 8.26 – 8.21 (m, 2H+0.5H), 8.16 – 8.13 (m, 2H+1H), 8.08 (d, *J* = 7.0 Hz, 1H), 7.92 (d, *J* = 8.4 Hz, 0.5H), 7.80 – 7.72 (m, 2H+0.5H), 7.65 (s, 0.5H), 7.62 – 7.52 (m, 3H+1.5H), 7.50 – 7.47 (m, 1H+0.5H), 5.06 (s, 2H), 4.98 (s, 1H), 2.98 (s, 1.5H), 2.90 (s, 3H). **^{13}C NMR** (126 MHz, $CDCl_3$) δ 163.4, 162.6, 157.3, 157.0, 148.7, 148.5, 141.8, 141.7, 139.4, 139.1, 130.9, 130.6, 130.0, 129.9, 129.7, 129.5, 129.0, 128.9, 127.6, 127.6, 127.0, 126.9, 125.6, 125.1, 123.2, 121.8, 119.0, 116.7, 50.1, 45.2, 34.4, 30.5. **GC-MS** (EI, m/z) for $C_{18}H_{16}N_2O$ calcd: 276.1, found 276.2. The spectra data is consistent with the reported literature.[7]

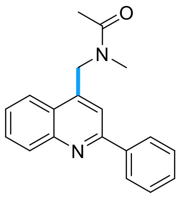

N-methyl-*N*-((2-phenylquinolin-4-yl)methyl)acetamide (**17**), a mixture of 1:05 rotamers. Following the general procedure A, the product was isolated by preparative TLC with Hex/EtOAc (1:1) as the eluent to give an oil (12.8 mg, 44%). **^{1}H NMR** (500 MHz, $CDCl_3$) δ 8.25 (d, *J* = 8.7 Hz, 0.5H), 8.20 (d, *J* = 8.6 Hz, 1H), 8.15 – 8.13 (m, 2H+1H), 8.07 (d, *J* = 8.4 Hz, 1H), 7.88 (d, *J* = 8.9 Hz, 0.5H), 7.80 – 7.77 (m, 0.5H), 7.76 – 7.73 (m, 1H), 7.67 (s, 1H), 7.62 – 7.52 (m, 3H+2H), 7.50 – 7.46 (m, 1H+0.5H), 5.14 (s, 2H), 5.07 (s, 1H), 3.15 (s, 1H), 2.98 (s, 3H), 2.25 (s, 3H), 2.15 (s, 1.5H). **^{13}C NMR** (126 MHz, $CDCl_3$) δ 171.5, 170.8, 157.6, 157.1, 148.6, 148.4, 143.1, 142.4, 139.6, 139.2, 130.9, 130.5, 129.9, 129.7, 129.7, 129.4, 129.0, 128.9, 127.6, 127.6, 126.8, 126.8, 125.8, 124.9, 123.3, 121.6, 118.5, 114.7, 51.5, 47.9, 35.6, 34.7, 21.9, 21.3. **GC-MS** (EI, m/z) for $C_{19}H_{18}N_2O$ calcd: 290.1, found: 290.1. The spectra data is consistent with the reported literature.[7]

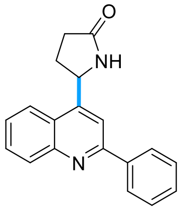

(*R*)-5-(2-phenylquinolin-4-yl)pyrrolidin-2-one (**18**). Following the general procedure A, the product was isolated by preparative TLC with Hex/EtOAc/MeOH (2:1:0.5) as the eluent to give a solid (11.5 mg, 40%). **^{1}H NMR** (500 MHz, $CDCl_3$) δ 8.25 (dd, *J* = 8.4, 1.3 Hz, 1H), 8.15 – 8.13 (m, 2H), 7.91 (d, *J* = 8.4 Hz, 1H), 7.87 (s, 1H), 7.79 – 7.75 (m, 1H), 7.60 – 7.57 (m, 1H), 7.54 – 7.45 (m, 3H), 6.22 (s, 1H), 5.57 – 5.54 (m, 1H), 2.95 – 2.88 (m, 1H), 2.51 (t, *J* = 8.1 Hz, 2H), 2.14 – 2.07 (m, 1H). **^{13}C NMR** (126 MHz, $CDCl_3$) δ 178.5, 157.4, 148.7, 148.4, 139.3, 130.9, 129.8, 129.7, 128.9 (X2), 127.6 (X2), 126.7, 124.2, 122.1, 113.6, 53.8, 29.7, 29.5. **GC-MS** (EI, m/z) for $C_{19}H_{16}N_2O$ calcd: 288.1, found: 288.1. The spectra data is consistent with the reported literature.[7]

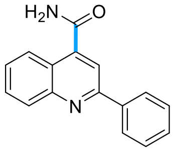

2-phenylquinoline-4-carboxamide (**19**). Following the general procedure A, the product was isolated by preparative TLC with Hex/EtOAc/MeOH (1:1:0.5) as the eluent to give a solid (9.7 mg, 39%). **^{1}H NMR** (500 MHz, $CDCl_3$) δ 8.30 (d, *J* = 9.2 Hz, 1H), 8.22 (d, *J* = 8.7 Hz, 1H), 8.18 – 8.17 (m, 2H), 8.01 (s, 1H), 7.80 – 7.77 (m, 1H), 7.63 – 7.60 (m, 1H), 7.56 – 7.53 (m, 2H), 7.51 – 7.49 (m, 1H), 6.02 (d, *J* = 46.3 Hz, 2H). **^{13}C NMR** (126 MHz, $CDCl_3$) δ 169.3, 156.8, 148.9, 141.6, 138.8, 130.3, 130.3, 129.8, 129.0 (X2), 127.5, 127.5 (X2), 124.9, 123.1, 116.7. **GC-MS** (EI, m/z) for $C_{16}H_{12}N_2O$ calcd: 248.1, found: 248.0. The spectra data is consistent with the reported literature.[10]

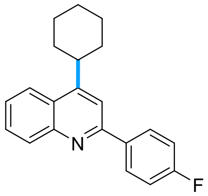

4-cyclohexyl-2-(4-fluorophenyl)quinoline (**20**). Following the general procedure A, the product was isolated by preparative TLC with Hex/EtOAc (10:1) as the eluent to give a solid (24.7 mg, 81%). **^{1}H NMR** (500 MHz, $CDCl_3$) δ 8.17 – 8.13 (m, 3H), 8.09 (d, *J* = 8.5 Hz, 1H), 7.72 – 7.68 (m, 2H), 7.55 – 7.52 (m, 1H), 7.23 – 7.18 (m, 2H), 3.40 – 3.35 (m, 1H), 2.10 – 2.07 (m, 2H), 1.98 – 1.95 (m, 2H), 1.90 – 1.87 (m, 1H), 1.64 – 1.55 (m, 4H), 1.39 – 1.34 (m, 1H). **^{13}C NMR** (126 MHz, $CDCl_3$) δ 163.7 (d, *J* = 247.6 Hz), 156.2, 154.1, 148.5, 136.4 (d, *J* = 2.7 Hz), 130.6 (X2), 129.5, 129.2 (d, *J* = 33.7 Hz), 125.9, 125.8, 122.9, 115.7 (d, *J* = 21.7 Hz), 115.1 (X2), 39.1, 33.7 (X2), 27.0 (X2), 26.3.**^{19}F NMR** (471 MHz, $CDCl_3$) δ -112.87 – -112.93 (m, 1F). **GC-MS** (EI, m/z) for $C_{21}H_{20}FN$ calcd: 305.2, found: 305.1. The spectra data is consistent with the reported literature.[7]

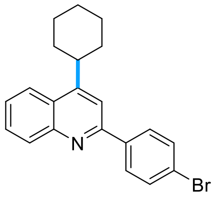

2-(4-bromophenyl)-4-cyclohexylquinoline (**21**). Following the general procedure A, the product was isolated by preparative TLC with Hex/EtOAc (10:1) as the eluent to give a solid (23.4 mg, 64%). **^{1}H NMR** (500 MHz, $CDCl_3$) δ 8.17 (d, *J* = 7.2 Hz, 1H), 8.09 (d, *J* = 7.2 Hz, 1H), 8.06 – 8.03 (m, 2H), 7.72 – 7.69 (m, 2H), 7.66 – 7.63 (m, 2H), 7.56 – 7.53 (m, 1H), 3.41 – 3.35 (m, 1H), 2.10 – 2.07 (m, 2H), 1.98 – 1.96 (m, 2H), 1.90 – 1.87 (m, 1H), 1.67 – 1.55 (m, 4H), 1.40 – 1.34 (m, 1H). **^{13}C NMR** (126 MHz, $CDCl_3$) δ 156.0, 154.3, 148.5, 139.1, 131.9 (X2), 130.7, 129.2, 129.2 (X2), 126.1, 126.0, 123.7, 122.9, 115.0, 39.2, 33.7 (X2), 27.0 (X2), 26.3. **GC-MS** (EI, m/z) for $C_{21}H_{20}BrN$ calcd: 365.1, found: 365.1. The spectra data is consistent with the reported literature.[7]

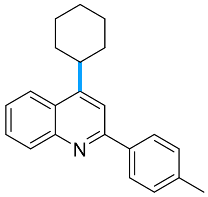

4-cyclohexyl-2-(*p*-tolyl)quinoline (**22**). Following the general procedure A, the product was isolated by preparative TLC with Hex/EtOAc (10:1) as the eluent to give an oil (20.5 mg, 68%). **^{1}H NMR** (500 MHz, $CDCl_3$) δ 8.17 (d, *J* = 8.4 Hz, 1H), 8.09 – 8.05 (m, 3H), 7.74 (s, 1H), 7.70 – 7.67 (m, 1H), 7.54 – 7.50 (m, 1H), 7.33 (d, *J* = 7.9 Hz, 2H), 3.40 – 3.34 (m, 1H), 2.44 (s, 3H), 2.10 – 2.07 (m, 2H), 1.98 – 1.95 (m, 2H), 1.90 – 1.86 (m, 1H), 1.67 – 1.54 (m, 4H), 1.40 – 1.34 (m, 1H). **^{13}C NMR** (126 MHz, $CDCl_3$) δ 157.3, 153.8, 148.6, 139.1, 137.5, 130.6, 129.5 (X2), 128.9, 127.5 (X2), 125.8, 125.7, 122.8, 115.3, 39.1, 33.7 (X2), 27.0 (X2), 26.4, 21.4. **GCMS** (EI, m/z) for $C_{22}H_{23}N$ calcd: 301.2, found: 301.1. The spectra data is consistent with the reported literature.[7]

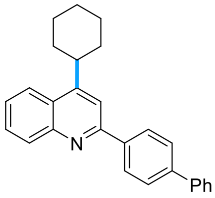

2-([1,1'-biphenyl]-4-yl)-4-cyclohexylquinoline (**23**). Following the general procedure A, the product was isolated by preparative TLC with Hex/EtOAc (10:1) as the eluent to give a solid (14.2 mg, 39%). **^{1}H NMR** (500 MHz, $CDCl_3$) δ 8.25 – 8.23 (m, 2H), 8.21 (d, *J* = 7.2 Hz, 1H), 8.10 (d, *J* = 7.3 Hz, 1H), 7.81 (s, 1H), 7.78 – 7.75 (m, 2H), 7.73 – 7.68 (m, 3H), 7.56 – 7.53 (m, 1H), 7.50 – 7.47 (m, 2H), 7.40 – 7.37 (m, 1H), 3.39 (tt, *J* = 11.5, 3.1 Hz, 1H), 2.12 – 2.09 (m, 2H), 1.99 – 1.97 (m, 2H), 1.91 – 1.88 (m, 1H), 1.70 – 1.55

(m, 4H), 1.44 – 1.36 (m, 1H). **^{13}C NMR** (126 MHz, $CDCl_3$) δ 156.9, 154.0, 148.7, 141.9, 140.7, 139.1, 130.7, 129.1, 128.9 (X2), 128.0 (X2), 127.6, 127.5 (X2), 127.2 (X2), 125.9, 125.9, 122.9, 115.4, 39.2, 33.7 (X2), 27.0 (X2), 26.4. **HRMS** (M+H$^+$) for $C_{27}H_{26}N$ calcd: 364.2060, found: 364.2056. The spectra data is consistent with the reported literature.[7]

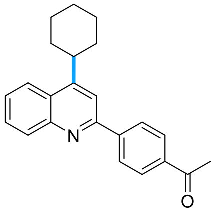

1-(4-(4-cyclohexylquinolin-2-yl)phenyl)ethan-1-one (**24**). Following the general procedure A, the product was isolated by preparative TLC with Hex/EtOAc (5:2) as the eluent to give a solid (18.8 mg, 57%). **^{1}H NMR** (500 MHz, $CDCl_3$) δ 8.26 (d, J = 8.5 Hz, 2H), 8.20 (d, J = 7.1 Hz, 1H), 8.12 – 8.10 (m, 3H), 7.79 (s, 1H), 7.74 – 7.71 (m, 1H), 7.59 – 7.55 (m, 1H), 3.42 – 3.37 (m, 1H), 2.67 (s, 3H), 2.11 – 2.08 (m, 2H), 2.00 – 1.95 (m, 2H), 1.90 – 1.88 (m, 1H), 1.68 – 1.55 (m, 4H), 1.41 – 1.35 (m, 1H). **^{13}C NMR** (126 MHz, $CDCl_3$) δ 197.9, 155.9, 154.4, 148.6, 144.5, 137.2, 130.9, 129.3, 128.8 (X2), 127.7 (X2), 126.4, 126.2, 122.9, 115.4, 39.2, 33.7 (X2), 27.0 (X2), 26.8, 26.3. **GC-MS** (EI, m/z) for $C_{23}H_{23}NO$ calcd: 329.2, found: 329.2. The spectra data is consistent with the reported literature.[7]

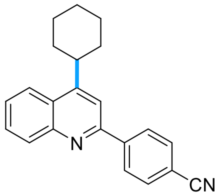

4-(4-cyclohexylquinolin-2-yl)benzonitrile (**25**). Following the general procedure A, the product was isolated by preparative TLC with Hex/EtOAc (5:1) as the eluent to give a solid (18.1 mg, 58%). **^{1}H NMR** (500 MHz, $CDCl_3$) δ 8.28 (d, J = 8.4 Hz, 2H), 8.18 (d, J = 7.2 Hz, 1H), 8.12 (d, J = 7.2 Hz, 1H), 7.81 (d, J = 8.5 Hz, 2H), 7.75 – 7.72 (m, 2H), 7.60 – 7.57 (m, 1H), 3.42 – 3.37 (m, 1H), 2.09 – 2.07 (m, 2H), 1.99 – 1.97 (m, 2H), 1.91 – 1.88 (m, 1H), 1.67 – 1.57 (m, 4H), 1.42 – 1.35 (m, 1H). **^{13}C NMR** (126 MHz, $CDCl_3$) δ 154.9, 154.7, 148.6, 144.4, 132.6 (X2), 130.9, 129.5, 128.1 (X2), 126.7, 126.2, 123.0, 118.9, 115.1, 112.5, 39.2, 33.7 (X2), 26.9 (X2), 26.3. **GCMS** (EI, m/z) for $C_{22}H_{20}N_2$ calcd: 312.2, found: 312.1. The spectra data is consistent with the reported literature.[7]

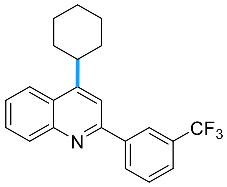

4-cyclohexyl-2-(3-(trifluoromethyl)phenyl)quinoline (**26**). Following the general procedure A, the product was isolated by preparative TLC with Hex/EtOAc (5:1) as the eluent to give a solid (26.0 mg, 73%). **^{1}H NMR** (500 MHz, $CDCl_3$) δ 8.43 (s, 1H), 8.34 (d, J = 7.8 Hz, 1H), 8.20 (d, J = 7.2 Hz, 1H), 8.11 (d, J = 7.2 Hz, 1H), 7.75 – 7.71 (m, 3H), 7.66 – 7.63 (m, 1H), 7.59 – 7.56 (m, 1H), 3.43 – 3.37 (m, 1H), 2.11 – 2.08 (m, 2H), 2.00 – 1.96 (m, 2H), 1.91 – 1.87 (m, 1H), 1.69 – 1.55 (m, 4H), 1.44 – 1.37 (m, 1H). **^{13}C NMR** (126 MHz, $CDCl_3$) δ 155.6, 154.6, 148.6, 141.0, 131.2 (q, J = 32.6 Hz), 130.8, 130.1, 129.3, 129.2, 126.4, 126.1, 125.7 (q, J = 3.7 Hz), 124.4 (q, J = 3.7 Hz), 124.3 (q, J = 273.8 Hz), 122.9, 115.0, 39.2, 33.7 (X2), 27.0 (X2), 26.3. **^{19}F NMR** (471 MHz, $CDCl_3$) δ -62.45 (s, 3F). **HRMS** (M+H$^+$) for $C_{22}H_{21}F_3N$ calcd: 356.1621, found: 356.1616. The compound is not reported in the literature.

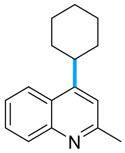

4-cyclohexyl-2-methylquinoline (**27**). Following the general procedure A, the product was isolated by preparative TLC with Hex/EtOAc (10:1) as the eluent to give an oil (10.8 mg, 48%). **^{1}H NMR** (500 MHz, $CDCl_3$) δ 8.03 (d, *J* = 8.8 Hz, 2H), 7.67 – 7.63 (m, 1H), 7.50 – 7.47 (m, 1H), 7.17 (s, 1H), 3.32 – 3.27 (m, 1H), 2.72 (s, 3H), 2.03 – 2.00 (m, 2H), 1.95 – 1.92 (m, 2H), 1.87 – 1.84 (m, 1H), 1.57 – 1.52 (m, 4H), 1.36 – 1.31 (m, 1H). **^{13}C NMR** (126 MHz, $CDCl_3$) δ 158.8, 153.3, 148.2, 129.6, 128.8, 125.3, 125.2, 122.8, 118.3, 38.8, 33.6 (X2), 27.0 (X2), 26.3, 25.6. **GC-MS** (EI, m/z) for $C_{16}H_{19}N$ calcd: 225.2, found: 225.1. The spectra data is consistent with the reported literature.[11]

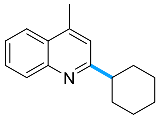

2-cyclohexyl-4-methylquinoline (**28**) Following the general procedure A, the product was isolated by preparative TLC with Hex/EtOAc (10:1) as the eluent to give an oil (13.1 mg, 58%). **^{1}H NMR** (500 MHz, $CDCl_3$) δ 8.04 (dd, *J* = 8.4, 1.3 Hz, 1H), 7.94 (dd, *J* = 8.4, 1.4 Hz, 1H), 7.68 – 7.65 (m, 1H), 7.51 – 7.48 (m, 1H), 7.17 (s, 1H), 2.90 – 2.85 (m, 1H), 2.68 (s, 3H), 2.03 – 1.99 (m, 2H), 1.91 – 1.87 (m, 2H), 1.81 – 1.77 (m, 1H), 1.67 – 1.58 (m, 2H), 1.51 – 1.43 (m, 2H), 1.37 – 1.32 (m, 1H). **^{13}C NMR** (126 MHz, $CDCl_3$) δ 166.5, 147.6, 144.2, 129.5, 128.9, 127.1, 125.4, 123.6, 120.3, 47.6, 32.8 (X2), 26.6 (X2), 26.1, 18.9. **GC-MS** (EI, m/z) for $C_{16}H_{19}N$ calcd: 225.2, found: 225.2. The spectra data is consistent with the reported literature.[12]

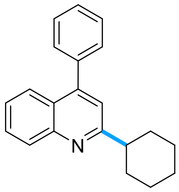

2-cyclohexyl-4-phenylquinoline (**29**). Following the general procedure A, the product was isolated by preparative TLC with Hex/EtOAc (5:1) as the eluent to give an oil (19.5 mg, 68%). **^{1}H NMR** (500 MHz, $CDCl_3$) δ 8.11 (d, *J* = 7.2 Hz, 1H), 7.86 (dd, *J* = 8.5, 1.4 Hz, 1H), 7.70 – 7.66 (m, 1H), 7.54 – 7.47 (m, 5H), 7.44 – 7.41 (m, 1H), 7.27 (s, 1H), 2.98 – 2.93 (m, 1H), 2.09 – 2.06 (m, 2H), 1.92 – 1.88 (m, 2H), 1.81 – 1.78 (m, 1H), 1.71 – 1.62 (m, 2H), 1.53 – 1.44 (m, 2H), 1.37 – 1.31 (m, 1H). **^{13}C NMR** (126 MHz, $CDCl_3$) δ 166.4, 148.6, 148.3, 138.6, 129.6 (X2), 129.4, 129.1, 128.5 (X2), 128.2, 125.7, 125.6, 125.5, 119.9, 47.7, 32.9 (X2), 26.6 (X2), 26.1. **GC-MS** (EI, m/z) for $C_{21}H_{21}N$ calcd: 287.2 found: 287.2. The spectra data is consistent with the reported literature [6]

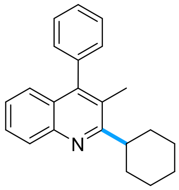

2-cyclohexyl-3-methyl-4-phenylquinoline (**30**). Following the general procedure A, the product was isolated by preparative TLC with Hex/EtOAc (5:1) as the eluent to give an oil (25.0 mg, 83%). **^{1}H NMR** (500 MHz, $CDCl_3$) δ 8.05 (d, *J* = 8.4 Hz, 1H), 7.59 – 7.56 (m, 1H), 7.53 – 7.50 (m, 2H), 7.48 –7.44 (m, 1H), 7.31 – 7.27 (m, 2H), 7.25 – 7.23 (m, 2H), 3.12 – 3.06 (m, 1H), 2.22 (s, 3H), 1.94 – 1.88 (m, 6H), 1.81 – 1.77 (m, 1H), 1.49 – 1.41 (m, 3H). **^{13}C NMR** (126 MHz, $CDCl_3$) δ 165.6, 146.4, 146.3, 138.3, 129.5 (X2), 129.1, 128.5

(X2), 127.8, 127.5, 126.5, 126.3, 126.0, 125.3, 43.1, 32.0 (X2), 26.9 (X2), 26.2, 16.3. **HRMS**: (M+H$^+$) for $C_{22}H_{24}N$ calcd: 302.1903, found: 302.1902. The compound is not reported in the literature.

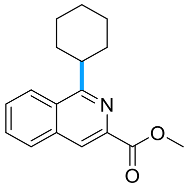

methyl 1-cyclohexylisoquinoline-3-carboxylate (**31**). Following the general procedure A, the product was isolated by preparative TLC with Hex/EtOAc (5:1) as the eluent to give a solid (17.2 mg, 64%). **^{1}H NMR** (500 MHz, $CDCl_3$) δ 8.39 (s, 1H), 8.28 – 8.26 (m, 1H), 7.95 – 7.93 (m, 1H), 7.73 – 7.69 (m, 2H), 4.03 (s, 3H), 3.60 – 3.54 (m, 1H), 2.02 – 1.93 (m, 6H), 1.83 – 1.79 (m, 1H), 1.59 – 1.50 (m, 2H), 1.46 – 1.37 (m, 1H).**^{13}C NMR** (126 MHz, $CDCl_3$). δ 166.9, 166.1, 140.7, 136.0, 130.1, 129.1, 129.0, 127.8, 125.0, 122.4, 52.7, 42.1, 32.2 (X2), 26.8 (X2), 26.1. **GCMS**: (EI, m/z) for $C_{17}H_{19}NO_2$ calcd: 269.1, found: 269.1. The spectra data is consistent with the reported literature.[13]

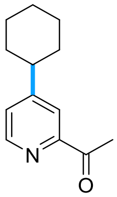

1-(4-cyclohexylpyridin-2-yl)ethan-1-one (**32a**). Following the general procedure A, the product was isolated by preparative TLC with Hex/EtOAc (10:1) as the eluent to give a solid (8.1 mg, 40%). **^{1}H NMR** (500 MHz, $CDCl_3$) δ 8.56 (d, *J* = 5.7 Hz, 1H), 7.91 (d, *J* = 1.8 Hz, 1H), 7.30 (dd, *J* = 5.0, 1.9 Hz, 1H), 2.72 (s, 3H), 2.60 – 2.55 (m, 1H), 1.89 – 1.85 (m, 4H), 1.80 – 1.75 (m, 1H), 1.48 – 1.35 (m, 4H), 1.31 – 1.27 (m, 1H). **^{13}C NMR** (126 MHz, $CDCl_3$) δ 200.5, 157.8, 153.7, 149.0, 125.8, 120.3, 43.9, 33.4 (X2), 26.5 (X2), 26.0, 25.8. **HRMS** (M+Na$^+$) for $C_{13}H_{17}NNaO$ calcd: 226.1198, found: 226.1202. The spectra data is consistent with the reported literature.[14]

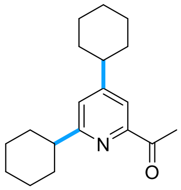

1-(4,6-dicyclohexylpyridin-2-yl)ethan-1-one (**32b**). Following the general procedure A, the product was isolated by preparative TLC with Hex/EtOAc (10:1) as the eluent to give a solid (7.1 mg, 25%). **^{1}H NMR** (500 MHz, $CDCl_3$) δ 7.70 (d, *J* = 1.6 Hz, 1H), 7.13 (d, *J* = 1.6 Hz, 1H), 2.71 (s, 3H), 2.55 – 2.50 (m, 1H), 1.97 – 1.94 (m, 2H), 1.86 – 1.84 (m, 6H), 1.78 – 1.74 (m, 2H), 1.59 – 1.56 (m, 3H), 1.45 – 1.36 (m, 6H), 1.32 – 1.28 (m, 2H). **^{13}C NMR** (126 MHz, $CDCl_3$) δ 201.5, 166.0, 157.6, 153.1, 123.3, 117.5, 46.2, 44.1, 33.6 (X2), 32.9 (X2), 26.5 (X2), 26.5 (X2), 26.1, 25.9, 25.9. **HRMS** (M+Na$^+$) for $C_{19}H_{27}NNaO$ calcd: 308.1979, found: 308.1985. The spectra data is consistent with the reported literature.[14]

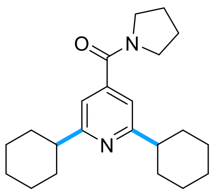

(2,6-dicyclohexylpyridin-4-yl)(pyrrolidin-1-yl)methanone (**33**). Following the general procedure A, the product was isolated by preparative TLC with Hex/EtOAc (5:2) as the eluent to give a solid (15.3 mg, 45%). **^{1}H NMR** (500 MHz, $CDCl_3$) δ 7.02 (s, 2H), 3.63 (t, *J* = 7.0 Hz, 2H), 3.35 (t, *J* = 6.6 Hz, 2H), 2.73 – 2.67 (m, 2H), 1.98 – 1.95 (m, 6H), 1.91 – 1.88 (m, 2H), 1.84 – 1.81 (m, 4H), 1.75 – 1.72 (m, 2H), 1.49 – 1.36 (m, 6H), 1.30 – 1.24 (m, 4H). **^{13}C NMR** (126 MHz, $CDCl_3$) δ 168.6, 166.3 (X2), 145.2, 115.2 (X2), 49.3, 46.6 (X2), 46.1, 33.0 (X4), 26.5 (X4), 26.3, 26.1 (X2), 24.4. **GC-MS** (EI, m/z) for $C_{22}H_{32}N_2O$ calcd: 340.3, found: 340.2. The spectra data is consistent with the reported literature.[15]

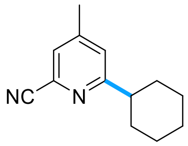

6-cyclohexyl-4-methylpicolinonitrile (**34**). Following the general procedure A, the product was isolated by preparative TLC with Hex/EtOAc (10:1) as the eluent to give an oil (10.2 mg, 51%). **^{1}H NMR** (500 MHz, $CDCl_3$) δ 7.33 (s, 1H), 7.17 (s, 1H), 2.73 – 2.67 (m, 1H), 2.38 (s, 3H), 1.92 – 1.84 (m, 4H), 1.77 – 1.73 (m, 1H), 1.54 – 1.46 (m, 2H), 1.44 – 1.35 (m, 2H), 1.32 – 1.26 (m, 1H).**^{13}C NMR** (126 MHz, $CDCl_3$) δ 168.3, 148.6, 133.0, 126.9, 125.4, 117.8, 46.3, 32.6 (X2), 26.4 (X2), 25.9, 20.9. **HRMS** (M+Na$^+$) for $C_{13}H_{16}N_2Na$ calc. 223.1206, found 223.1201. The compound is not reported in the literature.

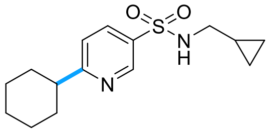

6-cyclohexyl-*N*-(cyclopropylmethyl)pyridine-3-sulfonamide (**35**). Following the general procedure A with 4 equiv of TFA, the product was isolated by preparative TLC with Hex/EtOAc (2:1) as the eluent to give a solid (12.9 mg, 44%). **^{1}H NMR** (500 MHz, $CDCl_3$) δ 8.97 (d, *J* = 3.2 Hz, 1H), 8.04 (dd, *J* = 8.3, 2.4 Hz, 1H), 7.29 (d, *J* = 7.5 Hz, 1H), 4.60 – 4.57 (m, 1H), 2.90 – 2.88 (m, 2H), 2.81 – 2.76 (m, 1H), 1.97 – 1.94 (m, 2H), 1.90 – 1.86 (m, 2H), 1.79 – 1.76 (m, 1H), 1.56 – 1.49 (m, 2H), 1.47 – 1.38 (m, 2H), 1.33 – 1.25 (m, 1H), 0.94 – 0.88 (m, 1H), 0.51 – 0.47 (m, 2H), 0.14 – 0.11 (m, 2H). **^{13}C NMR** (126 MHz, $CDCl_3$) δ 171.0, 147.5, 135.1, 134.1, 121.1, 48.4, 46.7, 32.6 (X2), 26.4 (X2), 25.9, 10.8, 3.6 (X2). **HRMS** (M-H$^+$) for $C_{15}H_{21}O_2N_2S$ calcd. 293.1329, found: 293.1329 The compound is not reported in the literature.

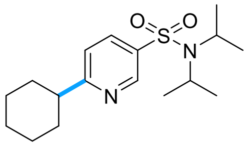

6-cyclohexyl-*N*,*N*-diisopropylpyridine-3-sulfonamide (**36**). Following the general procedure A with 4 equiv of TFA, the product was isolated by preparative TLC with Hex/EtOAc (2:1) as the eluent to give a solid (12.3 mg, 38%). **^{1}H NMR** (500 MHz, $CDCl_3$) δ 8.97 (d, *J* = 1.7 Hz, 1H), 8.03 (dd, *J* = 8.2, 2.4 Hz, 1H),7.24 (d, *J* = 8.3 Hz, 1H), 3.77 – 3.68 (m, 2H), 2.80 – 2.74 (m, 1H), 1.97 – 1.94 (m, 2H), 1.89 – 1.85 (m, 2H), 1.78 – 1.75 (m, 1H), 1.54 – 1.48 (m, 2H), 1.46 – 1.37 (m, 2H), 1.28 – 1.27 (m, 13H).**^{13}C NMR** (126 MHz, $CDCl_3$) δ 170.2, 147.6, 136.4, 135.1, 120.8, 48.8 (X2), 46.6, 32.7 (X2), 26.4 (X2), 25.9, 22.0 (X4). **GC-MS** (EI, m/z) for $C_{17}H_{28}N_2O_2S$ calcd: 324.2, found: 324.2. The spectra data is consistent with the reported literature.[7]

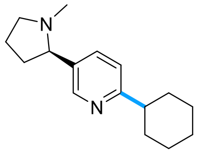

(*R*)-2-cyclohexyl-5-(1-methylpyrrolidin-2-yl)pyridine (**37**). Following the general procedure A with 4 equiv of TFA, the product was isolated by preparative TLC with Hex/EtOAc/MeOH (2:1:0.5) as the eluent to give a solid (16.8 mg, 69%). **^{1}H NMR** (500 MHz, $CDCl_3$) δ 8.41 (d, *J* = 2.4 Hz, 1H), 7.63 – 7.61 (m, 1H), 7.12 (d, *J* = 8.1 Hz, 1H), 3.26 – 3.22 (m, 1H), 3.06 – 3.03 (m, 1H), 2.72 – 2.66 (m, 1H), 2.32 – 2.26 (m, 1H), 2.19 – 2.14 (m, 4H), 1.97 – 1.94 (m, 2H), 1.87 – 1.83 (m, 3H), 1.76 – 1.73 (m, 2H), 1.55 – 1.36 (m, 5H), 1.32 – 1.27 (m, 1H). **^{13}C NMR** (126 MHz, $CDCl_3$) δ 165.5, 148.8 (X2), 135.2, 120.9, 68.7, 57.1, 46.3, 40.4, 35.0, 33.0, 29.7, 26.6 (X2), 26.1, 22.5. **GCMS** (EI, m/z) for $C_{16}H_{24}N_2$ calcd: 244.2, found: 244.2. The spectra data is consistent with the reported literature.[16]

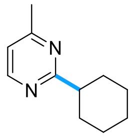

2-cyclohexyl-4-methylpyrimidine (**38a**). Following the general procedure A with 4 equiv of TFA, the product was isolated by preparative TLC with Hex/EtOAc (10:1) as the eluent to give an oil (7.9 mg, 45%). **^{1}H NMR** (500 MHz, $CDCl_3$) δ 8.51 (d, *J* = 5.1 Hz, 1H), 6.95 (d, *J* = 5.0 Hz, 1H), 2.86 – 2.80 (m, 1H), 2.49 (s, 3H), 1.99 – 1.96 (m, 2H), 1.87 – 1.83 (m, 2H), 1.75 – 1.71 (m, 1H), 1.66 – 1.58 (m, 2H), 1.45 – 1.36 (m, 2H), 1.34 – 1.29 (m, 1H). **^{13}C NMR** (126 MHz, $CDCl_3$) δ 174.3, 166.8, 156.6, 118.0, 47.6, 32.0 (X2), 26.3 (X2), 26.0, 24.3. **HRMS** ($M+H^+$) for $C_{11}H_{17}N_2$ calcd: 177.1386, found: 177.1384. The compound is not reported in the literature.

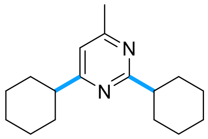

2,4-dicyclohexyl-6-methylpyrimidine (**38b**). Following the general procedure A with 4 equiv of TFA, the product was isolated by preparative TLC with Hex/EtOAc (10:1) as the eluent to give an oil (6.2 mg, 24%). **^{1}H NMR** (500 MHz, $CDCl_3$) δ 6.79 (s, 1H), 2.82 – 2.76 (m, 1H), 2.61 – 2.55 (m, 1H), 2.45 (s, 3H), 1.96 – 1.91 (m, 4H), 1.85 – 1.81 (m, 4H), 1.76 – 1.70 (m, 2H), 1.68 – 1.60 (m, 2H), 1.50 – 1.36 (m, 6H), 1.35 – 1.27 (m, 2H). **^{13}C NMR** (126 MHz, $CDCl_3$) δ 174.1, 173.7, 166.3, 114.6, 47.6, 45.9, 32.2 (X2), 32.0 (X2), 26.3 (X4), 26.0 (X2), 24.3. **HRMS** ($M+H^+$) for $C_{17}H_{27}N_2$ calcd: 259.2169, found: 259.2173. The compound is not reported in the literature.

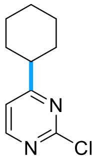

2-chloro-4-cyclohexylpyrimidine (**39a**). Following the general procedure A with 4 equiv of TFA, the product was isolated by preparative TLC with Hex/EtOAc (10:1) as the eluent to give an oil (7.5 mg, 38%). **^{1}H NMR** (500 MHz, $CDCl_3$) δ 8.49 (d, *J* = 5.1 Hz, 1H), 7.10 (d, *J* = 5.1 Hz, 1H), 2.70 – 2.64 (m, 1H), 1.97 – 1.94 (m, 2H), 1.89 – 1.85 (m, 2H), 1.77 – 1.74 (m, 1H), 1.51 – 1.46 (m, 2H), 1.44 – 1.35 (m, 2H), 1.32 – 1.27 (m, 1H). **^{13}C NMR** (126 MHz, $CDCl_3$) δ 178.6, 161.2, 159.3, 117.0, 45.9, 31.9 (X2), 26.1 (X2), 25.7. **HRMS** ($M+H^+$) for $C_{10}H_{14}ClN_2$ calcd: 197.0840, found: 197.0836. The spectra data is consistent with the reported literature.[7]

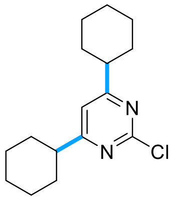

2-chloro-4,6-dicyclohexylpyrimidine (**39b**) Following the general procedure A with 4 equiv of TFA, the product was isolated by preparative TLC with Hex/EtOAc (10:1) as the eluent to give an oil (6.4 mg, 23%). **^{1}H NMR** (500 MHz, $CDCl_3$) δ 6.92 (s, 1H), 2.66 – 2.59 (m, 2H), 1.97 – 1.90 (m, 4H), 1.88 – 1.84 (m, 4H), 1.78 – 1.72 (m, 2H), 1.53 – 1.45 (m, 4H), 1.42 – 1.34 (m, 4H), 1.32 – 1.27 (m, 2H). **^{13}C NMR** (126 MHz, $CDCl_3$) δ 178.2 (X2), 160.7, 114.0, 45.9 (X2), 32.0 (X4), 26.1 (X4), 25.8 (X2). **HRMS** ($M+H^+$) for $C_{16}H_{24}ClN_2$ calcd: 279.1623, found: 279.1616. The spectra data is consistent with the reported literature.[7]

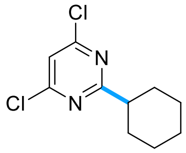

4,6-dichloro-2-cyclohexylpyrimidine (**40**). Following the general procedure A with 4 equiv of TFA, the product was isolated by preparative TLC with Hex/EtOAc (10:1) as the eluent to give an oil (13.6 mg, 59%). **^{1}H NMR** (500 MHz, $CDCl_3$) δ 7.22 (s, 1H), 2.88 – 2.82 (m, 1H), 2.00 – 1.97 (m, 2H), 1.87 – 1.83 (m, 2H), 1.75 – 1.71 (m, 1H), 1.65 – 1.57 (m, 2H), 1.43 – 1.29 (m, 3H). **^{13}C NMR** (126 MHz, $CDCl_3$) δ 176.5, 161.7 (X2), 118.5, 47.2, 31.5 (X2), 25.9 (X2), 25.7. **HRMS** ($M+H^+$) for $C_{10}H_{13}Cl_2N_2$ calcd: 231.0450, found: 231.0450. The spectra data is consistent with the reported literature.[7]

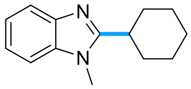

2-cyclohexyl-1-methyl-1H-benzo[*d*]imidazole (**41**). Following the general procedure A, with 4 equiv of TFA, the product was isolated by preparative TLC with Hex/EtOAc (5:2) as the eluent to give an oil (9.6 mg, 45%). **^{1}H NMR** (500 MHz, $CDCl_3$) δ 7.76 – 7.73 (m, 1H), 7.31 – 7.28 (m, 1H), 7.24 – 7.21 (m, 2H), 3.75 (s, 3H), 2.88 – 2.82 (m, 1H), 2.02 – 1.99 (m, 2H), 1.94 – 1.91 (m, 2H), 1.83 – 1.77 (m, 3H), 1.45 – 1.35 (m, 3H). **^{13}C NMR** (126 MHz, $CDCl_3$) δ 159.0, 142.5, 135.7, 121.9, 121.7, 119.3, 108.8, 36.4, 31.5 (X2), 29.6, 26.4 (X2), 25.8. **GC-MS** (EI, m/z) for $C_{14}H_{18}N_2$ calcd: 214.1, found: 214.1. The spectra data is consistent with the reported literature.[17]

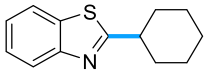

2-cyclohexylbenzo[*d*]thiazole (**42**). Following the general procedure A, with 4 equiv of TFA, the product was isolated by preparative TLC with Hex/EtOAc (10:1) as the eluent to give an oil (9.6 mg, 44%). **^{1}H NMR** (500 MHz, $CDCl_3$) δ 7.97 (d, *J* = 8.3 Hz, 1H), 7.85 (d, *J* = 7.5 Hz, 1H), 7.46 – 7.42 (m, 1H), 7.36 – 7.32 (m, 1H), 3.13 – 3.09 (m, 1H), 2.22 – 2.19 (m, 2H), 1.91 – 1.87 (m, 2H), 1.79 – 1.75 (m, 1H), 1.68 – 1.62 (m, 2H), 1.49 – 1.41 (m, 2H), 1.36 – 1.29 (m, 1H). **^{13}C NMR** (126 MHz, $CDCl_3$) δ 177.6, 153.1, 134.6, 125.8, 124.5, 122.6, 121.6, 43.5, 33.4 (X2), 26.1 (X2), 25.8. **GC-MS** (EI, m/z) for $C_{13}H_{15}NS$ calcd: 217.1, found: 217.1. The spectra data is consistent with the reported literature.[17]

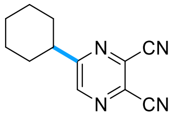

5-cyclohexylpyrazine-2,3-dicarbonitrile (**43**) Following the general procedure A, with 4 equiv of TFA, the product was isolated by preparative TLC with Hex/EtOAc (1:1) as the eluent to give an oil (7.6 mg, 36%). **^{1}H NMR** (500 MHz, $CDCl_3$) δ 8.74 (s, 1H), 2.95 – 2.89 (m, 1H), 1.97 – 1.90 (m, 4H), 1.82 – 1.79 (m, 1H), 1.63 – 1.56 (m, 2H), 1.45 – 1.39 (m, 2H), 1.35 – 1.29 (m, 1H). **^{13}C NMR** (126 MHz, $CDCl_3$) δ 165.7, 146.4, 133.1, 131.0, 113.1, 113.1, 44.3, 31.9 (X2), 25.9 (X2), 25.4. **GC-MS** (EI, m/z) for $C_{12}H_{12}N_4$ calcd: 212.1, found: 212.0. The spectra data is consistent with the reported literature.[7]

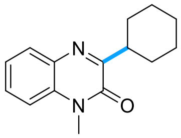

3-cyclohexyl-1-methylquinoxalin-2(1*H*)-one (**44**). Following the general procedure A, with 4 equiv of TFA, the product was isolated by preparative TLC with Hex/EtOAc (1:1) as the eluent to give a solid (16.9 mg, 70%). **^{1}H NMR** (500 MHz, $CDCl_3$) δ 7.84 (dd, *J* = 8.0, 1.5 Hz, 1H), 7.52 – 7.49 (m, 1H), 7.34 – 7.27 (m, 1H), 7.29 – 7.27 (m, 1H), 3.70 (s, 3H), 3.37 – 3.32 (m, 1H), 1.97 – 1.94 (m, 2H), 1.89 – 1.85 (m, 2H), 1.78 – 1.75 (m, 1H), 1.61 – 1.56 (m, 2H), 1.53 – 1.43 (m, 2H), 1.35 – 1.29 (m, 1H). **^{13}C NMR** (126 MHz, $CDCl_3$) δ 164.3, 154.6, 132.9, 132.9, 129.8, 129.4, 123.4, 113.5, 40.8, 30.5 (X2), 29.1, 26.3 (X2), 26.2. **GC-MS** (EI, m/z) for $C_{15}H_{18}N_2O$ calcd: 242.1, found: 242.1. The spectra data is consistent with the reported literature.[18]

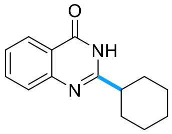

2-cyclohexylquinazolin-4(3*H*)-one (**45**). Following the general procedure A, with 4 equiv of TFA, the product was isolated by preparative TLC with Hex/EtOAc (1:1) as the eluent to give a solid (11.6 mg, 51%). **1H NMR** (500 MHz, $CDCl_3$) δ 9.87 (br, 1H), 8.26 (dd, *J* = 8.0, 1.6 Hz, 1H), 7.77 – 7.74 (m, 1H), 7.69 (dd, *J* = 8.2, 1.2 Hz, 1H), 7.47 – 7.44 (m, 1H), 2.67 – 2.62 (m, 1H), 2.08 – 2.04 (m, 2H), 1.94 – 1.90 (m, 2H), 1.81 – 1.78 (m, 1H), 1.69 – 1.61 (m, 2H), 1.48 – 1.34 (m, 3H). **^{13}C NMR** (126 MHz, $CDCl_3$) δ 163.0, 159.4, 149.3, 134.7, 127.4, 126.4, 126.3, 120.9, 44.8, 30.7 (X2), 25.9 (X2), 25.7. **GC-MS** (EI, m/z) for $C_{14}H_{16}N_2O$ calcd: 228.1, found: 228.0. The spectra data is consistent with the reported literature.[17]

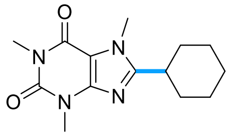

8-cyclohexyl-1,3,7-trimethyl-3,7-dihydro-1*H*-purine-2,6-dione (**46**). Following the general procedure A, with 4 equiv of TFA, the product was isolated by preparative TLC with Hex/EtOAc (1:1) as the eluent to give a solid (14.9 mg, 54%). **^{1}H NMR** (500 MHz, $CDCl_3$) δ 3.92 (s, 3H), 3.57 (s, 3H), 3.39 (s, 3H), 2.70 (tt, *J* = 11.5, 3.5 Hz, 1H), 1.91 – 1.85 (m, 4H), 1.78 – 1.64 (m, 3H), 1.40 – 1.31 (m, 3H). **^{13}C NMR** (126 MHz, $CDCl_3$) δ 158.0, 155.5, 151.8, 148.1, 107.0, 35.8, 31.4, 30.9 (X2), 29.7, 27.8, 26.0 (X2), 25.6. **GC-MS** (EI, m/z) for $C_{14}H_{20}N_4O_2$ calcd: 276.2, found: 276.1. The spectra data is consistent with the reported literature.[19]

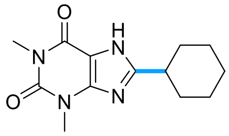

8-cyclohexyl-1,3-dimethyl-3,7-dihydro-1*H*-purine-2,6-dione (**47**). Following the general procedure A, with 4 equiv of TFA, the product was isolated by preparative TLC with Hex/EtOAc (1:1) as the eluent to give a solid (15.2 mg, 58%). **^{1}H NMR** (500 MHz, $CDCl_3$) δ 11.97 (br, 1H), 3.63 (s, 3H), 3.48 (s, 3H), 2.92 – 2.86 (m, 1H), 2.10 – 2.07 (m, 2H), 1.90 – 1.86 (m, 2H), 1.79 – 1.75 (m, 1H), 1.68 – 1.64 (m, 2H), 1.46 – 1.37 (m,

2H), 1.35 – 1.28 (m, 1H). **^{13}C NMR** (126 MHz, $CDCl_3$) δ 159.7, 155.6, 151.7, 149.1, 106.5, 38.8, 31.4 (X2), 30.2, 28.3, 26.0 (X2), 25.7. **HRMS** (M+Na$^+$) for $C_{13}H_{18}N_4NaO_2$ calcd: 285.1322, found: 285.1316. The spectra data is consistent with the reported literature.[20]

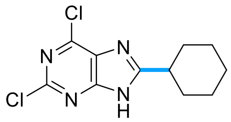

2,6-dichloro-8-cyclohexyl-9*H*-purine (**48**). Following the general procedure A, with 4 equiv of TFA, the product was isolated by preparative TLC with Hex/EtOAc (2:1) as the eluent to give a solid (15.7 mg, 58%). **^{1}H NMR** (500 MHz, $CDCl_3$) δ 11.37 (br, 1H), 3.10 – 3.04 (m, 1H), 2.18 – 2.15 (m, 2H), 1.95 – 1.90 (m, 2H), 1.81 – 1.70 (m, 3H), 1.49 – 1.40 (m, 2H), 1.38 – 1.32 (m, 1H). **^{13}C NMR** (126 MHz, $CDCl_3$) δ 163.1, 154.4, 151.3, 150.2, 131.2, 38.9, 31.3 (X2), 25.7 (X2), 25.4. **GCMS** (EI, m/z) for $C_{11}H_{12}Cl_2N_4$ calcd. 270.0, found. 270.1. The spectra data is consistent with the reported literature.[15]

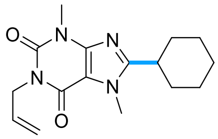

1-allyl-8-cyclohexyl-3,7-dimethyl-3,7-dihydro-1*H*-purine-2,6-dione (**49**). Following the general procedure A, with 4 equiv of TFA, the product was isolated by preparative TLC with Hex/EtOAc (2:1) as the eluent to give a solid (14.5 mg, 48%). **^{1}H NMR** (500 MHz, $CDCl_3$)5.96 – 5.88 (m, 1H), 5.27 – 5.15 (m, 2H), 4.63 – 4.61 (m, 2H), 3.92 (s, 3H), 3.57 (s, 3H), 2.73 – 2.67 (m, 1H), 1.91 – 1.84 (m, 4H), 1.78 – 1.75 (m, 1H), 1.72 – 1.63 (m, 2H), 1.40 – 1.32 (m, 3H). **^{13}C NMR** (126 MHz, $CDCl_3$) δ 158.1, 155.0, 151.4, 148.3, 132.6, 117.2, 107.0, 43.2, 35.8, 31.4, 30.9 (X2), 29.7, 26.0 (X2), 25.6. **HRMS** (M+Na$^+$) for $C_{16}H_{22}N_4NaO_2$ calcd: 325.1635, found: 325.1635. The compound is not reported in the literature.

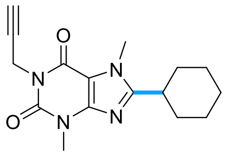

8-cyclohexyl-3,7-dimethyl-1-(prop-2-yn-1-yl)-3,7-dihydro-1*H*-purine-2,6-dione (**50**). Following the general procedure A, with 4 equiv of TFA, the product was isolated by preparative TLC with Hex/EtOAc (2:1) as the eluent to give a solid (21.9 mg, 73%). **^{1}H NMR** (500 MHz, $CDCl_3$) δ 4.79 (d, *J* = 2.5 Hz, 2H), 3.93 (s, 3H), 3.58 (s, 3H), 2.73 – 2.67 (m, 1H), 2.16 (t, *J* = 2.4 Hz, 1H), 1.90 – 1.84 (m, 4H), 1.78 – 1.75 (m, 1H), 1.72 – 1.64 (m, 2H), 1.42 – 1.32 (m, 3H). **^{13}C NMR** (126 MHz, $CDCl_3$) δ 158.5, 154.3, 151.0, 148.5, 106.9, 79.0, 70.2, 35.8, 31.5, 30.9 (X2), 30.3, 29.8, 26.0 (X2), 25.5. **HRMS** (M+Na$^+$) for $C_{16}H_{20}N_4NaO_2$ calcd: 323.1478, found: 323.1486. The compound is not reported in the literature.

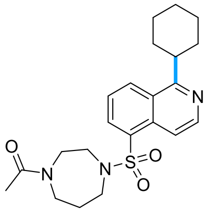

1-(4-((1-cyclohexylisoquinolin-5-yl)sulfonyl)-1,4-diazepan-1-yl)ethan-1-one (**51**), undefined ratio of conformational isomers. Following the general procedure A, with 4 equiv of TFA, the product was isolated by preparative TLC with Hex/EtOAc/MeOH (1:1:0.5) as the eluent to give a solid (25.3 mg, 61%). **^{1}H NMR** (500 MHz, $CDCl_3$) δ 8.64 – 8.62 (m, 1H), 8.50 – 8.48 (m, 1H), 8.32 – 8.28 (m, 1H), 8.21 (d, *J* = 6.1 Hz, 1H),

7.67 – 7.63 (m, 1H), 3.73 – 3.36 (m, 9H), 2.06 – 2.05 (m, 3H), 2.01 – 1.93 (m, 6H), 1.87 – 1.80 (m, 3H), 1.56 – 1.49 (m, 2H), 1.41 – 1.37 (m, 1H). **^{13}C NMR** (126 MHz, $CDCl_3$) δ 170.2, 170.0, 166.8, 166.8, 144.1, 144.0, 134.7, 132.5, 132.4, 132.2, 130.6, 130.6, 126.9, 125.2, 115.2, 115.2, 50.9, 50.1, 49.2, 48.3, 45.0, 47.6, 46.8, 44.4, 42.1, 42.1, 32.7, 28.9, 27.6, 26.8, 26.1, 21.6, 21.1. **HRMS** (M+H$^+$) for $C_{22}H_{30}N_3O_3S$ calcd: 416.2002, found: 416.1994. The spectra data is consistent with the reported literature.[21]

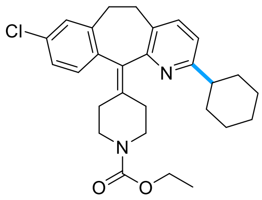

Ethyl 4-(8-chloro-2-cyclohexyl-5,6-dihydro-11*H*-benzo[5,6]cyclohepta[1,2-*b*]pyridin-11-ylidene)piperidine-1-carboxylate (**52**). Following the general procedure A, with 4 equiv of TFA, the product was isolated by preparative TLC with Hex/EtOAc (5:1) as the eluent to give a solid (30.6 mg, 66%). **^{1}H NMR** (500 MHz, $CDCl_3$) δ 7.34 (d, *J* = 8.0 Hz, 1H), 7.18 – 7.12 (m, 3H), 6.95 (d, *J* = 7.9 Hz, 1H), 4.16 – 4.12 (m, 2H), 3.83 (br, 2H), 3.38 – 3.34 (m, 1H), 3.30 – 3.24 (m, 1H), 3.12 – 3.07 (m, 2H), 2.84 – 2.74 (m, 2H), 2.71 – 2.66 (m, 1H), 2.49 (br, 1H), 2.35 – 2.31 (m, 3H), 1.96 – 1.93 (m, 1H), 1.90 – 1.87 (m, 1H), 1.83 – 1.81 (m, 2H), 1.75 – 1.72 (m, 1H), 1.48 – 1.35 (m, 4H), 1.25 (t, *J* = 7.0 Hz, 4H). **^{13}C NMR** (126 MHz, $CDCl_3$) 163.6, 155.7, 155.5, 134.0, 138.2, 138.0, 137.3, 134.6, 132.7, 130.5, 130.2, 128.8, 126.0, 118.8, 61.3, 46.1, 44.9, 33.9, 32.4, 31.8, 31.4, 31.0, 30.6, 29.7, 26.6, 26.4, 26.1, 14.7. **HRMS** (M+H$^+$) for $C_{28}H_{34}ClN_2O_2$ calcd: 465.2303, found: 465.2304. The spectra data is consistent with the reported literature.[22]

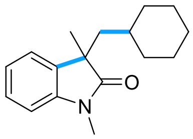

((*R*)-3-(cyclohexylmethyl)-1,3-dimethylindolin-2-one (**53**). The product was isolated by preparative TLC with Hex/EtOAc (5:1) as the eluent to give an oil. **^{1}H NMR** (500 MHz, $CDCl_3$) δ 7.28 – 7.24 (m, 1H), 7.16 (d, *J* = 6.2 Hz, 1H), 7.07 – 7.04 (m, 1H), 6.84 (d, *J* = 7.7 Hz, 1H), 3.22 (s, 3H), 1.92 (dd, *J* = 14.1, 6.9 Hz, 1H), 1.72 (dd, *J* = 14.1, 5.3 Hz, 1H), 1.52 – 1.45 (m, 3H), 1.36 – 1.31 (m, 4H), 1.22 – 1.19 (m, 1H), 0.99 – 0.91 (m, 4H), 0.86 – 0.73 (m, 2H). **^{13}C NMR** (126 MHz, $CDCl_3$) δ 181.2, 143.1, 134.4, 127.5, 122.7, 122.3, 108.0, 47.9, 45.4, 34.7, 34.5, 33.5, 26.2, 26.2, 26.1 (X2), 26.0. **GC-MS** (EI, m/z) for $C_{17}H_{23}NO$ calcd: 257.2, found: 257.1. The spectra data is consistent with the reported literature.[23]

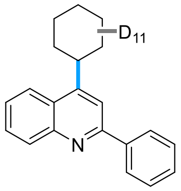

4-(cyclohexyl-*d*$_{11}$)-2-phenylquinoline (**3-d$_{11}$**) The product was isolated by preparative TLC with Hex/EtOAc (10:1) as the eluent to give a solid. **^{1}H NMR** (500 MHz, $CDCl_3$) δ 8.19 (d, *J* = 9.7 Hz, 1H), 8.15 – 8.14 (m, 2H), 8.09 (d, *J* = 9.8 Hz, 1H), 7.75 (s, 1H), 7.72 – 7.68 (m, 1H), 7.55 – 7.51 (m, 3H), 7.47 – 7.44 (m, 1H).**^{13}C NMR** (126 MHz, $CDCl_3$) δ 157.4, 154.0, 148.6, 140.3, 130.7, 129.1, 129.0, 128.8 (X2), 127.6 (X2), 125.9, 125.9, 122.9, 115.5. **^{2}H NMR** (77 MHz, $CDCl_3$) δ 3.28, 1.98, 1.85, 1.53, 1.47, 1.26. **HRMS** (M+H$^+$) for $C_{21}H_{11}D_{11}N$ calcd: 299.2436, found: 299.2437. The spectra data is consistent with the reported literature.[7]

Supplementary References

1. Tan, L.; Su, H.; Han, J.; Liu, M.; Li, C.-J. Selective conversion of methane to cyclohexane and hydrogen via efficient hydrogen transfer catalyzed by GaN supported platinum clusters. *Sci. Rep.* **12**, 18414 (2022).
2. Wang, Z.; Liu, Q.; Ji, X.; Deng, G.-J.; Huang, H. Bromide-promoted visible-light-induced reductive Minisci reaction with aldehydes. *ACS Cat.* **10**, 154-159 (2020).
3. Sang, L.; Zhu, Q. S.; Yang, S. Y.; Liu, G. P.; Li, H. J.; Wei, H. Y.; Jiao, C. M.; Liu, S. M.; Wang, Z. G.; Zhou, X. W.; et al. Band offsets of non-polar A-plane GaN/AlN and AlN/GaN heterostructures measured by X-ray photoemission spectroscopy. *Nanoscale Res. Lett.* **9**, 470 (2014).
4. Gan, L. T.; Zhang, Y.; Liu, P. F.; Yang, H. G. Enhanced surface kinetics and charge transfer of $BiVO_4$ photoanodes by Rh_2O_3 cocatalyst loading for improved solar water oxidation. *Chem. Asian J.* **17**, e202101359 (2022).
5. Li, L.; Mu, X.; Liu, W.; Kong, X.; Fan, S.; Mi, Z.; Li, C.-J. Thermal non-oxidative aromatization of light alkanes catalyzed by gallium nitride. *Angew. Chem. Int. Ed.* **53**, 14106-14109 (2014).
6. Zhao, H.; Jin, J. Visible light-promoted aliphatic C–H arylation using selectfluor as a hydrogen atom transfer reagent. *Org. Lett.* **21**, 6179-6184 (2019).
7. Huang, C.-Y.; Li, J.; Li, C.-J. A cross-dehydrogenative C(sp^3)−H heteroarylation via photo-induced catalytic chlorine radical generation. *Nat. Commun.* **12**, 4010 (2021).
8. Xu, P.; Chen, P.-Y.; Xu, H.-C. Scalable photoelectrochemical dehydrogenative cross-coupling of heteroarenes with aliphatic C−H bonds. *Angew. Chem. Int. Ed.* **59**, 14275-14280 (2020).
9. McCallum, T.; Pitre, S. P.; Morin, M.; Scaiano, J. C.; Barriault, L. The photochemical alkylation and reduction of heteroarenes. *Chem. Sci.* **8**, 7412-7418 (2017).
10. Ramaraju, A.; Chouhan, N. K.; Ravi, O.; Sridhar, B.; Bathula, S. R. Cu-catalyzed coupling of *O*-acyl oximes with isatins: domino rearrangement strategy for direct access to quinoline-4-carboxamides by C–N bond cleavage. *Eur. J. Org. Chem.* **23**, 2963-2971 (2018).
11. Ikarashi, G.; Morofuji, T.; Kano, N. Terminal-oxidant-free photocatalytic C–H alkylations of heteroarenes with alkylsilicates as alkyl radical precursors. *Chem. Commun.* **56**, 10006-10009 (2020).
12. Fu, M.-C.; Shang, R.; Zhao, B.; Wang, B.; Fu, Y. Photocatalytic decarboxylative alkylations mediated by triphenylphosphine and sodium iodide. *Science* **363**, 1429-1434 (2019).
13. Shao, X.; Wu, X.; Wu, S.; Zhu, C. Metal-free radical-mediated C(sp^3)–H heteroarylation of alkanes. *Org. Lett.* **22**, 7450-7454 (2020).
14. Chianelli, D.; Testaferri, L.; Tiecco, M.; Tingoli, M. Selective substitution of unprotonated phyridines by alkyl radicals. *Tetrahedron* **38**, 657-663 (1982).
15. Li, J.; Huang, C.-Y.; Han, J.-T.; Li, C.-J. Development of a quinolinium/cobaloxime dual photocatalytic system for oxidative C–C cross-couplings via H_2 release. *ACS Catal.* **11**, 14148-14158 (2021).
16. Huang, C.-Y.; Li, J.; Liu, W.; Li, C.-J. Diacetyl as a “traceless” visible light photosensitizer in metal-free cross-dehydrogenative coupling reactions. *Chem. Sci.* **10**, 5018-5024 (2019).
17. Zhang, L.; Liu, Z.-Q. Molecular oxygen-mediated Minisci-type radical alkylation of heteroarenes with boronic acids. *Org. Lett.* **19**, 6594-6597 (2017).
18. He, X.-K.; Lu, J.; Zhang, A.-J.; Zhang, Q.-Q.; Xu, G.-Y.; Xuan, J. BI-OAc-Accelerated C3–H alkylation of quinoxalin-2(1*H*)-ones under visible-light irradiation. *Org. Lett.* **22**, 5984-5989 (2020).
19. Antonchick, A. P.; Burgmann, L. Direct selective oxidative cross-coupling of simple alkanes with heteroarenes. *Angew. Chem. Int. Ed.* **52**, 3267-3271 (2013).
20. Tang, R.-J.; Kang, L.; Yang, L. Metal-free oxidative decarbonylative coupling of aliphatic aldehydes with azaarenes: successful Minisci-type alkylation of various heterocycles. *Adv. Synth. Catal.* **357**, 2055-2060 (2015).
21. Mondal, P. P.; Pal, A.; Prakash, A. K.; Sahoo, B. Ketone-derived 2,3-dihydroquinazolinones in *N*-heteroarene C–H alkylation via C–C bond scission under oxidative metal catalysis. *Chem. Commun.* **58**, 13202-13205 (2022).
22. Dong, J.; Lyu, X.; Wang, Z.; Wang, X.; Song, H.; Liu, Y.; Wang, Q. Visible-light-mediated Minisci C–H alkylation of heteroarenes with unactivated alkyl halides using O_2 as an oxidant. *Chem. Sci.* **10**, 976-982 (2019).
23. Ling, A.; Zhang, L.; Tan, R. X.; Liu, Z.-Q. Molecular oxygen-promoted general and site-specific alkylation with organoboronic Acid. *J. Org. Chem.* **83**, 14489-14497 (2018).
