# Supplementary material for: Acceptorless cross-dehydrogenative coupling for C(sp3)-H heteroarylation mediated by a heterogeneous GaN/ketone photocatalyst/photosensitizer system
Source: Commun Chem. 2023 Sep 1;6:181. doi: 10.1038/s42004-023-00947-w (PMC10474291; doi:10.1038/s42004-023-00947-w)
Supplement: Supplementary file 2 — Description of Additional Supplementary Files [file 42004_2023_947_MOESM2_ESM.pdf]

Description of Additional Supplementary Files

File name: Supplementary Data 1

Description: Dataset related to EPR spectra shown in the article

File name: Supplementary Data 2

Description: Dataset related to the heterogeneous catalyst recycling test shown in the article

File name: Supplementary Data 3

Description: NMR data
