# Supplementary material for: Acceptorless cross-dehydrogenative coupling for C(sp3)-H heteroarylation mediated by a heterogeneous GaN/ketone photocatalyst/photosensitizer system
Source: Commun Chem. 2023 Sep 1;6:181. doi: 10.1038/s42004-023-00947-w (PMC10474291; doi:10.1038/s42004-023-00947-w)

NMR spectra

Compound **3** ^{1}H-NMR

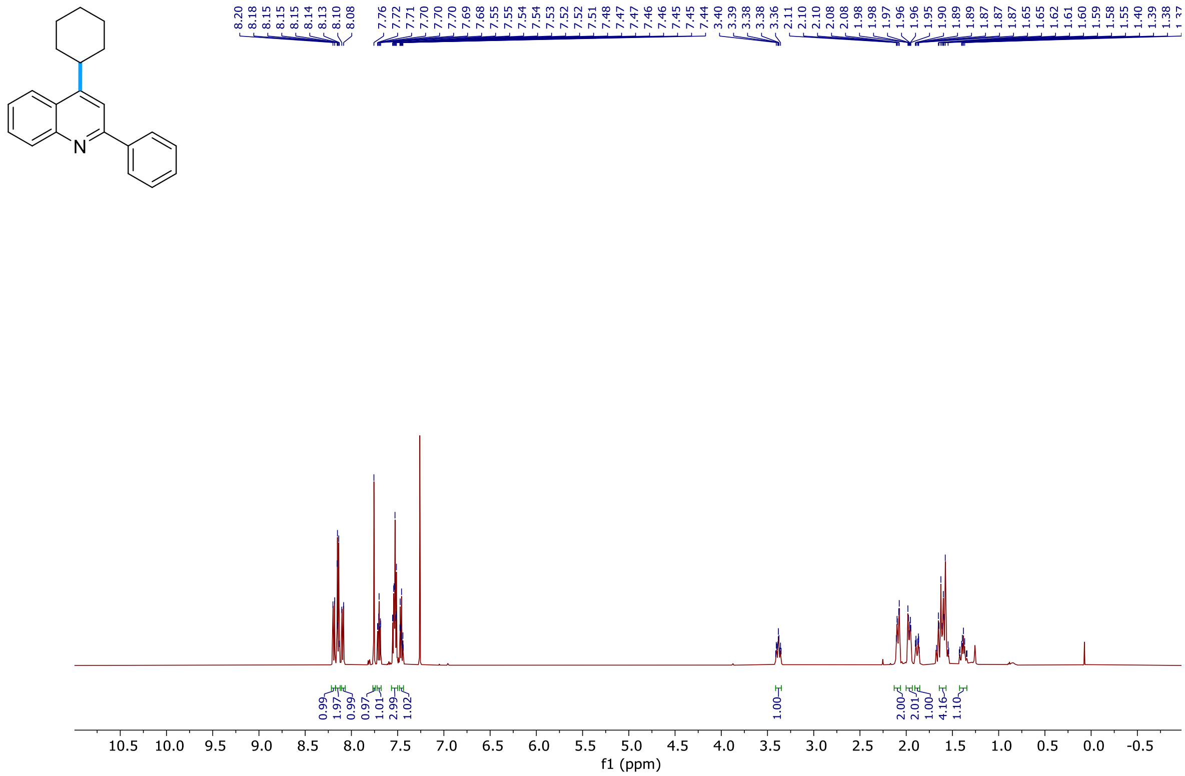

Compound **3** ^{13}C-NMR

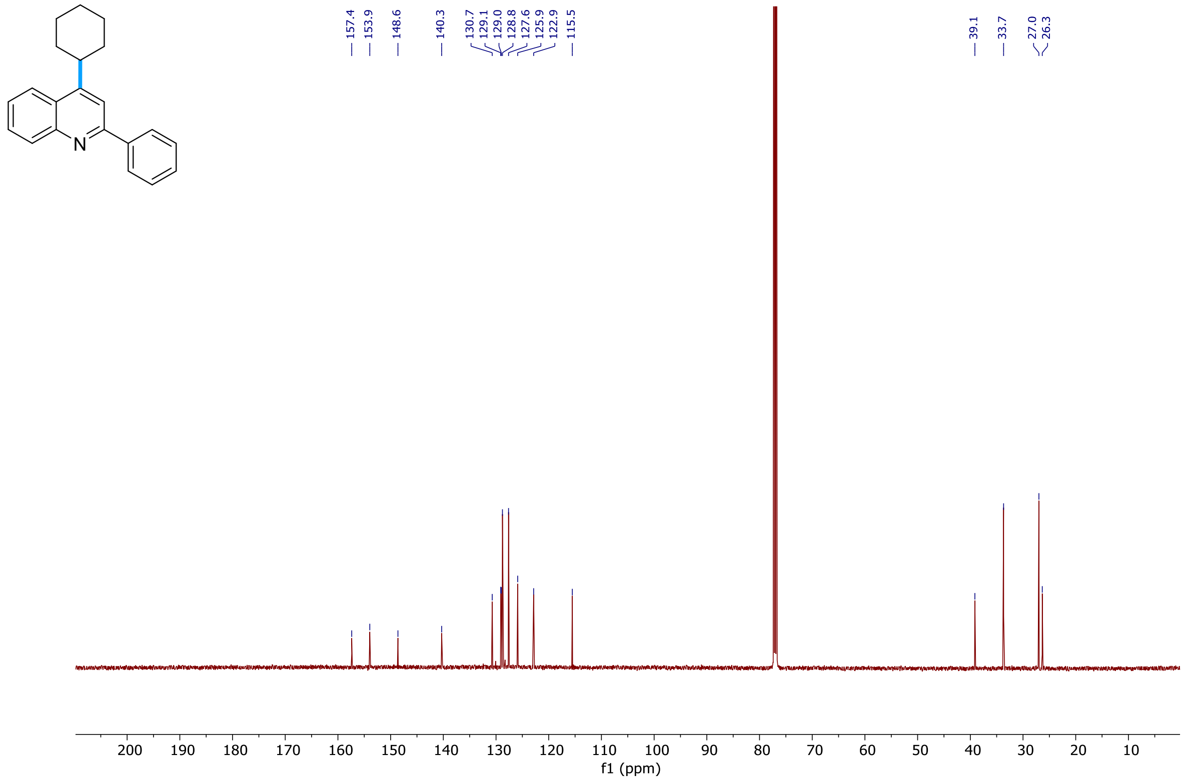

Compound **4** ^{1}H-NMR

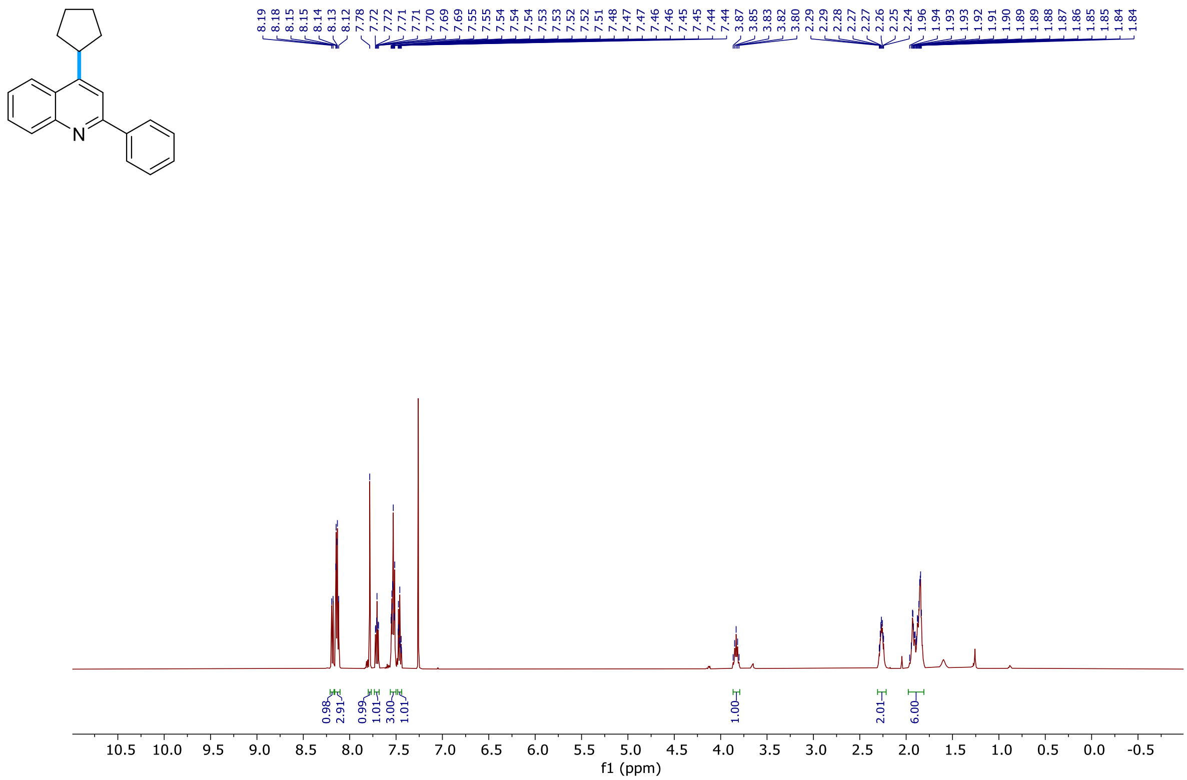

Compound **4** ^{13}C-NMR

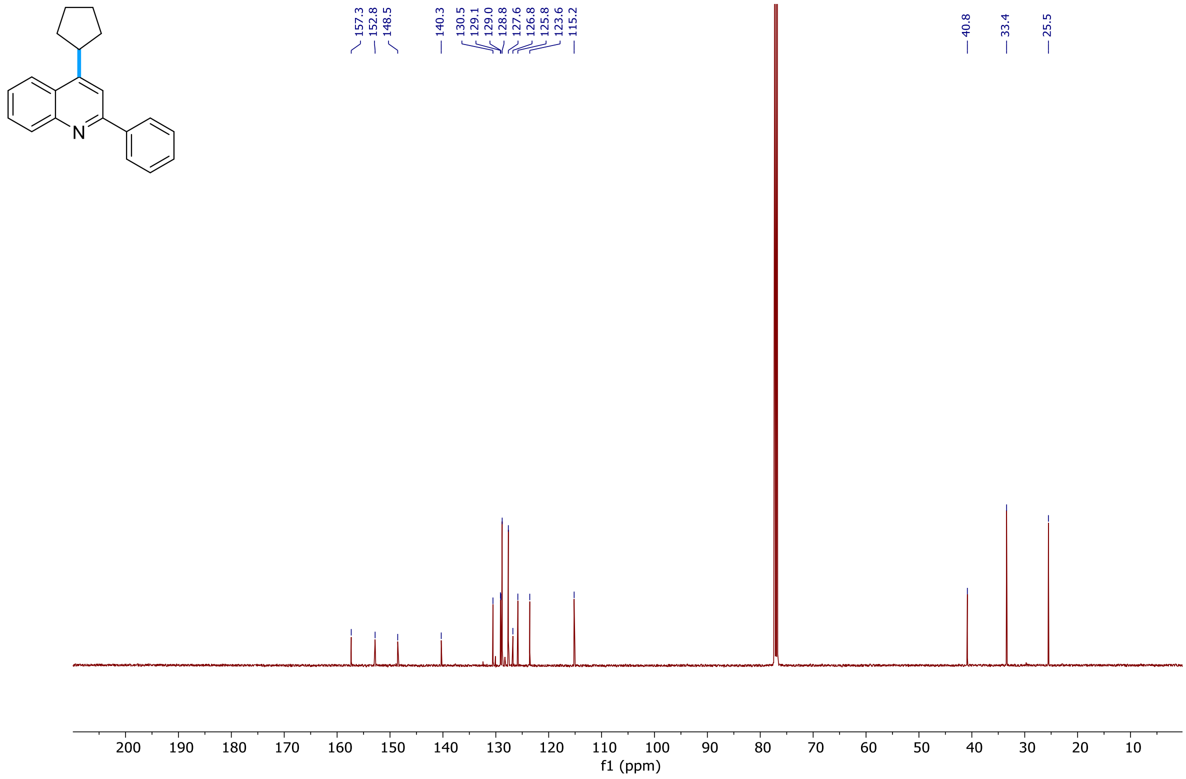

Compound **5** ^{1}H-NMR

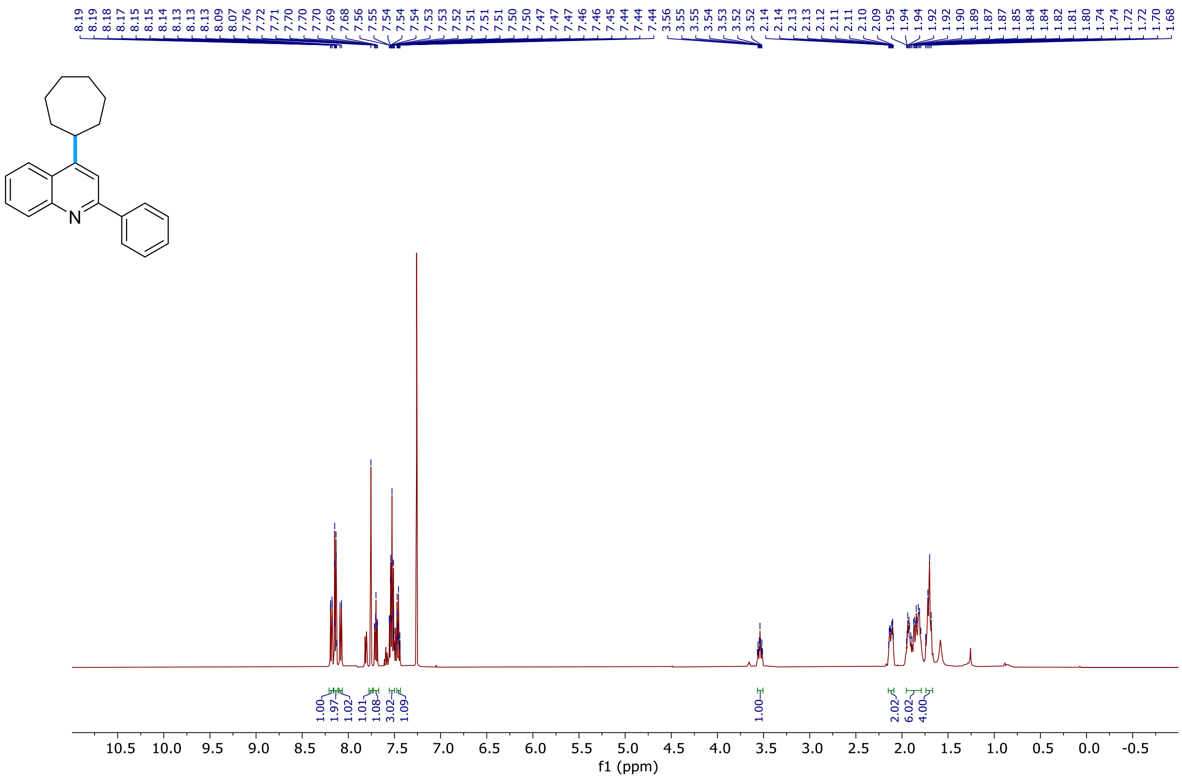

Compound **5** ^{13}C-NMR

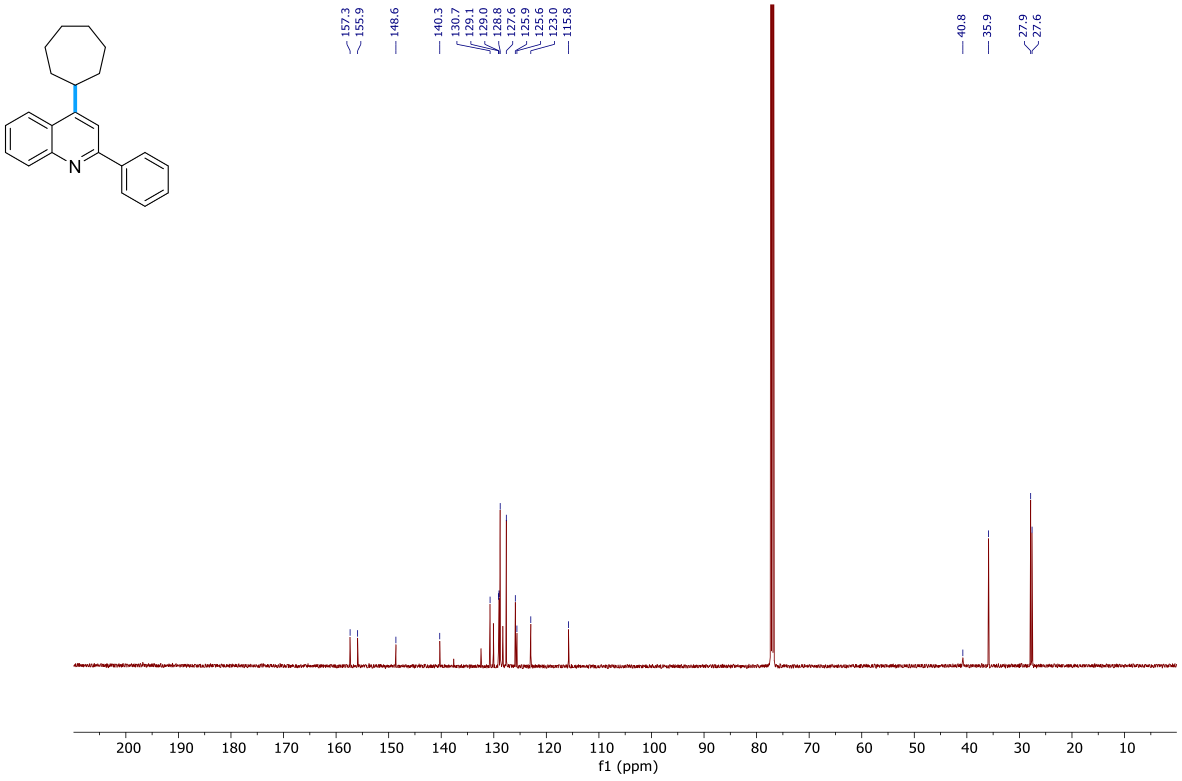

Compound **6** ^{1}H-NMR

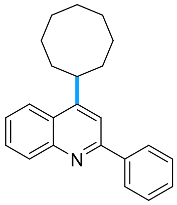

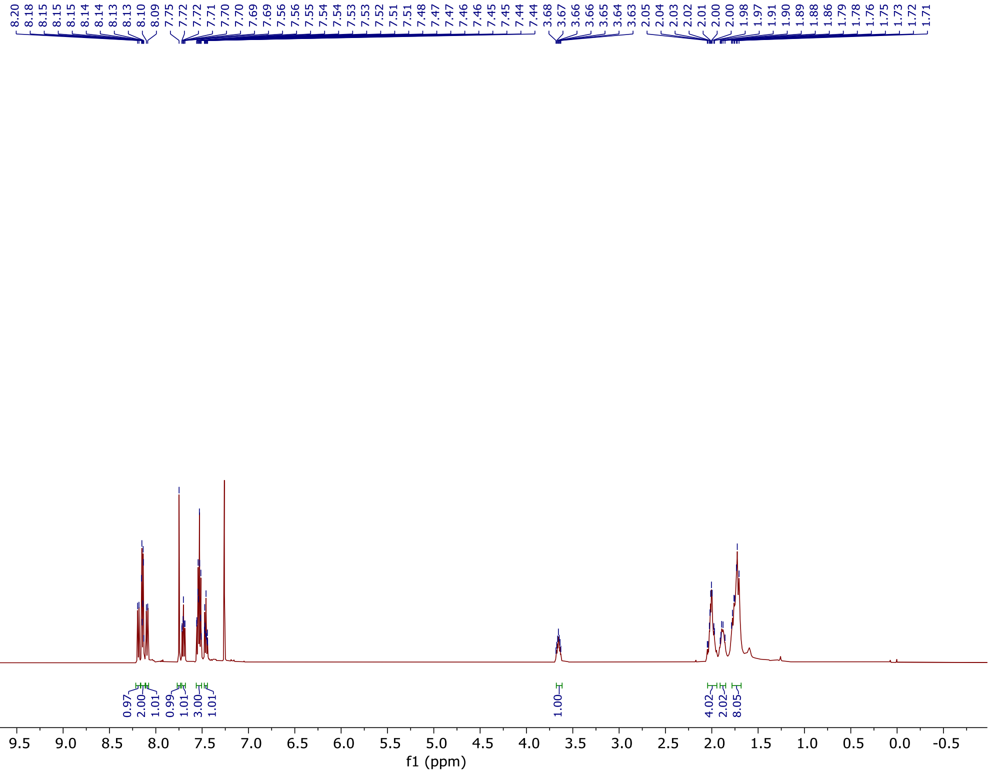

Compound **6** ^{13}C-NMR

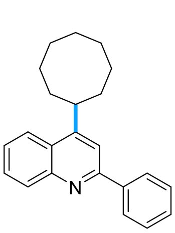

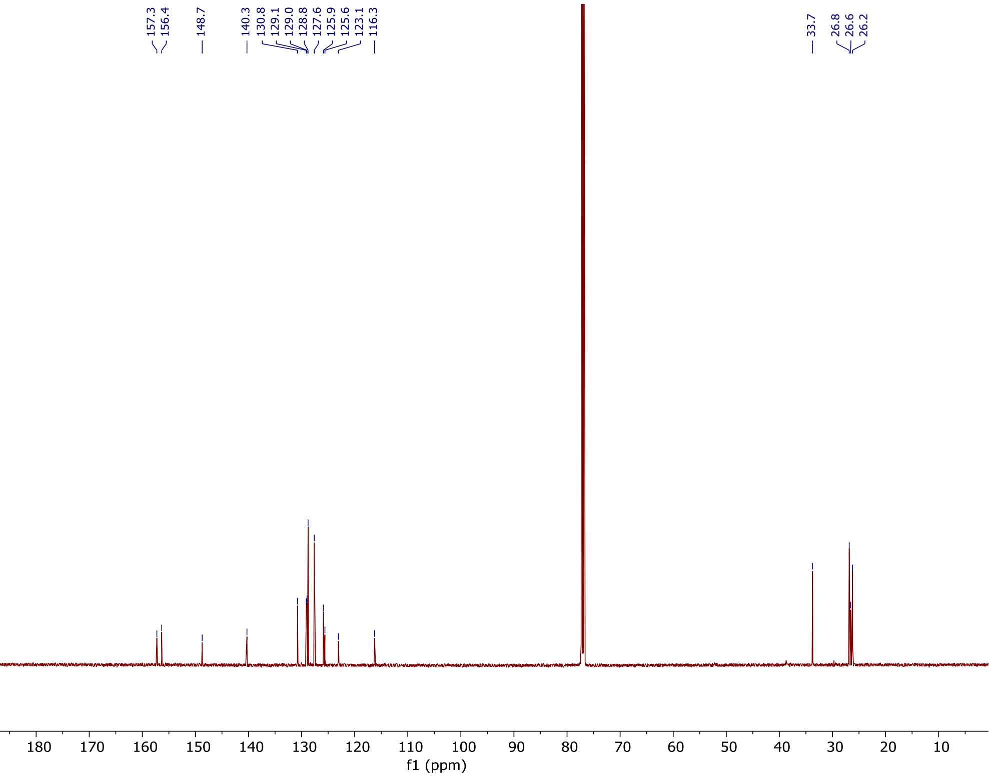

Compound **7** ¹H-NMR

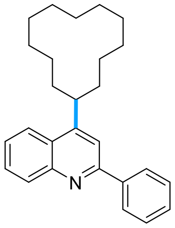

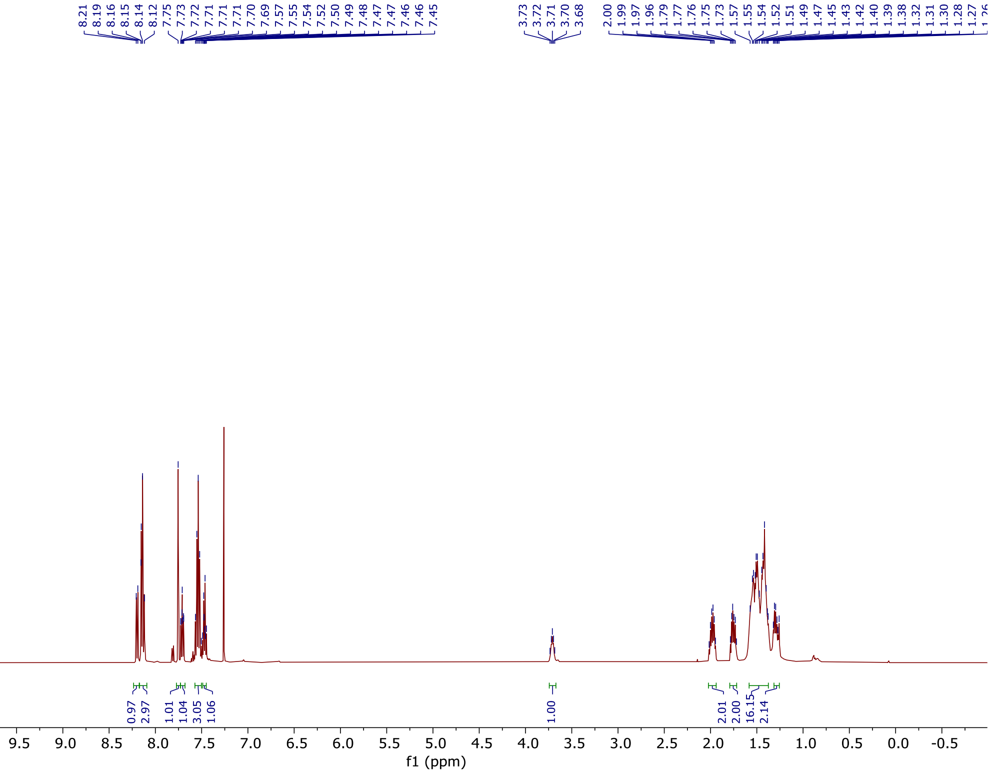

Compound **7** ¹³C-NMR

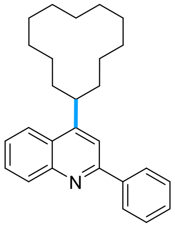

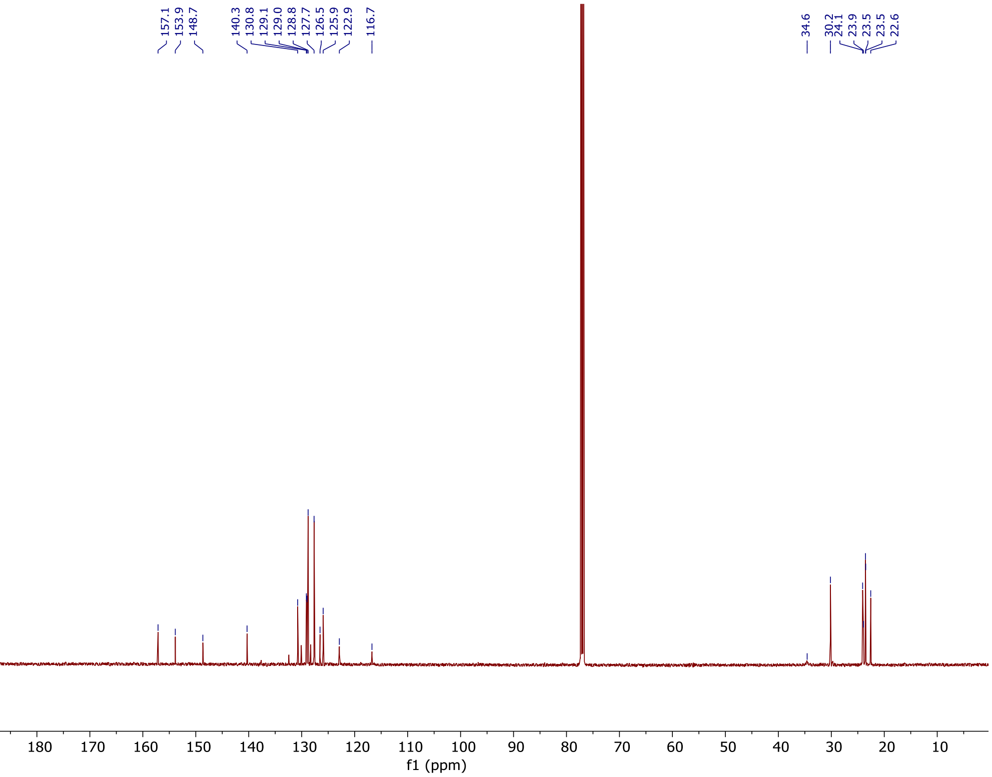

Compound **8** ¹H-NMR

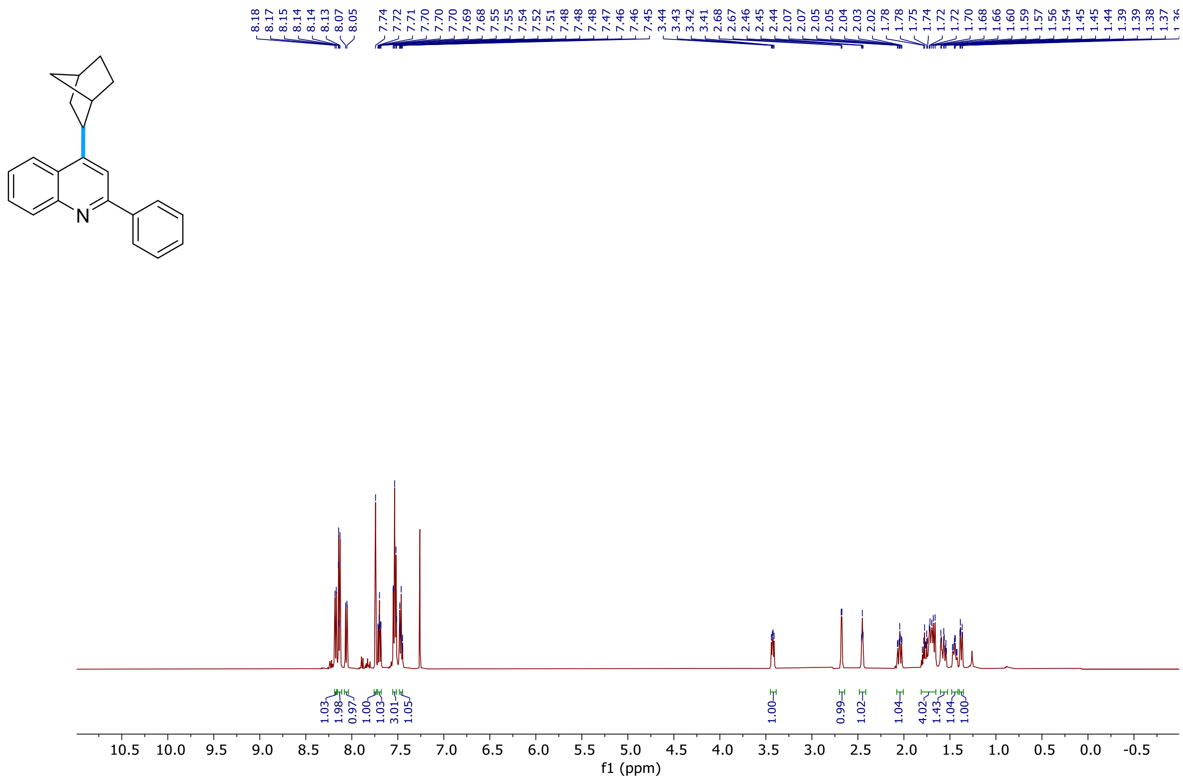

Compound **8** ¹³C-NMR

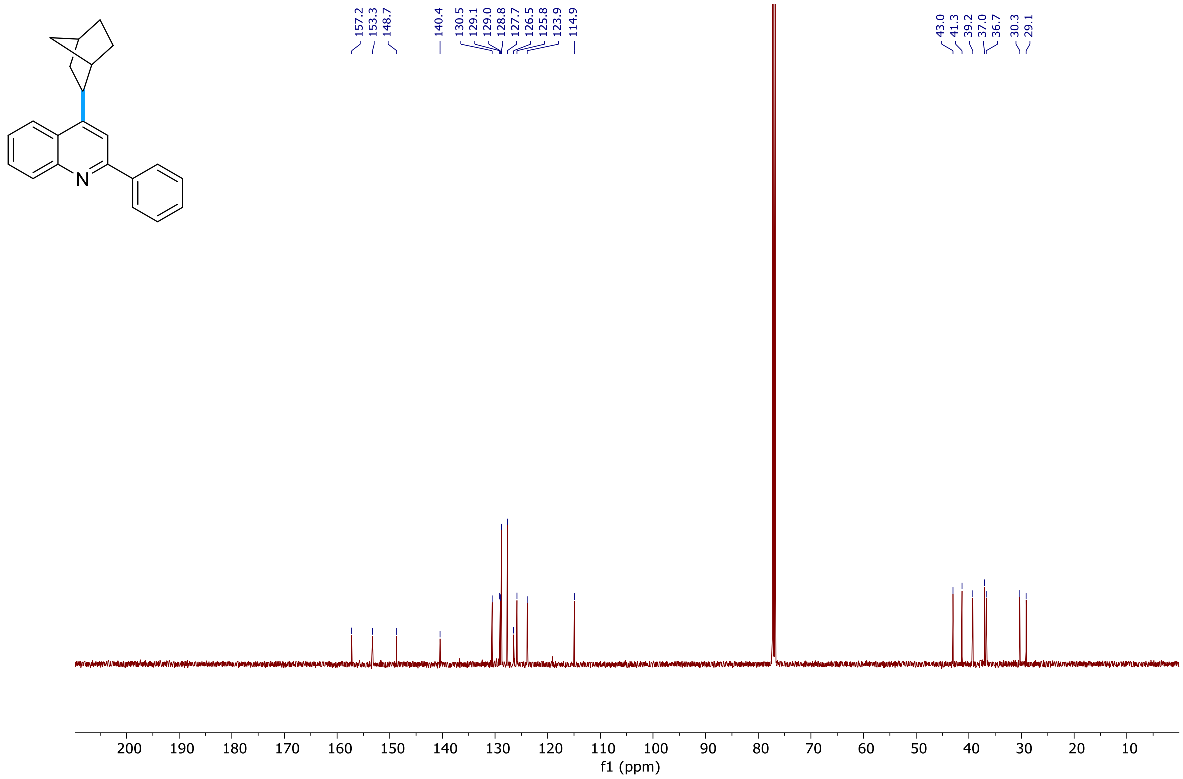

Compound **9** ^{1}H-NMR

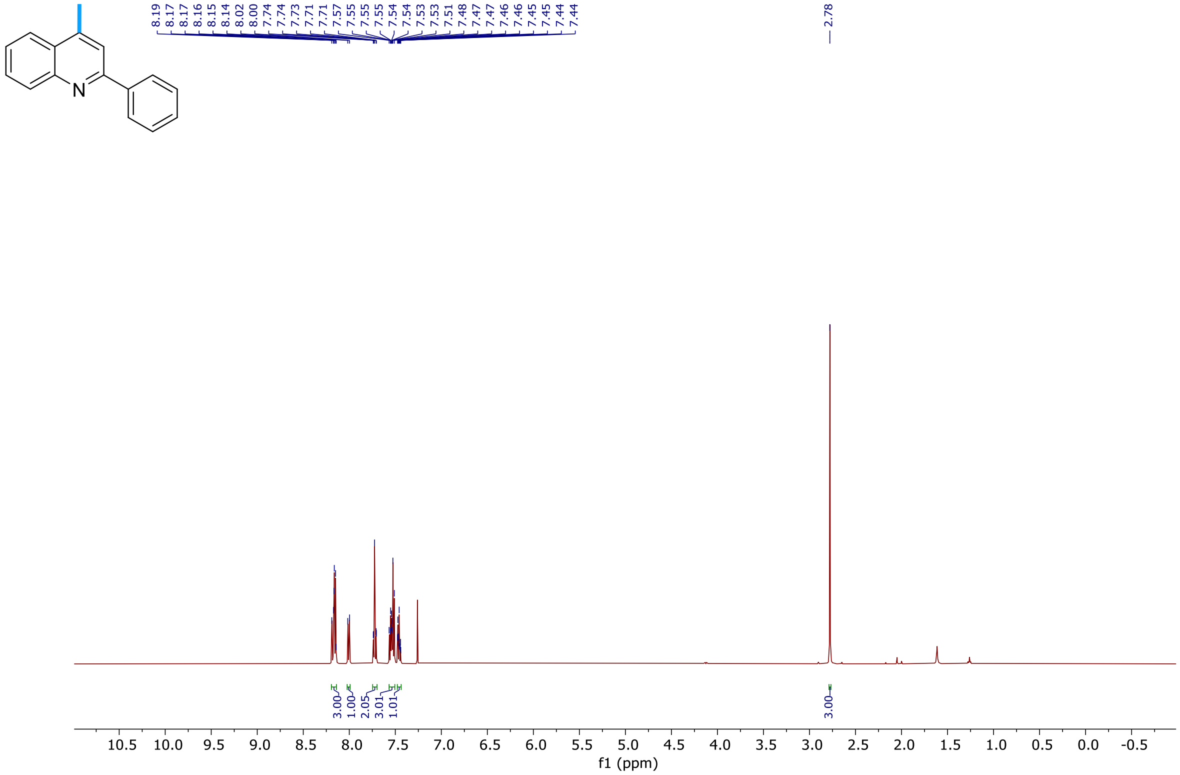

Compound **9** ^{13}C-NMR

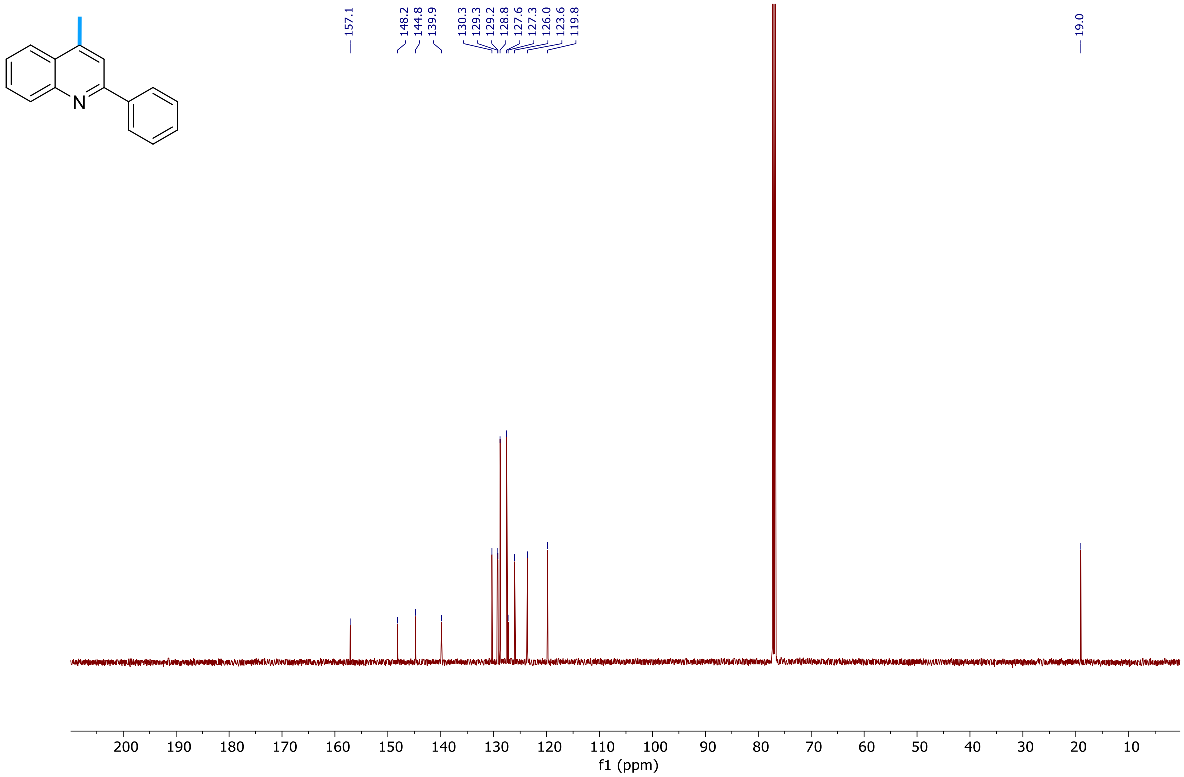

Compound **10** ^{1}H-NMR

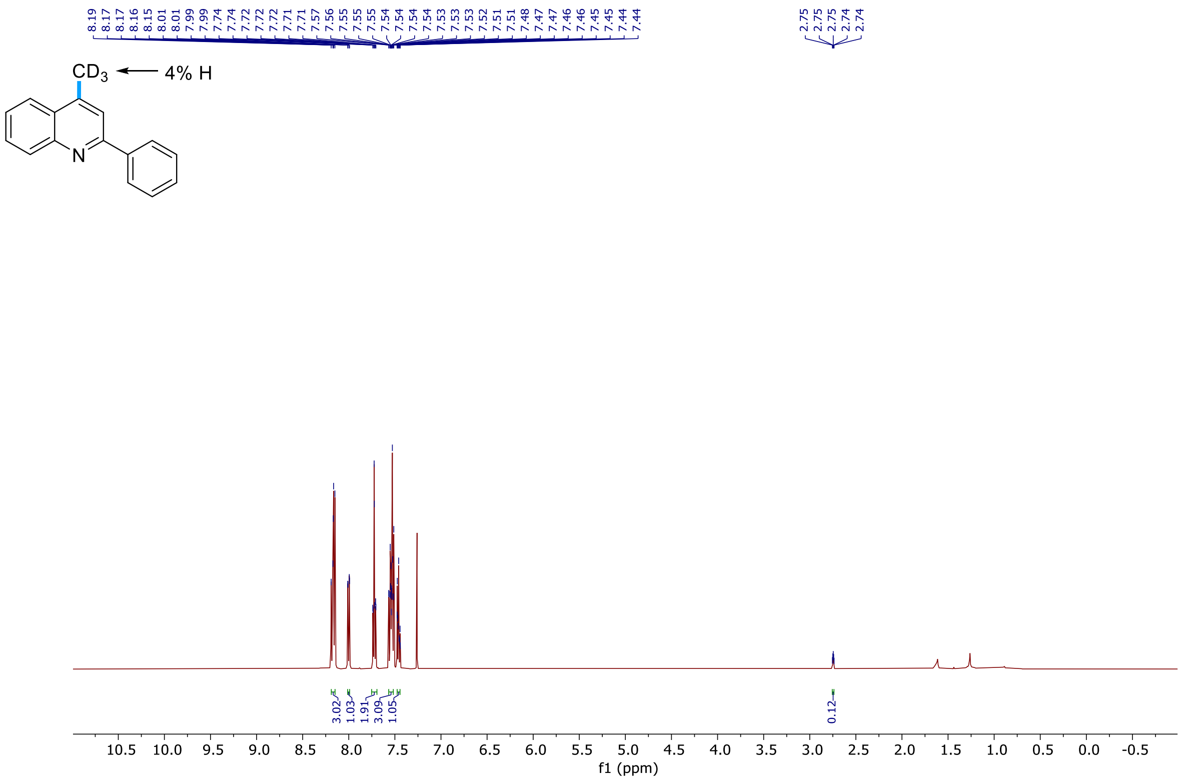

Compound **10** ^{13}C-NMR

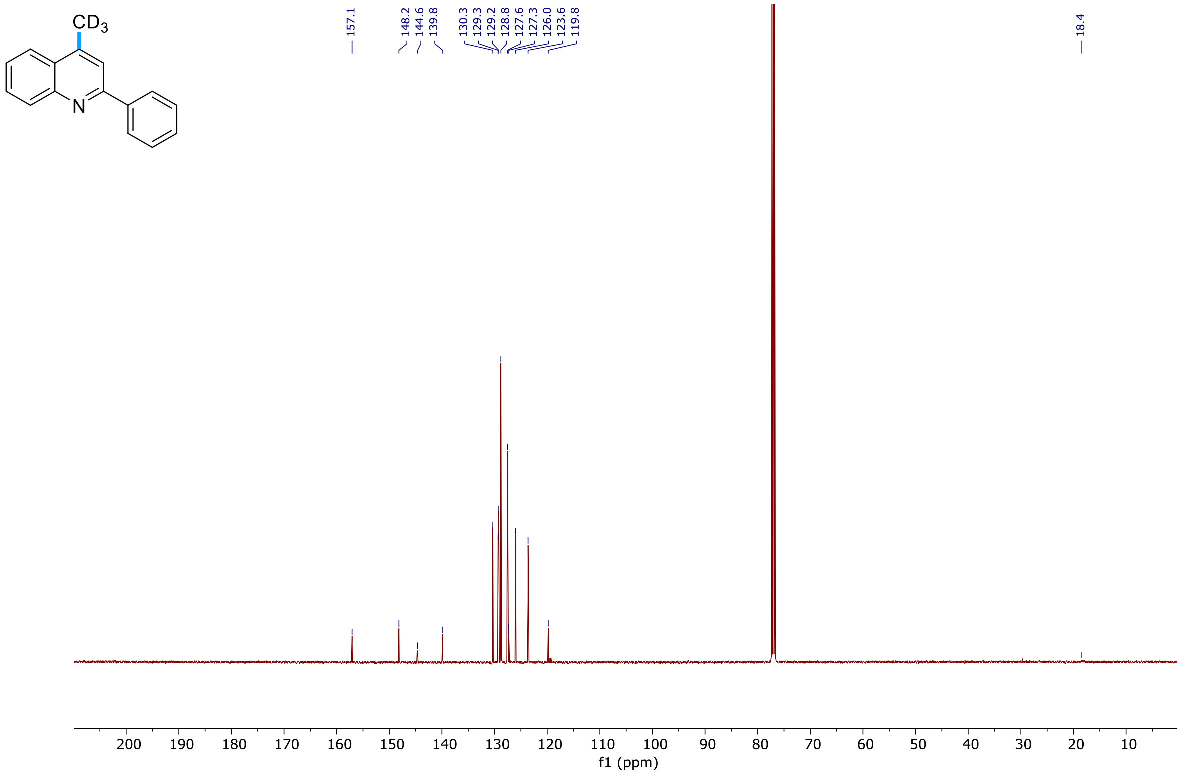

Compound **10** ^{2}H-NMR

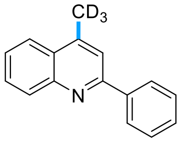

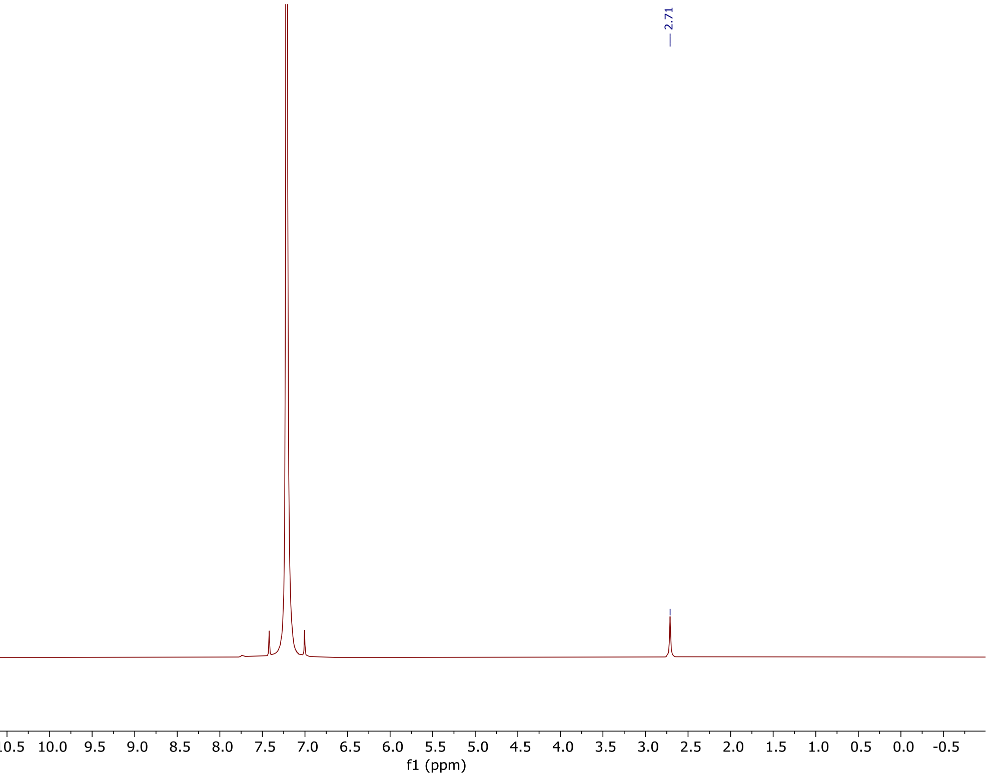

Compound **11** ^{1}H-NMR

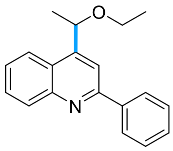

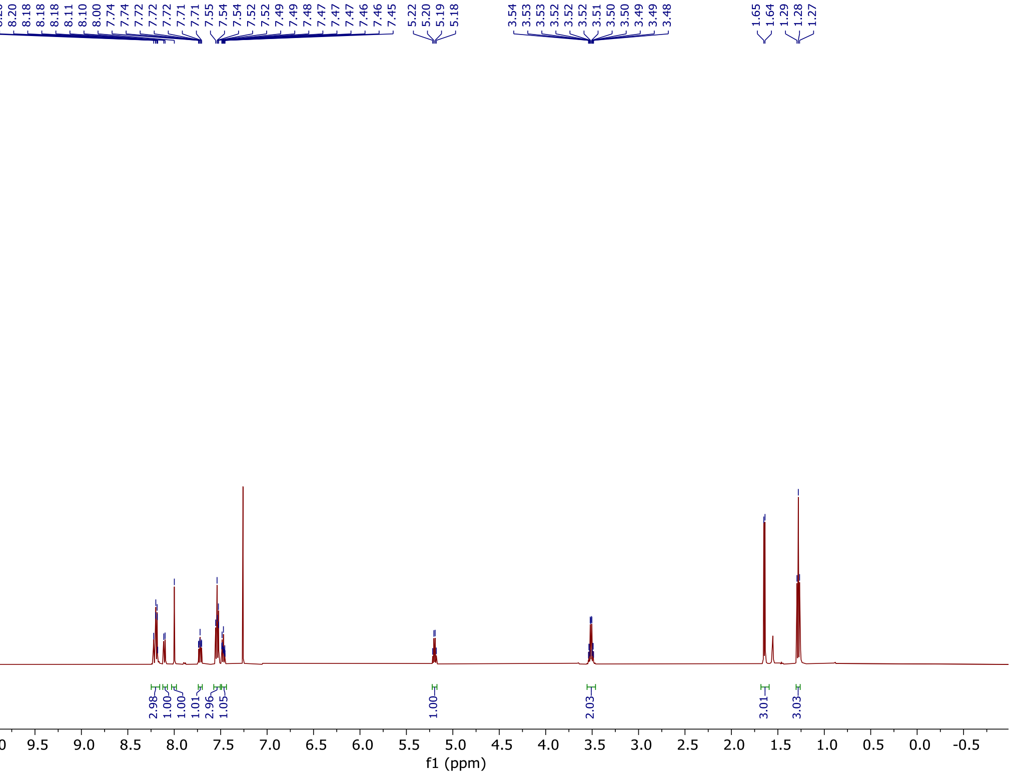

Compound **11** ^{13}C-NMR

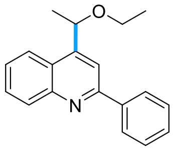

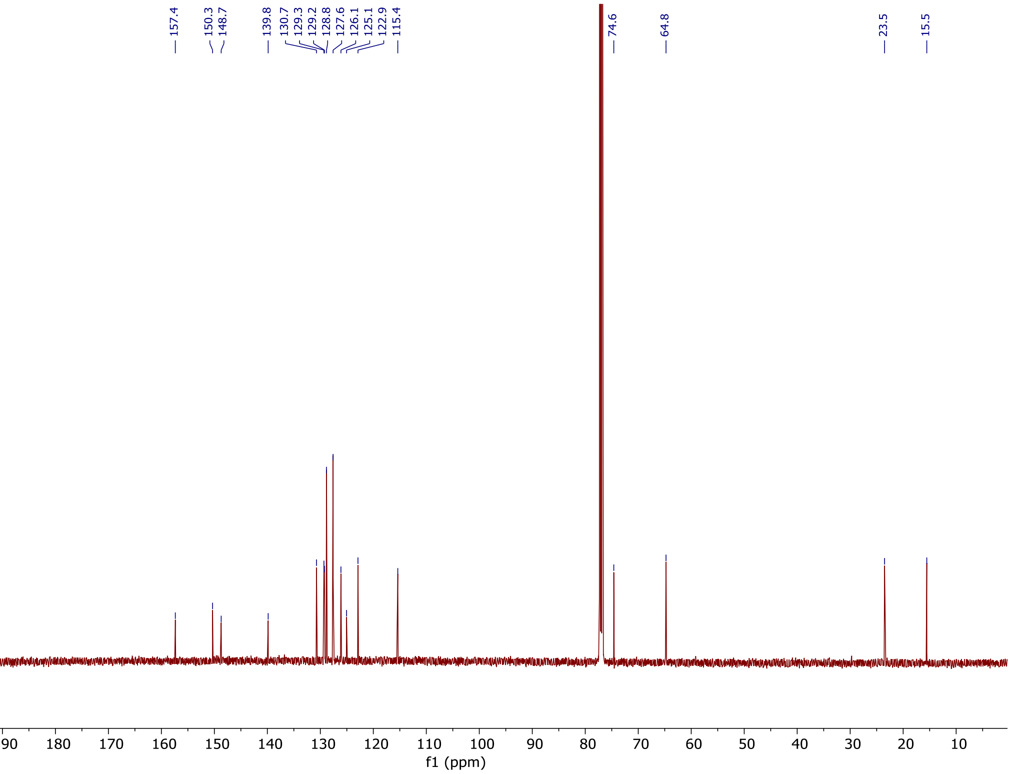

Compound **12** ^{1}H-NMR

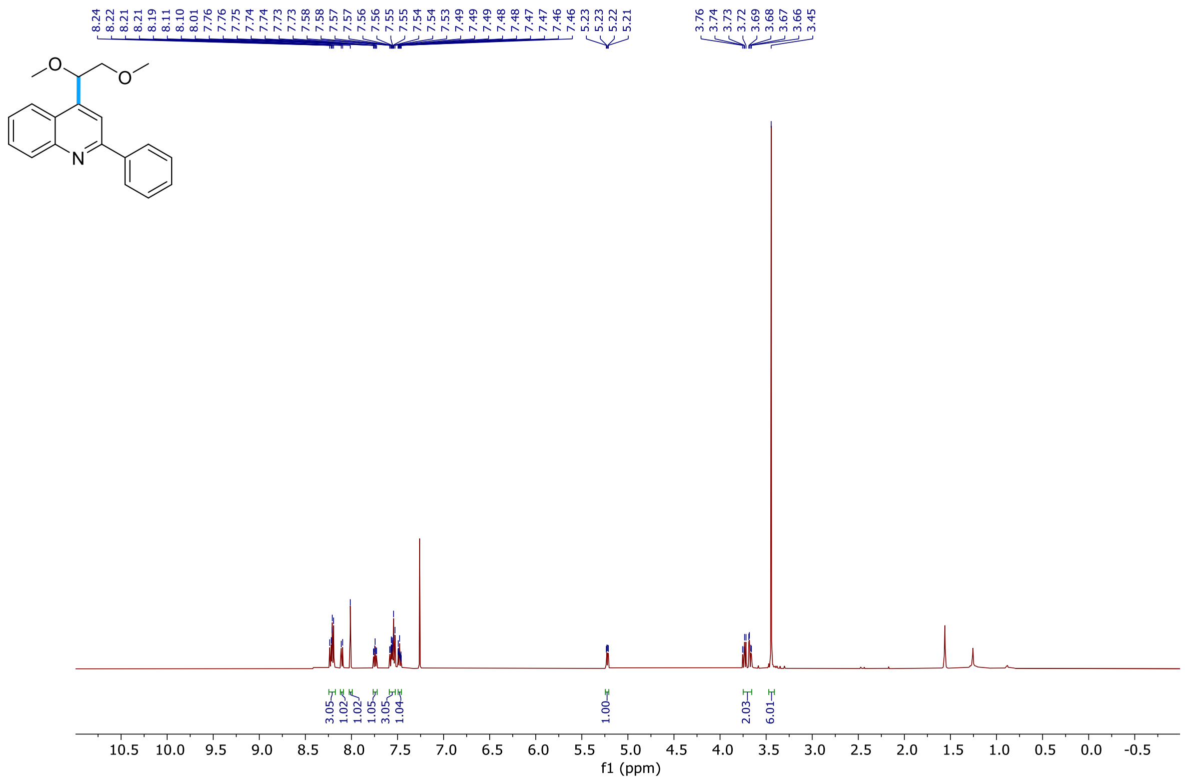

Compound **12** ^{13}C-NMR

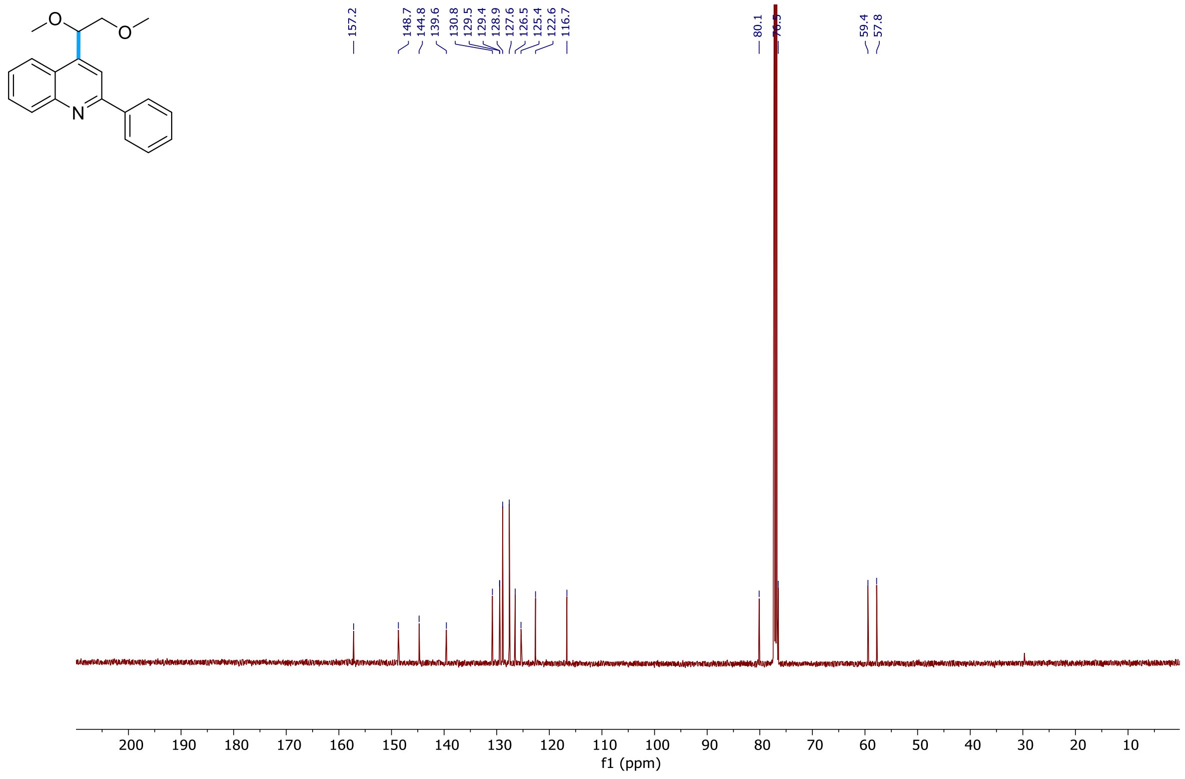

Compound **13** ^{1}H-NMR

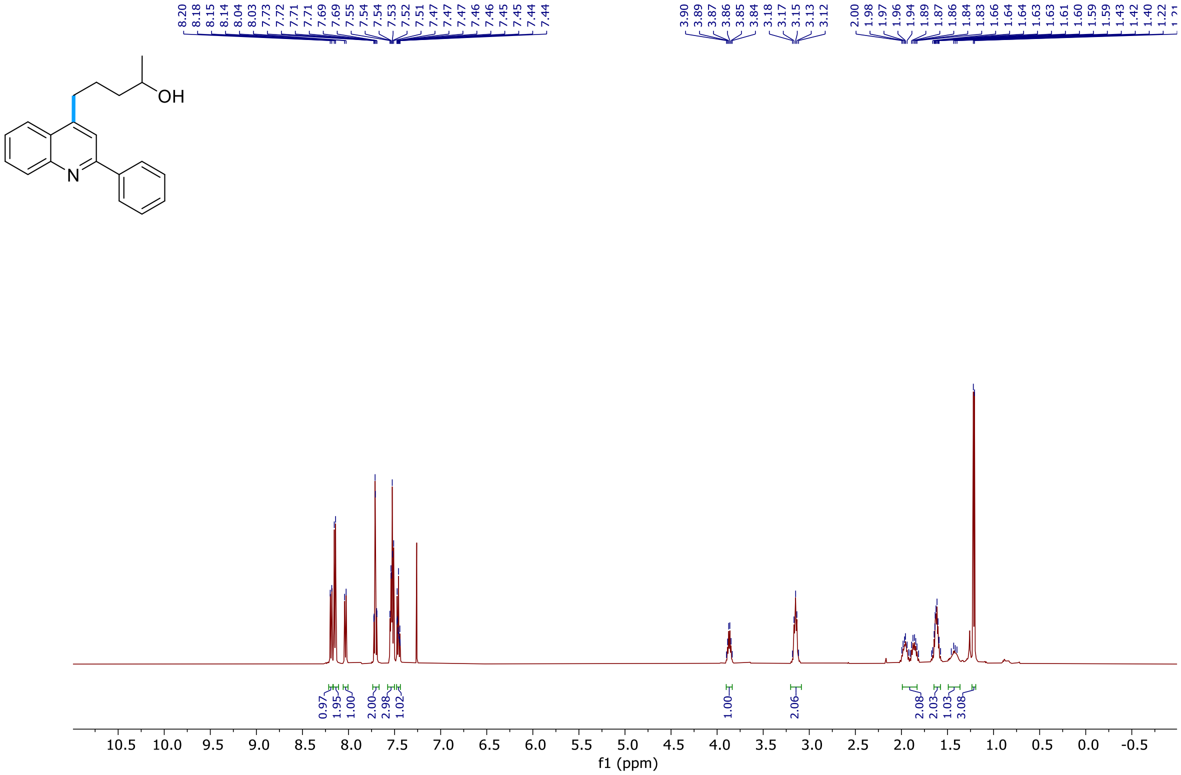

Compound **13** ^{13}C-NMR

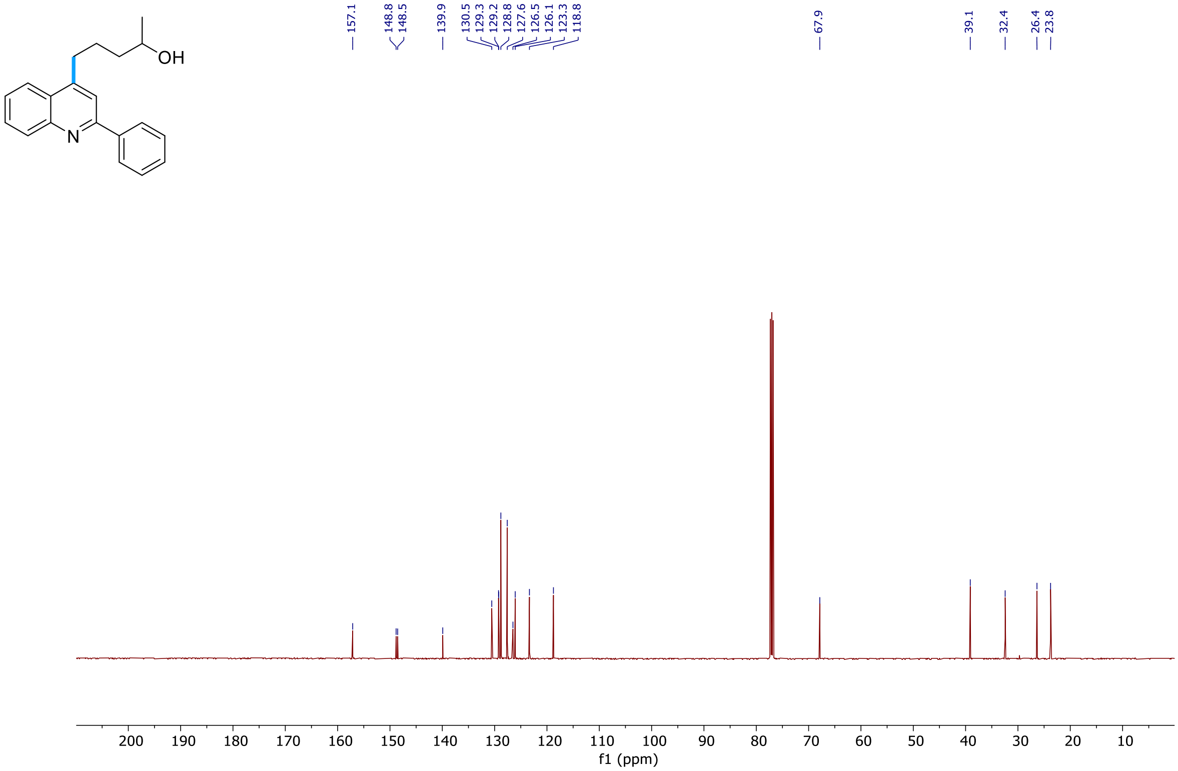

Compound **14** ^{1}H-NMR

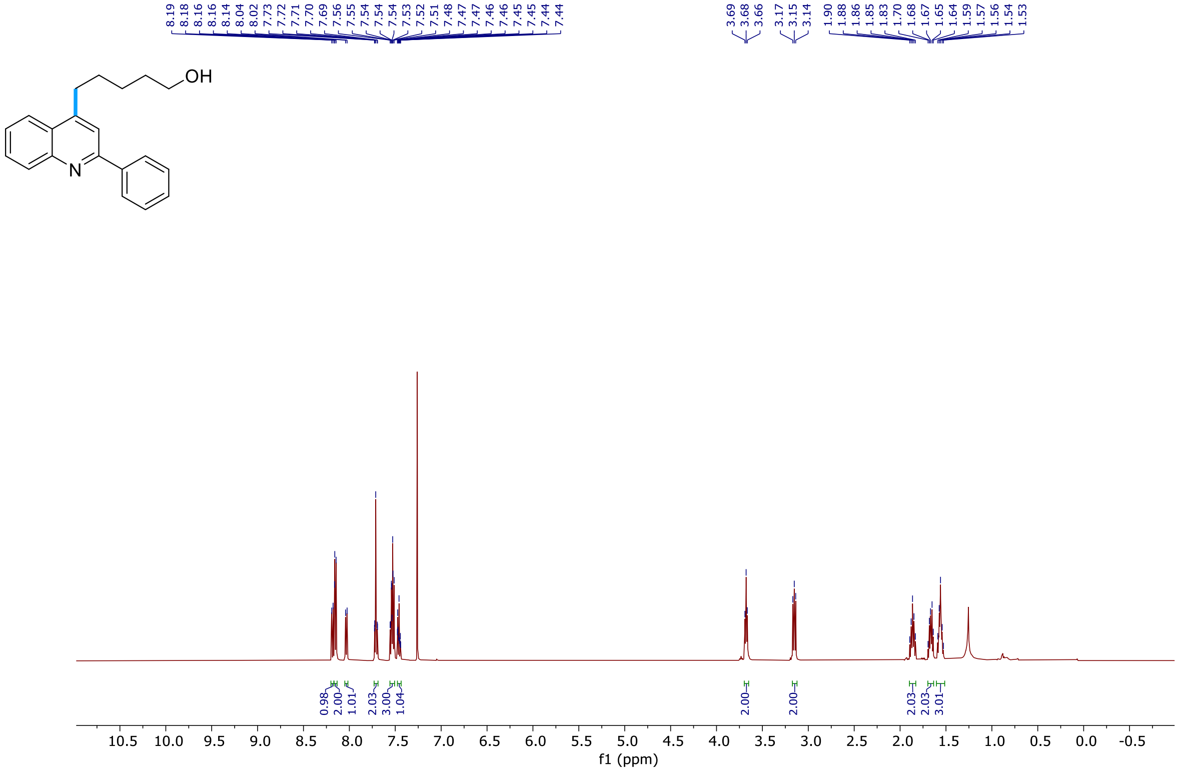

Compound **14** ^{13}C-NMR

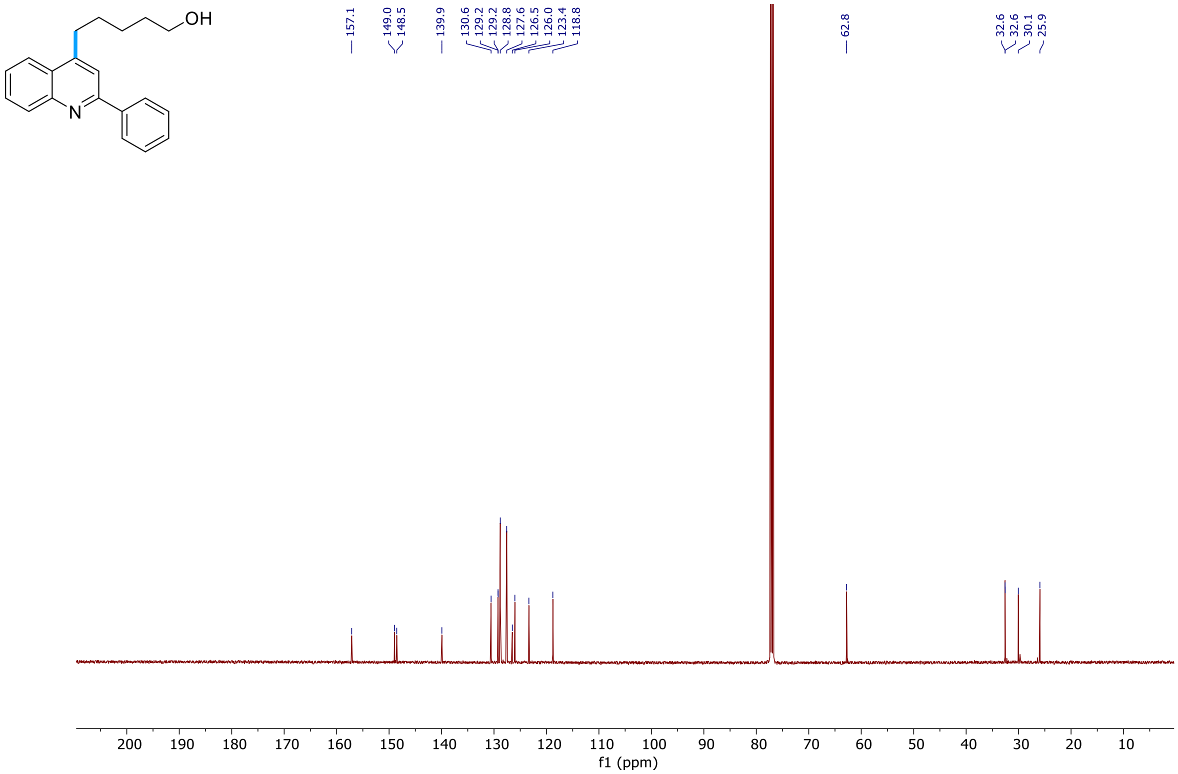

Compound **15** ^{1}H-NMR

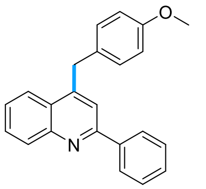

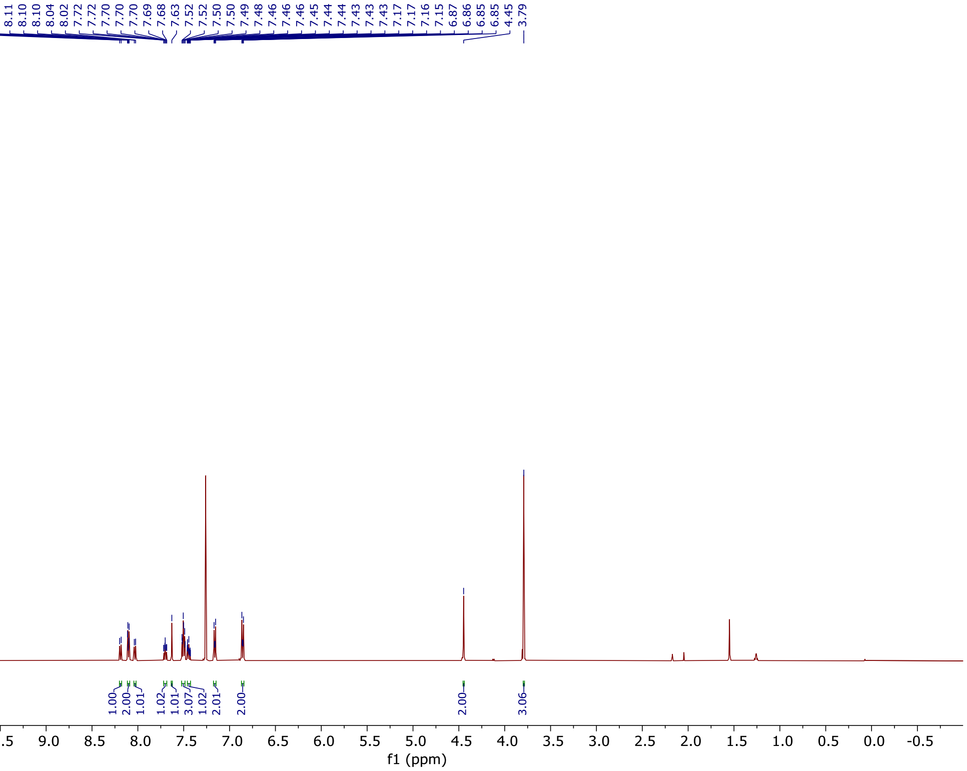

Compound **15** ^{13}C-NMR

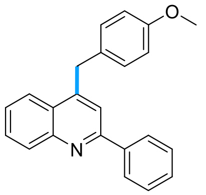

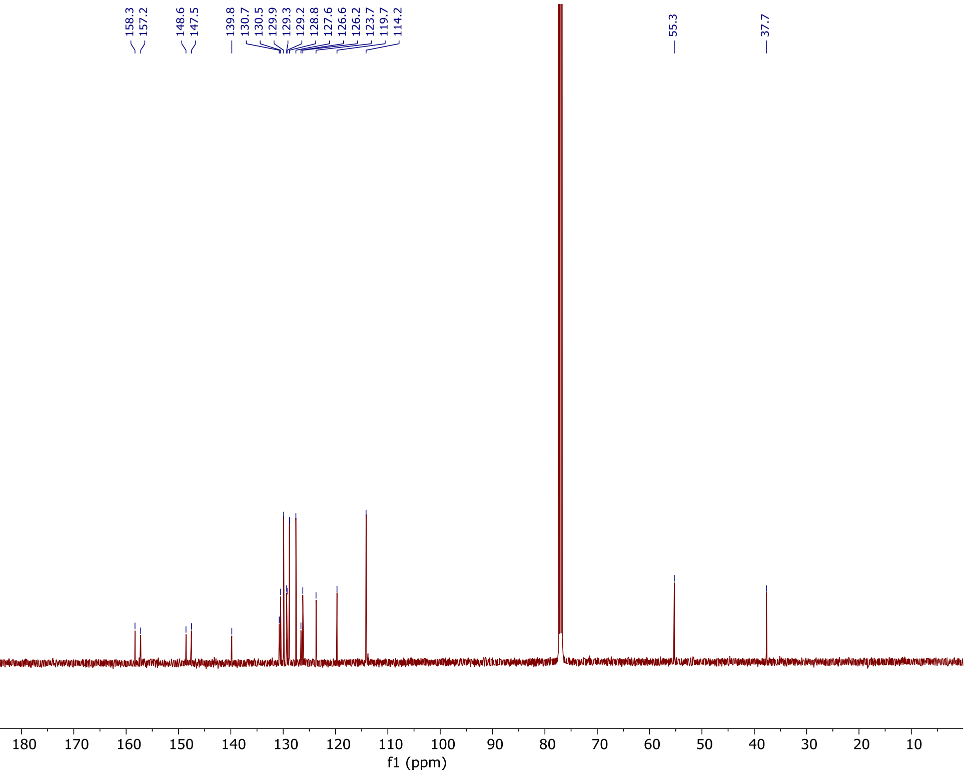

Compound **16** ^{1}H-NMR

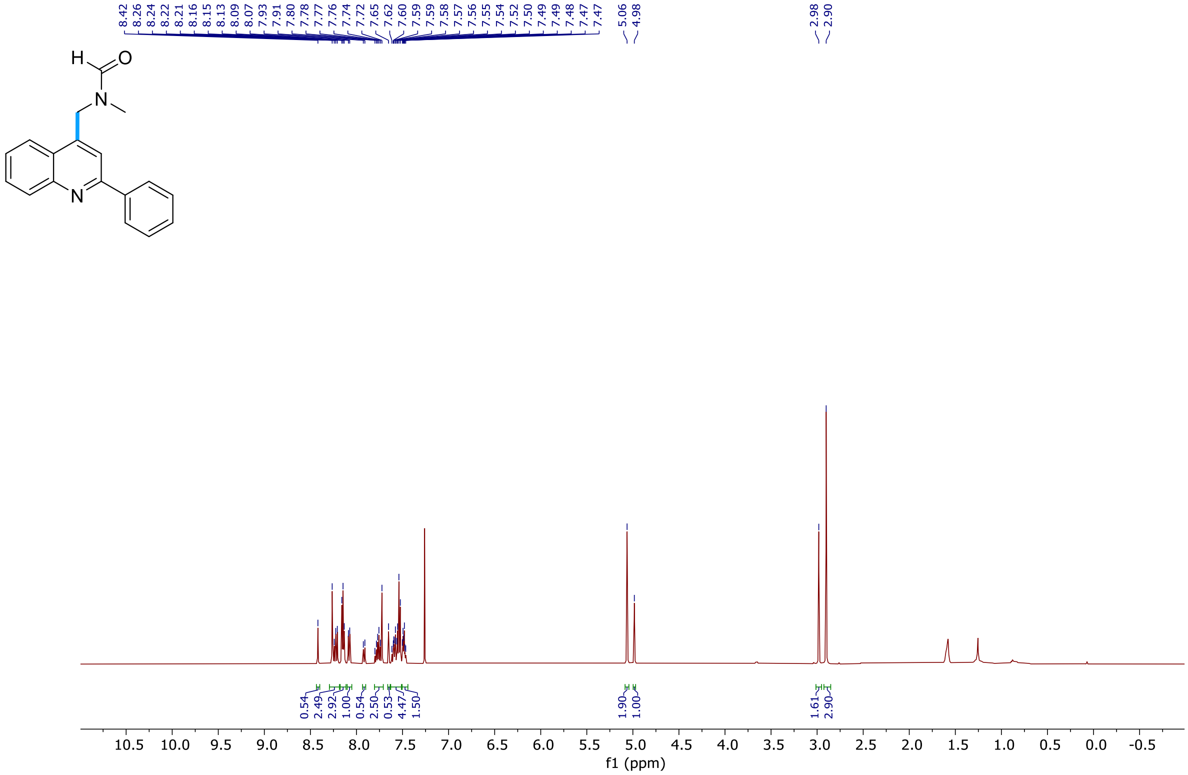

Compound **16** ^{13}C-NMR

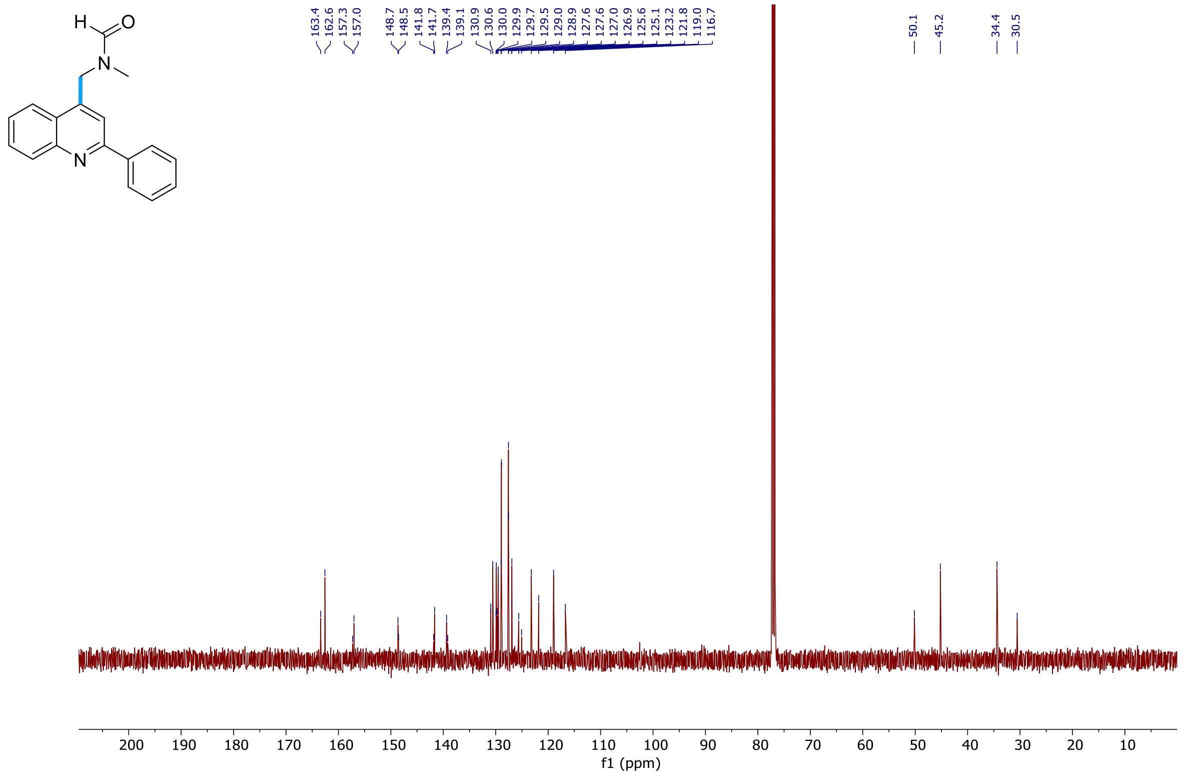

Compound **17** ^{1}H-NMR

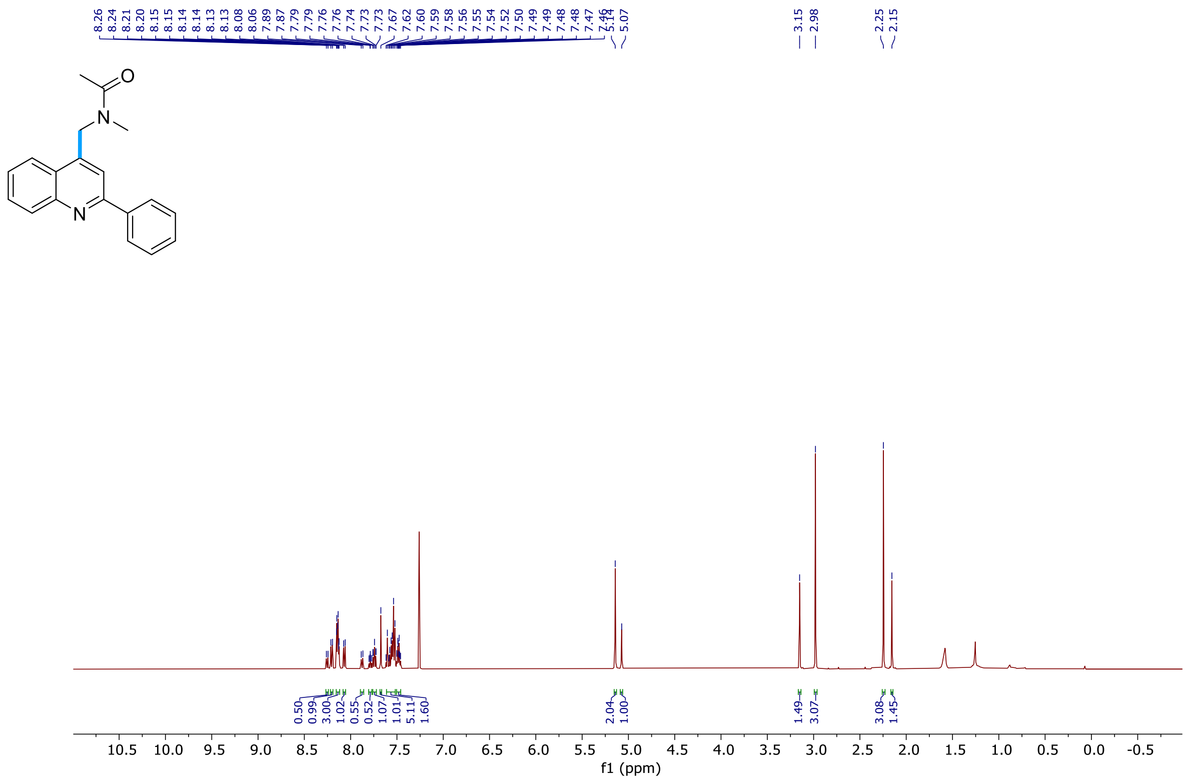

Compound **17** ^{13}C-NMR

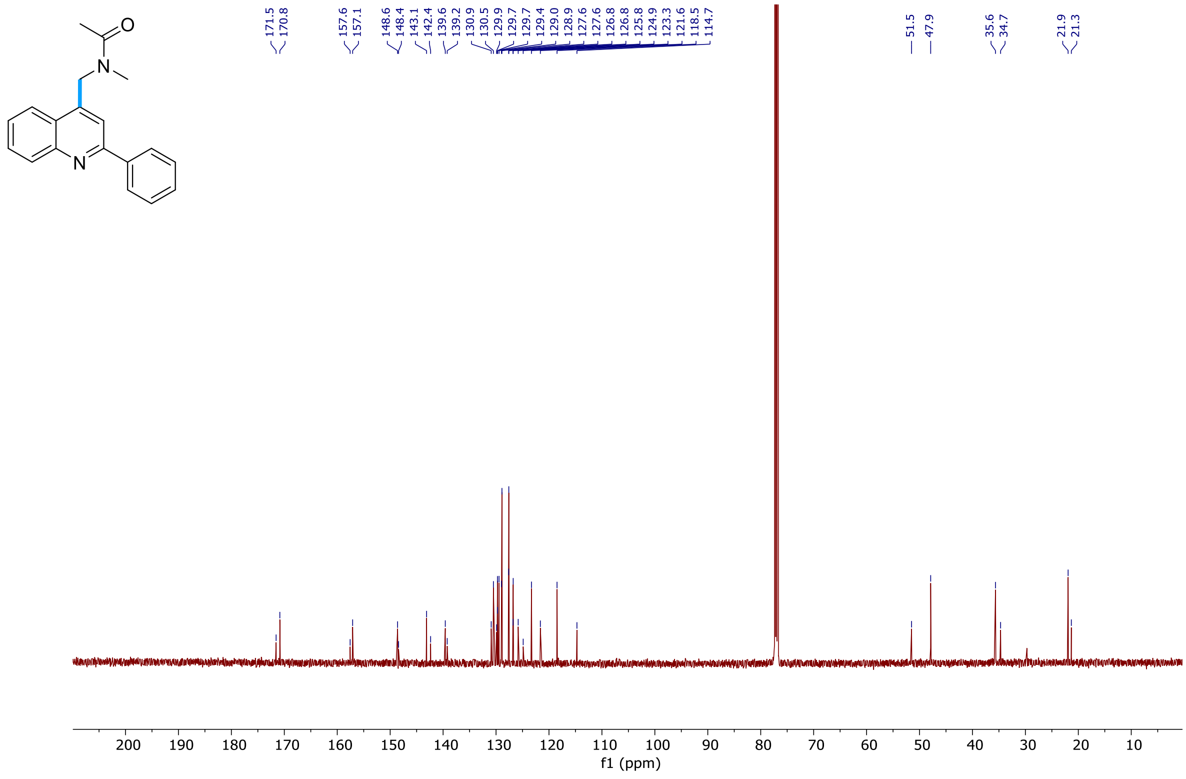

Compound **18** ^{1}H-NMR

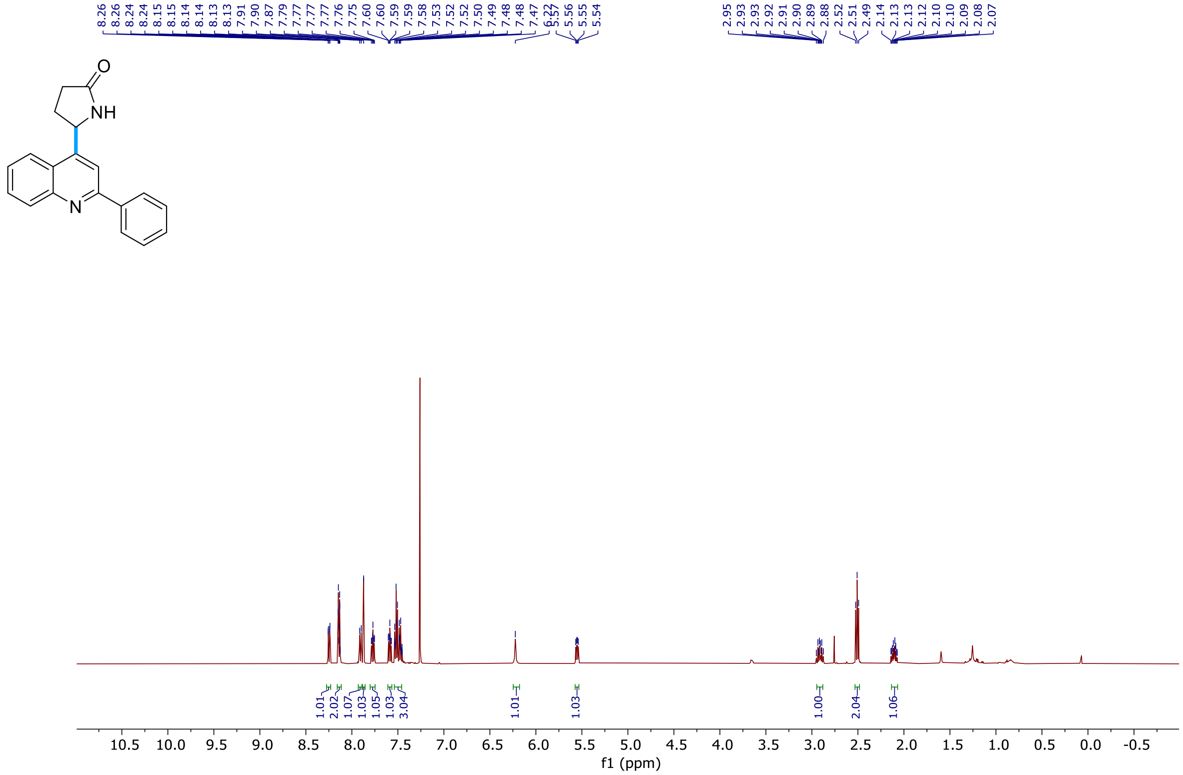

Compound **18** ^{13}C-NMR

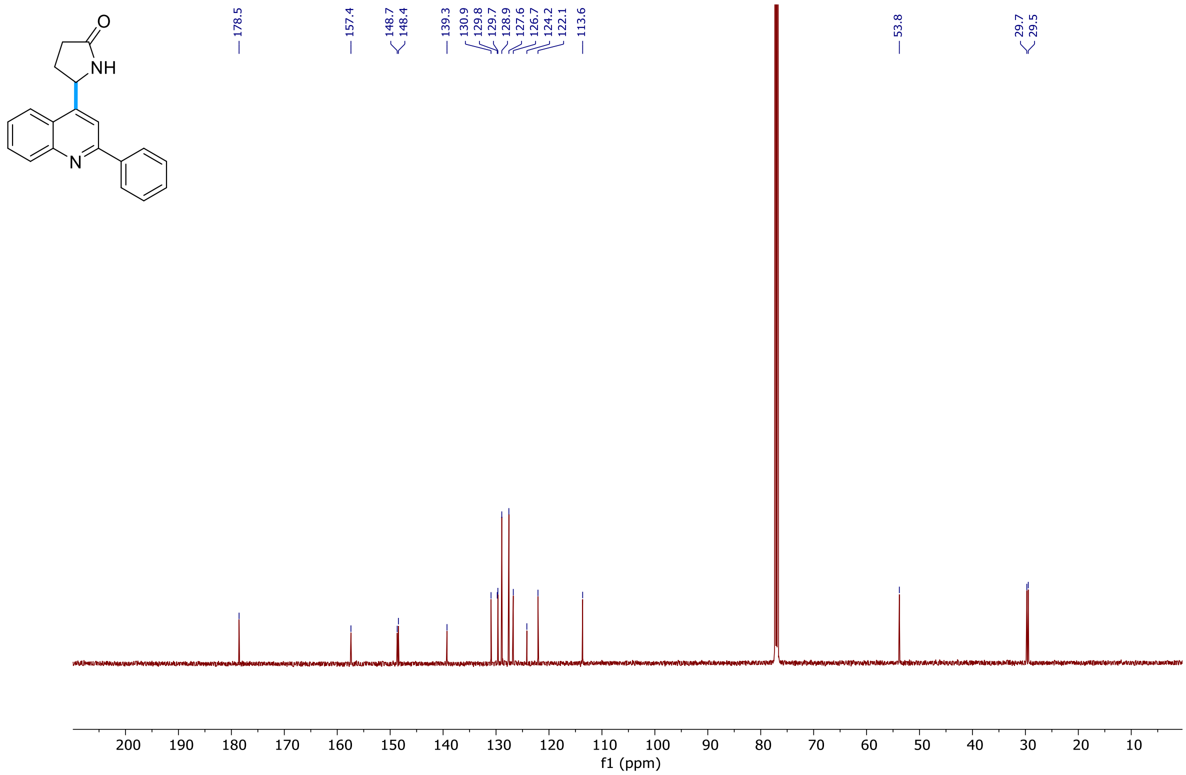

Compound **19** ^{1}H-NMR

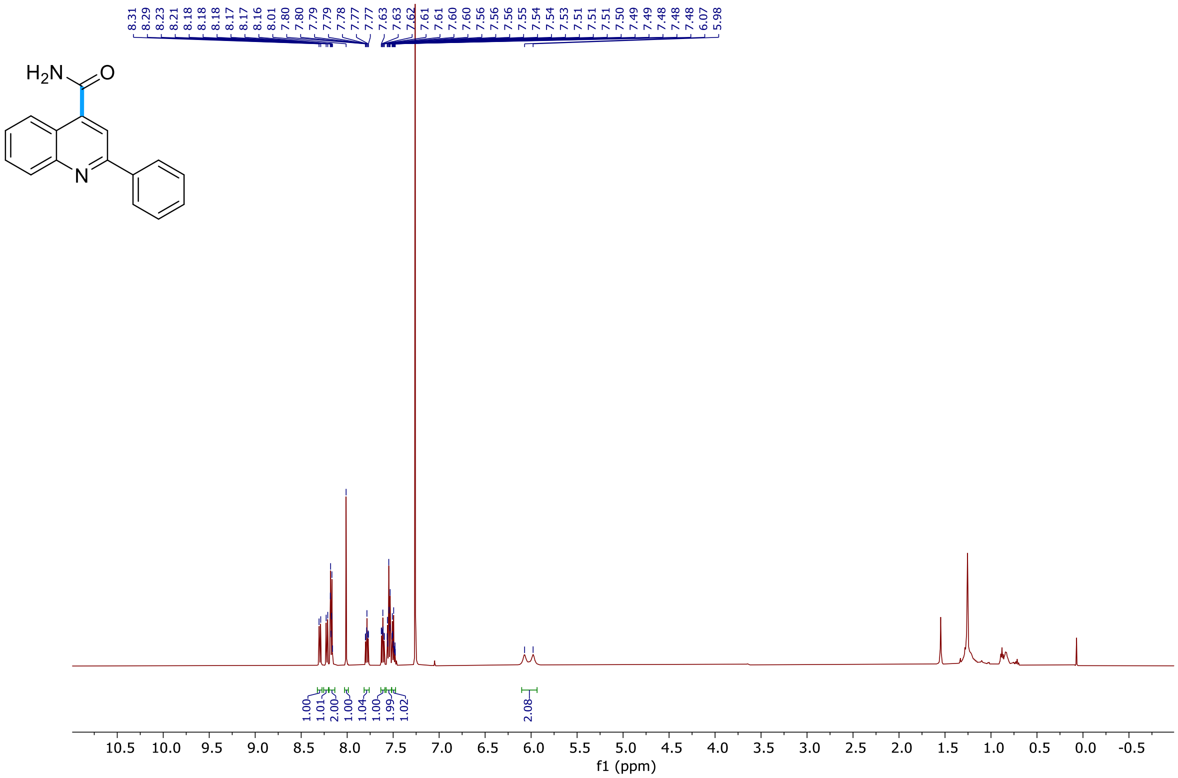

Compound **19** ^{13}C-NMR

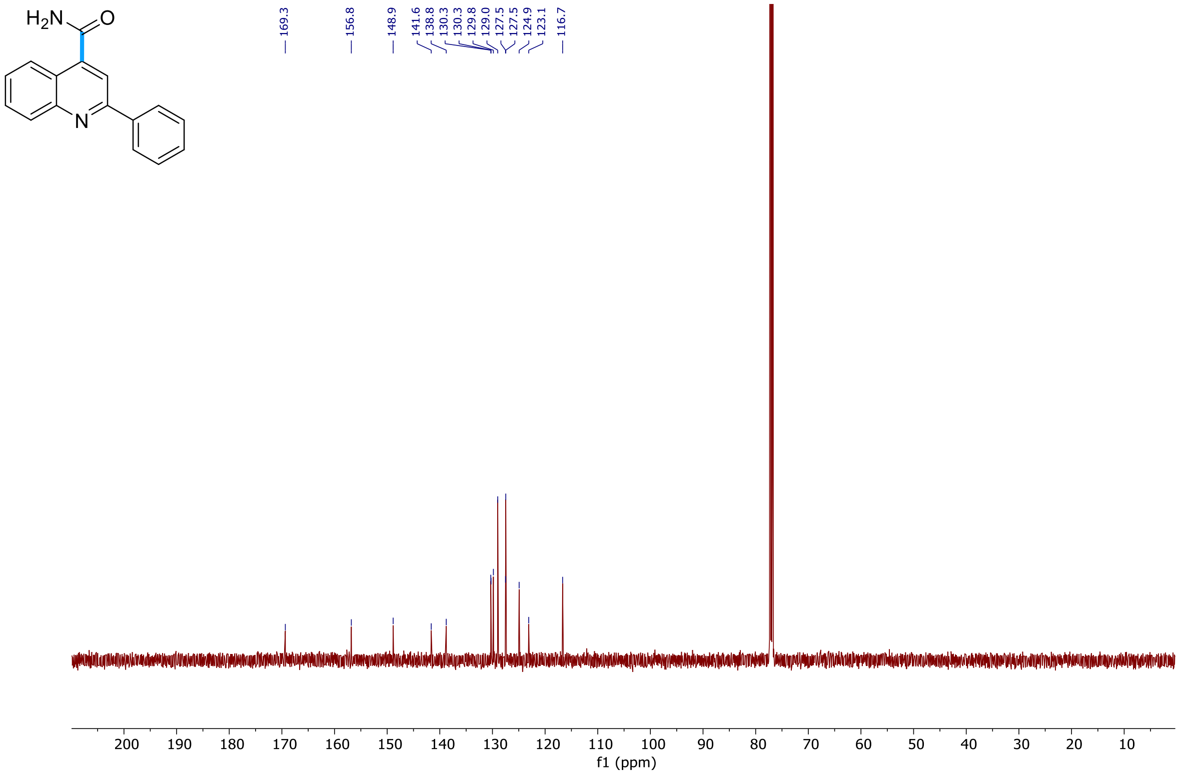

Compound **20** ^{1}H-NMR

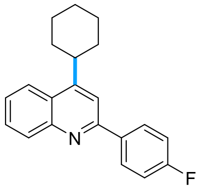

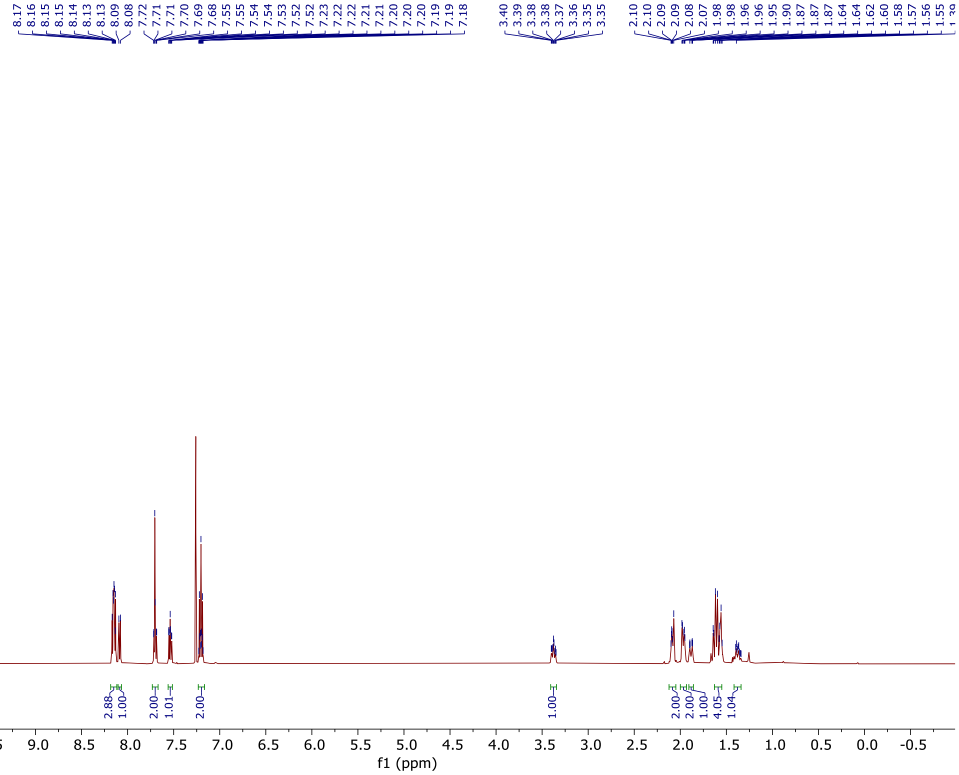

Compound **20** ^{13}C-NMR

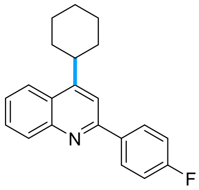

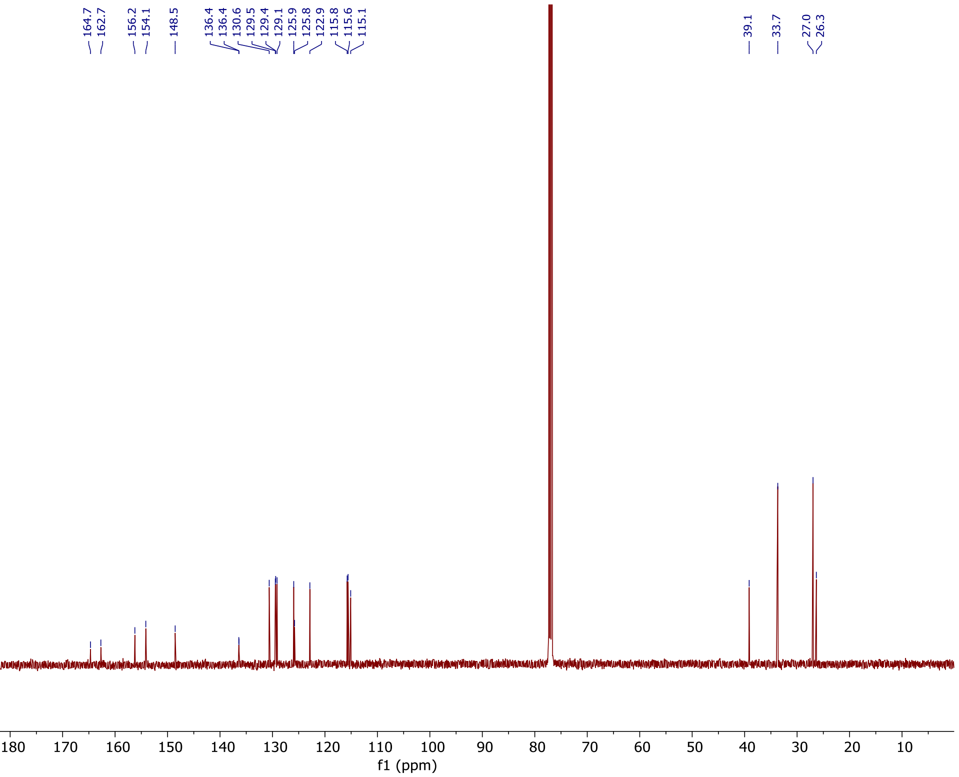

Compound **20** ^{19}F-NMR

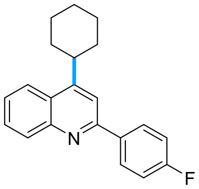

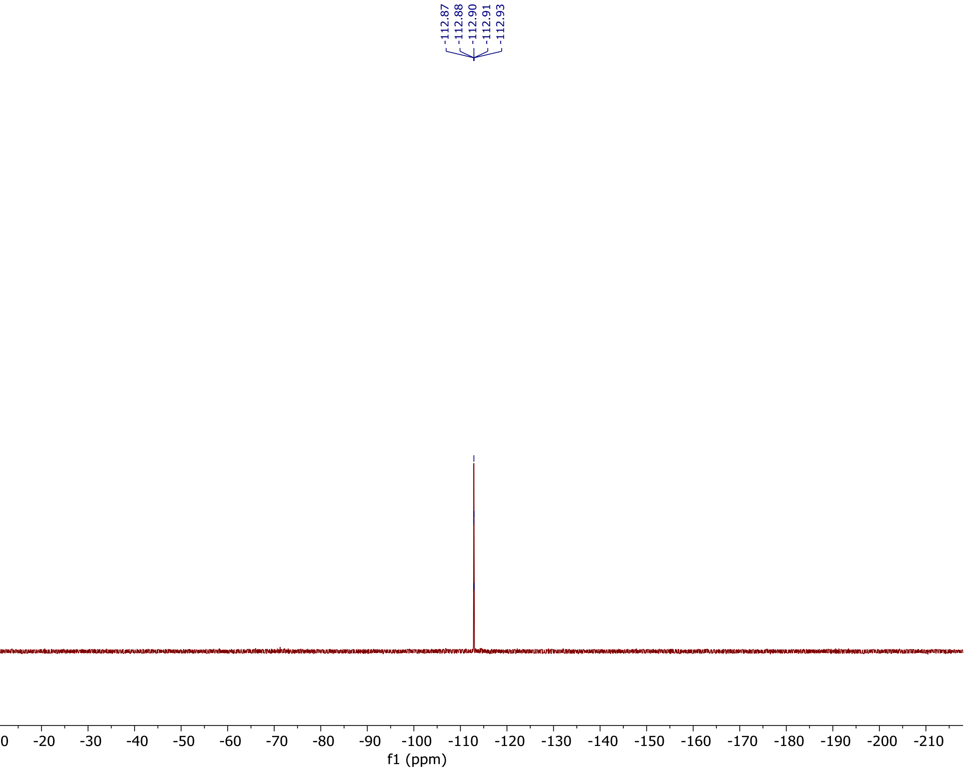

Compound **21** ^{1}H-NMR

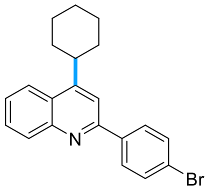

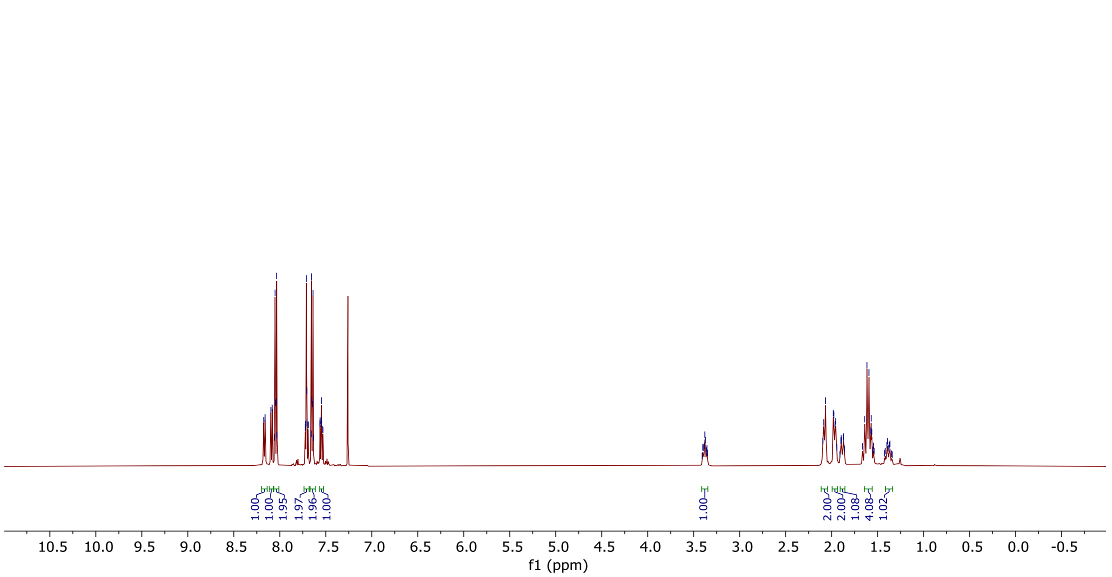

Compound **21** ^{13}C-NMR

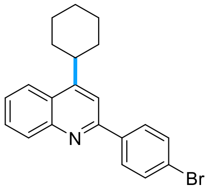

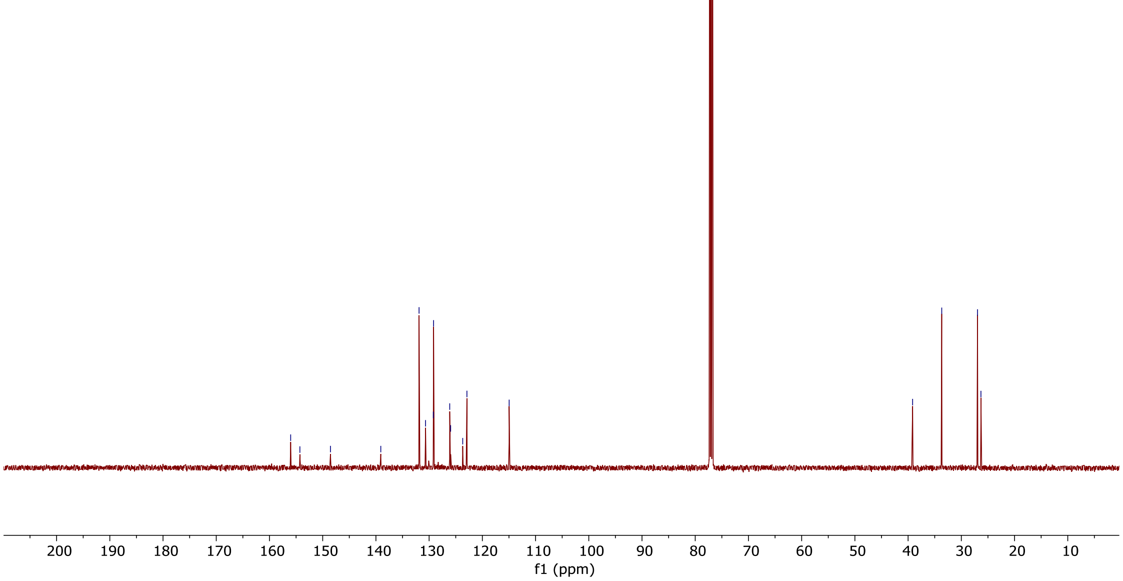

Compound **22** ^{1}H-NMR

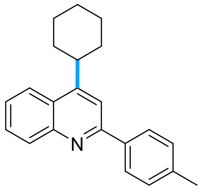

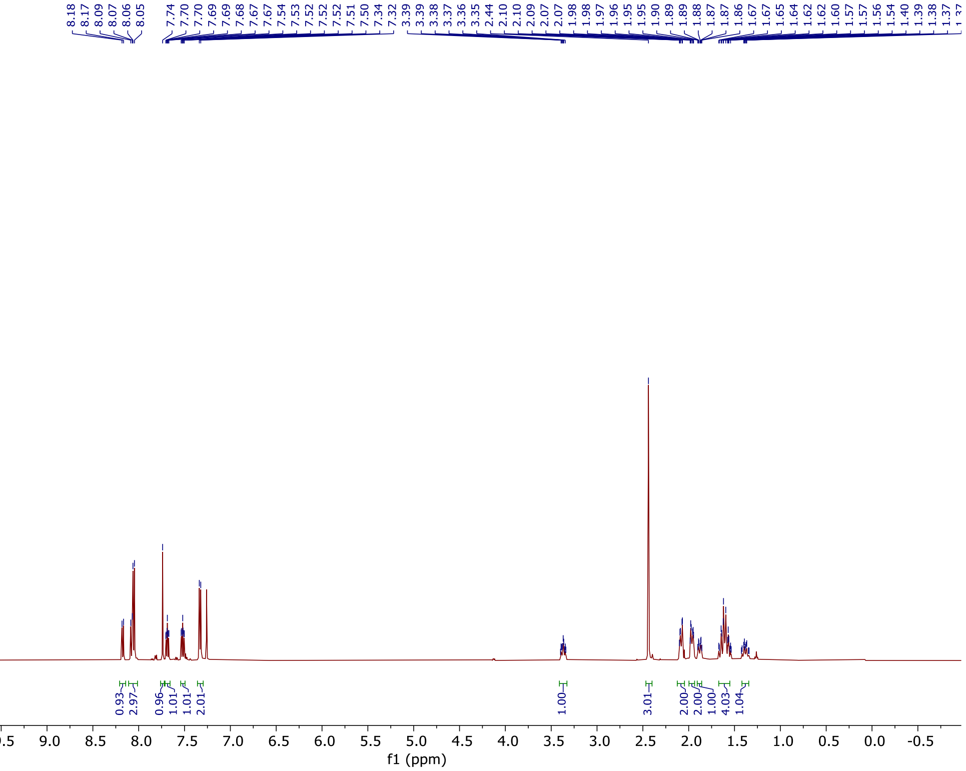

Compound **22** ^{13}C-NMR

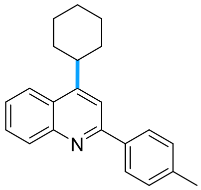

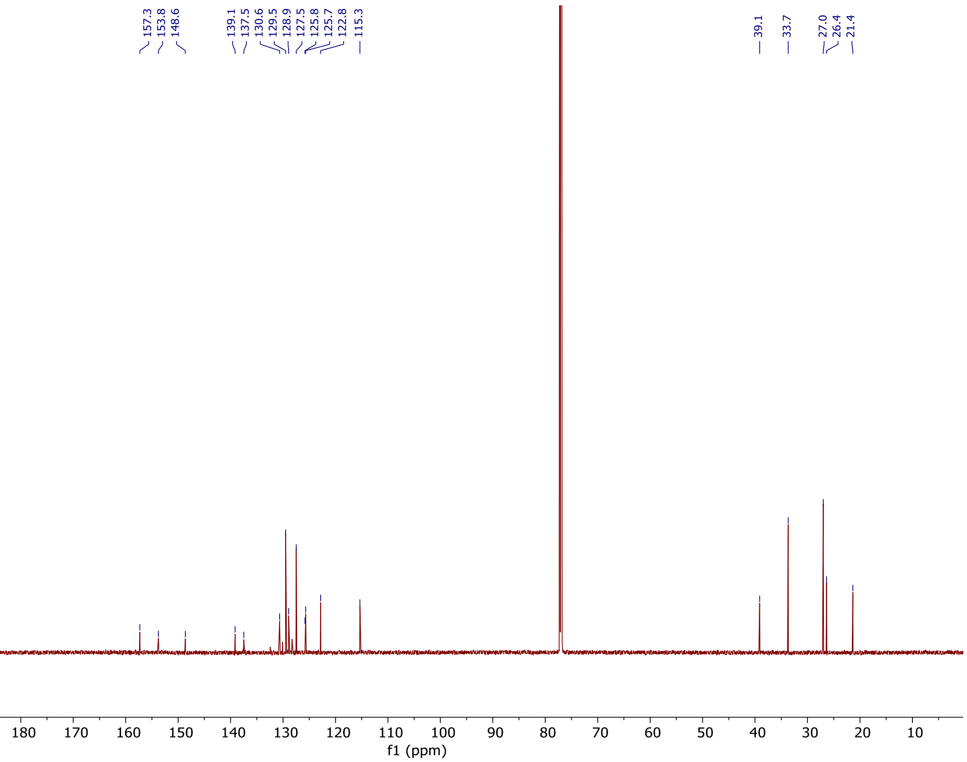

Compound **23** ^{1}H-NMR

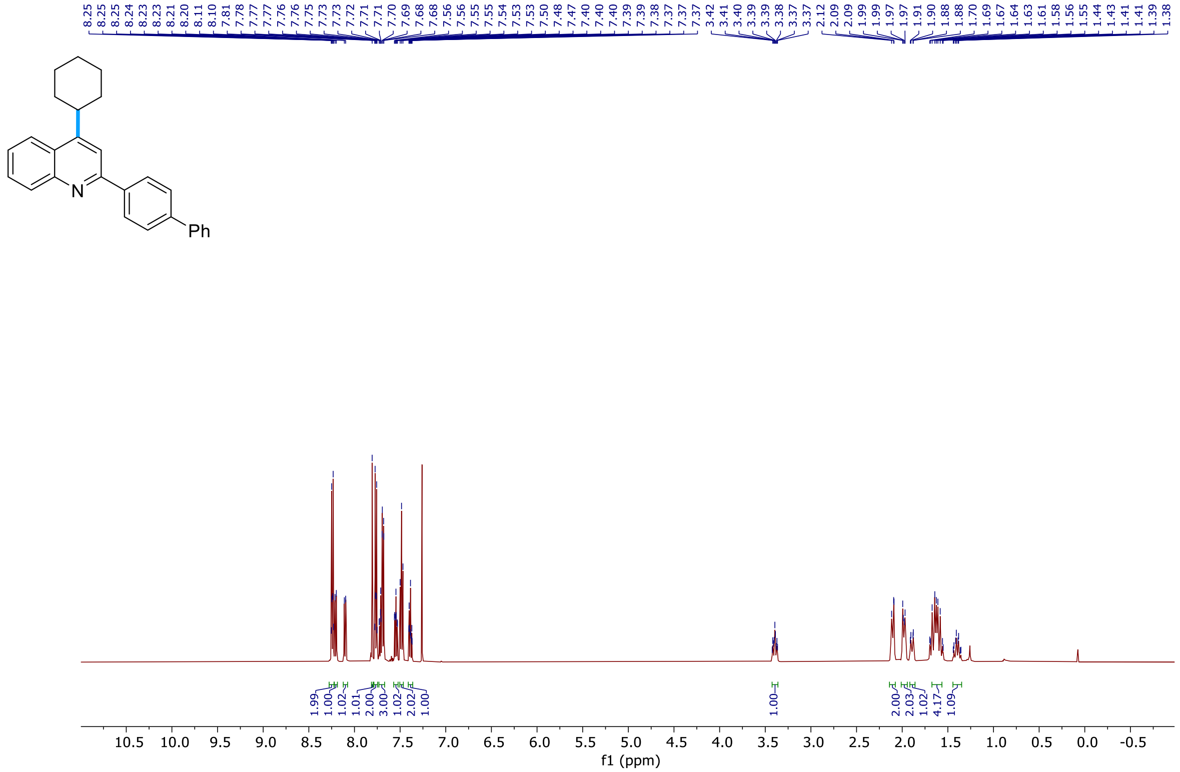

Compound **23** ^{13}C-NMR

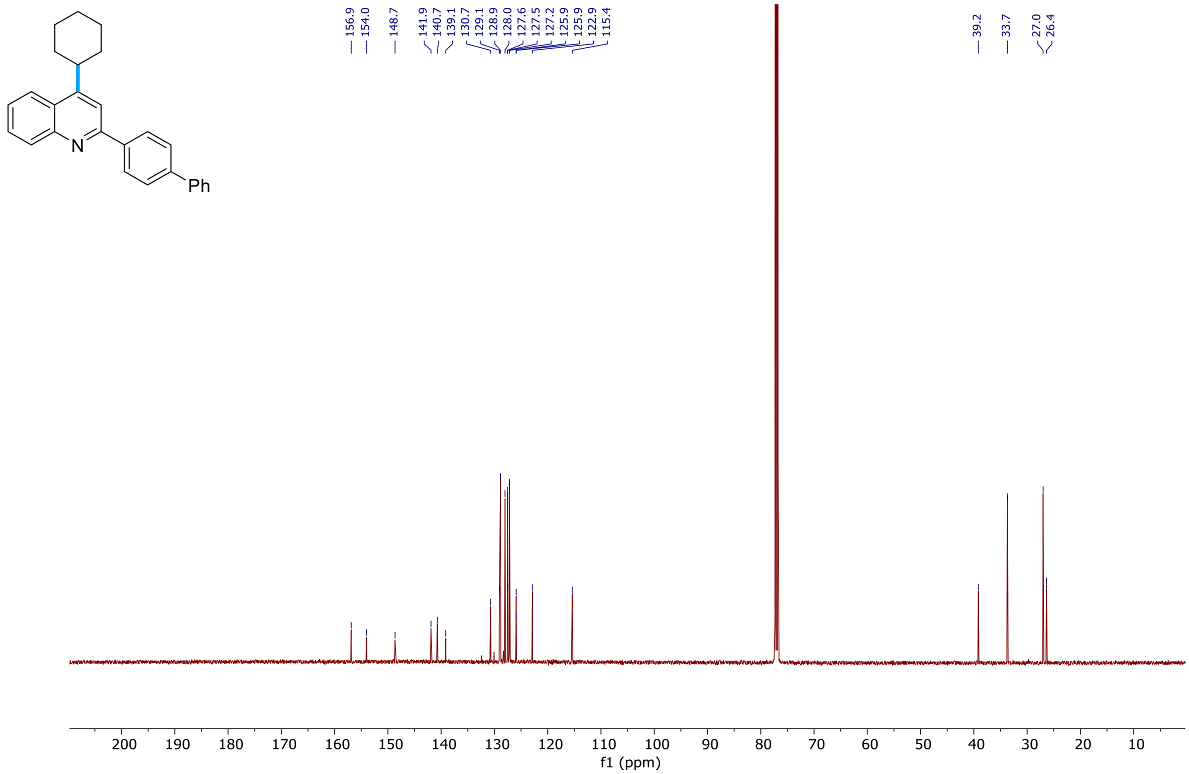

Compound **24** ^{1}H-NMR

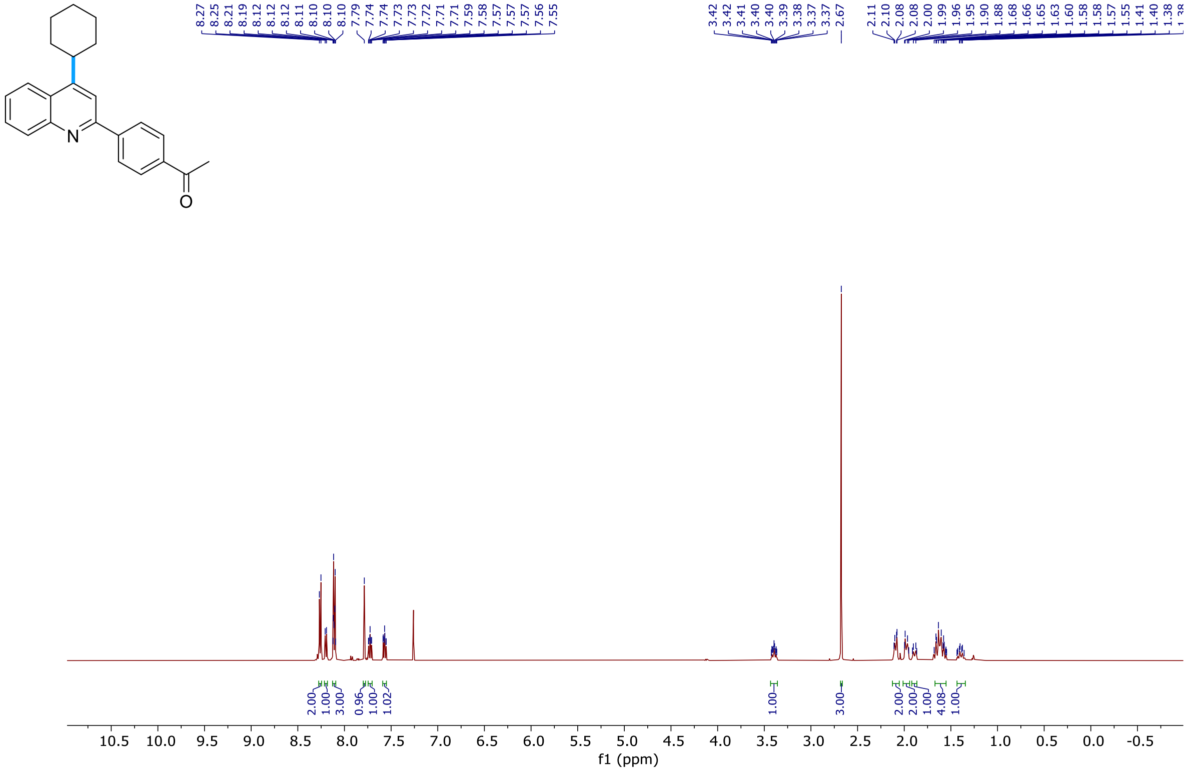

Compound **24** ^{13}C-NMR

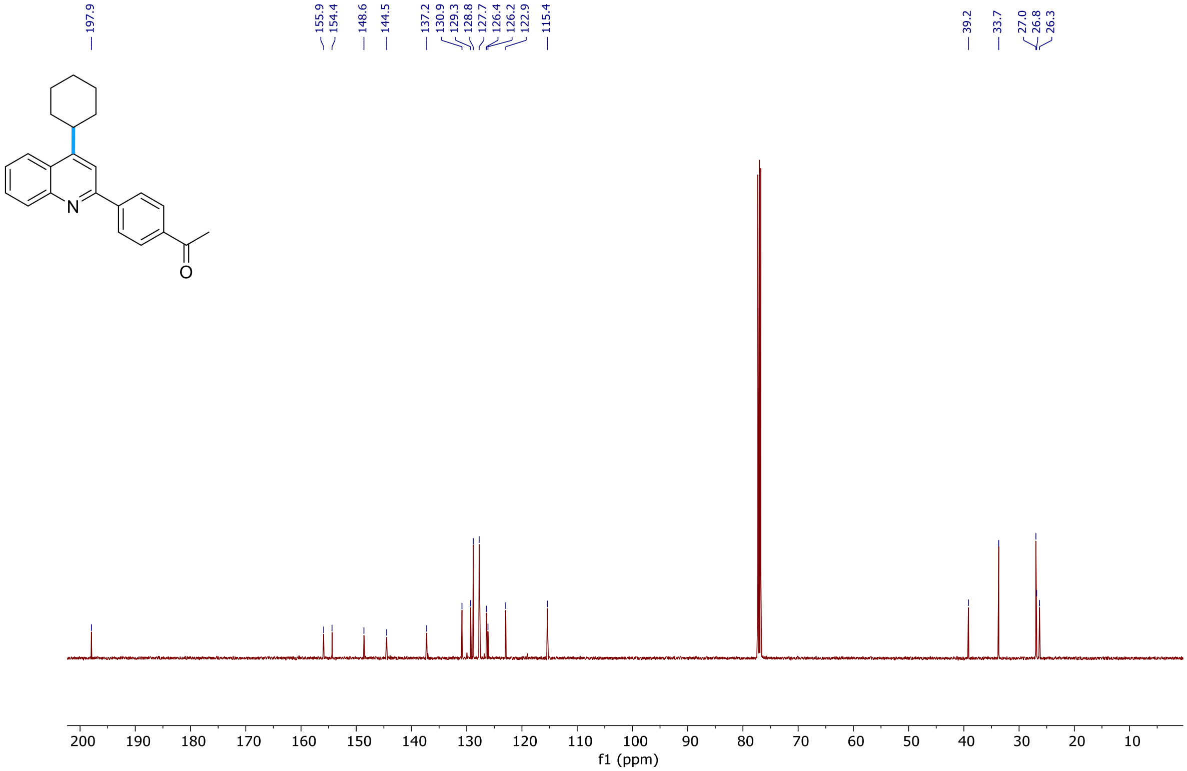

Compound **25** ^{1}H-NMR

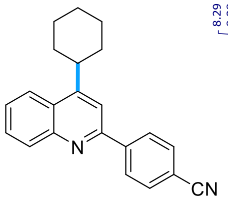

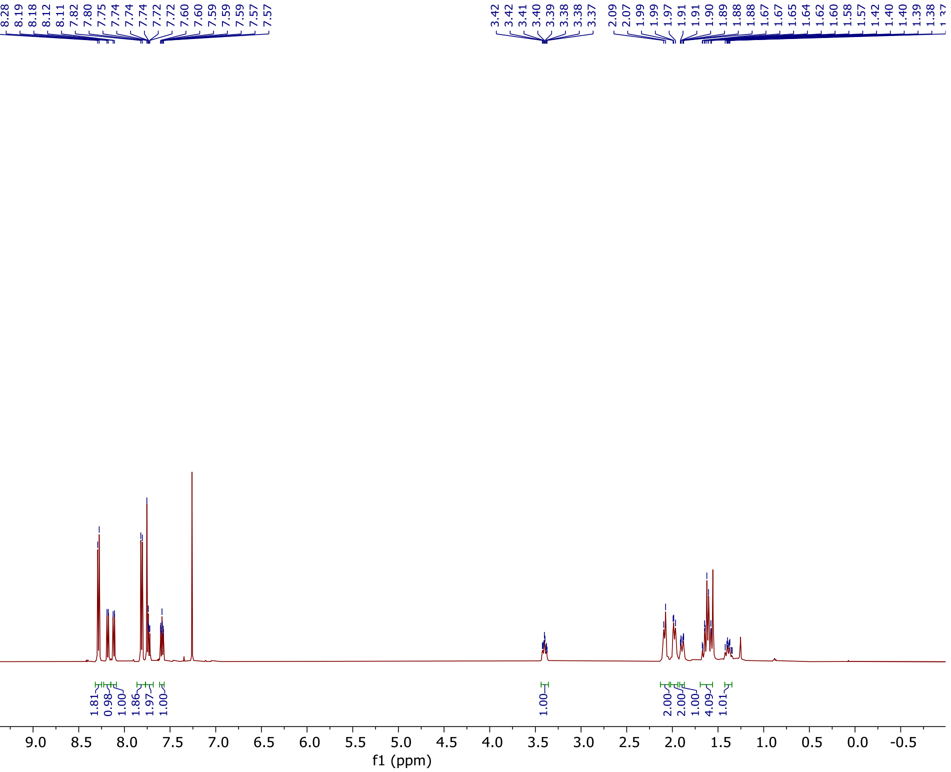

Compound **25** ^{13}C-NMR

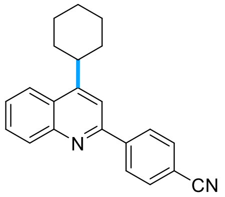

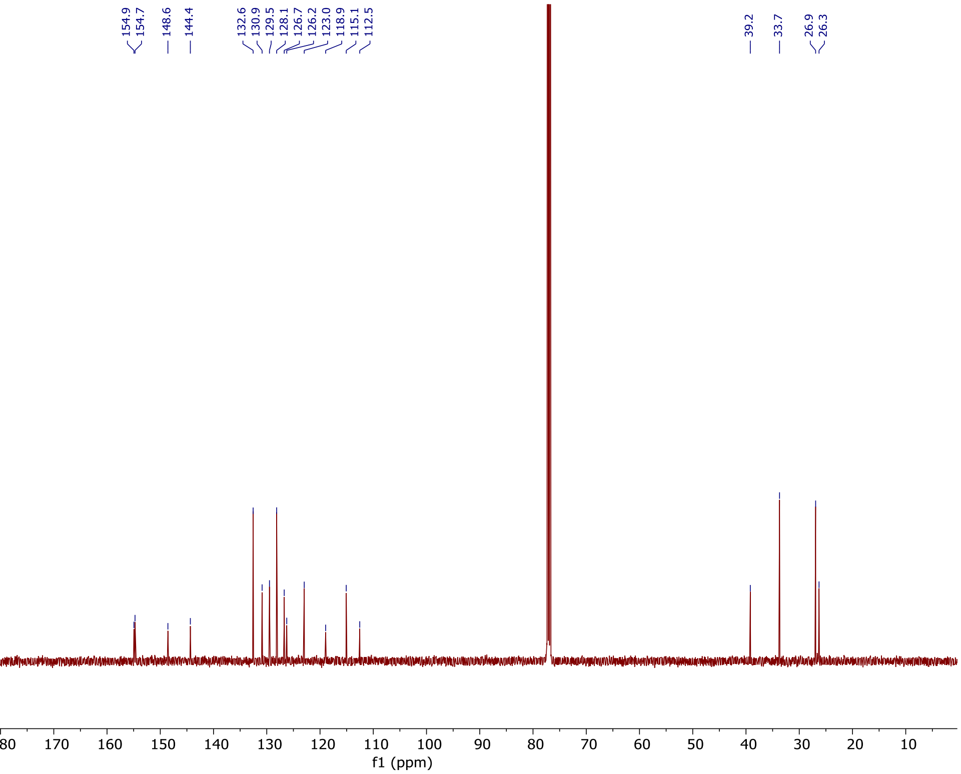

Compound **26** ¹H-NMR

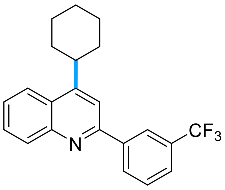

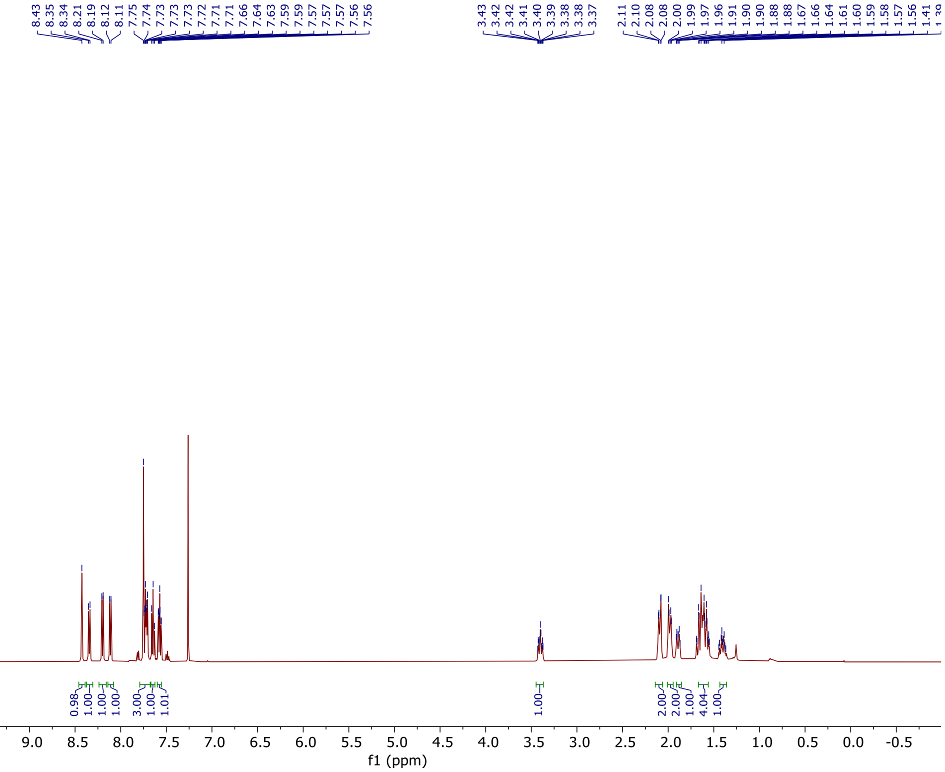

Compound **26** ¹³C-NMR

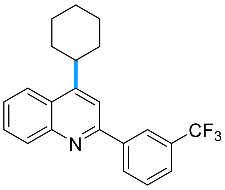

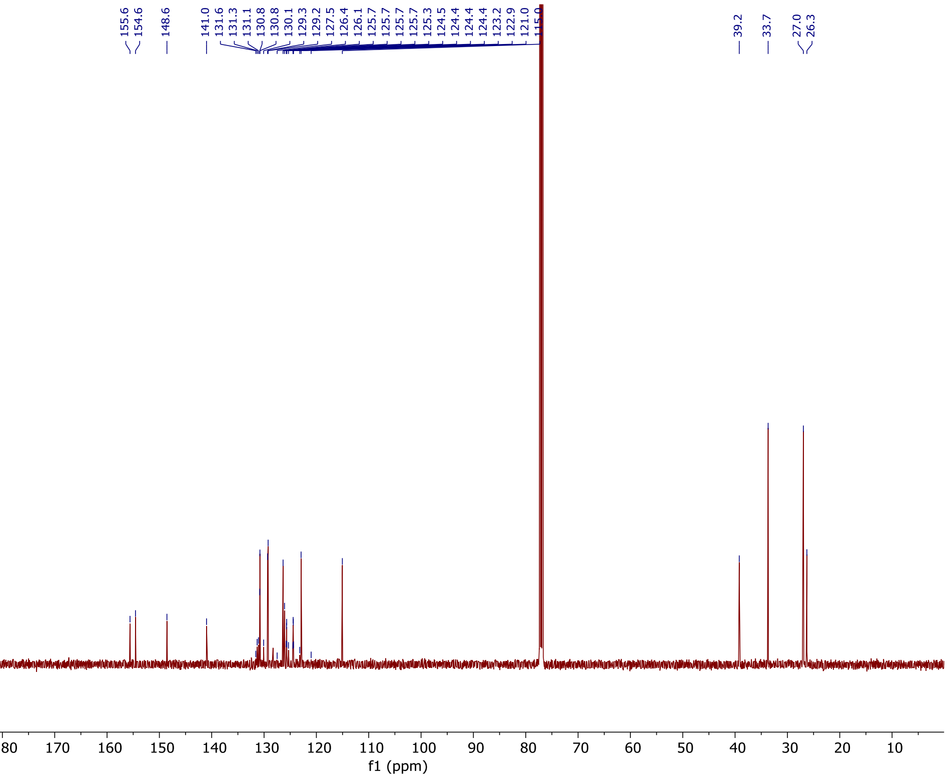

Compound **26** ^{19}F-NMR

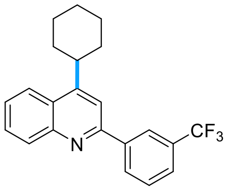

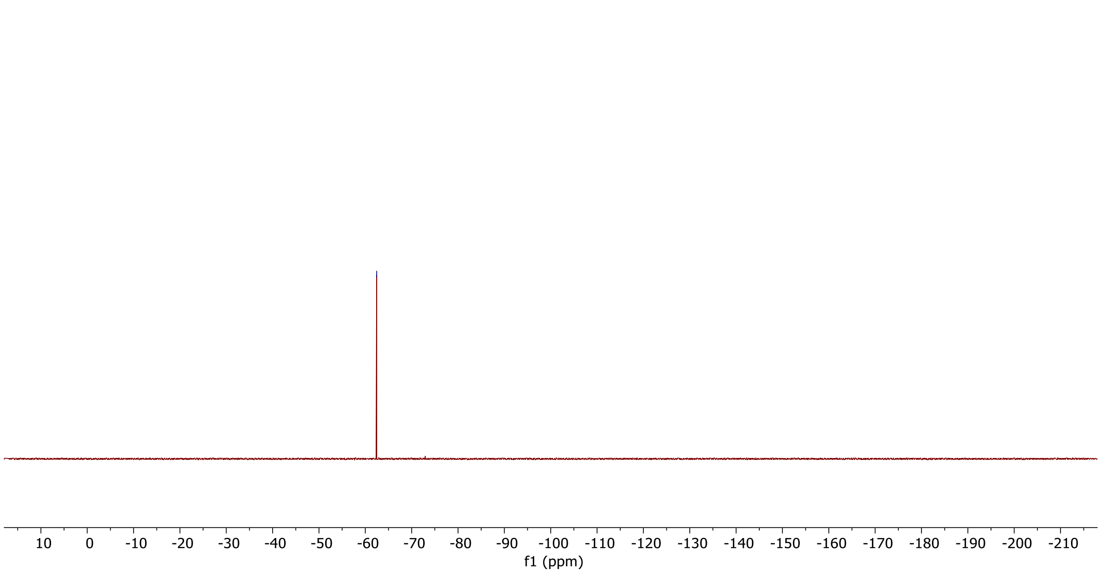

Compound **27** ^{1}H-NMR

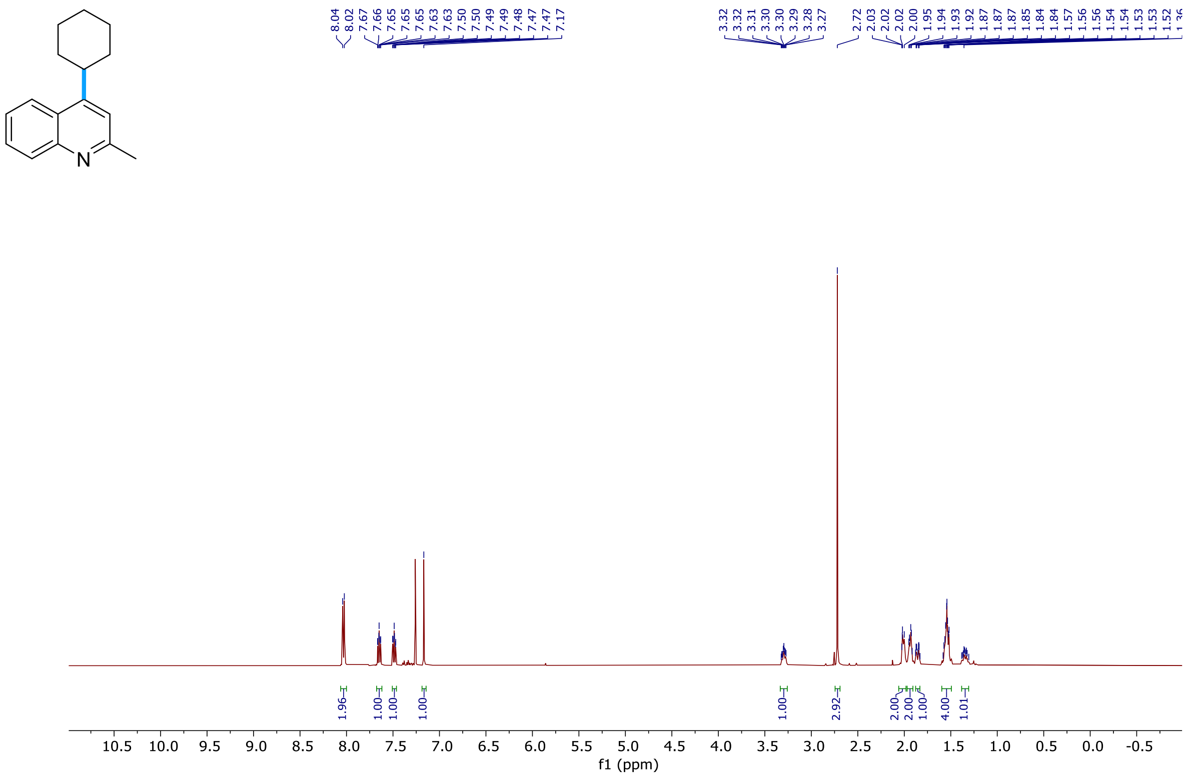

Compound **27** ^{13}C-NMR

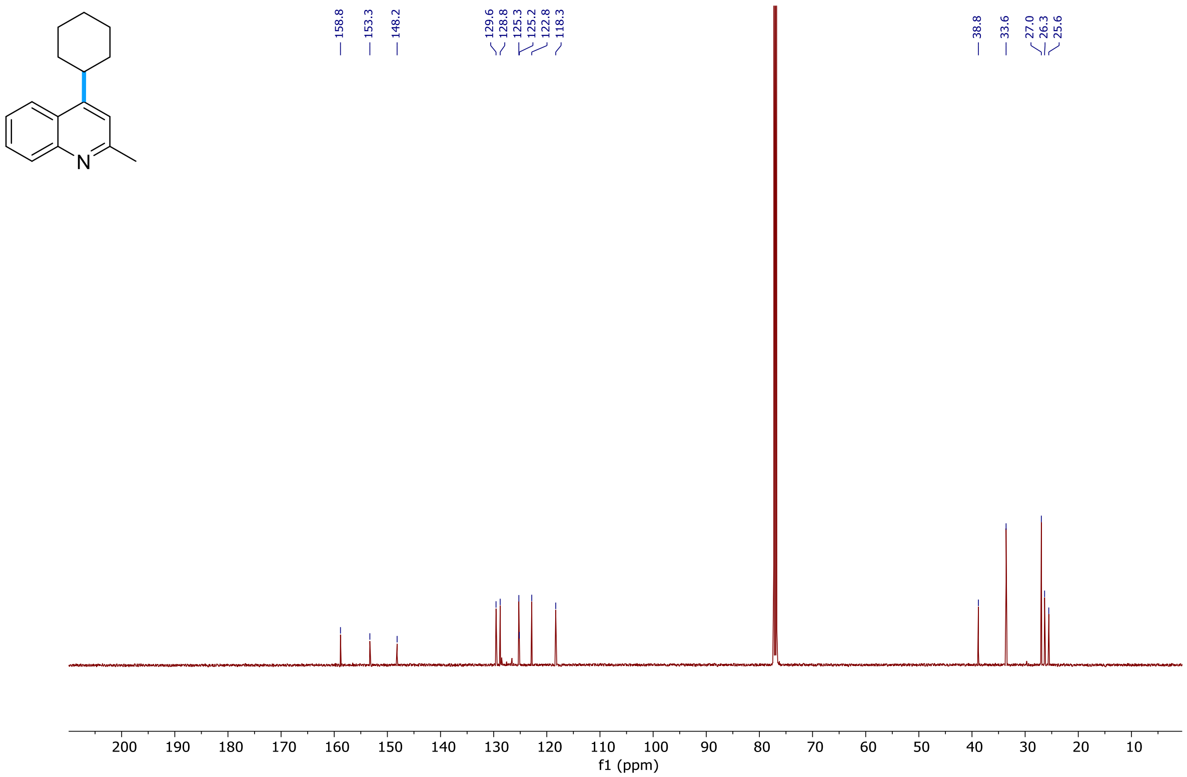

Compound **28** ^{1}H-NMR

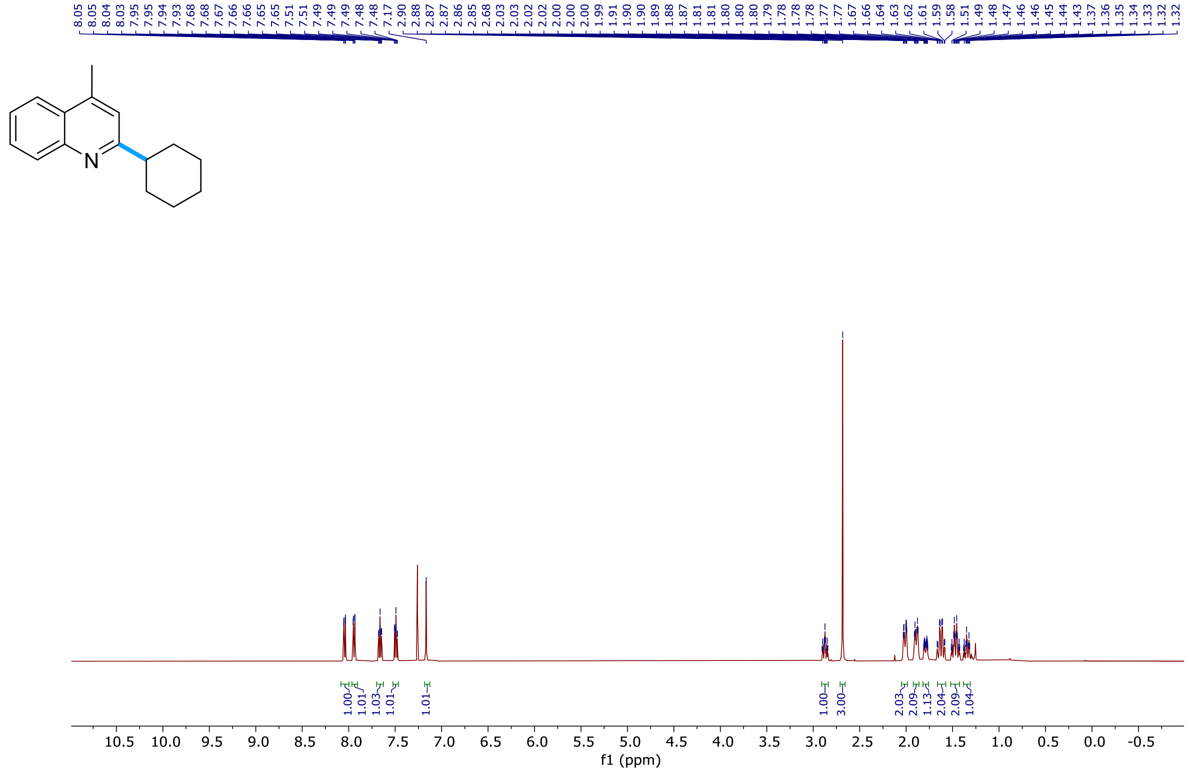

Compound **28** ^{13}C-NMR

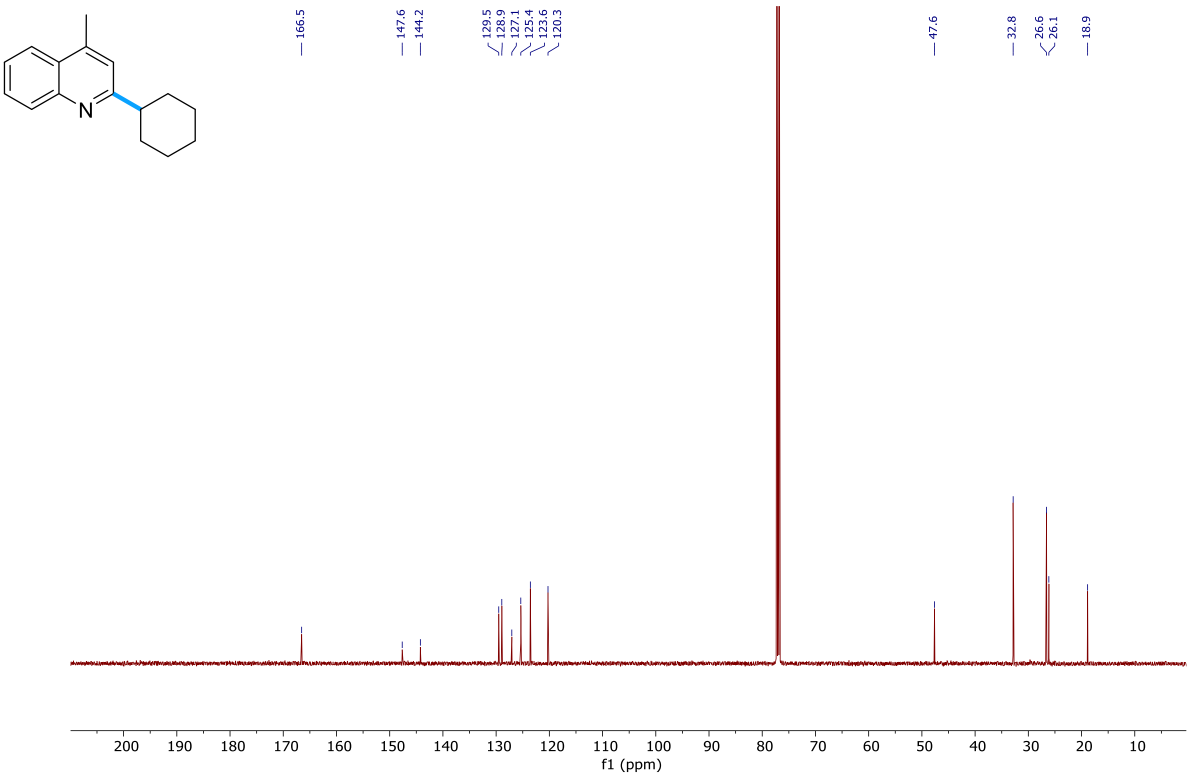

Compound **29** ^{1}H-NMR

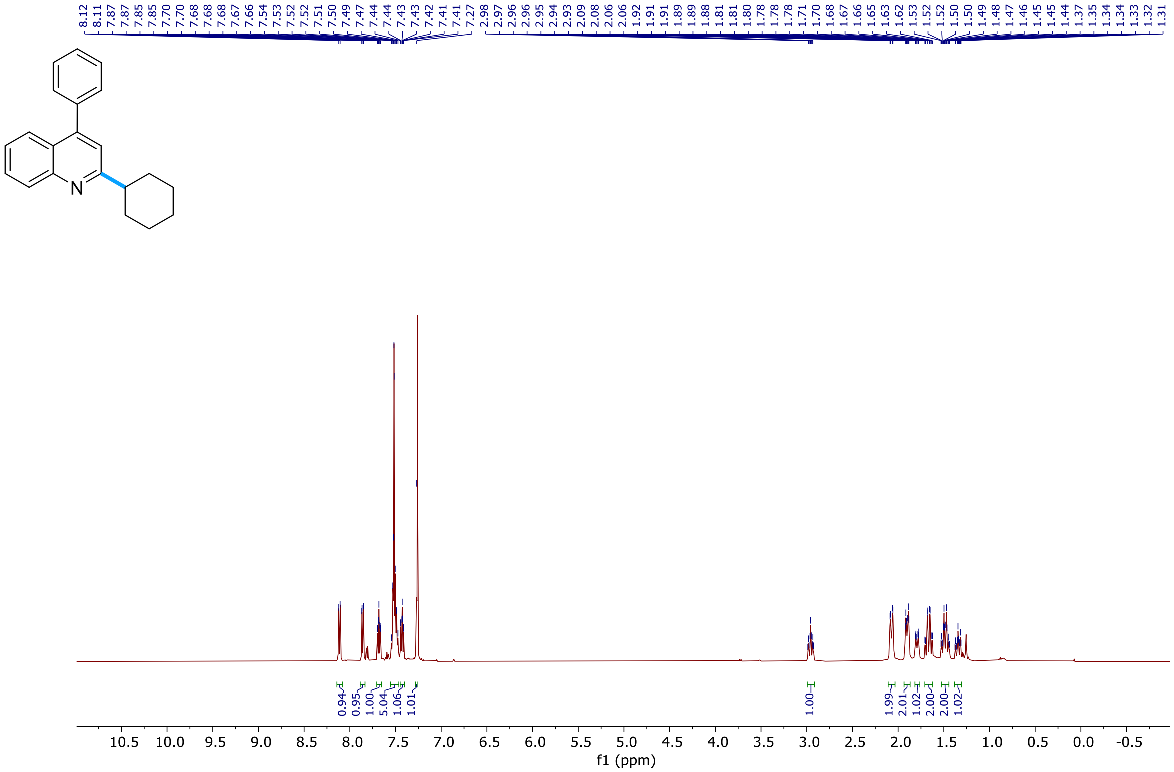

Compound **29** ^{13}C-NMR

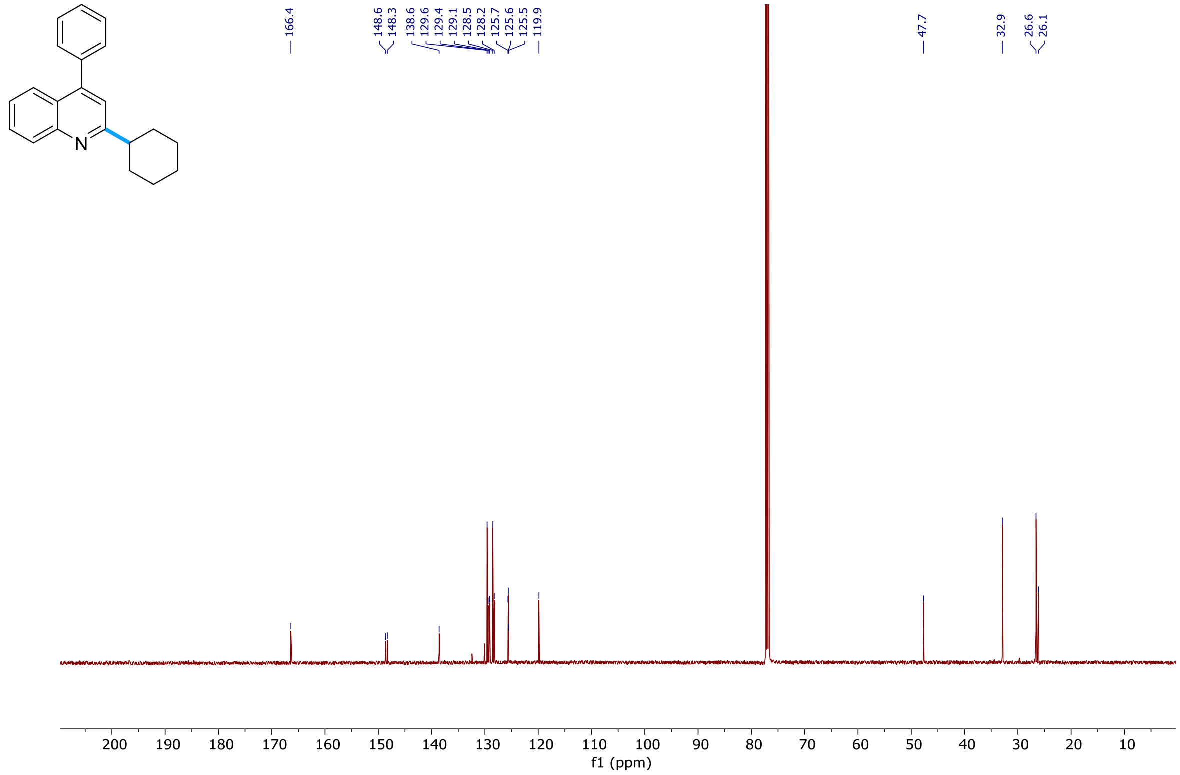

Compound **30** ^{1}H-NMR

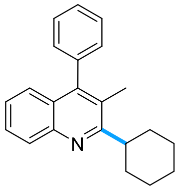

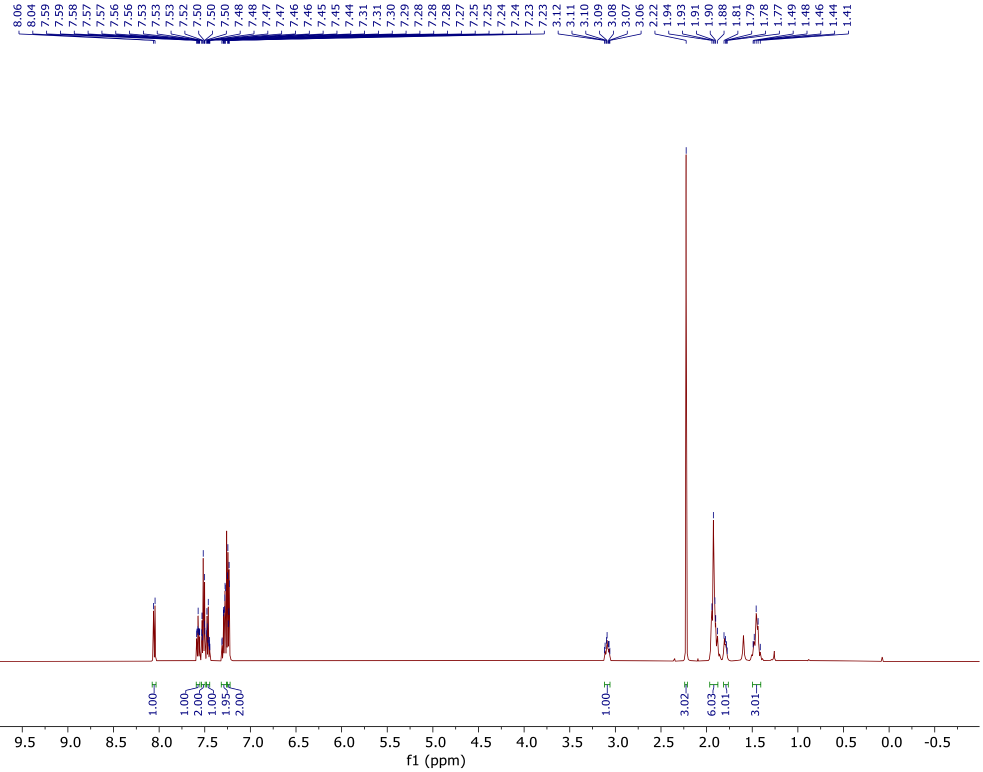

Compound **30** ^{13}C-NMR

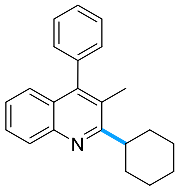

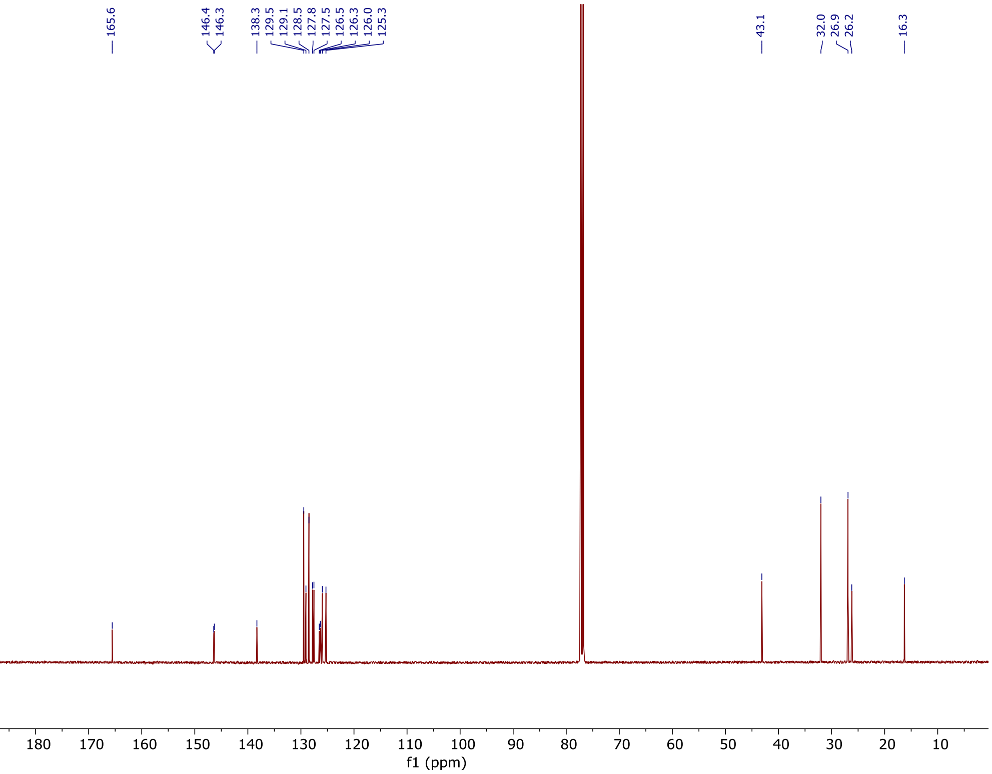

Compound **31** ^{1}H-NMR

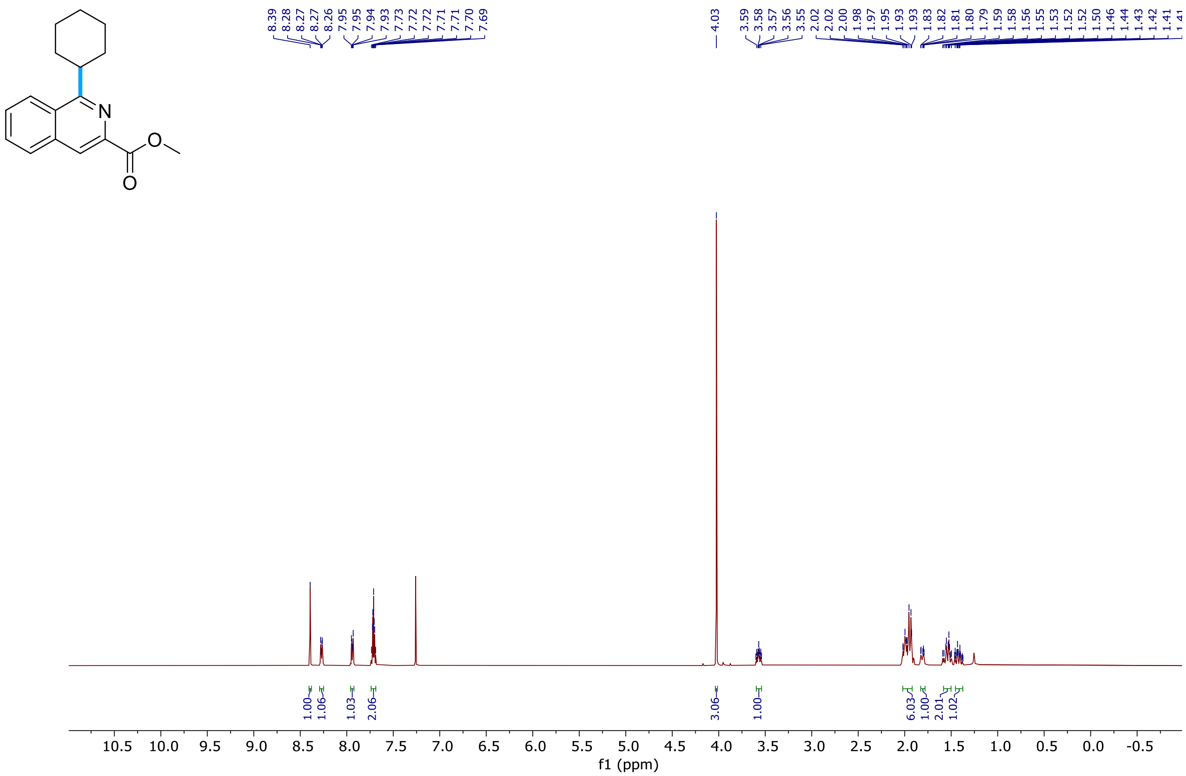

Compound **31** ^{13}C-NMR

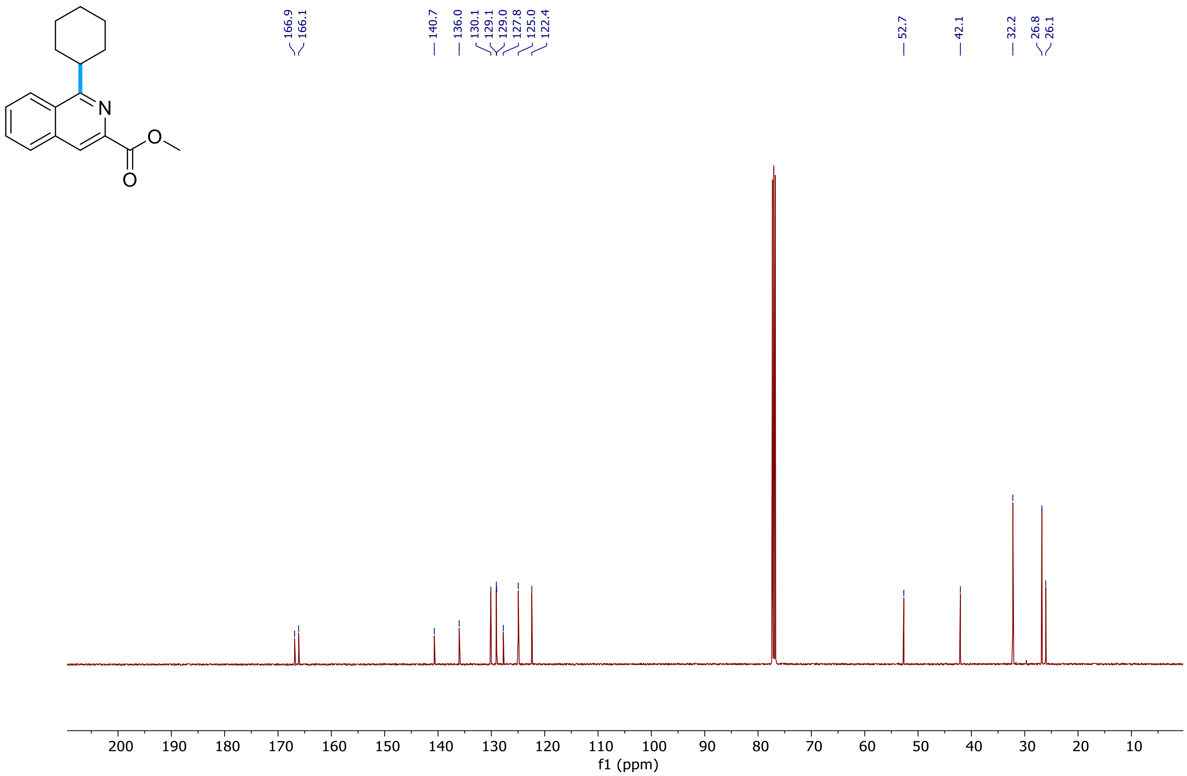

Compound **32a** ^{1}H-NMR

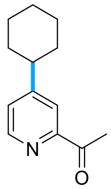

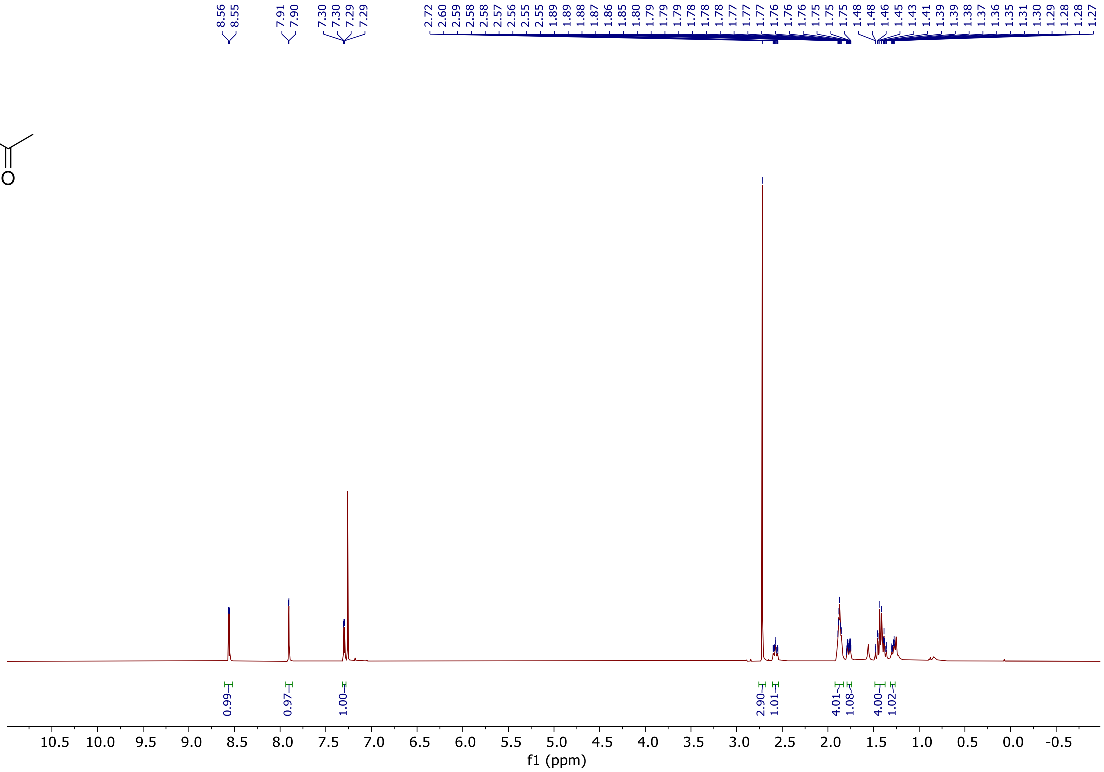

Compound **32a** ^{13}C-NMR

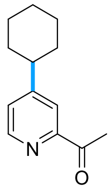

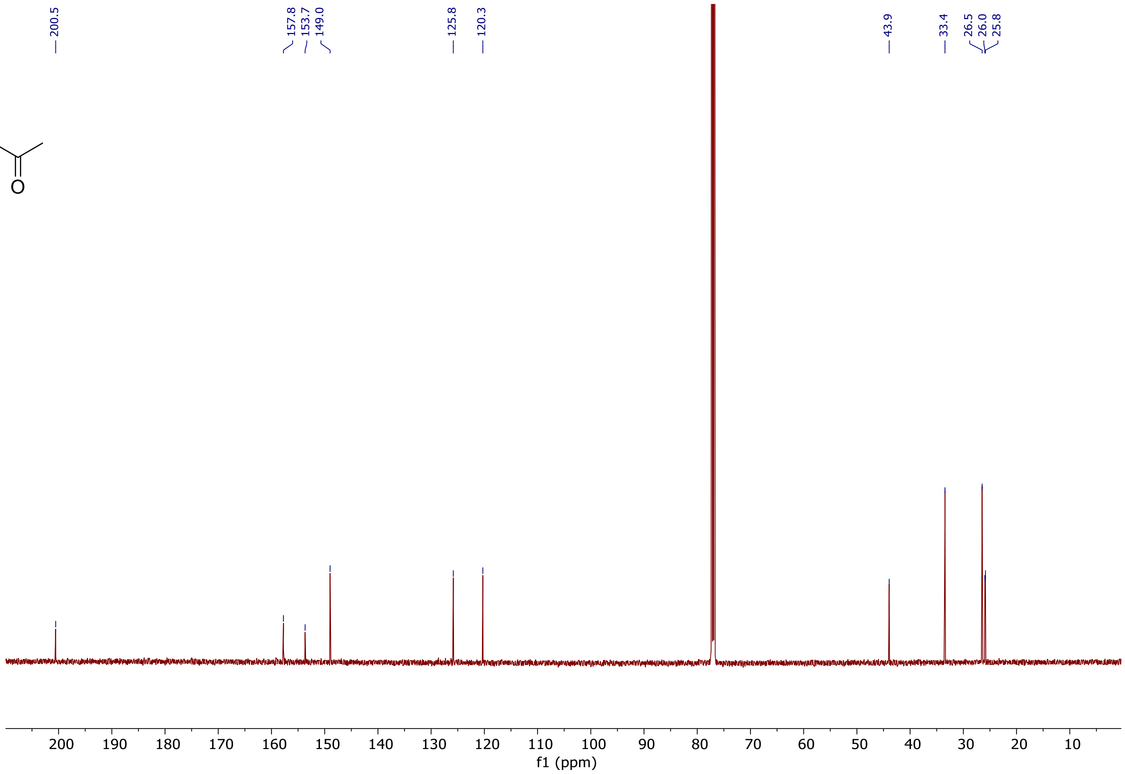

Compound **32b** ^{1}H-NMR

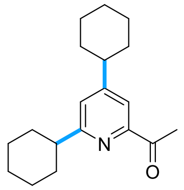

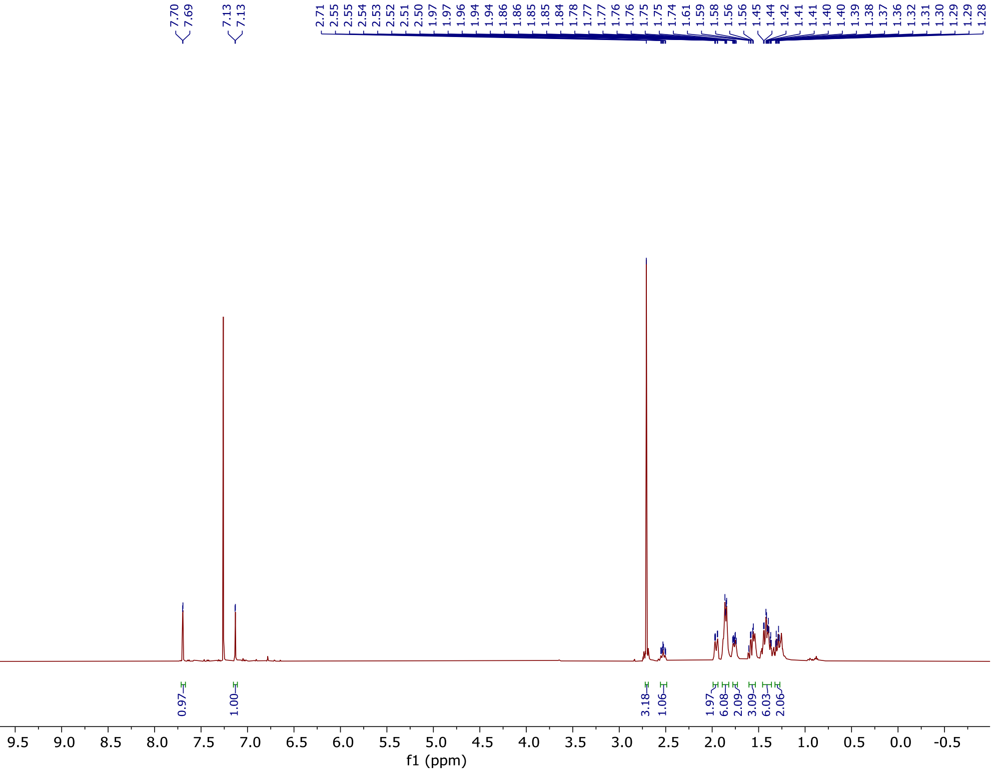

Compound **32b** ^{13}C-NMR

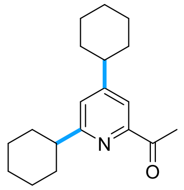

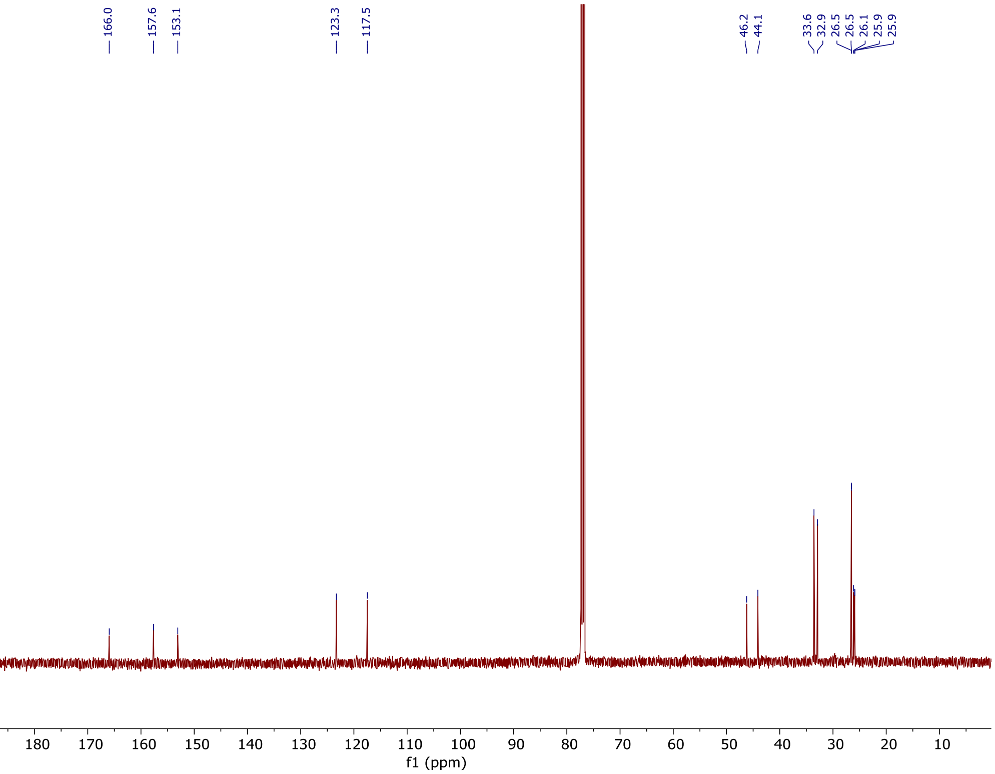

Compound **33** ^{1}H-NMR

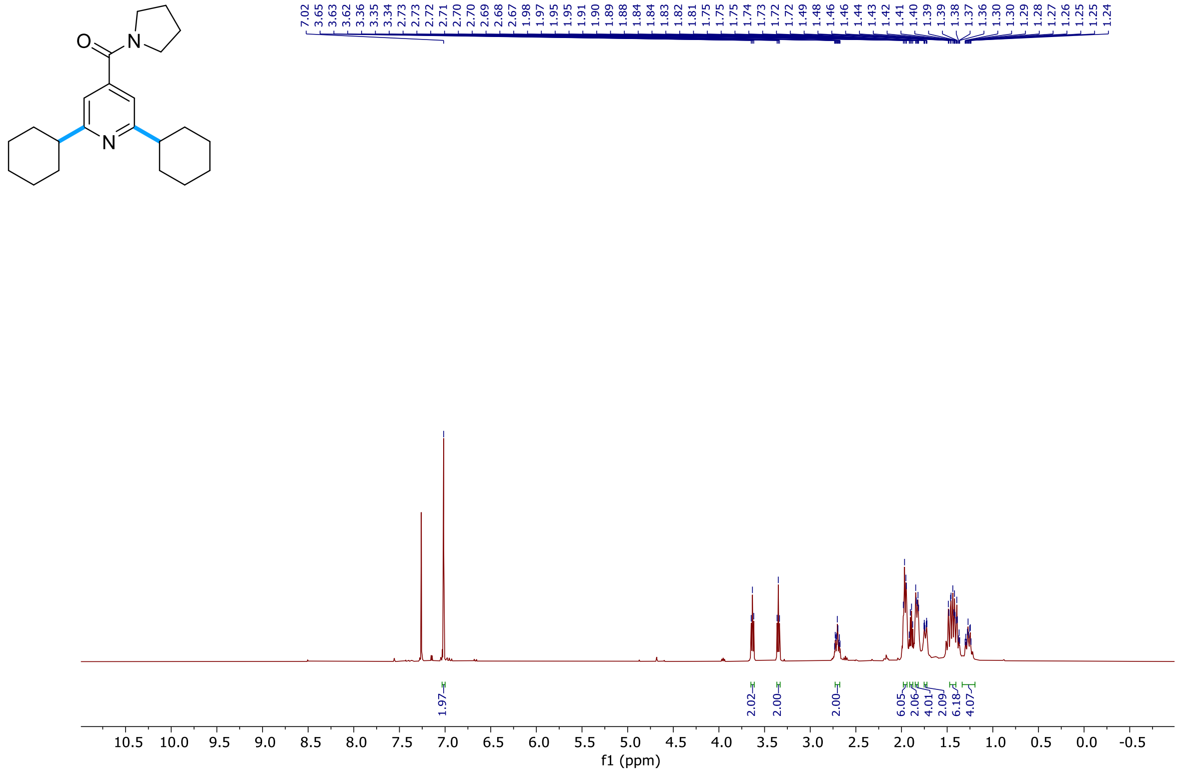

Compound **33** ^{13}C-NMR

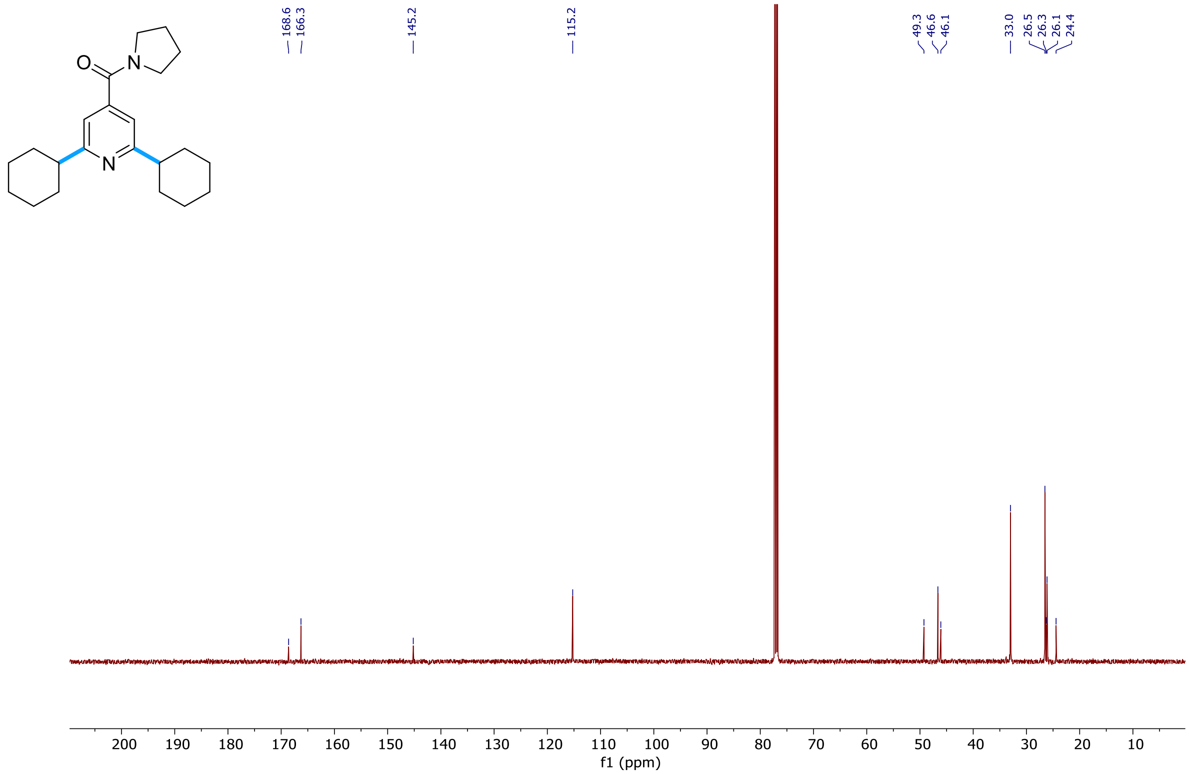

Compound **34** ^{1}H-NMR

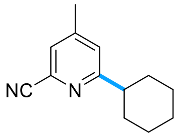

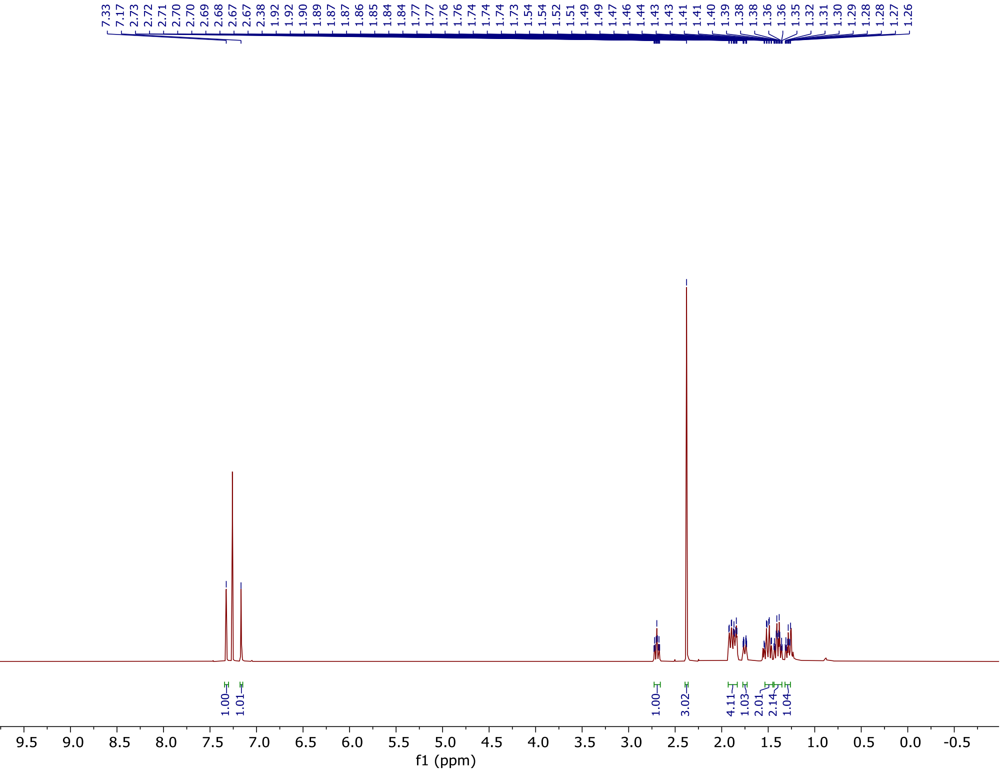

Compound **34** ^{13}C-NMR

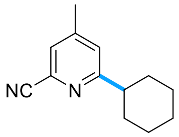

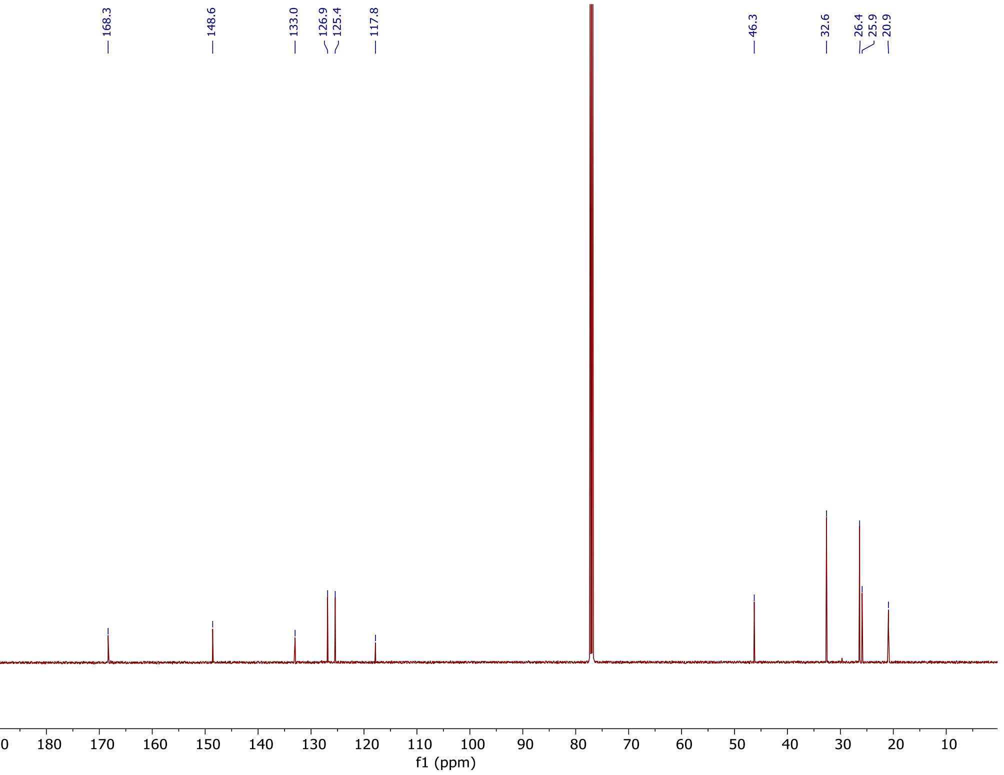

Compound **35** ^{1}H-NMR

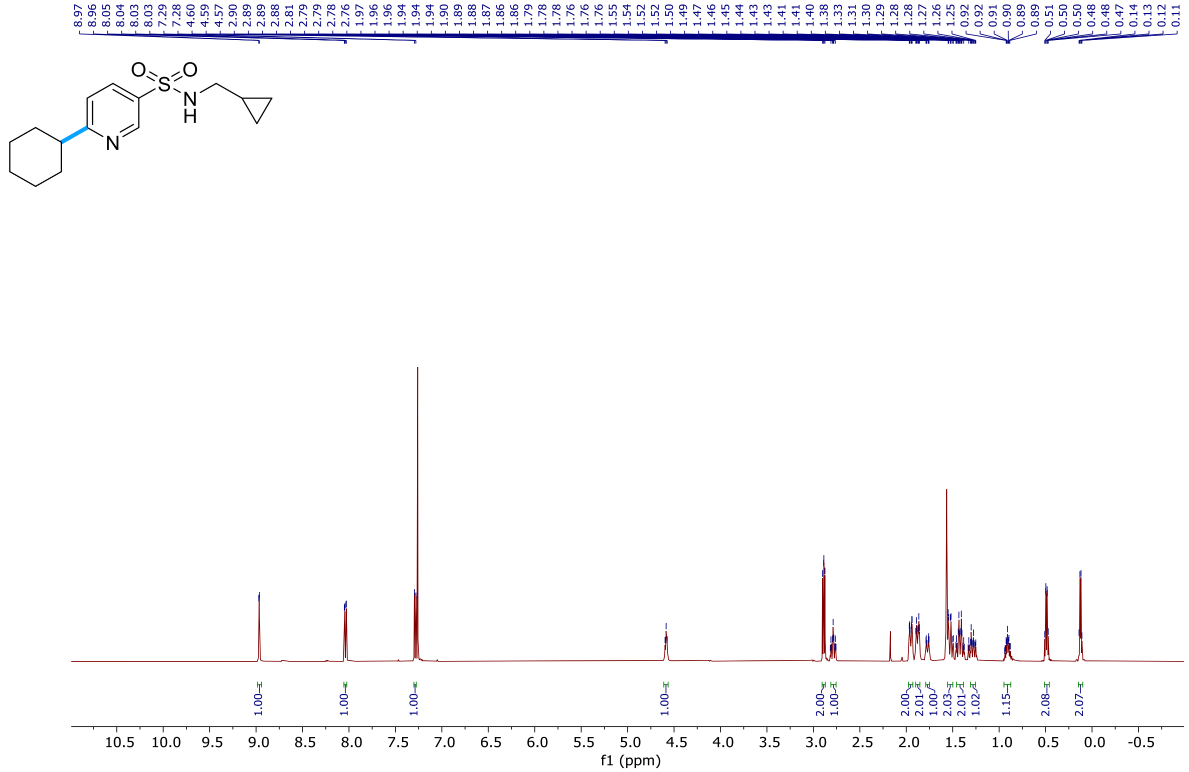

Compound **35** ^{13}C-NMR

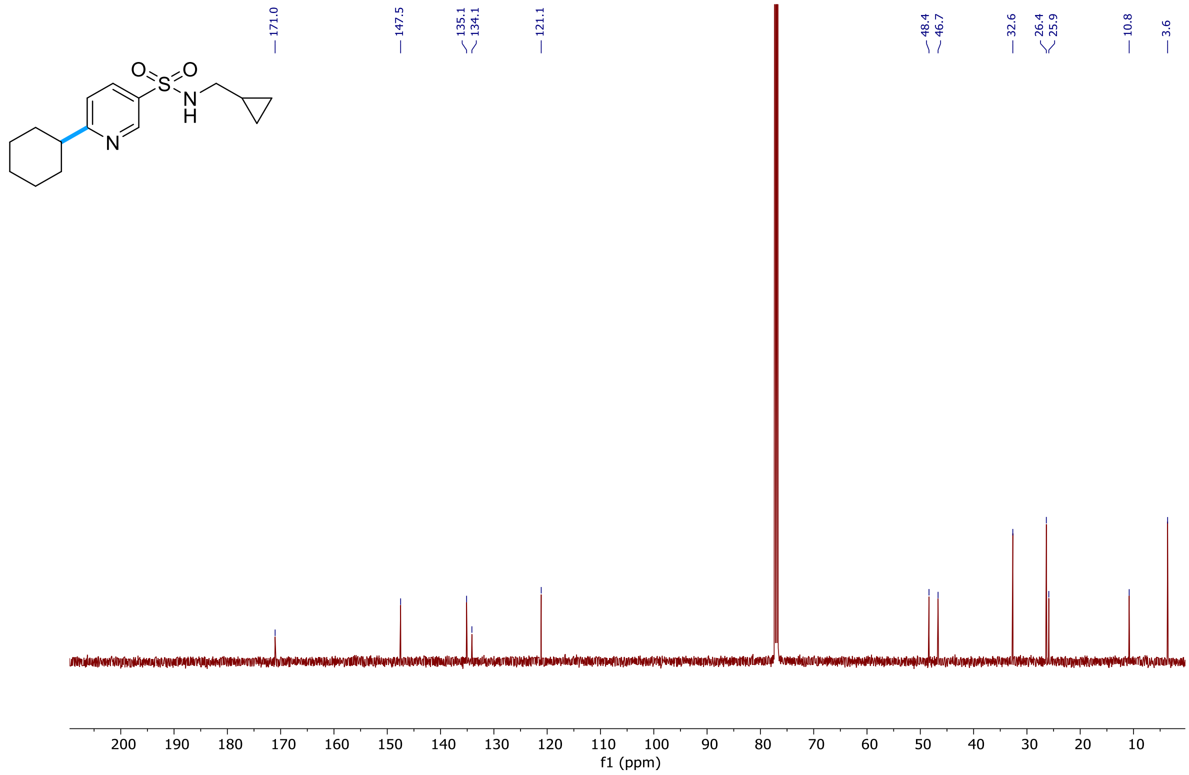

Compound **36** ^{1}H-NMR

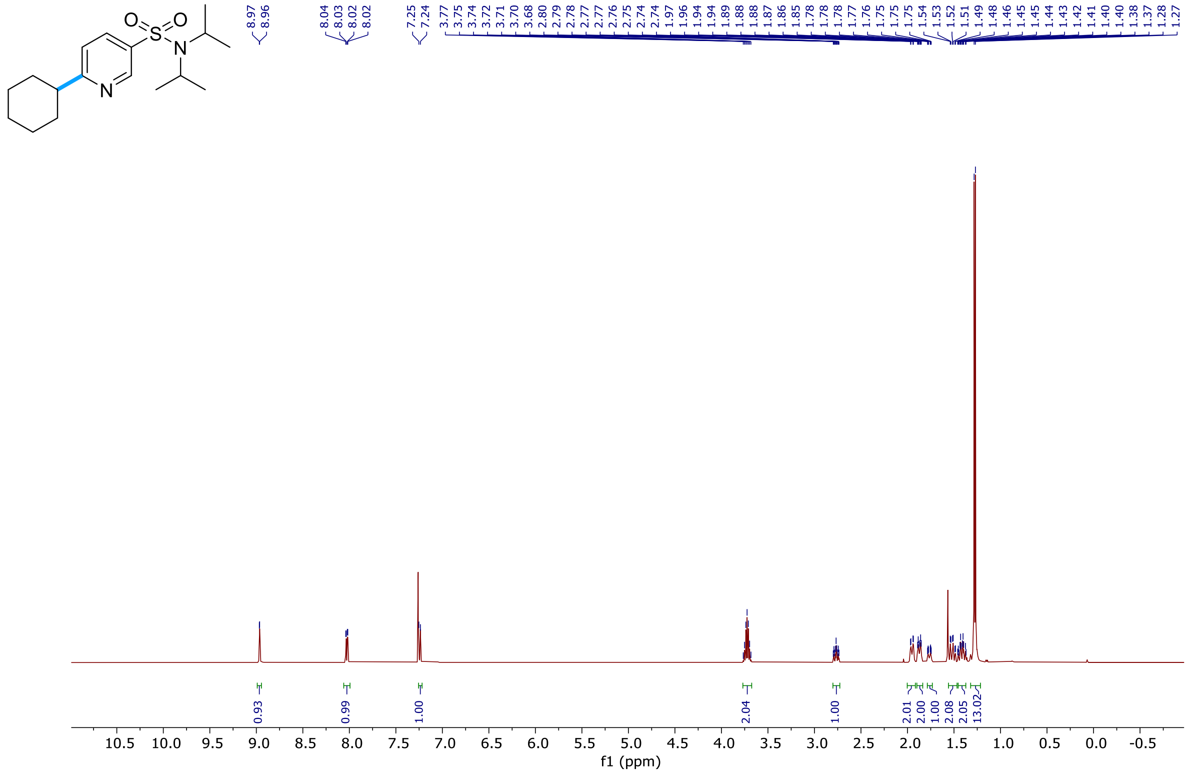

Compound **36** ^{13}C-NMR

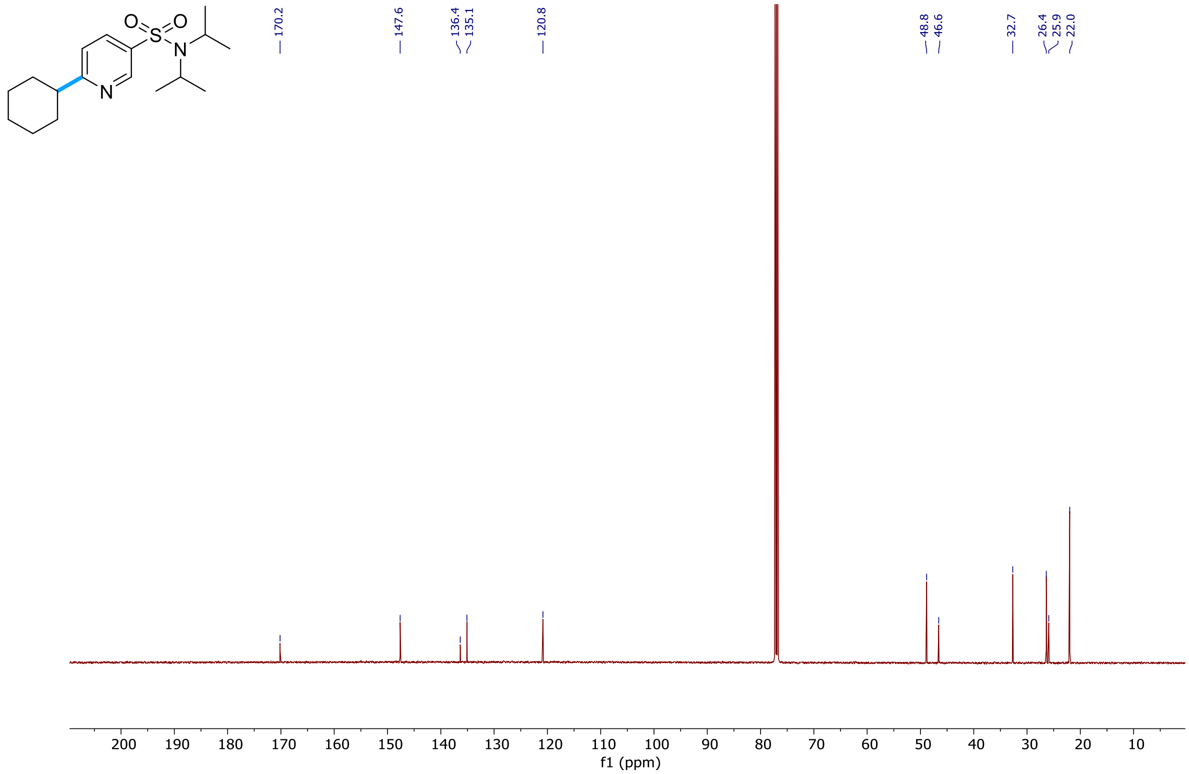

Compound **37** ^{1}H-NMR

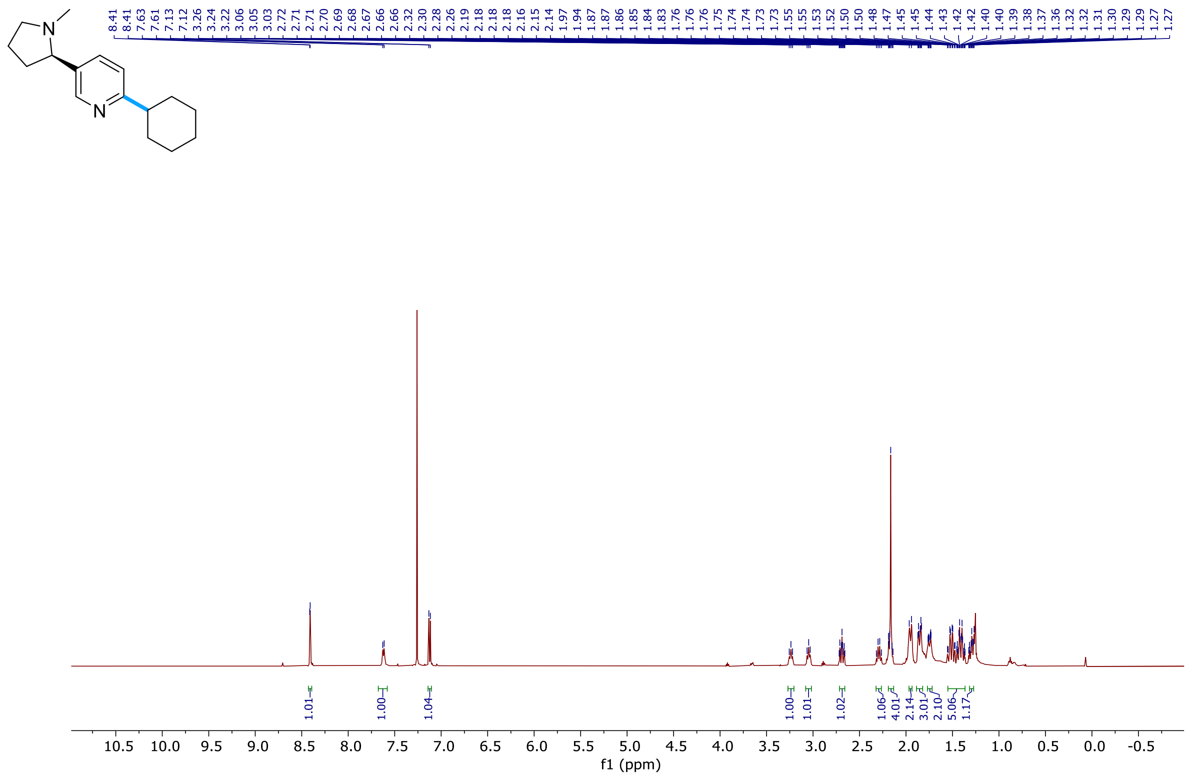

Compound **37** ^{13}C-NMR

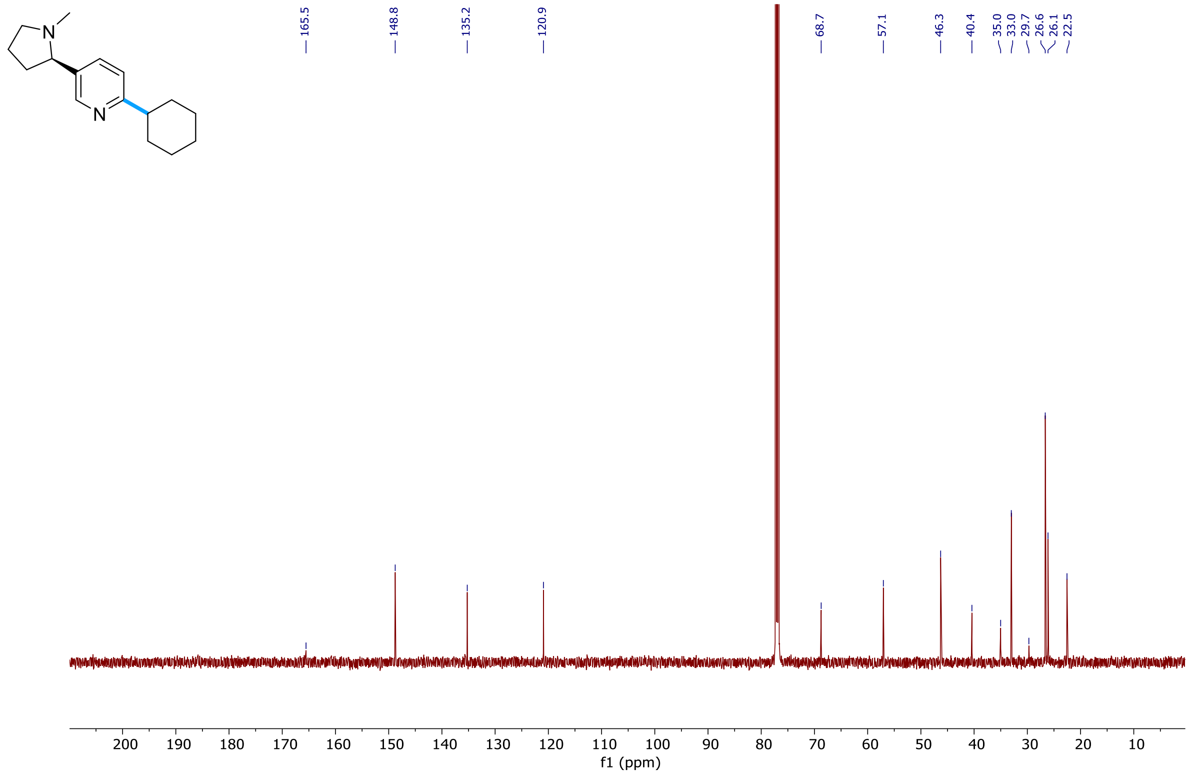

Compound **38a** ^{1}H-NMR

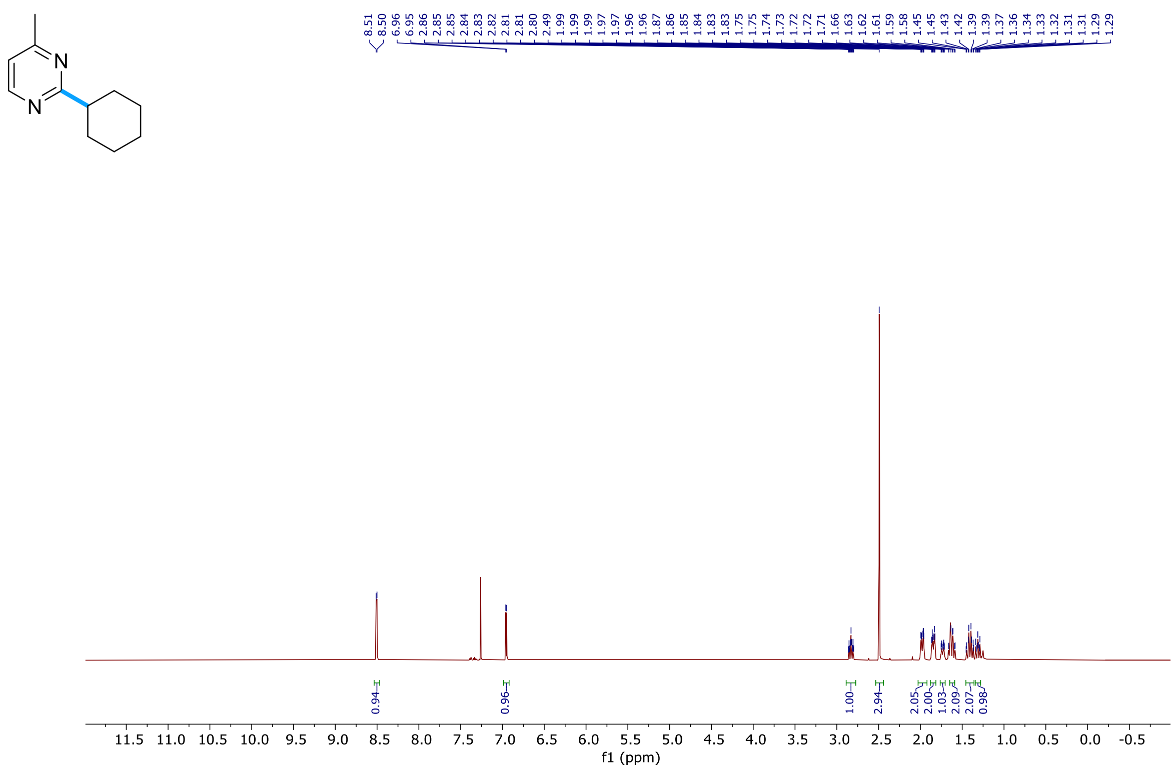

Compound **38a** ^{13}C-NMR

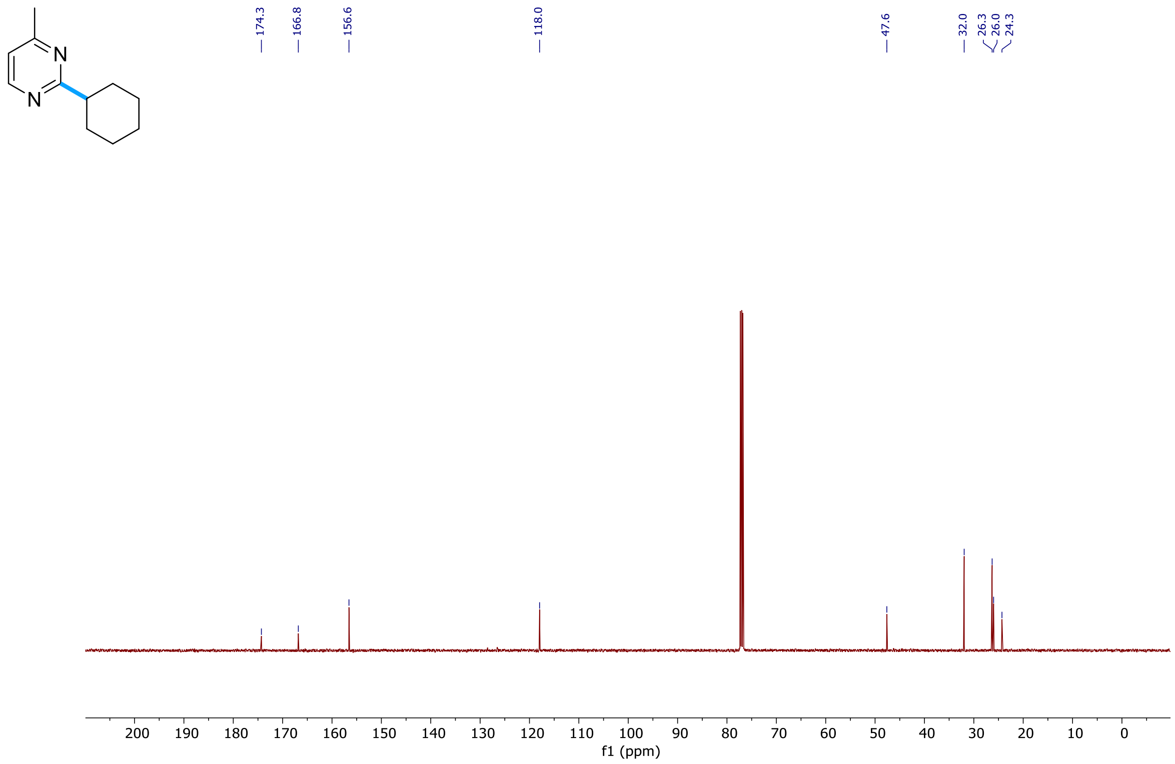

Compound **38b** ^{1}H-NMR

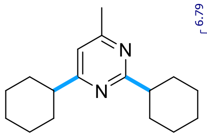

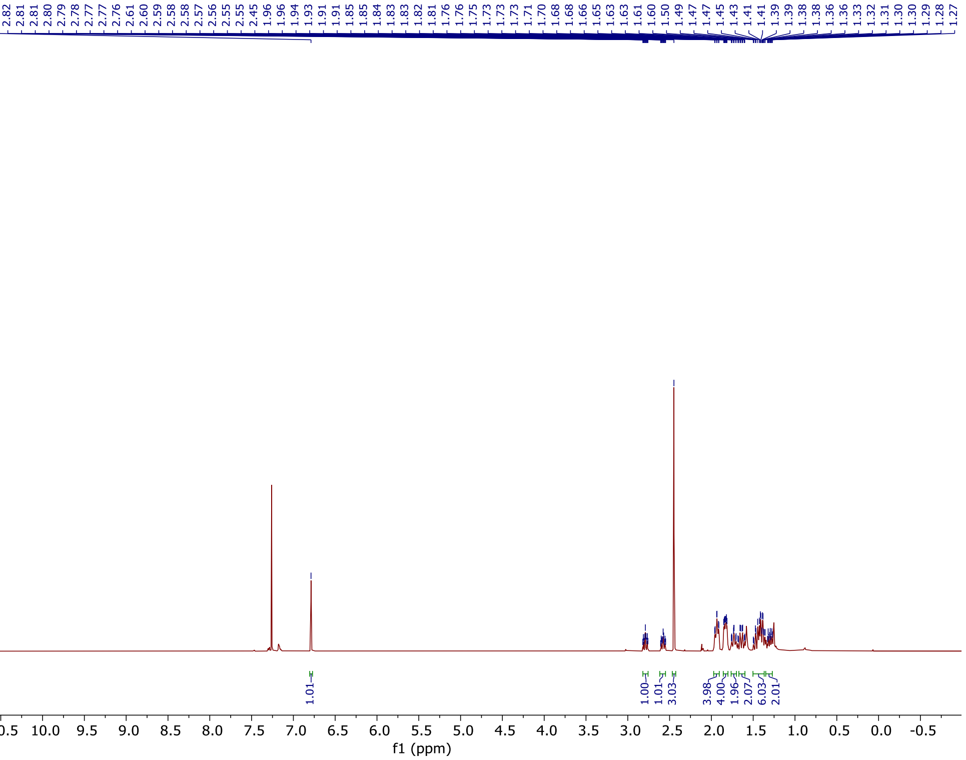

Compound **38b** ^{13}C-NMR

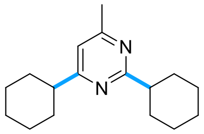

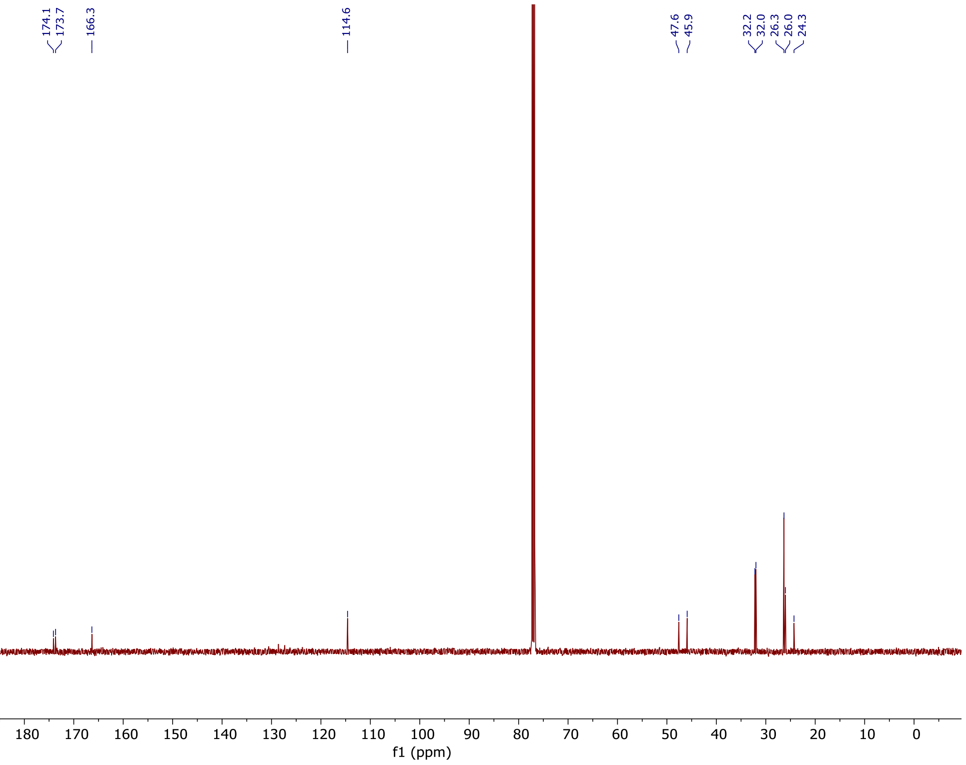

Compound **39a** ^{1}H-NMR

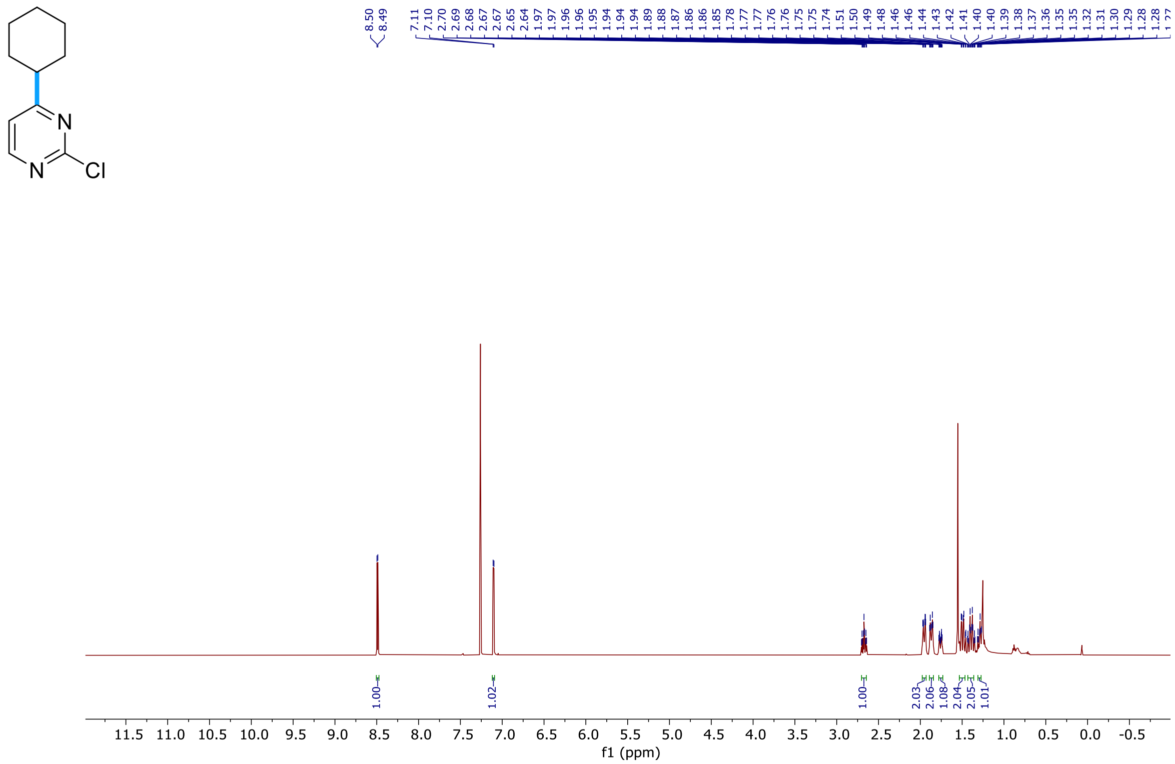

Compound **39a** ^{13}C-NMR

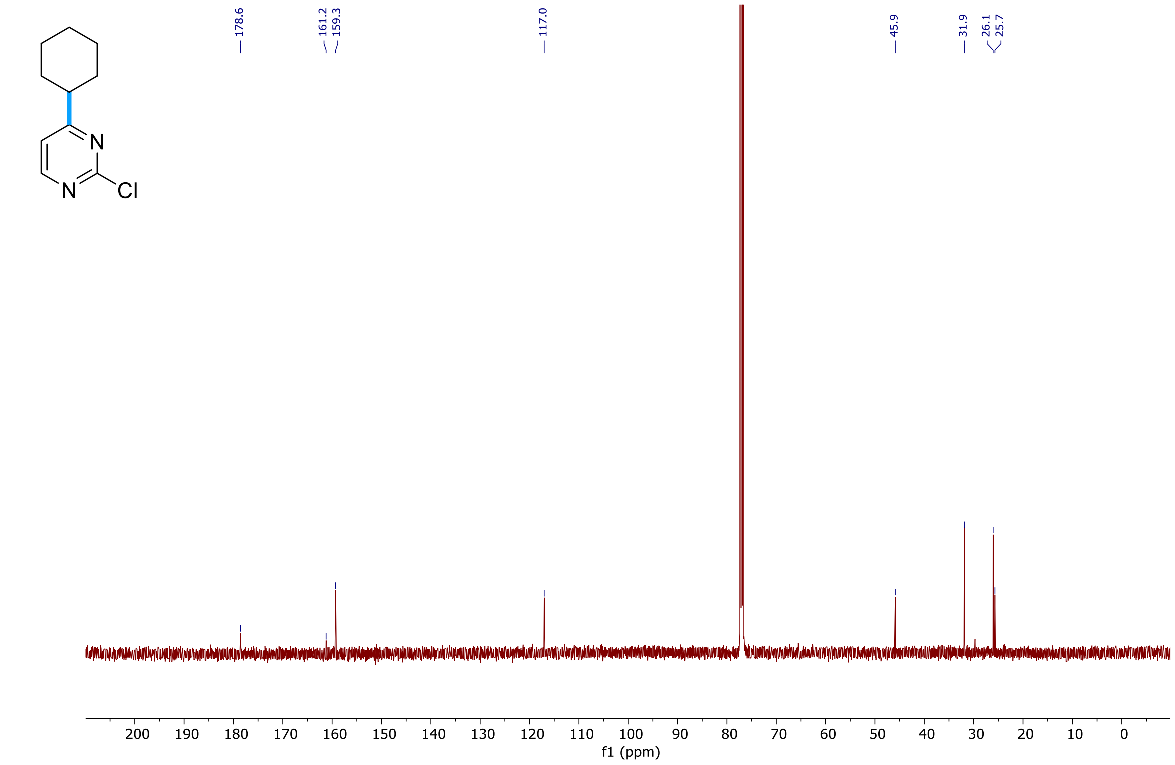

Compound **39b** ^{1}H-NMR

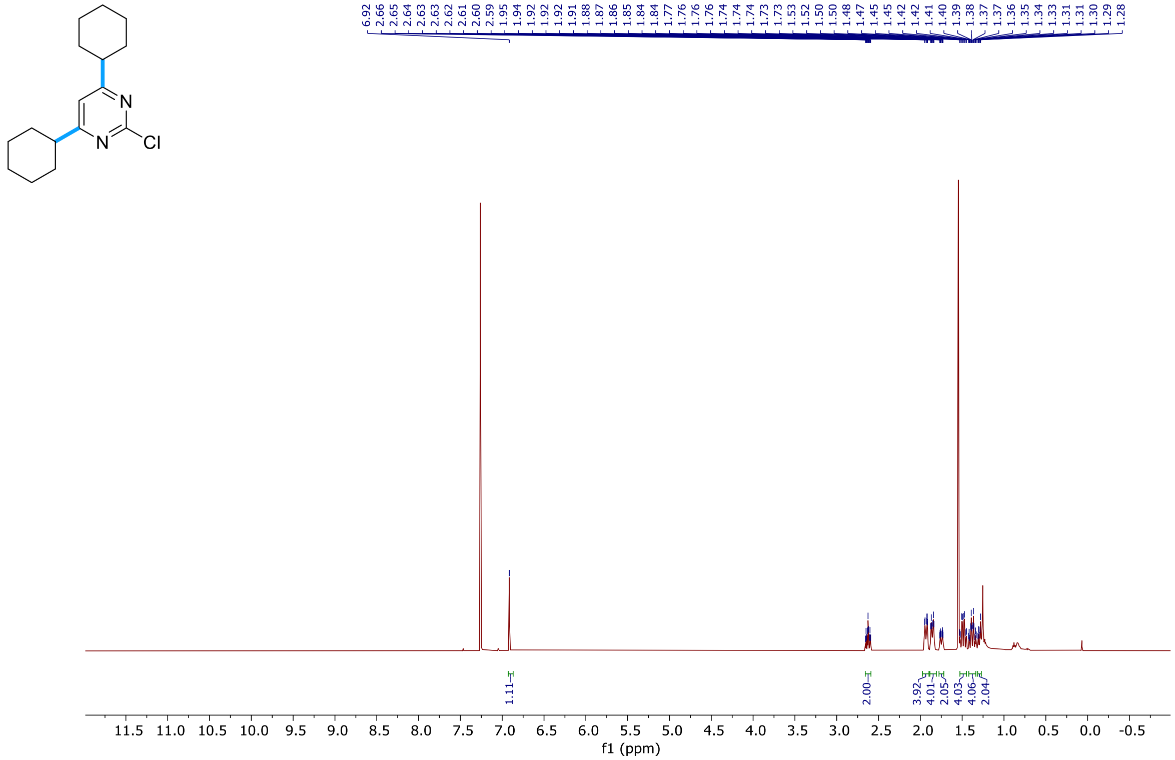

Compound **39b** ^{13}C-NMR

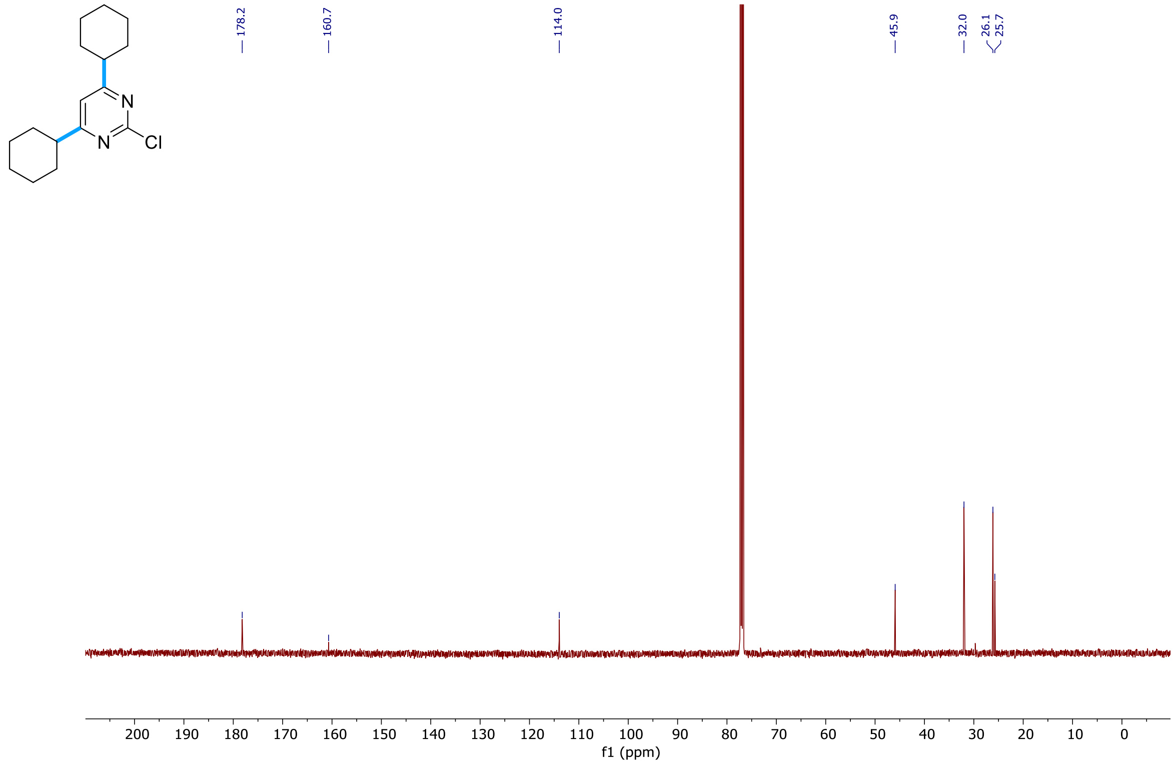

Compound **40** ^{1}H-NMR

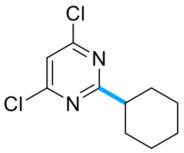

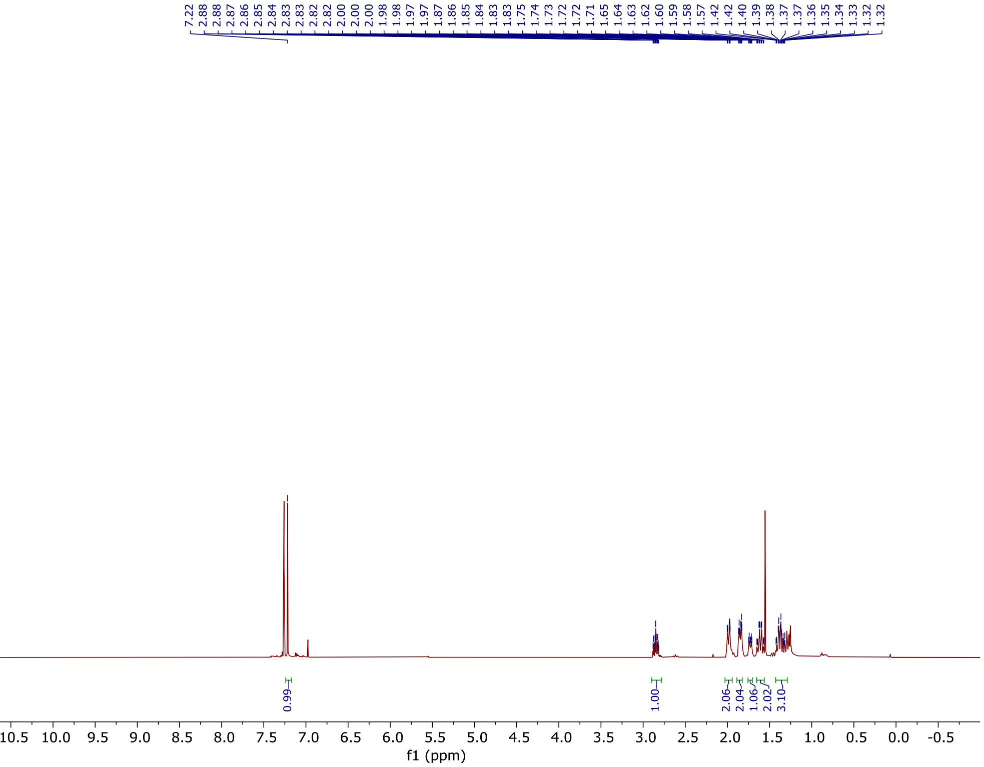

Compound **40** ^{13}C-NMR

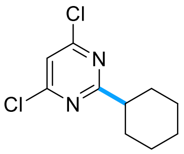

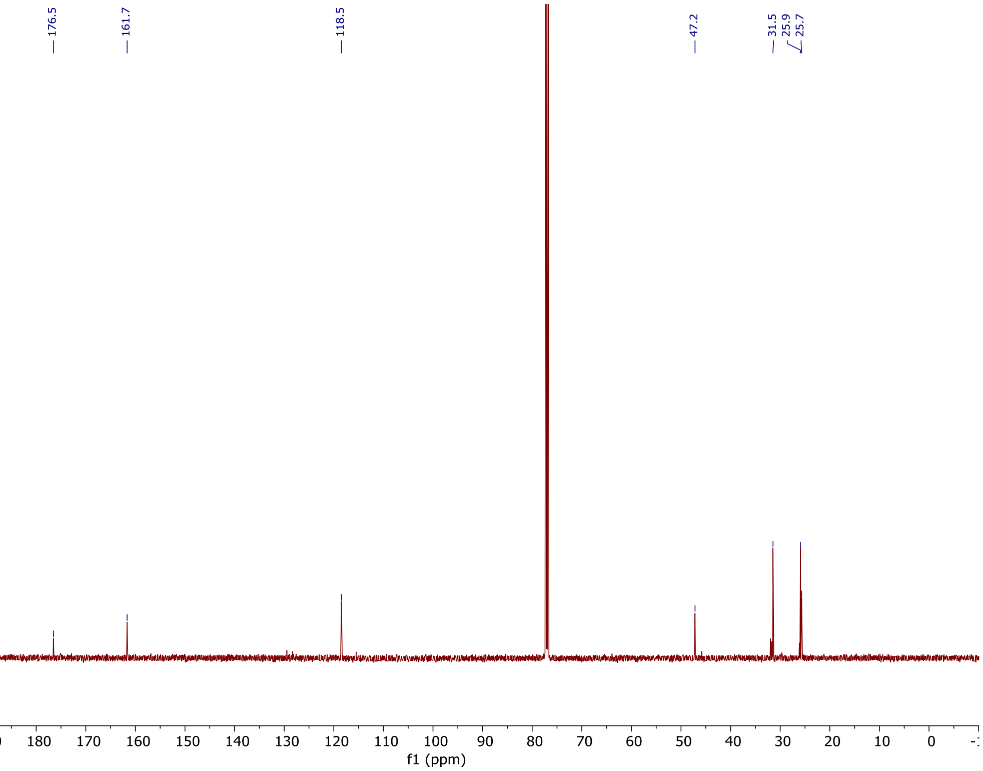

Compound **41** ^{1}H-NMR

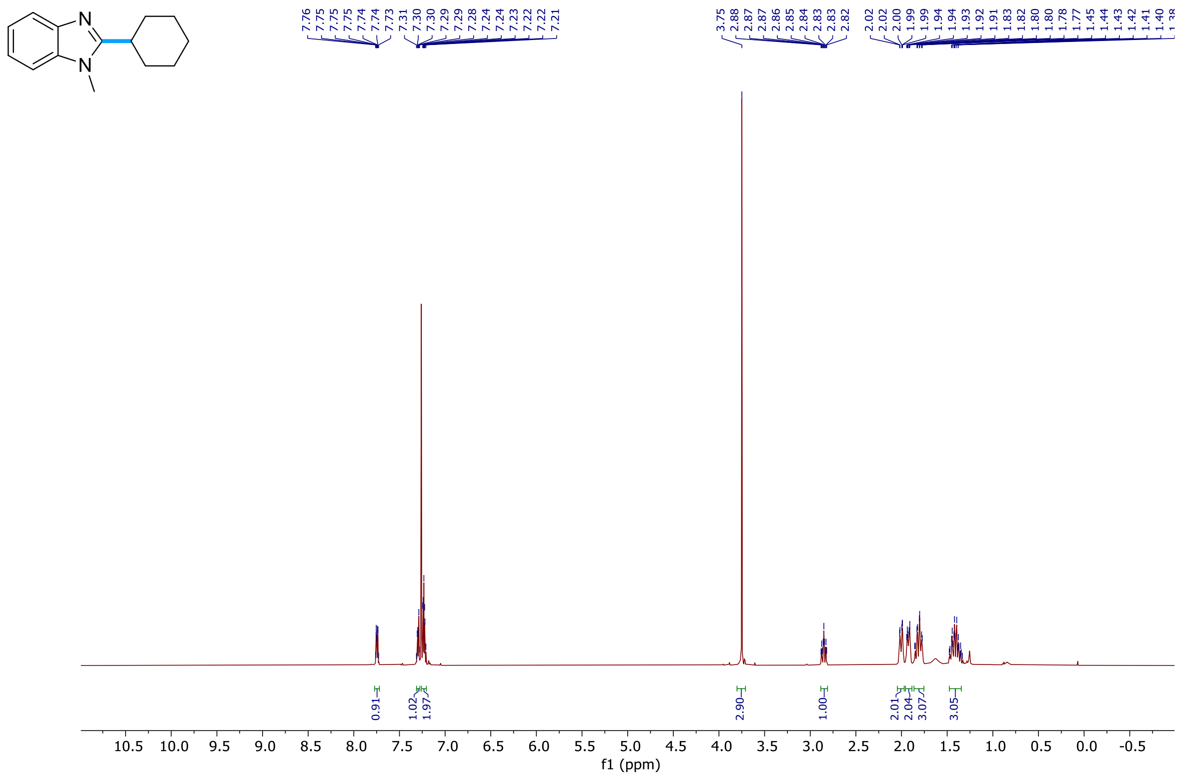

Compound **41** ^{13}C-NMR

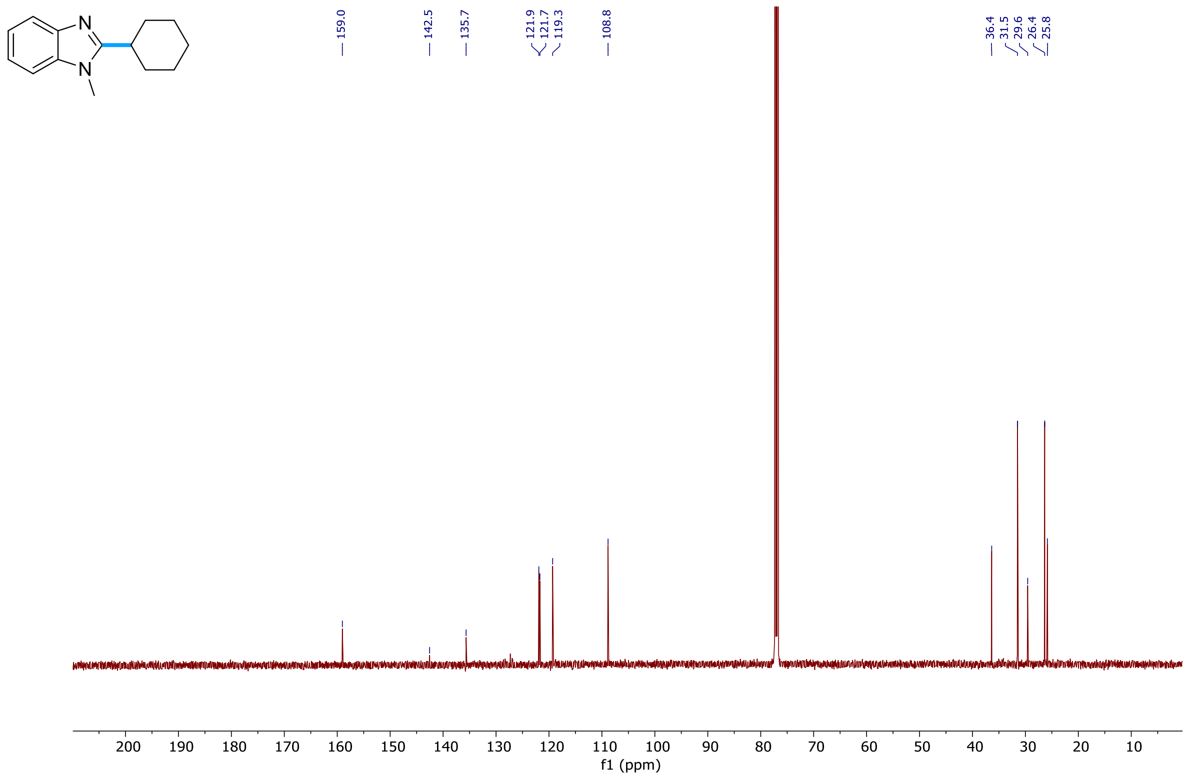

Compound **42** ^{1}H-NMR

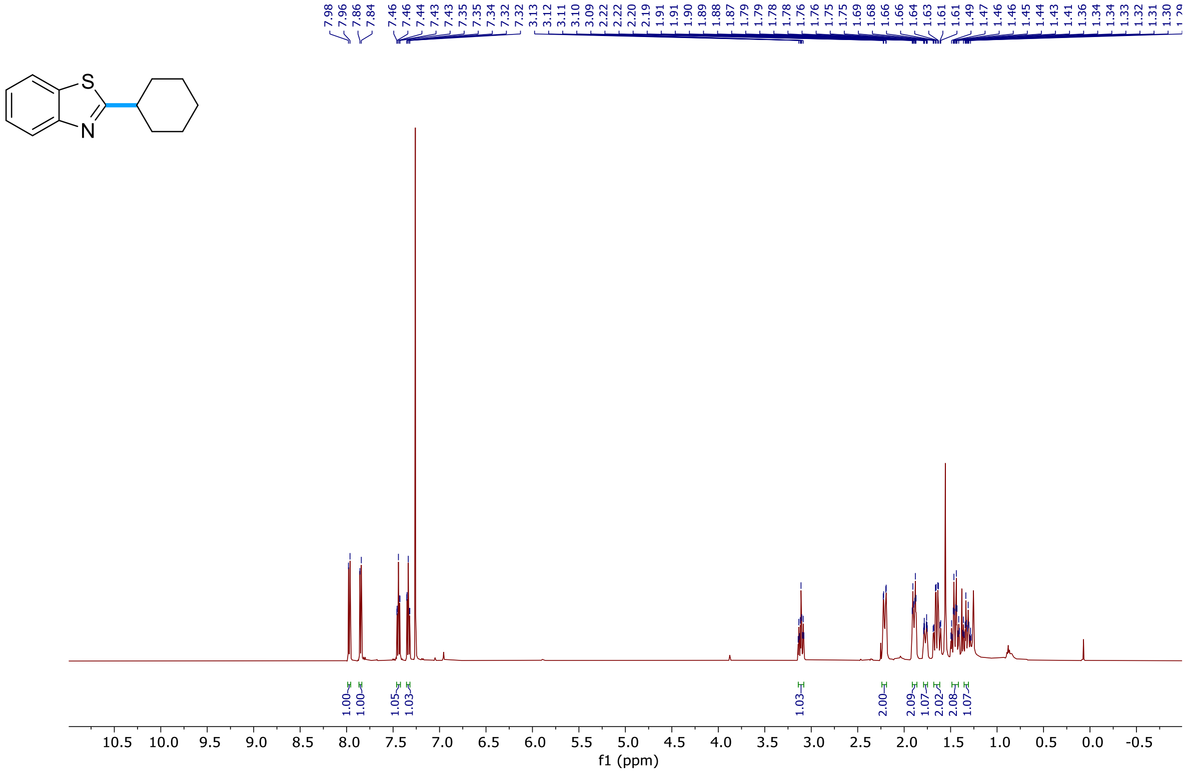

Compound **42** ^{13}C-NMR

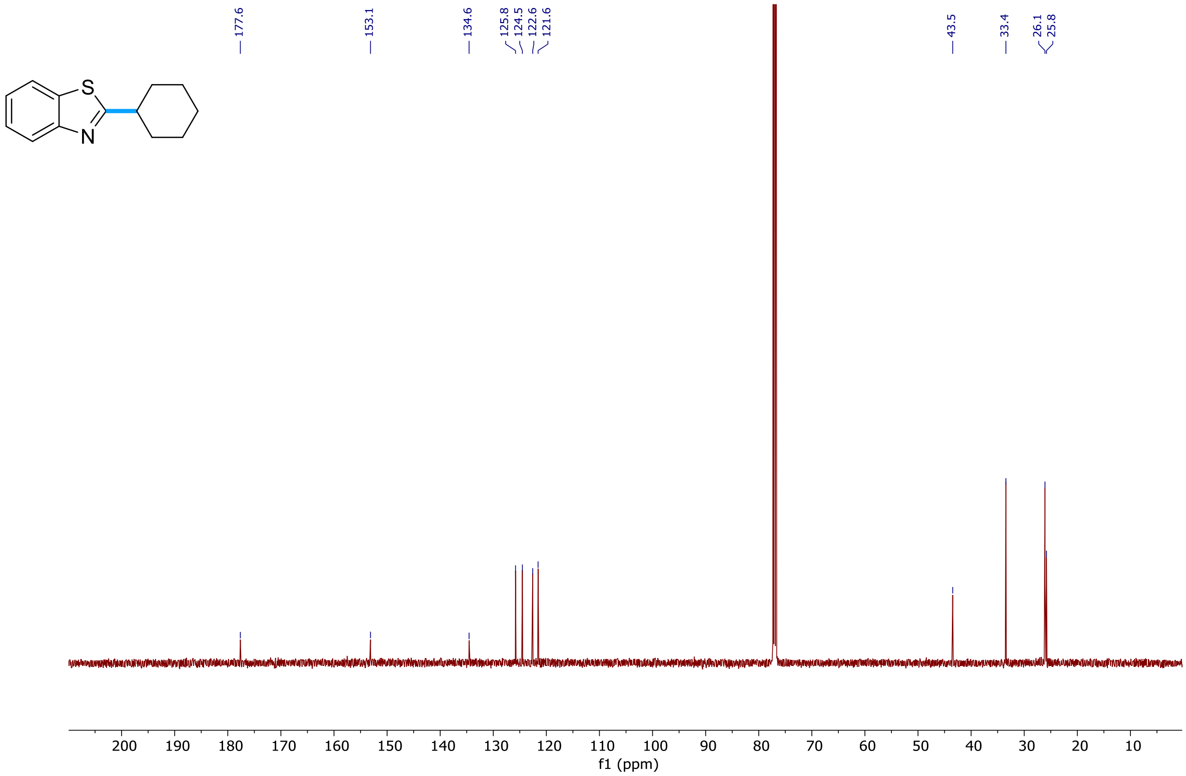

Compound **43** ^{1}H-NMR

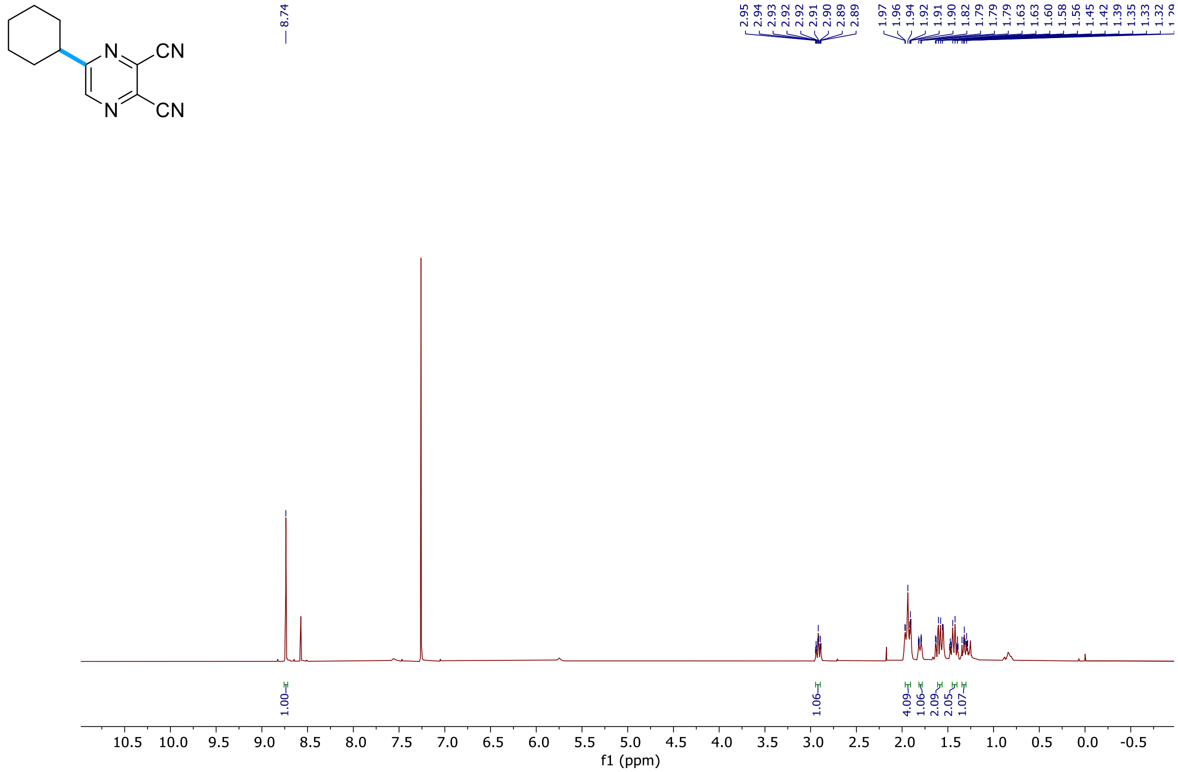

Compound **43** ^{13}C-NMR

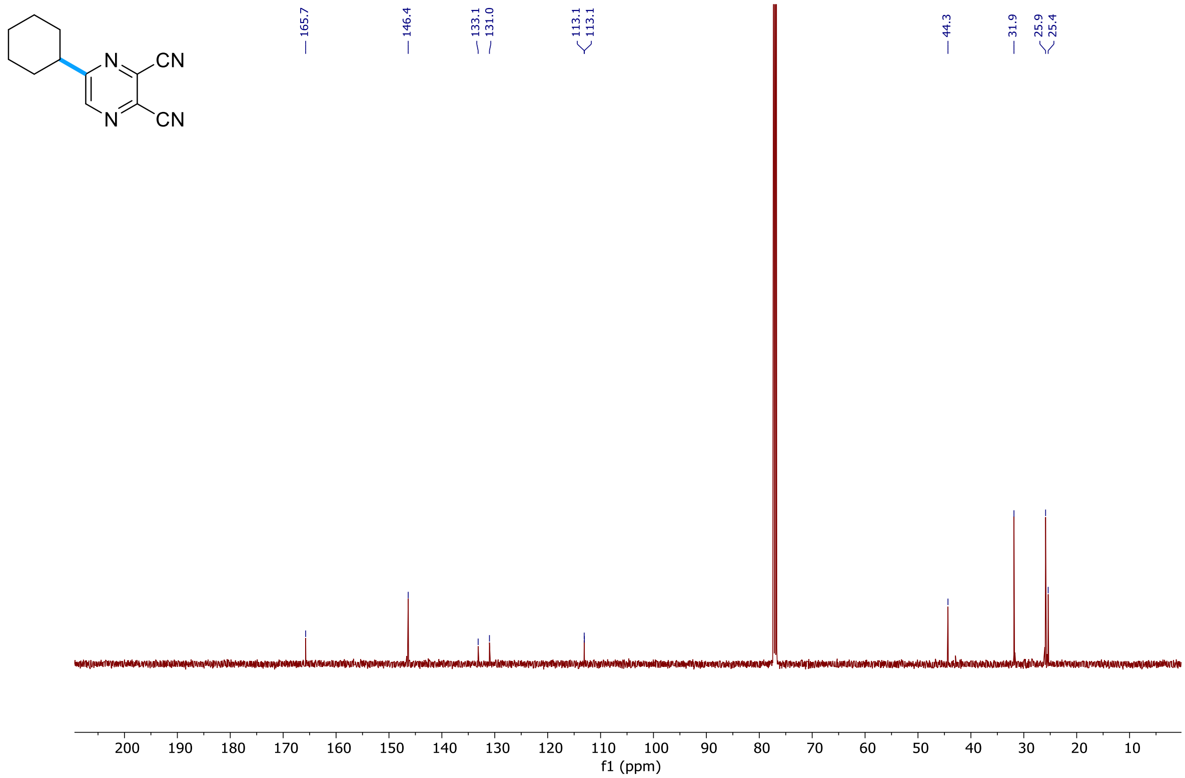

Compound **44** ^{1}H-NMR

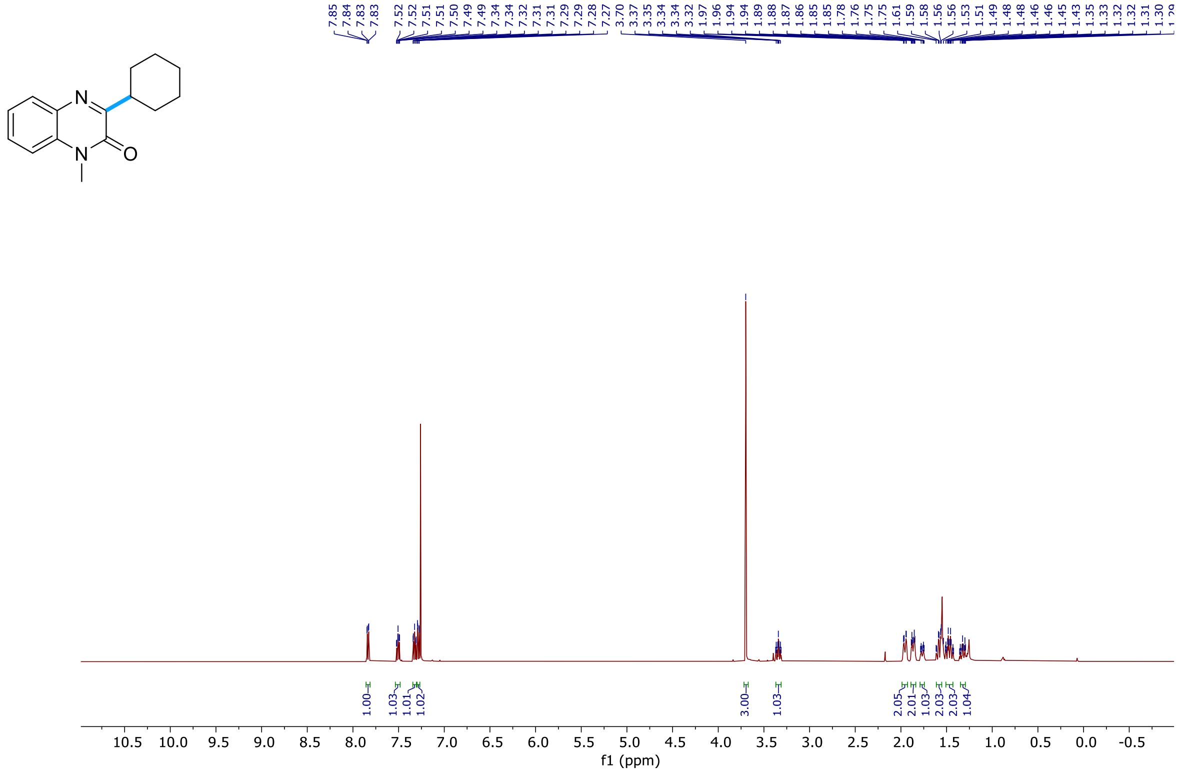

Compound **44** ^{13}C-NMR

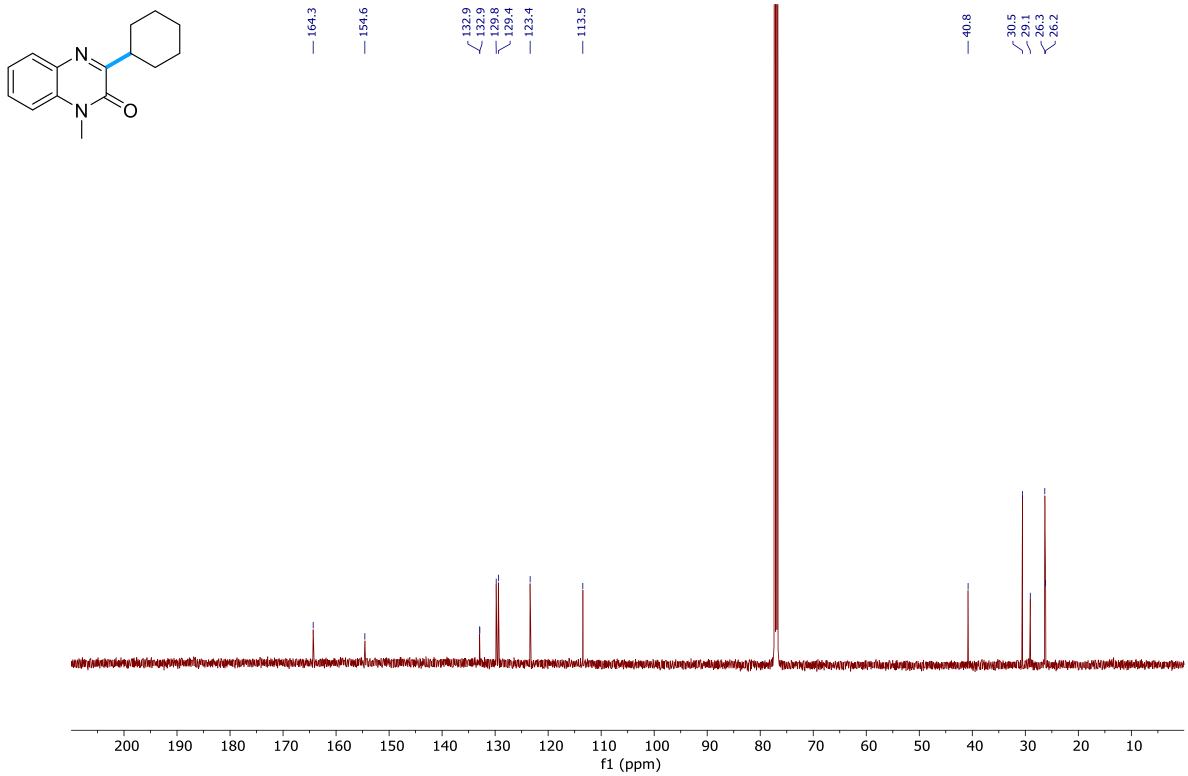

Compound **45** ^{1}H-NMR

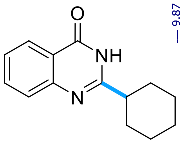

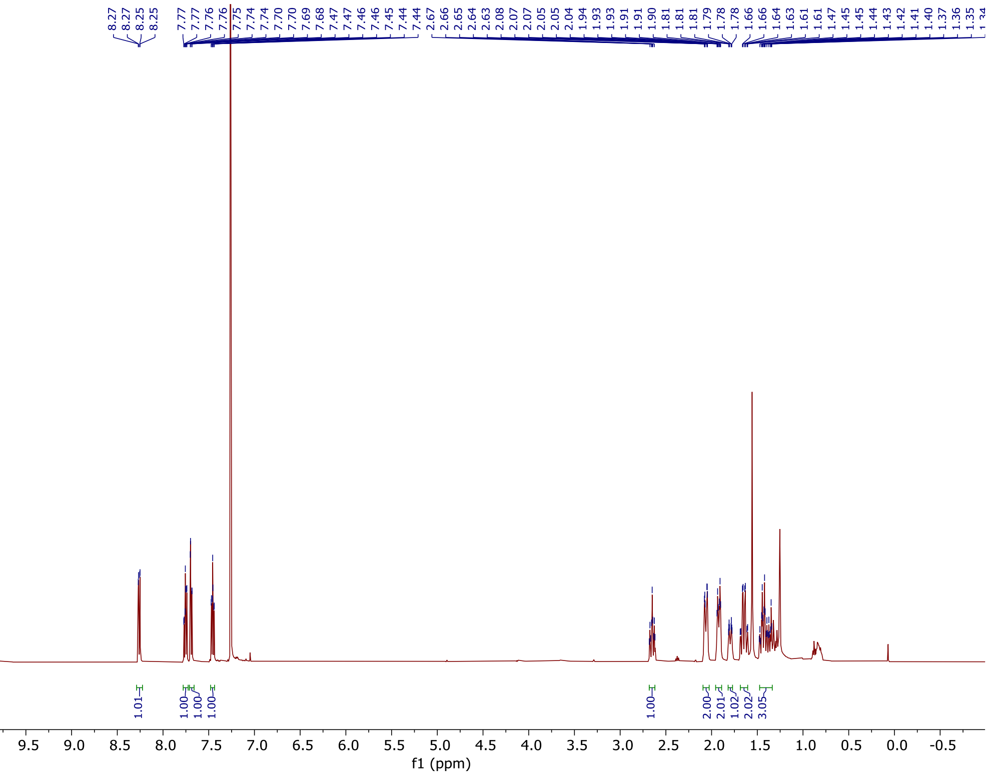

Compound **45** ^{13}C-NMR

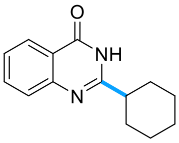

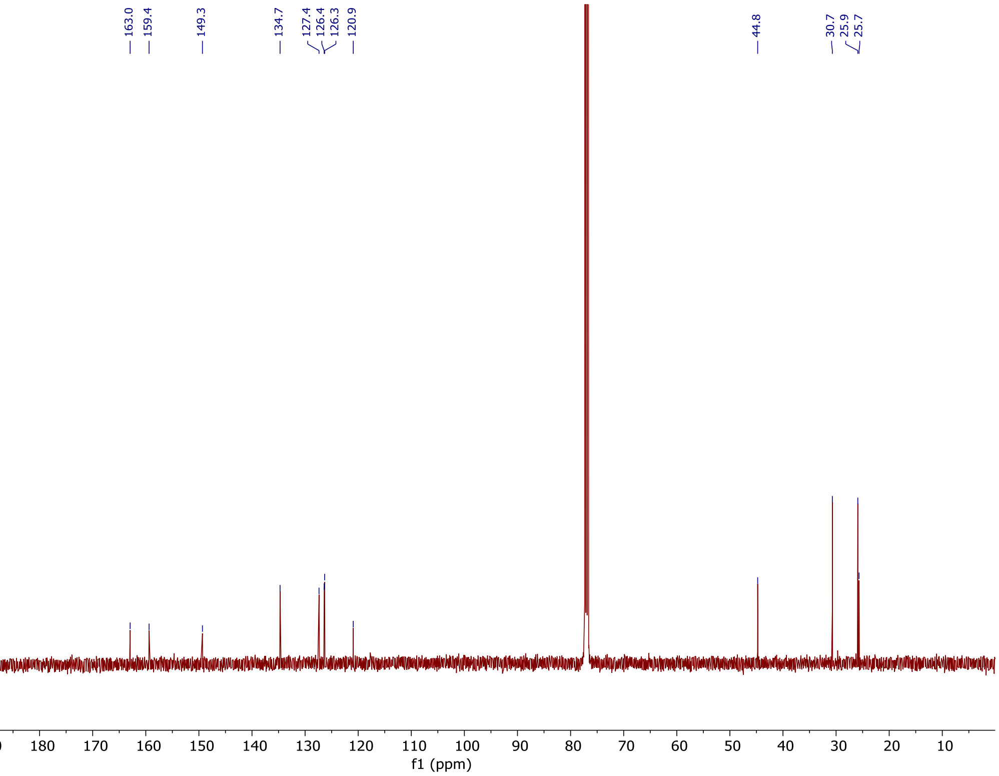

Compound **46** ^{1}H-NMR

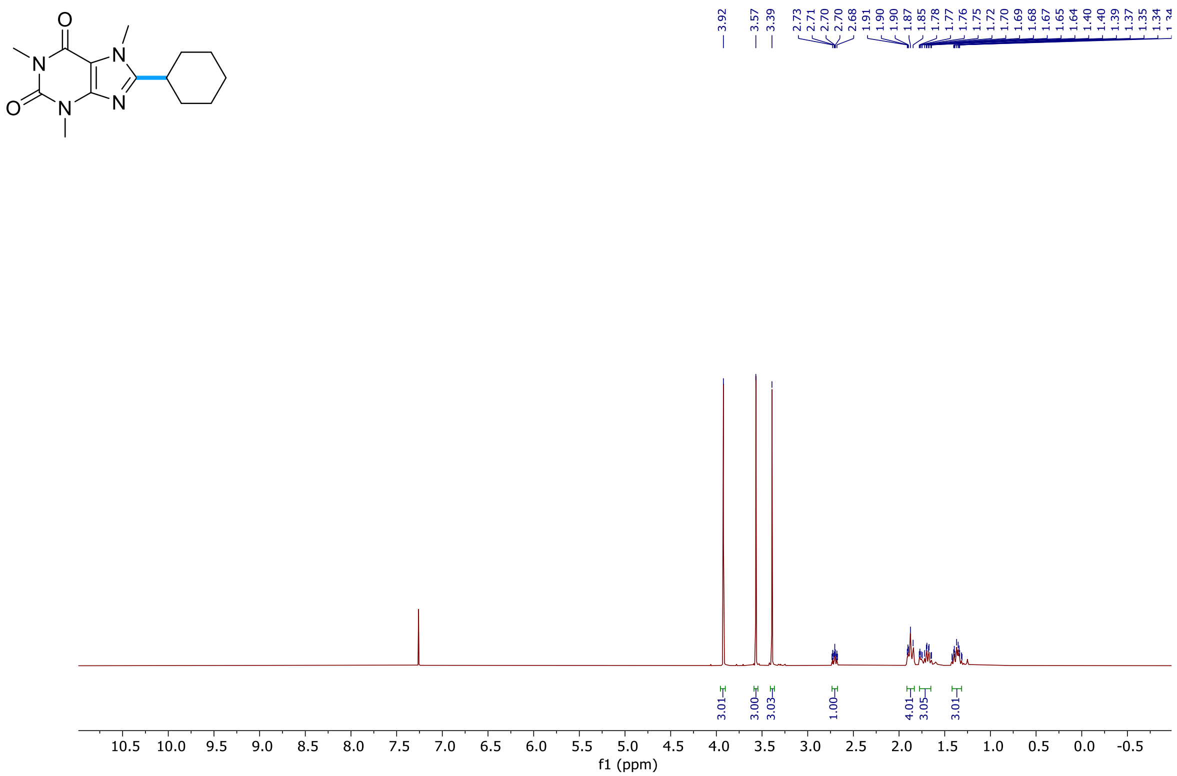

Compound **46** ^{13}C-NMR

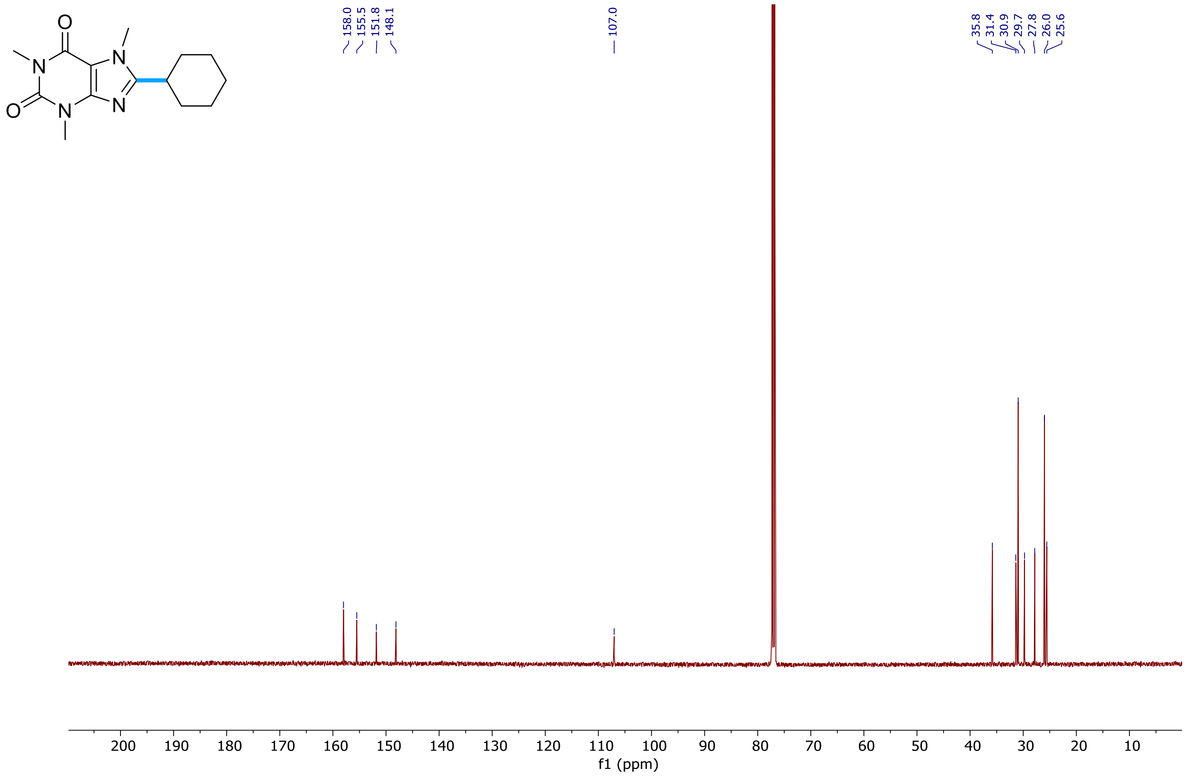

Compound **47** ^{1}H-NMR

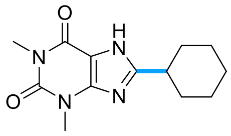

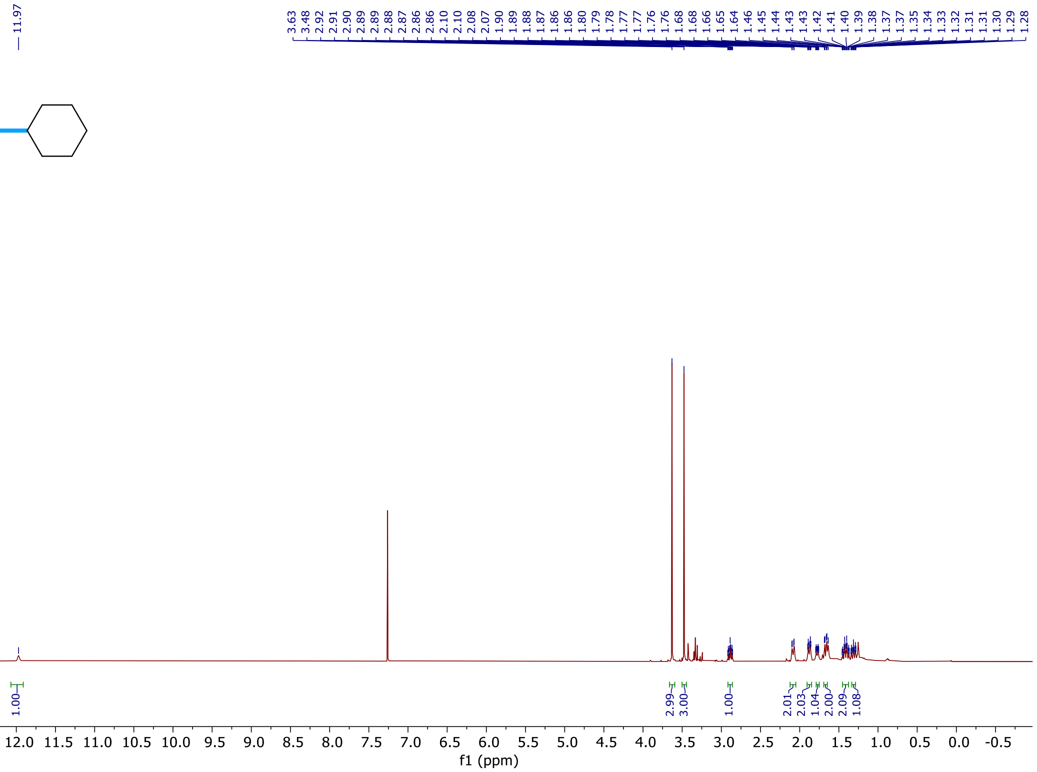

Compound **47** ^{1}H-NMR

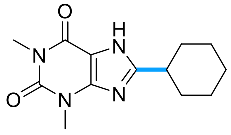

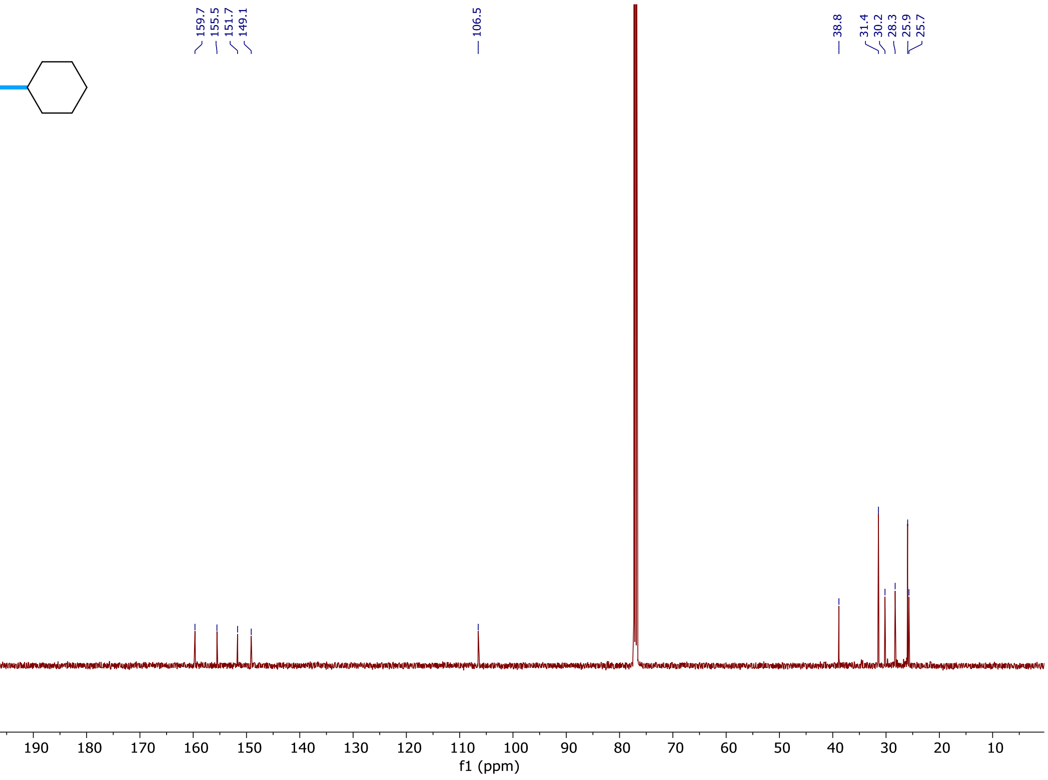

Compound **48** ^{1}H-NMR

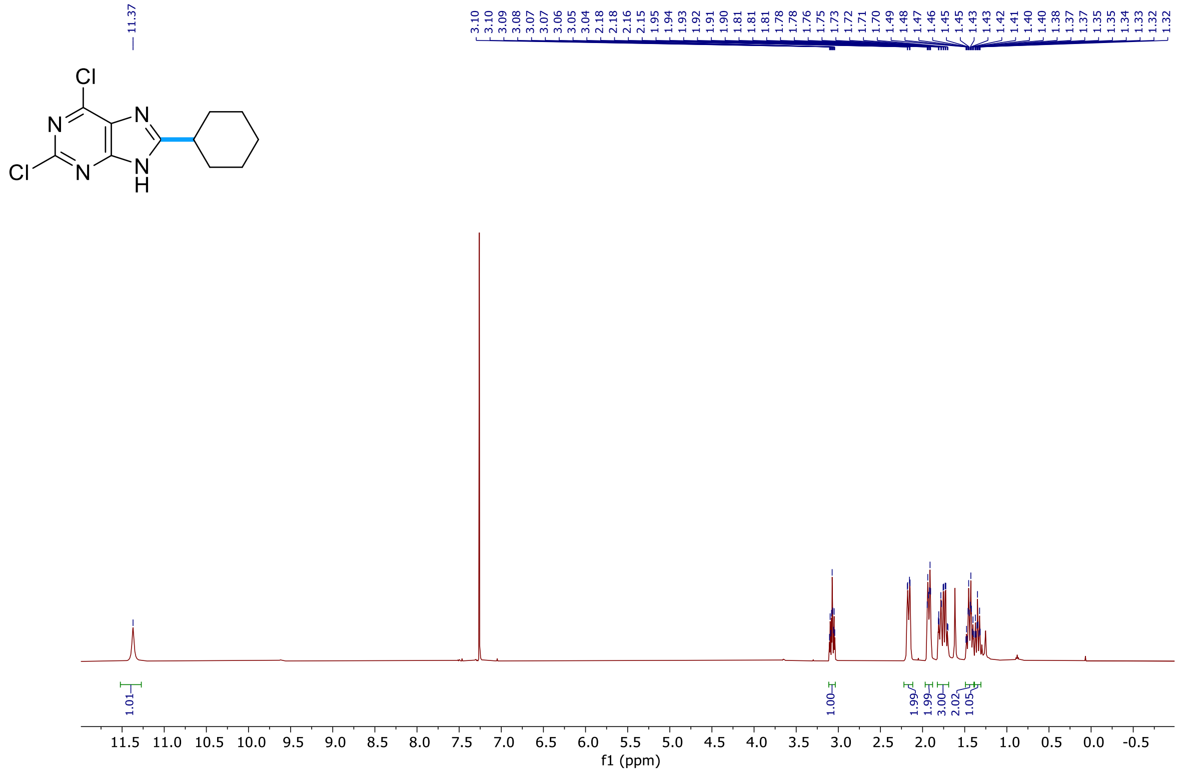

Compound **48** ^{13}C-NMR

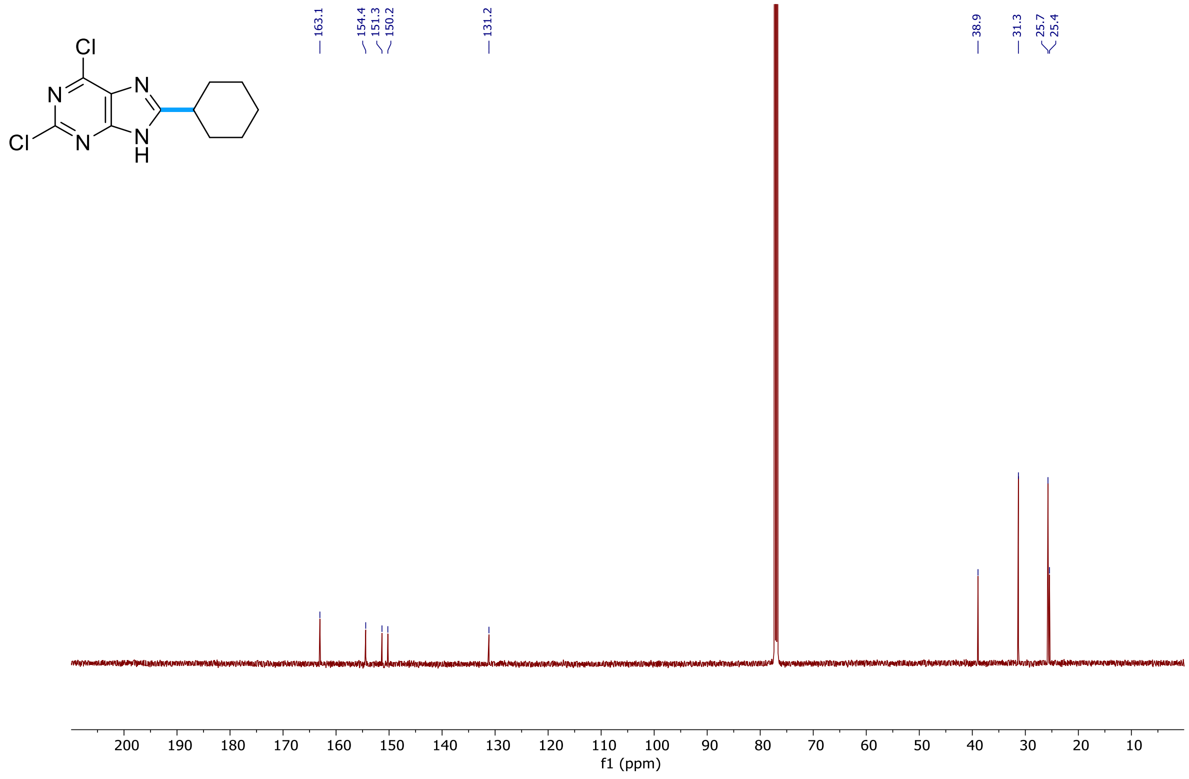

Compound **49** ^{1}H-NMR

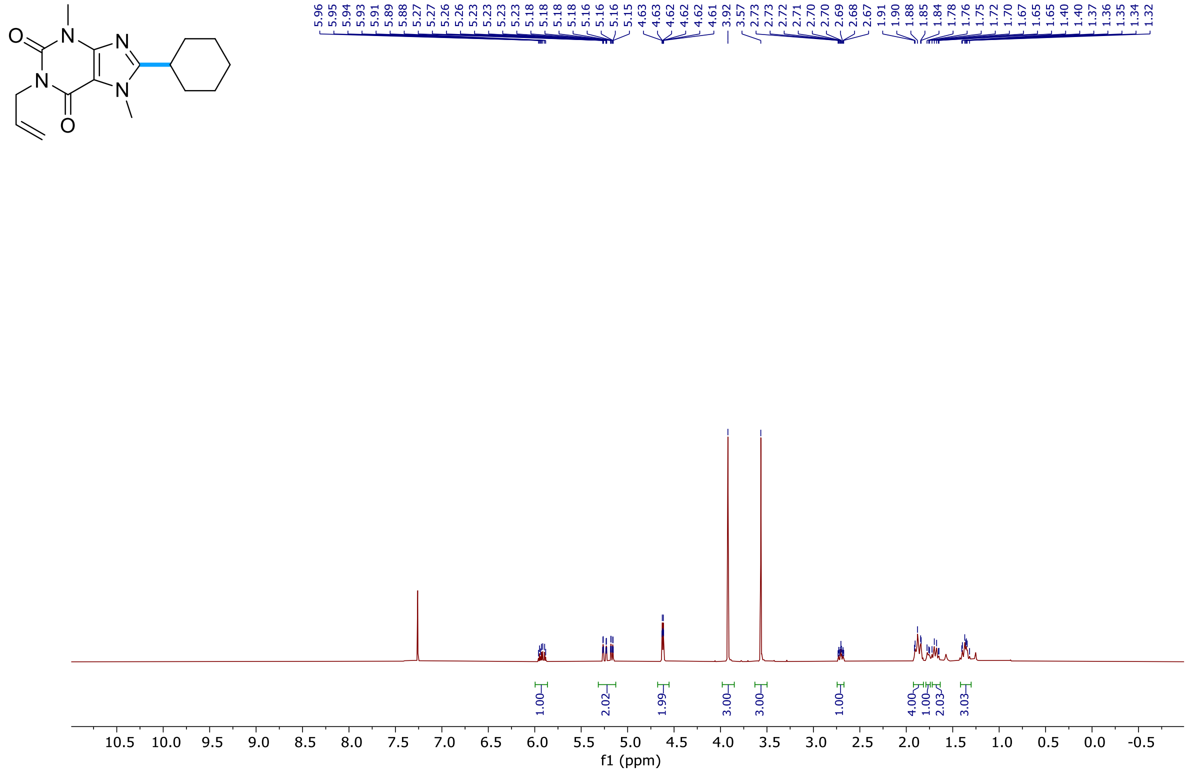

Compound **49** ^{13}C-NMR

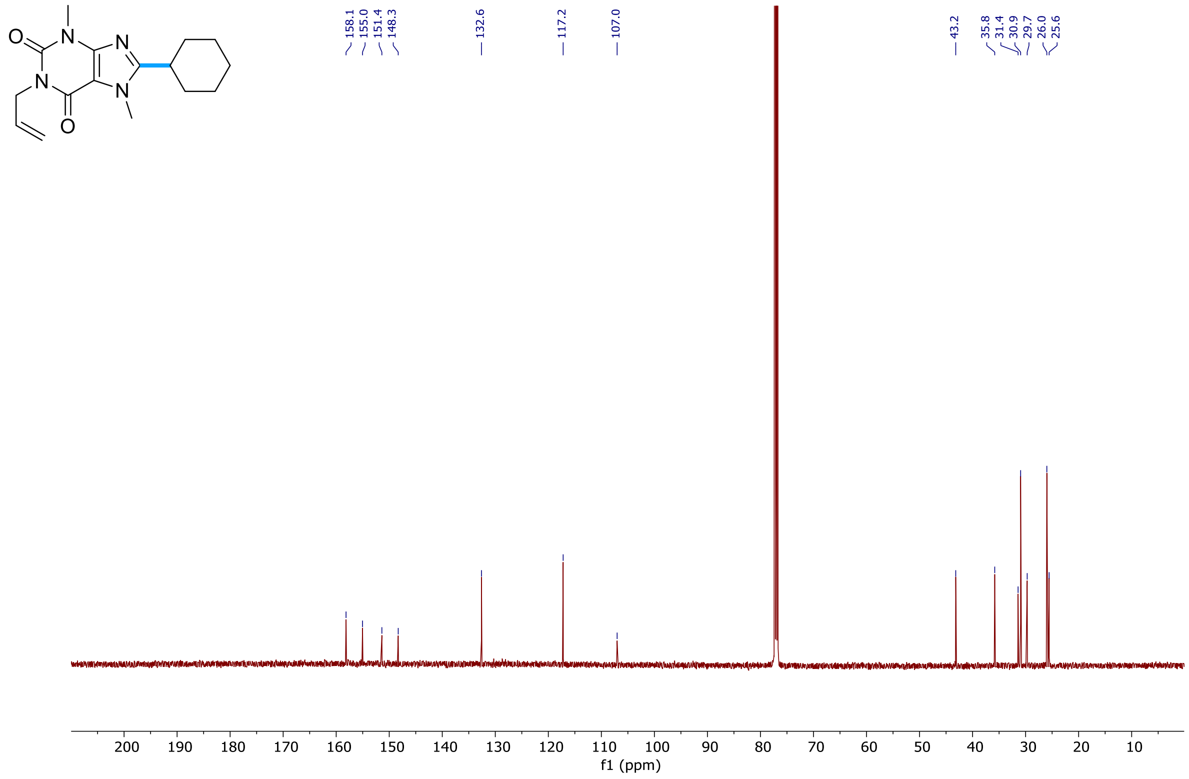

Compound **50** ^{1}H-NMR

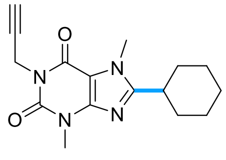

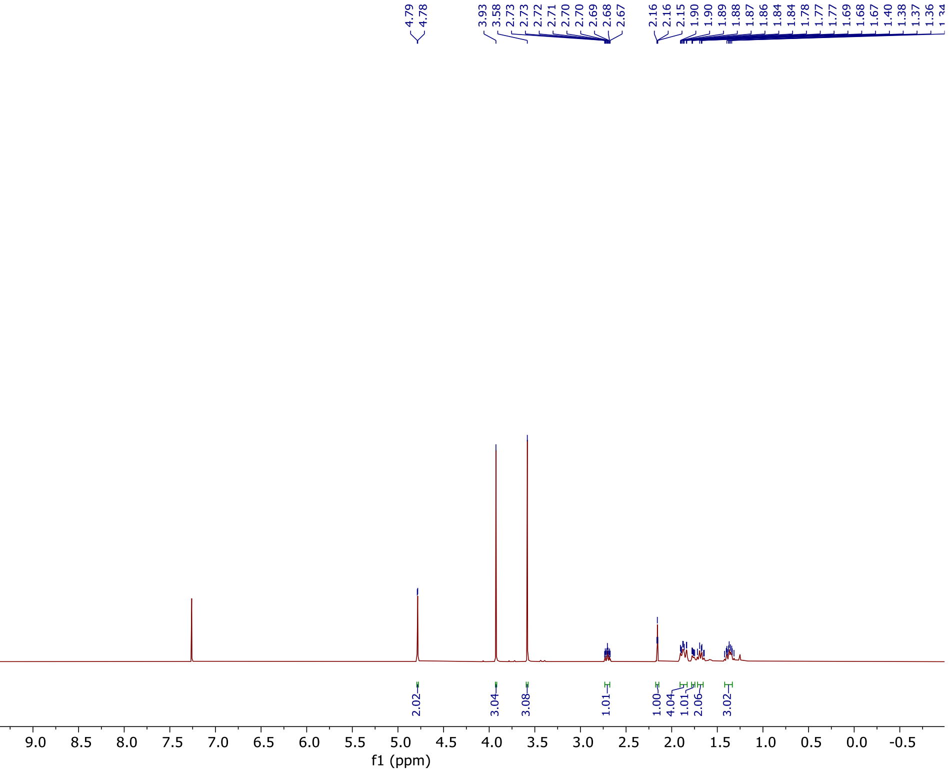

Compound **50** ^{13}C-NMR

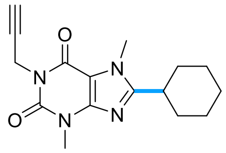

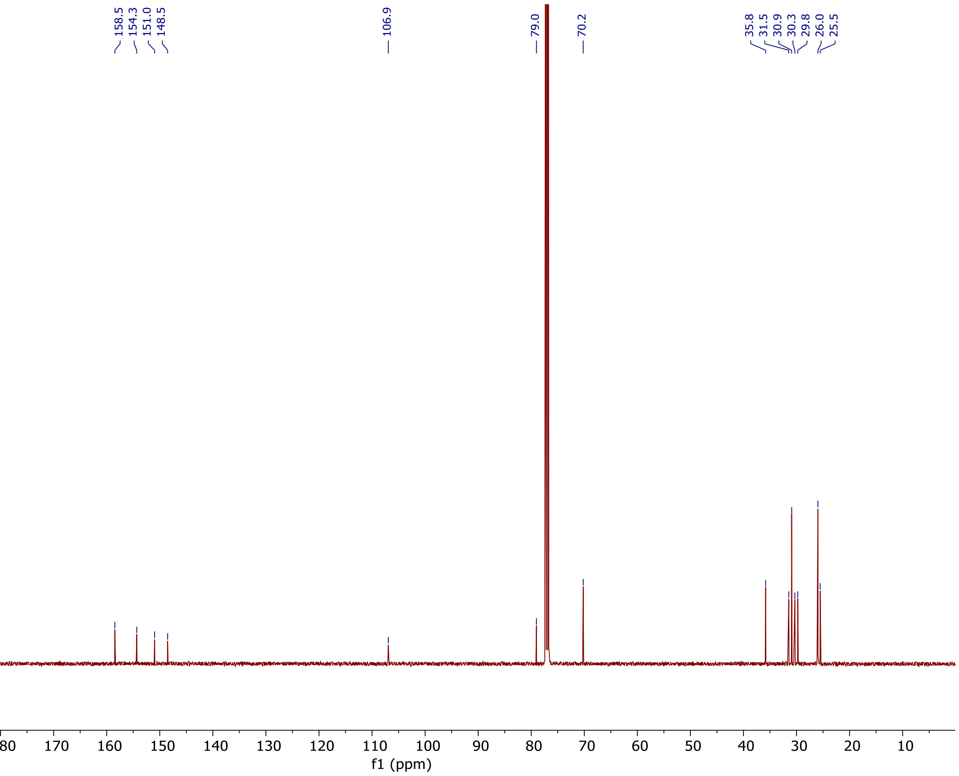

Compound **51** ^{1}H-NMR

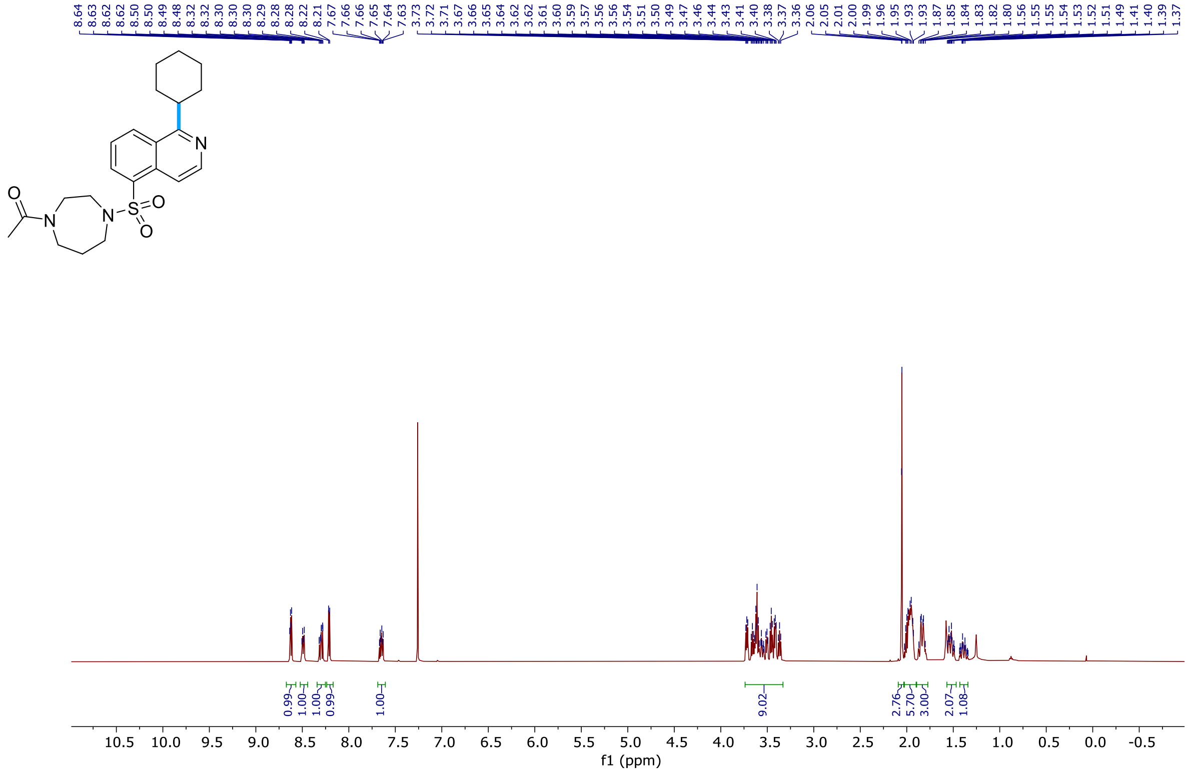

Compound **51** ^{13}C-NMR

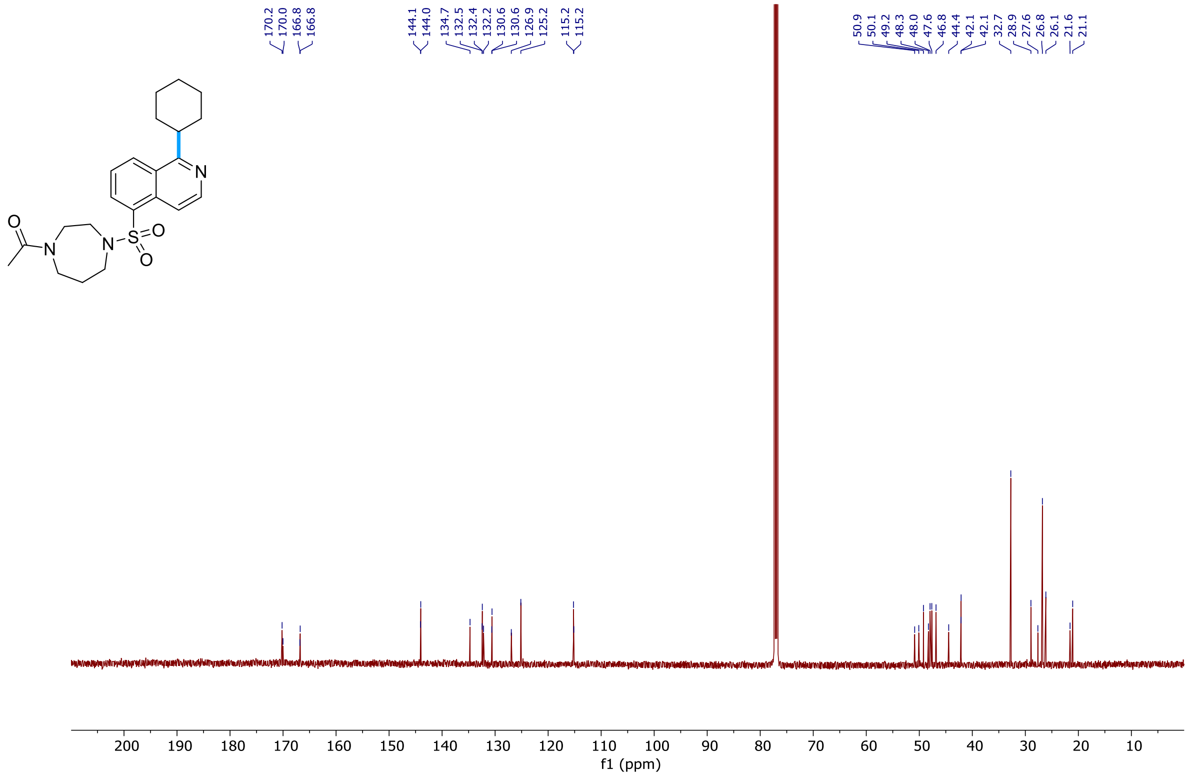

Compound **52** ^{1}H-NMR

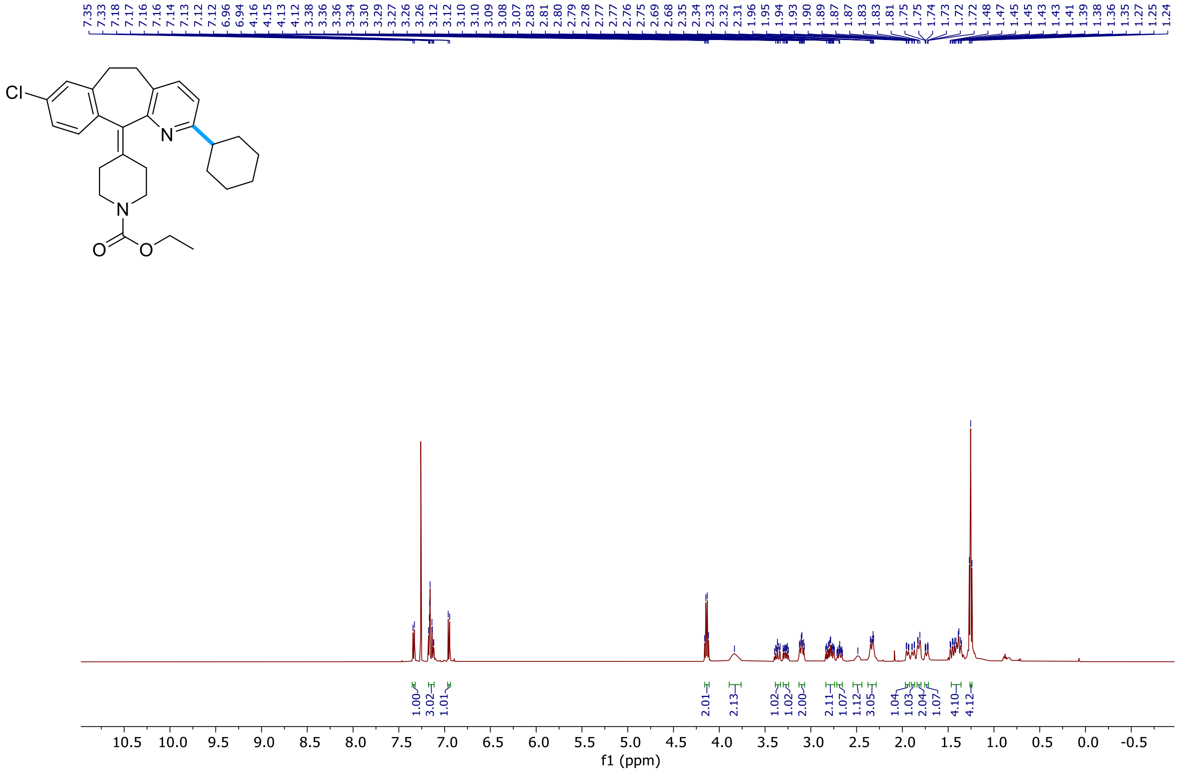

Compound **52** ^{13}C-NMR

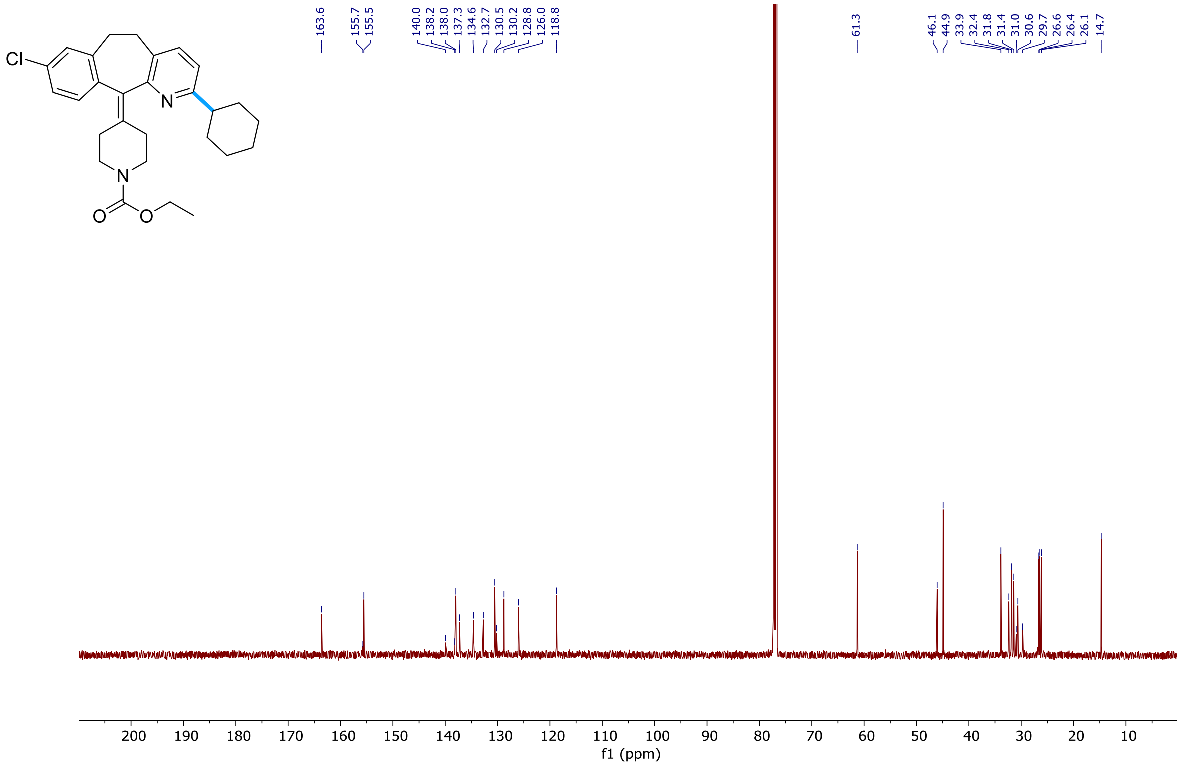

Compound **53** ^{1}H-NMR

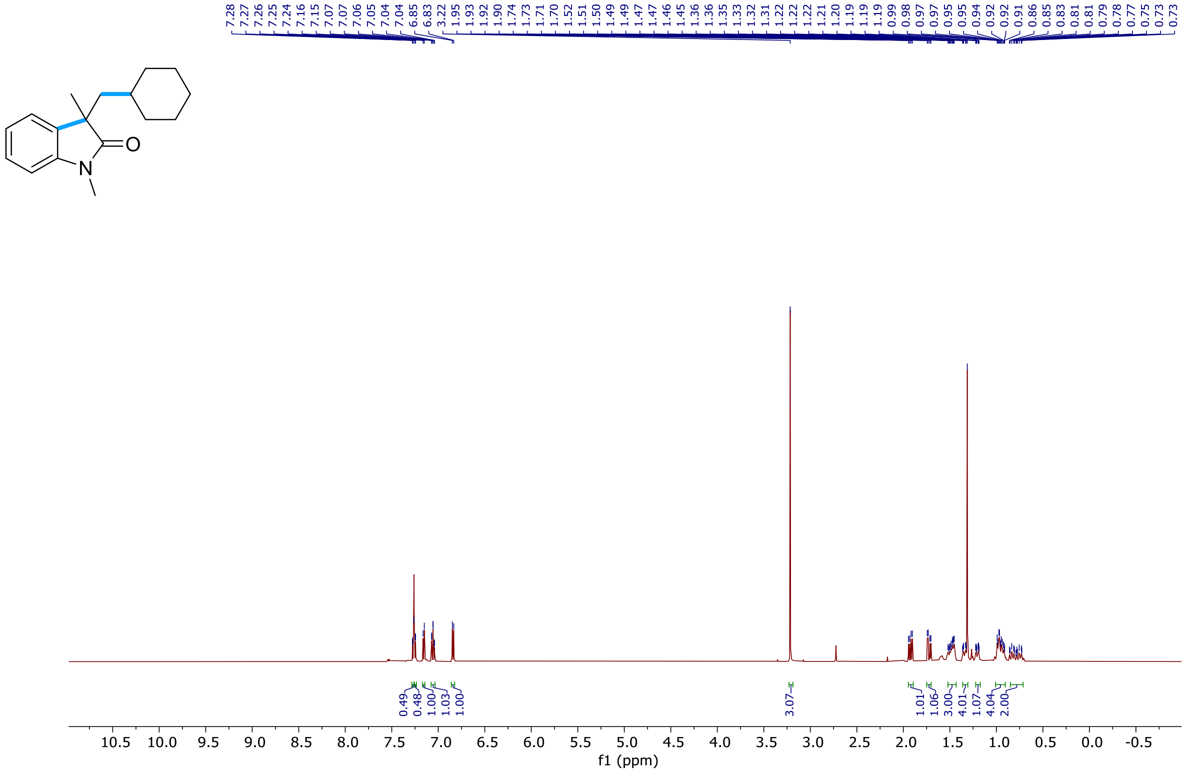

Compound **53** ^{13}C-NMR

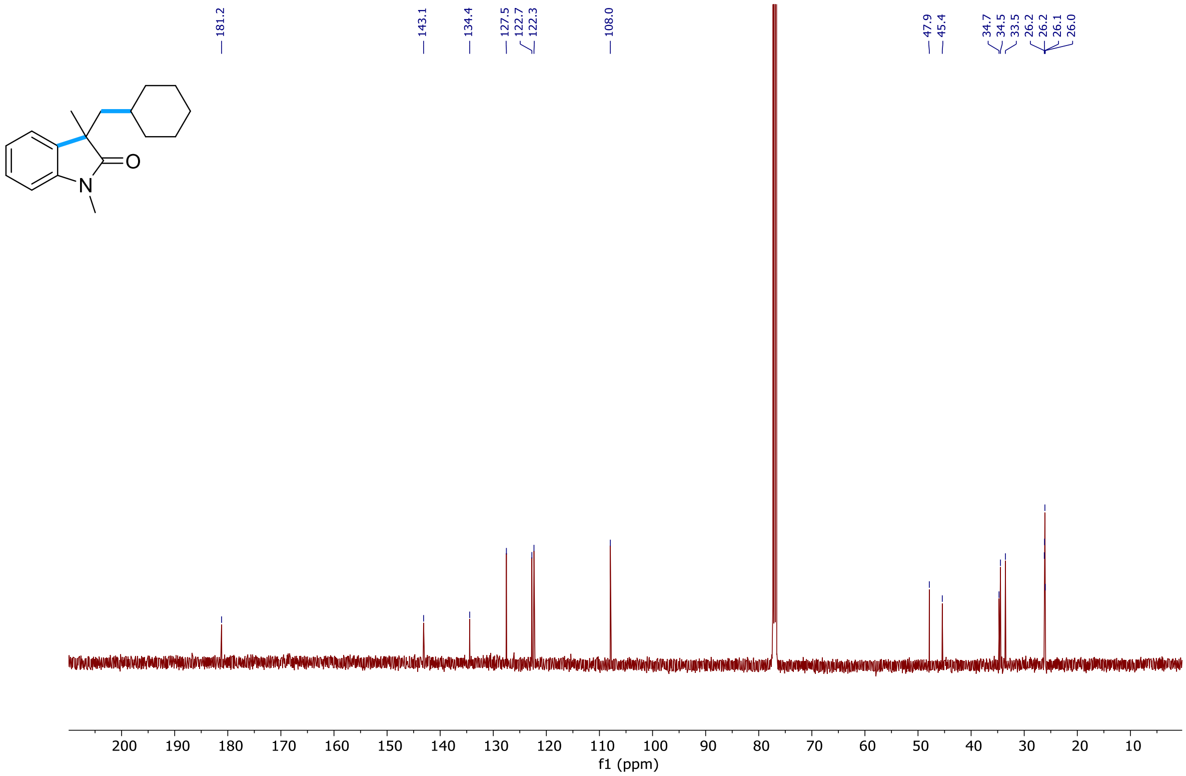

Compound **3-d₁₁** ¹H-NMR

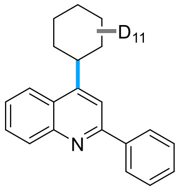

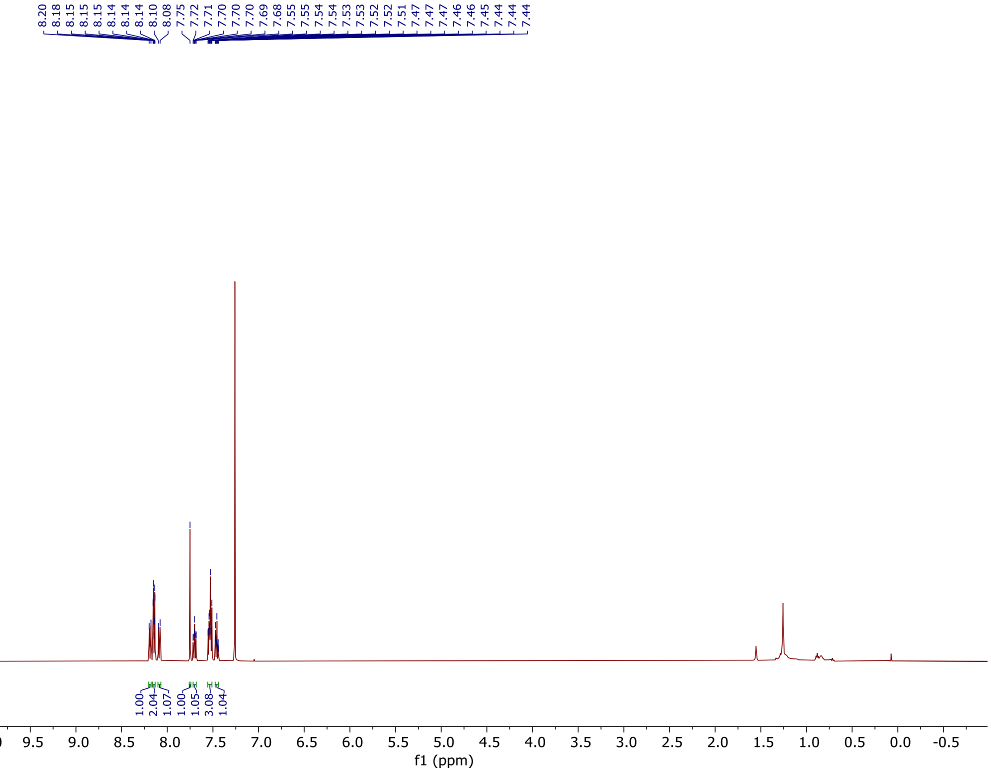

Compound **3-d₁₁** ¹³C-NMR

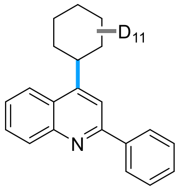

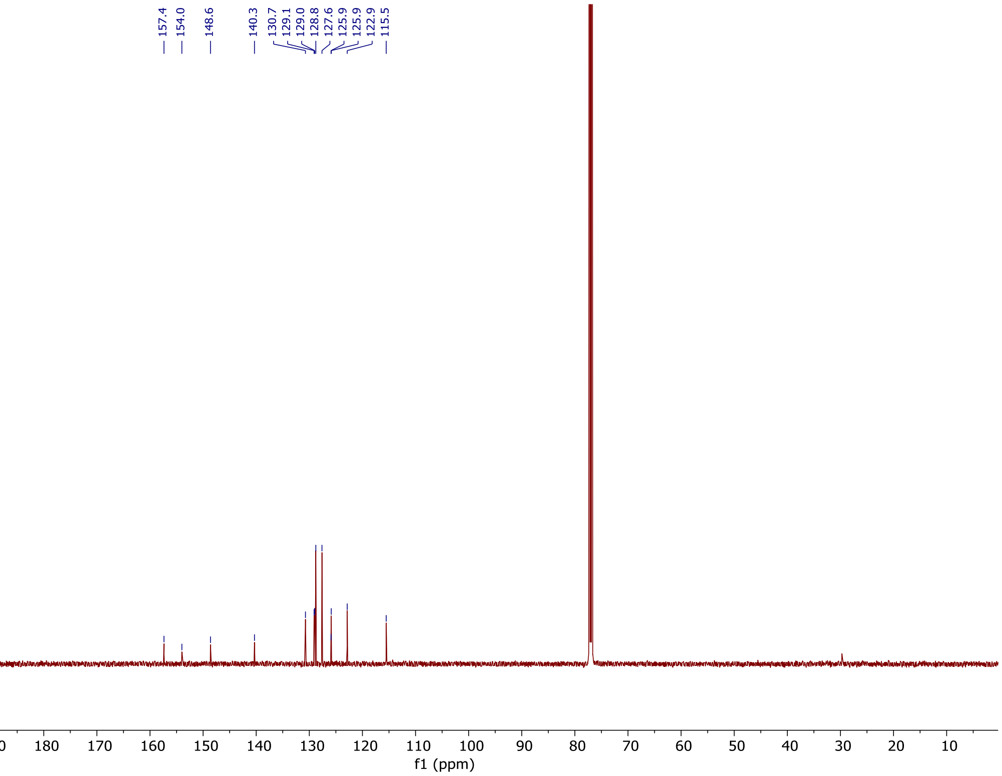

Compound **3-d$_{11}$** ^{2}H-NMR

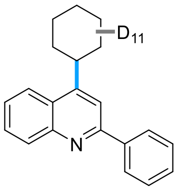

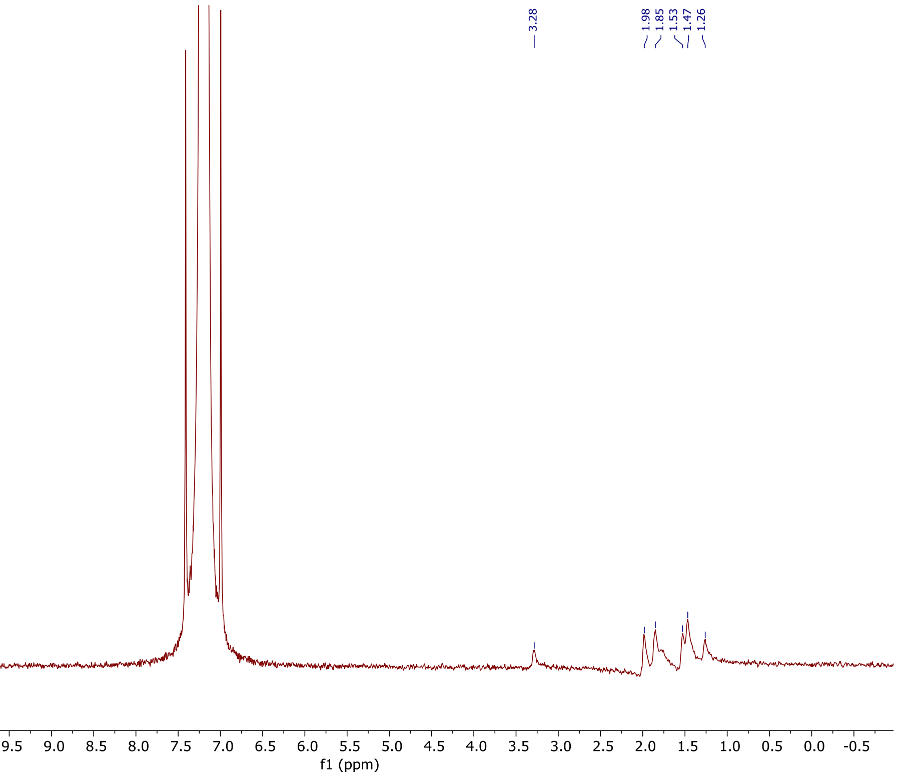

Supplement: Supplementary file 5 — Supplementary Data 3 [file 42004_2023_947_MOESM5_ESM.pdf]
